# Antiparasitic Activity of Sulfur- and Fluorine-Containing Bisphosphonates against Trypanosomatids and Apicomplexan Parasites

**DOI:** 10.3390/molecules22010082

**Published:** 2017-01-04

**Authors:** Tamila Galaka, Mariana Ferrer Casal, Melissa Storey, Catherine Li, María N. Chao, Sergio H. Szajnman, Roberto Docampo, Silvia N. J. Moreno, Juan B. Rodriguez

**Affiliations:** 1 Departamento de Química Orgánica and UMYMFOR (CONICET–FCEyN), Facultad de Ciencias Exactas y Naturales, Universidad de Buenos Aires, Pabellón 2, Ciudad Universitaria, C1428EHA Buenos Aires, Argentina; galakatamila@qo.fcen.uba.ar (T.G.); mferrer@qo.fcen.uba.ar (M.F.C.); nchao@qo.fcen.uba.ar (M.N.C.); shs@qo.fcen.uba.ar (S.H.S.); 2 Center for Tropical and Emerging Global Diseases and Department of Cellular Biology, University of Georgia, Athens, GA 30602, USA; mtayl04@uga.edu (M.S.); catli@uga.edu (C.L.); rdocampo@uga.edu (R.D.); smoreno@uga.edu (S.N.J.M.)

**Keywords:** bisphosphonates, farnesyl diphosphate synthase, *Trypanosoma cruzi*, *Toxoplasma gondii*, Chagas disease, toxoplasmosis, antiparasitic agents

## Abstract

Based on crystallographic data of the complexes 2-alkyl(amino)ethyl-1,1-bisphosphonates–*Trypanosoma cruzi* farnesyl diphosphate synthase, some linear 1,1-bisphosphonic acids and other closely related derivatives were designed, synthesized and biologically evaluated against *T. cruzi*, the responsible agent of Chagas disease and against *Toxoplasma gondii*, the etiologic agent of toxoplasmosis and also towards the target enzymes farnesyl pyrophosphate synthase of *T. cruzi* (*Tc*FPPS) and *T gondii* (*Tg*FPPS), respectively. The isoprenoid-containing 1,1-bisphosphonates exhibited modest antiparasitic activity, whereas the linear α-fluoro-2-alkyl(amino)ethyl-1,1-bisphosphonates were unexpectedly devoid of antiparasitic activity. In spite of not presenting efficient antiparasitic activity, these data turned out to be very important to establish a structural activity relationship.

## 1. Introduction

American Trypanosomiasis (Chagas disease) is a chronic parasitosis caused by the kinetoplastid parasite *Trypanosoma cruzi* and is the largest parasitic disease burden of the Americas [1,2]. Isoprenoids are essential compounds of the cellular machinery of all organisms due to their roles in a variety of biological processes. Several enzymes of this pathway in *T. cruzi*, involved in the synthesis of sterols [1,2] and farnesyl diphosphate [3] have been reported to be excellent targets against pathogenic parasites. Despite their structural and functional variety, all isoprenoids derive from a common precursor: isopentenyl diphosphate (IPP), and its isomer, dimethylallyl diphosphate (DMAPP). In *T. cruzi*, IPP is synthesized only via the so-called mevalonate pathway, which has the 3-hydroxy-3-methylglutaryl-CoA (HMG-CoA) reductase as the key regulatory enzyme [4]. In Apicomplexan parasites such as *Toxoplasma gondii*, the responsible agent of toxoplasmosis, IPP is synthesized by the prokaryotic-type 1-deoxy-d-xylulose-5-phosphate (DOXP) pathway [5], which localizes to the apicoplast and is essential [6]. *T. gondii* possesses a bifunctional farnesyl diphosphate synthase (FPPS)/geranyl diphosphate synthase (GGPPS) (*Tg*FPPS) that is able to catalyze the formation of both FPP and GGPP [7,8]. It is very relevant to state that it is possible to inhibit the host mevalonate pathway and the *Tc*FPPS or DOXP pathway of *T. gondii* synergistically [9]. Certainly, the use of two approved and widely used drugs, zoledronic acid and atorvastatin exhibited a strong synergism in the inhibition of *T. gondii* growth [9]. In addition, *Tc*FPPS has 50% identity with *Tg*FPPS, therefore, it would be possible to access very selective inhibitors of the activity of each enzyme [10].

**Figure 1:**
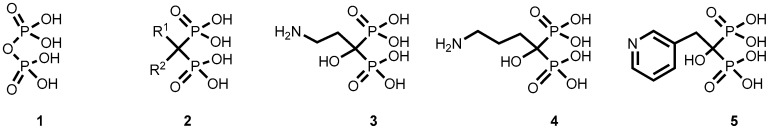
Chemical structures of pyrophosphoric acid, general formula of bisphosphonic acids and representative FDA-approved bisphosphonates clinically employed for the treatment of different bone disorders.

Bisphosphonates (**2**) are metabolically stable pyrophosphate analogs (**1**) in which a methylene group replaces the oxygen atom bridge between the two phosphorus atoms of the pyrophosphate moiety. Substitution at the carbon atom with different side chains has generated a large family of compounds. Several bisphosphonates are potent inhibitors of bone resorption and are in clinical use for the treatment and prevention of osteoporosis, Paget’s disease, hypercalcemia, tumor bone metastases, and other bone diseases [11,12]. Selective action on bone is based on the binding of the bisphosphonate moiety to the bone mineral [13]. Apart from their ability as inhibitors of bone resorption, bisphosphonates have turned out to be antibacterial agents [13], anticancer agents [14,15], selective inhibitors of acid sphingomyelinase [16], and stimulators of γδ T cells [17]. Interestingly, these compounds behave as antiparasitic agents as well [10,18]. Aminobisphosphonates such as pamidronate (**3**), alendronate (**4**), and risedronate (**5**) were first found to be effective in the inhibition of *T. cruzi* in vitro and in vivo without toxicity to the host cells (Figure 1) [19]. In addition, the scope of these compounds was broadened by finding that some bisphosphonates were also growth inhibitors of *T. gondii*, *T. brucei rhodesiense*, *Leishmania donovani* and *Plasmodium falciparum* [20,21,22]. In vivo testing in mice has shown that risedronate can have a significantly marked protective effect in mice infected by *T. cruzi* [23,24].

Pioneering work of Rogers et al. had shown that apoptosis induced by bisphosphonates in J774 macrophages was associated with the inhibition of post-translational prenylation of proteins such as Ras and that this effect can be reverted by the addition of components of the mevalonate pathway such as mevalonic acid, FPP, and GGPP [25]. Many years later, it was established that the primary molecular target of bisphosphonates was farnesyl diphosphate synthase (FPPS) [26]. Selective inhibition of parasite growth may be possible due to the incidence of very fast proliferation of parasites together with the presence of calcium polyphosphate stores in the acidocalcisomes, which mimic the calcium hydroxyapatite surfaces found in bone, facilitating bisphosphonate drug uptake [27,28].

**Figure 2:**
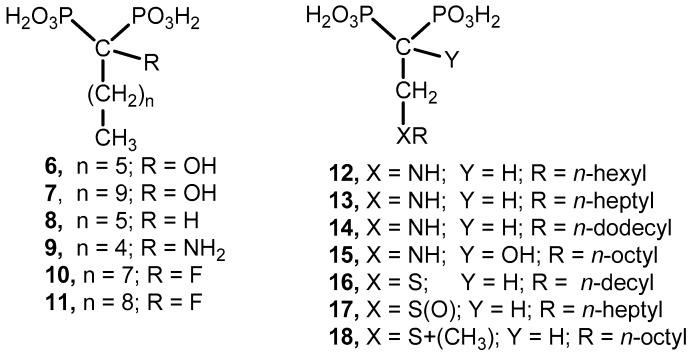
Chemical structures of representative lineal bisphosphonic acids as putative antiparasitic agents.

A relevant achievement of our laboratory was the finding that linear bisphosphonates became efficient antiparasitic agents [29,30,31] where, in most of them, the hydroxyl group at the C-1 position, usually found in those bisphosphonates currently employed in the treatment of bone disorders, is absent [26]. Compounds **6**–**9** were the first examples of linear bisphosphonates, which turned out to be antiparasitic agents (trypanosomatids and Apicomplexan parasites) targeting parasitic FPPS [7,29,30,31,32]. For example, **6** is a moderate growth inhibitor of intracellular *T. cruzi* [29] and also against tachyzoites of *T. gondii* [7,32], whereas **7** is effective against *P. falciparum* [7]. Compound **8** is practically devoid of antiparasitic activity [30], whereas **9** shows similar cellular activity against *T. cruzi* [31]. In addition, α-fluoro-1,1-bisphosphonates **10** and **11** are neither effective against amastigotes of *T. cruzi* nor the target enzyme *Tc*FPPS, but they are extremely potent inhibitors of the enzymatic activity of *Tg*FPPS [33]. Of paramount concern are 2-alkyl(amino)ethyl-1,1-bisphosphonates, which are potent inhibitors of *T. cruzi* proliferation acting against the target enzyme (*Tc*FPPS) at the low nanomolar range [34,35]. Undoubtedly, compounds **12**–**14** emerge as pertinent members of this type of bisphosphonates. For example, **12** is considerably more potent than the well-known antiparasitic agent **WC-9** against *T. cruzi* (amastigotes) [34], under the same assays conditions [36]; while **13** is a very effective inhibitor of the enzymatic activities of *Tc*FPPS and of *Tg*FPPS [34]. Moreover, **14**, the bisphosphonate bearing a long aliphatic chain, is an effective growth inhibitor of *T. cruzi* [35]. Compound **15** is an interesting example of linear bisphosphonates synthesized by an attempt to improve structures **12**–**14**. Compound **15** had been designed based on the fact that the presence of electron withdrawing group (HO-) at C-1 would enhance the ability to coordinate Mg^2+^, would increase p*K*_a_ and also by the fact that most bisphosphonates clinically in use have this functionality at C-1 [37]. Compound **15** is devoid of activity against *T. cruzi* growth and *Tc*FPPS, but exhibited a potent and selective inhibition of the enzymatic activity towards *Tg*FPPS [37]. Linear sulfur-containing bisphosphonates are interesting examples of selective anti-*Toxoplasma* agents as it is the case of **16** and **17** [38]. Certainly, **16** is a potent inhibitor of *T. gondii* proliferation. This cellular activity was associated with a potent action against the target enzyme *Tg*FPPS [38], while **17** is an unusually potent inhibitor towards *Tg*FPPS [38]. The methylsulfonium derivative **18** has proven to be a moderate growth inhibitor against both *T. cruzi* and *T. gondii* cells, but a very potent inhibitor of the enzymatic activity towards the target enzymes *Tc*FPPS and *Tg*FPPS (Figure 2; Table 1) [38].

## 2. Rationale

**Figure 3:**
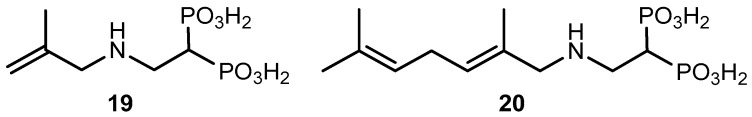
Chemical structures of the designed target molecules based on crystallographic and molecular modeling studies.

Prospects in antiparasitic (antileishmanial, antitrypanosomal, and Apicomplexan) drug research have changed substantially since the availability of the crystal structure of several complexes of 2-alkylaminoethyl-1,1-bisphosphonates with *T. cruzi* farnesyl diphosphate synthase [39]. These X-ray crystallographic studies on the interaction of inhibitor **13** with *Tc*FPPS (**13**–*T*cFPPS) [39,40] have indicated that the nitrogen atom does not coordinate the Mg^2+^ present at the active site of the target enzyme [40]. The tridentate coordination structure is circumvented to the hydroxyl groups bonded to the phosphorus atoms either for 2-alkylaminoethyl- or 1-hydroxy-1,1-bisphosphonates [41,42]. X-ray structures and thermodynamic data of the complexes *Tc*FPPS with **12** and **13** indicated that these compounds bind to the allylic site of *Tc*FPPS having the hydrophobic alkyl chain buried in the cavity that binds the isoprenoid chain of the substrate [39]. The structures of **19** and **20** were envisioned by molecular modeling studies, which suggested that these compounds would increase affinity substantially (Figure 3) [39].

On the other hand, α-fluoro-2-alkylaminoethyl-1,1-bisphosphonates such as **54**–**61** are also very interesting structural variations taking into account the selective and potent action against *Tg*FPPS exhibited by 1-fluoro-1,1-bisphosphonates [33] and also the selective and efficient biological activity exhibited by 2-alkylaminoethyl-1,1-bisphosphonates towards both *T. cruzi* cells and *Tc*FPPS [34,35,39].

## 3. Results and Discussion

**Scheme 1:**
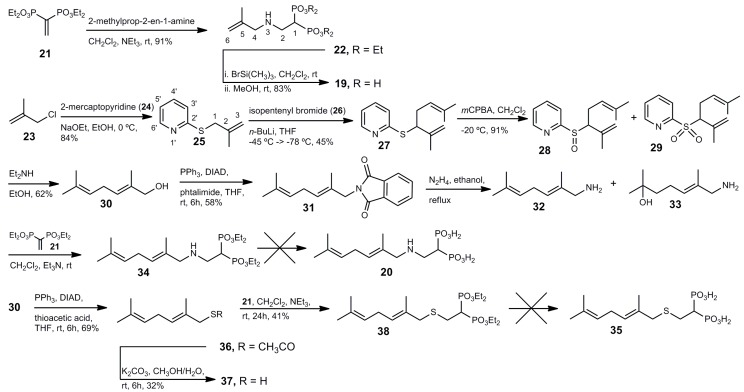
Synthetic approach to access the envisioned putative inhibitors of farnesyl pyrophosphate synthase.

Preparation of the allylamine analog of the lead chemical structures **12**–**14** (compound **19**) was performed according to already published methods [34,35]. Succinctly, the Michael-type acceptor **21** [43], which was synthesized from commercially available tetraethyl methylene-1,1-bisphosphonate, was reacted with 2-methylallylamine in methylene chloride to produce the Michael adduct **22**. This compound was hydrolyzed via the method developed by McKenna [44,45] by treatment with bromotrimethylsilane in methylene chloride followed by digestion with methanol [38] to yield the desired compound **19** (Scheme 1). The synthesis of the desired compound **20** was attempted following a slightly modified method previously described by Mori and Ueda (Scheme 1) [46]. Therefore, methallyl chloride (**23**) was coupled with 2-mercaptopyridine (**24**) to give the corresponding sulfide **25** in 84% yield, which on reaction with *n*-buthyllithium and isopentenyl bromide at low temperature gave **27** in 45% yield. This compound was treated with *m*-chloroperbenzoic acid at −20 °C to produce the corresponding sulfoxide **28** as main product and a small amount of the sulfone **29**. Basic treatment of **28** with diethylamine gave rise to the rearranged allyl alcohol **30**, which under Mitsunobu-type conditions by treatment with triphenylphosphine, diisopropyl azodicarboxylate and phtalimide [47] yielded amine precursor **31**. Cleavage of the phtalimide protecting group by treatment with hidrazine in refluxing ethanol [47] gave rise to the desired amine **32** as a main product and the hydrated product **33** as a consequence of the reaction work-up. This compound was reacted with the Michael acceptor **21** to produce **34** in good yields. Unexpectedly, it was not possible to carry out deprotection of the tetraethyl ester functionality. The presence of two double bonds indicated that hydrolysis by treatment with concentrated hydrochloric acid was an unlikely transformation [43]. The most commonly employed deprotection protocol, that is, reaction with bromotrimethylsilane followed by digestion with methanol [43], underwent a retro-Michael reaction yielding the corresponding free amine **32** together with the Michael acceptor **21**. Actually, this retro-Michael reaction could take place during or after silylation; therefore, it is more likely that **21** or its Michael adduct with amine (**34**) upon solvolysis there will probably be no silyl ester, just free acid, product of solvolysis of silyl ester (after/before Michael addition). Hydrolysis in the presence of 2,4,6-collidine as described for other closely related compounds [48] was not satisfactory to avoid this behavior. The silyl ester of **21** has been described as a suitable Michael acceptor for a number of soft nucleophiles [49,50]. However, in our hands, we were not able to prepare addition product **20** by reaction between the silyl ester of **21** and the allylic amine **32**. Evidently, the presence of the amino group at the C-3 position is not compatible with the available methodology to perform this transformation and new chemistry has to be developed to encourage this task. Efforts in these aspects are currently being pursued in our laboratory. Synthetic efforts to prepare the sulfur-containing analog of the envisioned compound **20**, namely, **35** are outlined in Scheme 1 starting form allylic alcohol **30**. Thus, **30** was treated under Mitsunobu-type conditions with triphenylphosphine, diisopropyl azodicarboxylate and thioacetic acid [51] to yield the thiolacetate **36**, which was hydrolyzed by treatment with potassium carbonate [51] to produce the corresponding mercaptan **37** in low but reproducible yields. Michael addition of **37** on **21** gave rise to **38**, which could not be converted into the envisioned title compound **35**.

**Scheme 2:**
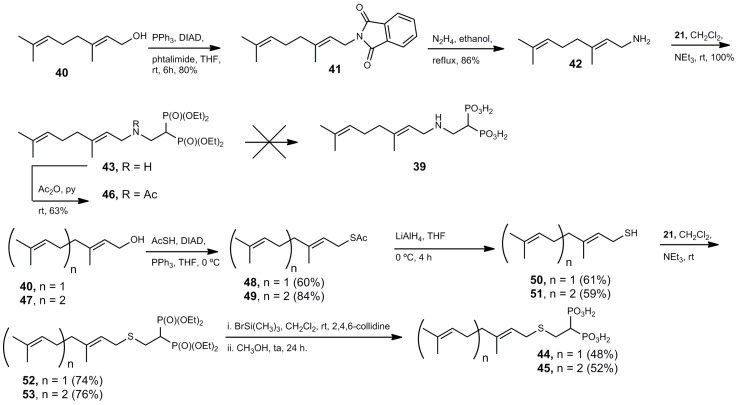
Synthetic approach for the preparation of geranyl and farnesyl containing 1,1-bisphosphonates.

Compounds **39**, **44** and **45** are isoprenoid derivatives, which are structurally related to **19**, **20**, and **35**. These compounds, envisioned as optimized inhibitors of the target enzyme *Tc*FPPS, are outlined in Scheme 2. Then, following a similar approach, geraniol (**40**) was converted into **41** by treatment with triphenylphosphine, diisopropyl azodicarboxylate and phtalimide in 80% yield [51]. Synthesis of the free amine **42** followed by conjugate addition on **21** gave **43** in very good yields. Neither **43** nor its acetyl derivative **46** was able to produce the desired hypothetical desired compound **39** employing the currently known methods of phosphonates ester hydrolysis. Certainly, phosphonate esters bearing an amino group at the C-3 position requires a new method for hydrolysis reaction to occur.

On the other hand, **44** and **45** were straightforwardly prepared starting from geraniol (**40**) and farnesol (**47**), respectively. These alcohols under Mitsunobu type conditions [47] gave thiol acetates **48** and **49** in good yields that on treatment with lithium aluminum hydride produced the corresponding mercaptans **50** and **51**. On reaction with **21**, both compounds were converted into precursors **52** and **53**, which after final treatment of bromotrimethylsilane and digestion with methanol yielded the title compounds **44** and **45**.

**Scheme 3:**
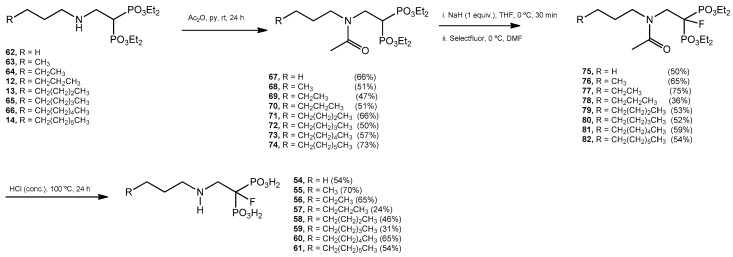
Synthetic approach to access 2-alkylaminoethyl-1-fluoro-1,1-bisphosphonates.

The synthesis of 1-fluoro-2-alkylaminoethyl-1,1-bisphosphonate derivatives **54**–**61** was accomplished following a similar approach with other closely related compounds [33] employing Selectfluor (1-chloromethyl-4-fluoro-1,4-diazobicyclo[2.2.2]octane bis(tetrafluoroborate), F-TEDA BF_4_) as fluorinating agent as shown in Scheme 3 [52,53]. The presence of the amino group interfered with Selectfluor; therefore, protection of this group was required for the fluorination reaction to occur. Then, each tetraethyl 2-alkylaminoethyl-1,1-bisphosphonate **12**–**14**, **62**–**66** was reacted with acetic anhydride in pyridine to produce the corresponding amides **67**–**74**, which treated with sodium hydride followed by addition of Selectfluor [54] yielded the respective monofluoro bisphosphonate derivatives **75**–**82**. Hydrolysis of these tetraethyl esters by treatment with concentrated hydrochloric acid generated the title compounds **54**–**61** (Scheme 3).

Biological results are shown in Table 2. Most of the envisioned compounds resulted to be devoid of antiparasitic activity. Compound **19** was inactive against *T. cruzi* and *T. gondii* cells but exhibited a marginal activity against the target enzymes. Sulfur-containing isoprenoid 1,1-bisphosphonates **44** and **45** were also devoid of cellular activity against *T. cruzi* and *T. gondii.* Biological evaluation of α-fluoro-2-alkyl(amino)ethyl-1,1-bisphosphonates **54**–**61** was very surprising. Certainly, in spite of having a small structural variation in comparison to **12**–**14**, which are very effective growth inhibitors of *T. cruzi* cells targeting *Tc*FPPS at the low nanomolar range, most of these fluorine derivatives, with the exception of **59**, exhibited vanishing antiparasitic action. Bisphosphonate **59** was a growth inhibitor of tachyzoites of *T. gondii* at the low micromolar range (ED_50_ = 4.0 μM). It is worth mentioning the work on other bisphosphonates on bone mineral affinity and mevalonate pathway inhibition evaluations performed by McKenna et al. [55], where there is a drop of activity between α-hydroxy and α-fluoro analogs, but desoxy and fluoro analogs behave in a similar way. The derivatives that bear long chain length such as **60** and **61** turned out to be toxic compounds against Vero cells; therefore, they were not further analyzed.

In summary, the modest activity shown by these new families of bisphosphonates bring about important insights concerning structure–activity relationship. The simple replacement of the hydrogen atom by a fluorine atom had a dramatic effect on the biological activity, giving rise to inactive compounds. Effects in optimizing bisphosphonate structures are presently being pursued in our laboratory.

## 4. Experimental Section

The glassware used in air- and/or moisture-sensitive reactions was flame-dried and reactions were carried out under dry argon. Unless otherwise noted, chemicals were commercially available and used without further purification. Solvents were distilled before use. Dichloromethane was distilled from phosphorus pentoxide. Nuclear magnetic resonance spectra were recorded with a Bruker AM-500 MHz spectrometer. The ^1^H NMR spectra are referenced with respect to the residual CHCl_3_ proton of the solvent CDCl_3_ at δ = 7.26 ppm. Coupling constants are reported in Hz. ^13^C NMR spectra were fully decoupled and are referenced to the middle peak of the solvent CDCl_3_ at δ = 77.0 ppm. ^31^P NMR spectra are referenced with respect to the peak of 85% H_3_PO_4_ as external reference. For comparative purposes, all NMR spectra acquired in D_2_O for free bisphosphonic acids were performed at the same conditions. Splitting patterns are designated as s, singlet; d, doublet; t, triplet; q, quadruplet; dd, double doublet, etc. Melting points were determined with a Fisher–Johns apparatus and are uncorrected. High-resolution mass spectra were obtained using a Bruker micrOTOF-Q II spectrometer, which is a hybrid quadrupole time of flight mass spectrometer with MS/MS capability. Analytical TLC was performed on commercial 0.2 mm aluminum-coated silica gel plates (F254) and visualized by 254 nm UV or immersion in an aqueous solution of (NH_4_)_6_Mo_7_O_24_·4H_2_O (0.04 M), Ce(SO_4_)_2_ (0.003 M) in concentrated H_2_SO_4_ (10%).

As judged from the homogeneity of the ^1^H, ^13^C, ^31^P NMR spectra and HPLC analyses of the title compounds employing a Beckmann Ultrasphere ODS-2 column 5 μM, 250 × 10 mm eluting with water–acetonitrile (9:1) at 3.00 mL/min with a refractive index detector indicated a purity >97%.

*Tetraethyl 1-[(2-methylall-1-ylamino)ethyl] 1,1-bisphosphonate* (**22**). A solution of compound **21** (300 mg, 1.0 mmol) in anhydrous methylene chloride (10 mL) was treated with triethylamine (121.2 mg, 168 μL, 1.2 mmol) and 2-methylallylamine hydrochloride (129 mg, 1.2 mmol) under an argon atmosphere. The reaction mixture was stirred at room temperature overnight. The solvent was evaporated and the residue was purified by column chromatography (silica gel) employing hexane–EtOAc (17:3) as eluent to produce 340 mg (91% yield) of **22** as a colorless oil: ^1^H NMR (500.13 MHz, CDCl_3_) δ 1.34 (t, *J* = 7.1 Hz, 12H, H-2′), 1.75 (s, 3H, C*H*_3_ at C-5), 2.66 (tt, *J* = 23.5, 5.8 Hz, 1H, H-1), 3.11 (dt, *J* = 16.9, 5.8 Hz, 2H, H-2), 3.17 (s, 2H, H-4), 4.19 (m, 8H, H-1′), 4.83 (br s, 1H, H-6_a_), 4.89 (br s, 1H, H-6_b_); ^13^C NMR (125.77 MHz, CDCl_3_) δ 16.4 (dd, *J* = 6.4, 2.6 Hz, C-2′), 20.6 (*C*H_3_ at C-5), 37.7 (t, *J* = 132.5 Hz, C-1), 44.9 (t, *J* = 4.3 Hz, C-2), 55.1 (C-4), 62.6 (dd, *J* = 32.6, 6.5 Hz, C-1′), 111.0 (C-6) 143.3 (C-5); ^31^P NMR (202.46 MHz, CDCl_3_) δ 22.73. HRMS (ESI) calcd. for (C_14_H_32_O_6_NP_2_) [M + H]^+^ 372.1707; found 372.1712.

*1-[(2-Methylall-1-ylamino)ethyl] 1,1-bisphosphonic Acid* (**19**). To a solution of the tetraethyl ester **22** (320 mg, 0.86 mmol) in anhydrous methylene chloride was added dropwise trimethylsilyl bromide (1.276 g, 1.10 mL, 8.6 mmol) under an argon atmosphere. The reaction mixture was stirred at room temperature for 48 h. After cooling at 0 °C, anhydrous methanol (10 mL) was added, and the resulting mixture was allowed to reach room temperature and stirred for 24 h. The solution was then concentrated under reduced pressure. The residue was dissolved in dry methanol (10 mL) and subsequently concentrated under reduced pressure twice. The solvent was evaporated and the residue was crystallized from ethanol–water to yield 183 mg (83% yield) of pure **19** as a white solid: ^1^H NMR (500.13 MHz, D_2_O) δ 1.74 (s, 3H, C*H*_3_ at C-5), 2.44 (m, 1H, H-1), 3.36 m, 2H, H-2), 3.59 (s, 2H, H-4), 5.03 (s, 1H, H-6_a_), 5.12 (s, 1H, H-6_b_); ^13^C NMR (125.77 MHz, D_2_O) δ 19.5 (*C*H_3_ at C-5), 35.7 (t, *J* = 128.8 Hz, C-1), 44.3 (C-2), 52.5 (C-4), 117.4 (C-6) 135.9 (C-5); ^31^P NMR (202.46 MHz, D_2_O) δ 16.96. HRMS (ESI) calcd. for (C_6_H_15_O_6_NP_2_Na) [M + Na]^+^ 282.0272; found 282.0268.

*2-((2-Methylallyl)thio)pyridine* (**25**). To a solution of sodium ethoxide (prepared from sodium (620 mg, 27.0 mmol) and anhydrous ethanol (20 mL)) was added 2-mercaptopyridine (**24**; 3.00 g, 27.0 mmol) at 0 °C. The mixture was stirred for 15 min. Then, methallyl chloride (**23**; 3.06 mL, 2.81 g, 31.0 mmol) was added and the mixture was stirred at 0 °C for 3 h. The solvent was evaporated and the residue was partitioned between water (50 mL) and ethyl ether (50 mL). The organic phase was washed with an aqueous 5% solution of sodium hydroxide (2 × 30 mL) and water (2 × 30 mL). The organic layer was dried (MgSO_4_) and the solvent was evaporated to produce 3.743 g (84% yield) of **25** as a colorless oil: ^1^H NMR (500.13 MHz, CDCl_3_) δ 1.85 (s, 3H, C*H*_3_ at C-2), 3.86 (s, 2H, H-1), 4.86 (t, *J* = 1.4 Hz, 1H, H-3_a_), 5.02 (q, *J* = 0.9 Hz, 1H, H-3_b_), 6.97 (ddd, *J* = 7.3, 4.9, 1.0 Hz, 1H, H-5′), 7.18 (dt, *J* = 8.1, 0.9 Hz, 1H, H-3′), 7.47 (ddd, *J* = 7.9, 7.5, 1.8 Hz, 1H, H-4′), 8.42 (ddd, *J* = 4.9, 1.7, 0.9 Hz, 1H, H-6′); ^13^C NMR (125.77 MHz, CDCl_3_) δ 21.5 (C*H*_3_ at C-2), 37.1 (C-3), 113.7 (C-1), 119.5 (C-5′), 122.3 (C-3′),135.9 (C-4′), 141.3 (C-2), 149.4 (C-6′), 159.0 (C-2′).

*2-((2,6-Dimethylhepta-1,5-dien-3-yl)thio)pyridine* (**27**). A solution of **25** (3.74 g, 22.7 mmol) in anhydrous tetrahydrofuran (50 mL) cooled at −50 °C was treated dropwise with an 1.1 M solution of *n*-butyllithium in hexane (22 mL). The reaction mixture turned deep-red and was cooled to −78 °C. Then, to the resulting mixture a solution of isopentenyl bromide (**26**; 2.8 mL, 3.61 g, 24.2 mmol) in anhydrous tetrahydrofuran (10 mL) was added dropwise. The reaction mixture was stirred at −78 °C for 1 h. Then, the mixture was allowed to reach room temperature gradually. The reaction was quenched by addition of a saturated solution of ammonium chloride (50 mL). The organic layer was separated and the aqueous phase was extracted with ethyl ether (50 mL). The combined organic layers were dried (MgSO_4_) and the solvent was evaporated to give 2.384 g of **27** (45% yield) as a colorless oil: ^1^H NMR (500.13 MHz, CDCl_3_) δ 1.64 (s, 3H, C*H*_3_ at C-6), 1.68 (s, 3H, H-7), 1.82 (s, 3H, C*H*_3_ at C-2), 2.43 (m, 1H, H-4_a_), 2.56 (m, 1H, H-4_b_), 4.40 (dd, *J* = 8.6, 6.6 Hz, 1H, H-3), 4.85 (br s, 1H, H-1_a_), 4.98 (br s, 1H, H-1_b_), 5.15 (tt, *J* = 7.0, 1.2 Hz, 1H, H-5), 6.92 (ddt, *J* = 7.3, 5.1, 1.0 Hz, 1H, H-5′), 7.17 (ddd, *J* = 8.0, 1.9, 1.0 Hz, 1H, H-3′), 7.46 (m, 1H, H-4′), 8.42 (ddd, *J* = 4.0, 1.8, 0.9 Hz, 1H, H-6′); ^13^C NMR (125.77 MHz, CDCl_3_) δ 18.0 (*C*H_3_ at C-6), 19.5 (*C*H_3_ at C-2), 25.8 (C-7), 32.1 (C-4), 51.2 (C-3), 113.3 (C-1), 119.6 (C-5′), 121.2 (C-3′), 123.2 (C-5), 133.5 (C-6), 135.9 (C-4′), 144.2 (C-2), 149.4 (C-6′), 159.1 (C-2′). HRMS (ESI) calcd. for C_14_H_20_NS [M + H]^+^ 234.1316; found 234.1319.

*2-((2,6-Dimethylhepta-1,5-dien-3-yl)sulfinyl)pyridine* (**28**) *and 2-((2,6-dimethylhepta-1,5-dien-3-yl)sulfonyl)pyridine* (**29**). To a solution of **27** (5.91 mg, 25.3 mmol) in methylene chloride (100 mL) cooled at −25 °C under an argon atmosphere was added dropwise a solution of *m*-chloroperbenzoic acid (77% purity, 5.61 g, 26.6 mmol) in methylene chloride (100 mL). The mixture was stirred at −25 °C for 1 h. Then, the temperature was allowed to reach room temperature. Then, the mixture was extracted with a saturated solution of potassium carbonate (2 × 75 mL) and water (2 × 75 mL). The organic phase was dried (MgSO_4_), and the solvent was evaporated. The residue was purified by column chromatography (silica gel) eluting was hexane–EtOAc (19.1) to give 5.68 g of **28** (91% yield) and 403 mg of **29** (6% yield) as colorless oils: Compound **28** (two diastereomers): ^1^H NMR (500.13 MHz, CDCl_3_) δ 1.42 (s, 3H, C*H*_3_ at C-6), 1.55 (s, 3H, C*H*_3_* at C-6), 1.50 (s, 3H, H-7), 1.70 (s, 3H, H-7*), 1.73 (s, 3H, C*H*_3_ at C-2), 1.88 (s, 3H, C*H*_3_* at C-2), 2.25 (m, 1H, H-4_a_), 2.60 (m, 1H, H-4_a_*), 2.47 (m, 1H, H-4_b_), 2.85 (m, 1H, H-4_b_*), 3.46 (dd, *J* = 8.2, 7.9 Hz, 1H, H-3), 3.57 (dd, *J* = 10.3, 4.9 Hz, 1H, H-3*), 4.48 (br s, 1H, H-1_a_), 4.77 (p, *J* = 1.5 Hz, 1H, H-1_a_*), 4.83 (tp, *J* = 7.0, 1.4 Hz, 1H, H-3), 5.03 (br s, 1H, H-1_b_), 5.13 (p, *J* = 1.5 Hz, 1H, H-1_b_*), 5.19 (m, 1H, H-3*), 7.31 (m, 1H, aromatic proton), 7.88–7.97 (m, 2H, aromatic proton), 8.60–8.64 (m, 1H, aromatic proton); compound **29** ^1^H NMR (500.13 MHz, CDCl_3_) δ 1.57 (s, 3H, C*H*_3_ at C-6), 1.67 (s, 3H, H-7), 1.82 (br s, 3H, C*H*_3_ at C-2), 2.48 (m, 1H, H-4_a_), 2.72 (m, 1H, H-4_b_), 4.21 (dd, *J* = 11.2, 4.2 Hz, 1H, H-3), 4.84 (br s, 1H, H-1_a_), 4.88 (t, *J* = 7.0 Hz, 1H, H-5), 5.02 (br s, 1H, H-1_b_), 7.51 (ddd, *J* = 7.7, 4.7, 1.1 Hz, 1H, H-5′), 7.91 (dt, *J* = 7.7, 1.7 Hz, 1H, H-4′), 8.02 (d, *J* = 7.9 Hz, 1H, H-3′), 8.74 (ddd, *J* = 4.6, 1.6, 0.8 Hz, 1H, H-6′); ^13^C NMR (125.77 MHz, CDCl_3_) δ 17.9 (*C*H_3_ at C-6), 20.2 (*C*H_3_ at C-2), 24.6 (C-7), 25.7 (C-4), 67.7 (C-3), 118.4 (C-1), 120.8 (C-5′), 123.4 (C-3′), 127.1 (C-5), 135.1 (C-6), 136.4 (C-2), 137.7 (C-4′), 150.1 (C-6′), 156.6 (C-2′).

*(E)-2,6-Dimethylhepta-2,5-dien-1-ol* (**30**). A solution of **28** (5.65 g, 22.7 mmol) in methanol (100 mL) was treated with diethylamine (100 mL) and the resulting mixture was stirred at room temperature overnight. The solvent was evaporated and the residue was partitioned between ethyl ether (100 mL) and water (100 mL). The aqueous phase was extracted with ethyl ether (2 × 50 mL). The combined organic layers were washed with 5% hydrochloric acid (2 × 30 mL), a saturated solution of sodium bicarbonate (2 × 30 mL), brine (3 × 30 mL). The organic layer was dried (MgSO_4_) and the solvent was evaporated. The product was purified by column chromatography (silica gel) eluting with a mixture of hexane EtOAc (19:1) to yield 1.973 g (62% yield) of alcohol **30** as a colorless oil: *R*_f_ = 0.51 (hexane–EtOAc, 1:1): ^1^H NMR (500.13 MHz, CDCl_3_) δ 1.64 (s, 3H, C-7), 1.70 (s, 6H, C*H*_3_ at C-2, C*H*_3_ at C-6), 2.73 (t, *J* = 7.2 Hz, 2H, H-4), 4.00 (s, 2H, H-1), 5.11 (tp, *J* = 7.0, 1.3 Hz, 1H, H-3), 5.39 (tq, *J* = 7.3, 1.3 Hz, 1H, H-5); ^13^C NMR (125.77 MHz, CDCl_3_) δ 13.6 (*C*H_3_ at C-2), 17.7 (*C*H_3_ at C-6), 25.7 (C-7), 26.7 (C-4), 69.0 (C-1), 122.4 (C-3), 125.1 (C-5), 132.0 (C-6), 134.5 (C-2).

*(E)-2-(2,6-Dimethylhepta-2,5-dien-1-yl)isoindoline-1,3-dione* (**31**). To a mixture of alcohol **30** (406 mg, 2.9 mmol), triphenylphosphine (911.3 mg, 3.5 mmol), phtalimide (511.2 mg, 3.5 mmol) in anhydrous tetrahydrofuran (10 mL) cooled at 0 °C was added a solution of diisopropyl azodicarboxylate (702.6 mg, 3.5 mmol). The reaction mixture was stirred at room temperature overnight. The solvent was evaporated and the residue was purified by column chromatography (silica gel) eluting with a mixture of hexane–EtOAc (19:1) to give 453.0 mg (58% yield) of pure **31** as a colorless oil: ^1^H NMR (500.13 MHz, CDCl_3_) δ 1.60 (s, 3H, C-7), 1.67 (s, 6H, C*H*_3_ at C-2, C*H*_3_ at C-6), 2.70 (t, *J* = 7.3 Hz, 2H, H-4), 4.20 (s, 2H, H-1), 5.05 (tp, *J* = 7.0, 1.4 Hz, 1H, H-3), 5.36 (tq, *J* = 7.2, 1.3 Hz, 1H, H-5); ^13^C NMR (125.77 MHz, CDCl_3_) δ 14.6 (*C*H_3_ at C-2), 17.7 (*C*H_3_ at C-6), 25.6 (C-7), 26.9 (C-4), 45.0 (C-1), 122.3 (C-3), 123.3 (C-2′), 126.6 (C-5), 129.0(C-1′), 132.0 (C-6), 132.1 (C-2), 133.9 (C-3′), 168.3 (*C*O). HRMS (ESI) calcd. for C_17_H_19_O_2_NNa [M + Na]^+^ 292.1313; found 292.1315.

*(E)-2,6-Dimethylhepta-2,5-dien-1-amine* (**32**) *and (E)-7-amino-2,6-dimethylhept-5-en-2-ol* (**33**). A solution of **31** (400 mg, 1.5 mmol) in ethanol (10 mL) was treated with a solution of 60% hidrazine in water (0.1 mL). The reaction mixture was refluxed for 3 h. The mixture was allowed to cool to room temperature and was filtered. The solvent was evaporated. The residue was treated with a 20% aqueous solution of hydrochloric acid (10 mL) and the mixture was filtered. The pH of the solution was adjusted to 11 by addition of a 1.0 M solution of sodium hydroxide. The aqueous solution was extracted with methylene chloride (3 × 30 mL), dried (MgSO_4_) and the solvent was evaporated. The residue was purified by column chromatography (silica gel) employing a mixture of hexane–EtOAc (4:1) to give 175.4 mg of **32** (84% yield) and 21.2 mg of **33** (9% yield): Compound **32**: ^1^H NMR (500.13 MHz, CDCl_3_) δ 1.64 (s, 3H, C-7), 1.66 (C*H*_3_ at C-2), 1.70 (C*H*_3_ at C-6), 2.72 (t, *J* = 7.1 Hz, 2H, H-4), 3.17 (s, 2H, H-1), 5.11 (tq, *J* = 7.1, 1.2 Hz, 1H, H-3), 5.26 (tt, *J* = 7.1, 1.0 Hz, 1H, H-5); ^13^C NMR (125.77 MHz, CDCl_3_) δ 14.6 (*C*H_3_ at C-2), 17.7 (*C*H_3_ at C-6), 25.7 (C-7), 26.8 (C-4), 49.9 (C-1), 122.6 (C-3), 122.9 (C-5), 131.7 (C-6), 136.3 (C-2). Compound **33**: ^1^H NMR (500.13 MHz, CDCl_3_) δ 1.24 (s, 6H, H-7, C*H*_3_ at C-6), 1.36 (s, 2H, N*H*_2_), 1.55 (m, 2H, H-5), 1.66 (s, 3H, C*H*_3_ at C-2), 2.11 (m, 2H, H-4), 3.17 (s, 2H, H-1), 5.30 (tq, *J* = 7.1, 1.2 Hz, 1H, H-3); ^13^C NMR (125.77 MHz, CDCl_3_) δ 14.5 (*C*H_3_ at C-2), 22.7 (C-4), 29.2 (C-7, *C*H_3_ at C-6), 43.5 (C-5), 49.9 (C-1), 70.8 (C-6), 123.7 (C-3), 136.6 (C-2). HRMS (ESI) calcd. for C_9_H_19_ONNa [M + Na]^+^ 180.1364; found 180.1353.

*Tetraethyl (E)-2,6-dimethylhepta-2,5-dien-1-ylamino ethyl-1,1-bisphosphonate* (**34**). A solution of **21** (327.8 mg, 1.1 mmol) in anhydrous methylene chloride (10 mL) was treated with triethylamine (133.1 mg, 185 μL, 1.3 mmol) and **32** (152.0 mg, 1.1 mmol) as described for the preparation of **22**. Evaporation of the solvent yielded 483.2 mg (100% yield) of pure **34** as a colorless oil: ^1^H NMR (500.13 MHz, CDCl_3_) δ 1.34 (t, *J* = 7.1 Hz, 12H, CH_2_C*H*_3_), 1.63 (s, 3H, C-10), 1.66 (C*H*_3_ at C-5), 1.68 (C*H*_3_ at C-9), 2.64 (tt, *J* = 23.7, 5.9 Hz, 1H, H-1), 2.71 (t, *J* = 6.8 Hz, 2H, H-7), 3.08 (dt, *J* = 16.8, 5.9 Hz, 2H, H-2), 3.12 (s, 2H, H-4), 4.18 (m, 8H, C*H*_2_CH_3_), 5.09 (tt, *J* = 7.1, 1.3 Hz, 1H, H-6), 5.29 (t, *J* = 7.2 Hz, 1H, H-8); ^13^C NMR (125.77 MHz, CDCl_3_) δ 14.6 (*C*H_3_ at C-5), 16.3 (d, *J* = 3.0 Hz, CH_2_*C*H_3_), 16.4 (d, *J* = 2.7 Hz, CH_2_*C′*H_3_), 18.7 (*C*H_3_ at C-9), 24.7 (C-10), 26.9 (C-7), 37.6 (t, *J* = 132.4 Hz, C-1), 44.8 (t, *J* = 4.3 Hz, C-2), 57.0 (C-4), 62.4 (d, *J* = 6.6 Hz, *C*H_2_CH_3_), 62.7 (d, *J* = 6.6 Hz, *C*′H_2_CH_3_), 122.8 (C-6), 125.1 (C-8), 131.5 (C-9), 132.8 (C-5); ^31^P NMR (202.46 MHz, CDCl_3_) δ 22.82 ppm.

*(E)-S-(2,6-Dimethylhepta-2,5-dien-1-yl) ethanethioate* (**36**). To a mixture of alcohol **30** (471.0 mg, 3.4 mmol), triphenylphosphine (1.32 g, 5.04 mmol), thioacetic acid (255.7 mg, 3.4 mmol) in anhydrous tetrahydrofuran (10 mL) cooled at 0 °C was added a solution of diisopropyl azodicarboxylate (1.02 g, 5.04 mmol) as depicted for the preparation of **31**. The product was purified by column chromatography (silica gel) eluting with a mixture of hexane–EtOAc (49:1) to give 459.0 mg (69% yield) of **36** as a colorless oil: *R*_f_ 0.37 (hexane–EtOAc, 19:1); ^1^H NMR (500.13 MHz, CDCl_3_) δ 1.62 (s, 3H, C-7), 1.65 (m, 3H, C*H*_3_ at C-2), 1.68 (d, *J* = 1.1 Hz, 3H, C*H*_3_ at C-6), 2.33 (s, 3H, C*H*_3_C(O)), 2.68 (t, *J* = 6.9 Hz, 2H, H-4), 3.55 (q, *J* = 0.9 Hz, 2H, H-1), 5.11 (tq, *J* = 7.1, 1.2 Hz, 1H, H-3), 5.38 (tq, *J* = 7.3, 1.2 Hz, 1H, H-5); ^13^C NMR (125.77 MHz, CDCl_3_) δ 15.2 (C*H*_3_ at C-2), 17.7 (C*H*_3_ at C-6), 25.7 (C-7), 27.2 (C-4), 30.5 (*C*H_3_C(O)), 38.2 (C-1), 122.2 (C-3), 127.9 (C-5), 130.0 (C-2), 132.1 (C-6), 195.1 (CH_3_C(*O*)).

*(E)-2,6-Dimethylhepta-2,5-dien-1-thiol* (**37**). To a solution of **36** (353.0 mg, 1.78 mmol) in 20.0 mL of a mixture of methanol–water (5:3) was added potassium carbonate (100 mg, 0.72 mmol). The reaction mixture was stirred at room temperature for 6 h. Then, water (50 mL) was added and the mixture was extracted with methylene chloride (3 × 30 mL). The combined organic layers were washed with brine (2 × 30 mL), dried (MgSO_4_) and the solvent was evaporated. The residue was purified by column chromatography (silica gel) eluting with hexane to yield 89 mg (32% yield) of pure **37** as a colorless oil: *R*_f_ 0.57 (hexane–EtOAc, 19:1); ^1^H NMR (300.18 MHz, CDCl_3_) δ 1.40 (t, *J* = 7.8 Hz, 1H, S*H*), 1.63 (s, 3H, C-7), 1.69 (s, 3H, C*H*_3_ at C-2), 1.75 (q, *J* = 1.0 Hz, 3H, C*H*_3_ at C-6), 2.71 (q, *J* = 7.9 Hz, 2H, H-4), 3.12 (d, *J* = 7.8 Hz, 2H, H-1), 5.08 (tp, *J* = 7.1, 1.4 Hz, 1H, H-3), 5.31 (tq, *J* = 7.2, 1.0 Hz, 1H, H-5).

*Tetraethyl (E)-2,6-dimethylhepta-2,5-dien-1-ylthioethyl-1,1-bisphosphonate* (**38**). A solution of **21** (430.3 mg, 1.43 mmol) in anhydrous methylene chloride (15 mL) was treated with triethylamine (147.1 mg, 205 μL, 1.43 mmol) and **37** (224.1 mg, 1.43 mmol) as described for the preparation of **22**. The solvent was evaporated and the residue was purified by column chromatography (silica gel) employing hexane–EtOAc (4:1) as eluent to give 268 mg (41% yield) of **38** as a colorless oil: ^1^H NMR (500.13 MHz, CDCl_3_) δ 1.366 (t, *J* = 7.0 Hz, 6H, CH_2_C*H*_3_), 1.374 (t, *J* = 7.1 Hz, 6H, CH_2_C*H′*_3_), 1.64 (s, 3H, C-10), 1.70 (s, 3H, C*H*_3_ at C-5), 1.74 (m, 3H, C*H*_3_ at C-9), 2.61 (tt, *J* = 23.8, 6.2 Hz, 1H, H-1), 2.74 (t, *J* = 7.6 Hz, 2H, H-7), 2.94 (dt, *J* = 16.2, 6.2 Hz, 2H, H-2), 3.15 (s, 2H, H-4), 4.21 (m, 8H, C*H*_2_CH_3_), 5.11 (tp, *J* = 7.0, 1.6 Hz, 1H, H-6), 5.31 (t, *J* = 7.1 Hz, 1H, H-8); ^13^C NMR (125.77 MHz, CDCl_3_) δ 14.9 (C*H*_3_ at C-5), 16.2 (t, *J* = 3.3 Hz, CH_2_*C*H_3_), 16.4 (dd, *J* = 6.5, 1.3 Hz, CH_2_*C′*H_3_), 17.7 (C*H*_3_ at C-9), 25.6 (C-10), 26.6 (t, *J* = 5.0 Hz, C-2), 27.2 (C-7), 38.7 (t, *J* = 131.7 Hz, C-1), 42.4.0 (C-4), 62.7 (d, *J* = 4.8 Hz, *C*H_2_CH_3_), 62.8 (d, *J* = 6.7 Hz, *C*′H_2_CH_3_), 122.2 (C-6), 127.9 (C-8), 130.0 (C-4), 132.1 (C-9); ^31^P NMR (202.47 MHz, CDCl_3_) δ 21.77 ppm.

*(E)-2-(3,7-Dimethylocta-2,6-dien-1-yl)isoindoline-1,3-dione* (**41**). To a mixture of geraniol **40** (1.00 g, 6.5 mmol), triphenylphosphine (1.70 g, 6.5 mmol), phtalimide (953.3 mg, 6.5 mmol) in anhydrous tetrahydrofuran (15 mL) cooled at 0 °C was added a solution of diisopropyl azodicarboxylate (1.38 g, 6.5 mmol) as described for the preparation of **31**. The residue was purified by column chromatography (silica gel) eluting with hexane–EtOAc (19:1) to give 1.47 g (80 yield) of pure **41** as a colorless oil: *R*_f_ 0.52 (hexane–EtOAc, 4:1); ^1^H NMR (200 MHz, CDCl_3_) δ 1.56 (s, 3H, H-8), 1.63 (s, 3H, C*H*_3_ at C-7), 1.82 (s, 3H, C*H*_3_ at C-3), 2.00 (m, 2H, H-5), 2.08 (p, *J* = 7.0 Hz, 2H, H-4), 4.27 (d, *J* = 7.2 Hz, 2H, H-1), 5.05 (m, 1H, H-6), 5.27 (t, *J* = 7.4 Hz, 1H, H-2).

*(E)-3,7-Dimethylocta-2,6-dien-1-amine* (**42**). A solution of **41** (900 mg, 3.2 mmol) in ethanol (13 mL) was treated with a solution of 60% hidrazine in water (150 µL, 101 mg, 3.2 mmol). The reaction mixture was refluxed for 3 h and was treated as described for the preparation of **32** to yield 422 mg (86% yield) as a colorless oil: ^1^H NMR (500.13 MHz, CDCl_3_) δ 1.60 (s, 3H, H-8), 1.63 (s, 3H, C*H*_3_ at C-7), 1.69 (s, 3H, C*H*_3_ at C-3), 2.00 (m, 2H, H-5), 2.08 (p, *J* = 7.0 Hz, 2H, H-4), 3.27 (d, *J* = 6.8 Hz, 2H, H-1), 5.10 (tp, *J* = 6.7, 1.3 Hz, 1H, H-6), 5.26 (tp, *J* = 6.9, 1.2 Hz, 1H, H-2); ^13^C NMR (125.77 MHz, CDCl_3_) δ 16.1 (*C*H_3_ at C-3), 17.7 (*C*H_3_ at C-7), 25.7 (C-8), 26.5 (C-5), 39.5 (C-4), 39.6 (C-1), 124.1 (C-2), 125.9 (C-6), 131.5 (C-7), 136.3 (C-3).

*Tetraethyl 1-[((E)-3,7-dimethylocta-2,6-diene-1-amino)ethyl] 1,1-bisphosphonate* (**43**). A solution of **21** (400.1 mg, 1.33 mmol) in anhydrous methylene chloride (10 mL) was treated with amine **42** (204.0 mg, 1.33 mmol) as described for the preparation of **22**. Evaporation of the solvent gave 611.1 mg (100% yield) of pure **43** as a colorless oil: ^1^H NMR (500.13 MHz, CDCl_3_) δ 1.34 (t, *J* = 7.1 Hz, 12H, CH_2_C*H*_3_), 1.63 (s, 3H, H-11), 1.66 (s, 3H, C*H*_3_ at C-10), 1.68 (s, 3H, C*H*_3_ at C-6), 2.71 (t, *J* = 6.8 Hz, 4H, H-7, H-8), 2.65 (tt, *J* = 23.7, 5.9 Hz, 1H, H-1), 3.08 (dt, *J* = 16.8, 5.9 Hz, 2H, H-2), 3.13 (br s, 2H, H-4), 5.09 (tt, *J* = 7.1, 1.3 Hz, 1H, H-9), 5.29 (t, *J* = 7.2 Hz, 1H, H-5); ^13^C NMR (125.77 MHz, CDCl_3_) δ 14.6 (*C*H_3_ at C-6), 16.3 (d, *J* = 3.0 Hz, CH_2_*C*H_3_), 16.4 (d, *J* = 2.7 Hz, CH_2_*C*H_3_′), 17.7 (*C*H_3_ at C-10), 25.6 (C-11), 26.9 (C-8), 37.6 (t, *J* = 132.4 Hz, C-1), 44.8 (t, *J* = 4.3 Hz, C-2), 57.0 (C-4), 62.4 (d, *J* = 6.8 Hz, *C*H_2_CH_3_), 62.7 (d, *J* = 6.8 Hz, *C*H_2_CH_3_′), 122.8 (C-5), 125.1 (C-9), 131.5 (C-10), 132.8 (C-6); ^31^P NMR (202.46 MHz, CDCl_3_) δ 22.82 ppm.

*Tetraethyl 1-[((E)-3,7-dimethylocta-2,6-diene-1-acetamido)ethyl] 1,1-bisphosphonate* (**46**). A solution of **43** (227 mg, 0.5 mmol) in anhydrous pyridine (3.0 mL) was treated with acetic anhydride (3.0 mL) and the mixture was stirred at room temperature overnight. The solvent was evaporated and the residue was purified by column chromatography (silica gel) eluting with a mixture of CH_2_Cl_2_–methanol (19:1) to give 312.2 mg (63% yield) of pure **46** as a colorless oil: ^1^H NMR (500.13 MHz, CDCl_3_) δ 1.34 (t, *J* = 7.0 Hz, 6H, CH_2_C*H*_3_), 1.35 (t, *J* = 7.1 Hz, 6H, CH_2_C*H*_3_′), 1.61 (s, 3H, H-11), 1.68 (s, 3H, C*H*_3_ at C-10), 1.69 (s, 3H, C*H*_3_ at C-6), 2.00–2.11 (m, 4H, H-7, H-8), 2.07 (s, 3H, C(O)C*H*_3_), 3.38 (tt, *J* = 22.7, 5.5 Hz, 1H, H-1), 3.71 (ddd, *J* = 14.7, 11.1, 7.4 Hz, 2H, H-2), 4.04 (d, *J* = 6.4 Hz, 2H, H-4), 4.18 (m, 8H, C*H*_2_CH_3_), 5.07 (m, 2H, H-5, H-9); ^13^C NMR (125.77 MHz, CDCl_3_) δ 16.3 (*C*H_3_ at C-6), 16.34 (d, *J* = 6.7 Hz, CH_2_*C*H_3_), 16.37 (d, *J* = 6.6 Hz, CH_2_*C*H_3_′), 17.7 (*C*H_3_ at C-10), 21.9 (C(O)*C*H_3_), 25.7 (C-11), 26.3 (C-8), 34.4 (t, *J* = 129.9 Hz, C-1), 39.5 (C-7), 44.4 (t, *J* = 3.5 Hz, C-2), 48.8 (C-4), 62.4 (d, *J* = 6.8 Hz, *C*H_2_CH_3_), 62.7 (d, *J* = 6.8 Hz, *C*H_2_CH_3_′), 119.7 (C-5), 123.6 (C-9), 131.9 (C-10), 139.4 (C-6), 171.2 (*C*(O)CH_3_); ^31^P NMR (202.46 MHz, CDCl_3_) δ 21.49 ppm.

*(E)-S-(3,7-Dimethylocta-2,6-dien-1-yl) ethanethioate* (**48**). To a mixture of geraniol **40** (1.00 g, 6.5 mmol), triphenylphosphine (2.55 g, 9.7 mmol), thioacetic acid (0.55 mL, 592.2 mg, 7.8 mmol) in anhydrous tetrahydrofuran (20 mL) cooled at 0 °C was added a solution of diisopropyl azodicarboxylate (1.62 mL, 1.66 g, 9.7 mmol) as depicted for the preparation of **31**. The product was purified by column chromatography (silica gel) eluting with hexane–EtOAc (9:1) to give 828.2 mg (60% yield) of **48** as a colorless oil: *R*_f_ 0.39 (hexane–EtOAc, 4:1); ^1^H NMR (500.13 MHz, CDCl_3_) δ 1.59 (s, 3H, H-8), 1.68 (s, 6H, C*H*_3_ at C-3, C*H*_3_ at C-7), 2.00 (m, 2H, H-4), 2.06 (m, 2H, H-4), 2.32 (s, 3H, SC(O)C*H*_3_), 3.54 (d, *J* = 7.9 Hz, 2H, H-1), 5.06 (tp, *J* = 6.8, 1.4 Hz, 1H, H-6), 5.21 (tp, *J* = 7.9, 1.3 Hz, 1H, H-2); ^13^C NMR (125.77 MHz, CDCl_3_) δ 16.2 (*C*H_3_ at C-2), 17.7 (*C*H_3_ at C-6), 25.6 (C-1), 26.4 (C-8), 27.3 (C-5), 30.4 (SC(O)*C*H_3_), 39.5 (C-4), 118.4 (C-2), 123.8 (C-5), 131.7 (C-7), 140.1 (C-3), 196.1 (S*C*(O)CH_3_). HRMS (ESI) calcd. for C_12_H_20_OS [M + Na]^+^ 235.1133; found 235.1138.

*(E)-3,7-Dimethylocta-2,6-diene-1-thiol* (**50**). A solution of **48** (846 mg, 3.98 mmol) in anhydrous tetrahydrofuran (10 mL) cooled at 0 °C was treated with portionwise with lithium aluminum hydride (181 mg, 4.74 mmol). The mixture was stirred at 0 °C for 4 h. The reaction was quenched by addition of ethyl acetate (2.0 mL). Then, the mixture was partitioned between methylene chloride (50 mL) and an aqueous saturated solution of potassium tartrate (50 mL). The organic phase was washed with water (2 × 30 mL), dried (MgSO_4_), and the solvent was evaporated. The product was purified by column chromatography (silica gel) eluting with hexane to give 411 mg (61% yield) of **50** as a colorless oil: ^1^H NMR (500.13 MHz, CDCl_3_) δ 1.40 (t, *J* = 7.1 Hz, 1H, S*H*), 1.60 (s, 3H, H-8), 1.66 (d, *J* = 1.2 Hz, C*H*_3_ at C-7), 1.68 (d, *J* = 1.2 Hz, C*H*_3_ at C-3), 2.00 (m, 2H, H-4), 2.07 (m, 2H, H-4), 3.16 (ddd, *J* = 7.6, 7.3, 0.4 Hz, 2H, H-1), 5.08 (tq, *J* = 6.9, 1.4 Hz, 1H, H-6), 5.34 (tsxt, *J* = 7.8, 1.3 Hz, 1H, H-2); ^13^C NMR (125.77 MHz, CDCl_3_) δ 15.8 (*C*H_3_ at C-2), 17.7 (*C*H_3_ at C-6), 22.1 (C-1), 25.7 (C-8), 26.4 (C-5), 39.4 (C-4), 123.3 (C-2), 123.6 (C-5), 131.7 (C-7), 137.5 (C-3). HRMS (ESI) calcd. for C_10_H_19_S [M + H]^+^ 171.1207; found 171.1194.

*Tetraethyl 1-[((E)-3,7-dimethylocta-2,6-diene-1-thio)ethyl] 1,1-bisphosphonate* (**52**). A solution of **21** (1.00 mg, 3.34 mmol) in anhydrous methylene chloride (15 mL) was treated with triethylamine (337 mg, 470 μL, 3.34 mmol) and **50** (580.9 mg, 3.34 mmol) as described for the preparation of **22**. The solvent was evaporated and the residue was purified by column chromatography (silica gel) employing EtOAc as eluent to give 1.163 g (74% yield) of **52** as a colorless oil: ^1^H NMR (500.13 MHz, CDCl_3_) δ 1.35 (t, *J* = 7.1 Hz, 12H, CH_2_C*H*_3_), 1.60 (s, 3H, H-11), 1.67 (s, 3H, C*H*_3_ at C-10), 1.68 (s, 3H, C*H*_3_ at C-6), 2.02 (m, 2H, H-7), 2.07 (m, 2H, H-8), 2.61 (tt, *J* = 23.8, 6.1 Hz, 1H, H-1), 3.02 (dt, *J* = 16.2, 6.1 Hz, 2H, H-2), 3.22 (d, *J* = 7.7 Hz, 2H, H-4), 5.08 (m, 1H, H-9), 5.26 (t, *J* = 7.7 Hz, 1H, H-5); ^13^C NMR (125.77 MHz, CDCl_3_) δ 16.2 (*C*H_3_ at C-6), 16.4 (d, *J* = 6.1 Hz, CH_2_*C*H_3_), 17.7 (*C*H_3_ at C-10), 25.7 (C-11), 26.5 (C-8), 27.3 (t, *J* = 5.0 Hz, C-2), 30.7 (C-4), 38.9 (t, *J* = 131.6 Hz, C-1), 39.6 (C-4), 119.9 (C-5), 123.9 (C-9), 131.7 (C-10), 139.4 (C-6); ^31^P NMR (202.46 MHz, CDCl_3_) δ 21.72 ppm. HRMS (ESI) calcd. for C_20_H_40_O_6_SP_2_ [M + Na]^+^ 493.1919; found 493.1929.

*1-[((E)-3,7-Dimethylocta-2,6-diene-1-thio)ethyl] 1,1-bisphosphonic Acid* (**44**). A solution of the Michael adduct **52** (997 mg, 2.1 mmol) and 2,4,6-collidine (2.53 g, 2.76 mL, 21.0 mmol) in anhydrous methylene chloride (20 mL) was treated with bromotrimethylsilane (3.23 g, 2.73 mL, 21.1 mmol) under an argon atmosphere. The reaction mixture was stirred at room temperature for 48 h. Then, methanol (1.0 mL) was added and the solvent was evaporated. The residue was dissolved in methanol (10 mL) and the mixture was stirred at room temperature for 24 h. The solvent was evaporated and the residue redissolved/evaporated in methanol four times. The product was purified by column chromatography on reverse phase eluting with a mixture of water–acetonitrile (3:2) to give 361.2 mg (48% yield) of **44** as an amorphous solid: ^1^H NMR (500.13 MHz, D_2_O) δ 1.54 (s, 3H, H-11), 1.61 (s, 3H, C*H*_3_ at C-10), 1.62 (s, 3H, C*H*_3_ at C-6), 1.94–2.08 (m, 5H, H-1, H-7, H-8), 2.93 (ddd, *J* = 15.1, 14.0, 7.3 Hz, 2H, H-2), 3.20 (d, *J* = 7.7 Hz, 2H, H-4), 5.11 (t, *J* = 7.0 Hz, 1H, H-9), 5.30 (t, *J* = 7.5 Hz, 1H, H-5); ^13^C NMR (125.77 MHz, D_2_O) δ 15.3 (*C*H_3_ at C-6), 16.9 (*C*H_3_ at C-10), 24.8 (C-11), 25.7 (C-8), 28.7 (t, *J* = 3.7 Hz, C-2), 29.9 (C-4), 40.1 (t, *J* = 109.3 Hz, C-1), 38.7 (C-4), 119.8 (C-5), 124.2 (C-9), 133.6 (C-10), 140.2 (C-6); ^31^P NMR (202.46 MHz, D_2_O) δ 15.85 ppm. HRMS (ESI) calcd. for C_12_H_22_O_6_SP_2_Na_3_ [M + H]^+^ 425.0305; found 425.0298.

*S-((2E,6E)-3,7,11-Trimethyldodeca-2,6,10-trien-1-yl) ethanethioate* (**49**). To a mixture of farnesol **47** (1.00 g, 4.5 mmol), triphenylphosphine (1.77 g, 6.7 mmol), thioacetic acid (0.38 mL, 410.1 mg, 5.4 mmol) in anhydrous tetrahydrofuran (20 mL) cooled at 0 °C was added a solution of diisopropyl azodicarboxylate (1.13 mL, 1.15 g, 6.7 mmol) as depicted for the preparation of **31**. The product was purified by column chromatography (silica gel) eluting with hexane–EtOAc (9:1) to give 840.0 mg (84% yield) of **49** as a colorless oil: *R*_f_ 0.38 (hexane–EtOAc, 4:1); ^1^H NMR (500.13 MHz, CDCl_3_) δ 1.59 (s, 3H, C-12), 1.60 (s, 3H, C*H*_3_ at C-7), 1.68 (s, 6H, C*H*_3_ at C-3C*H*_3_ at C-11), 1.95–2.10 (m, 8H, H-4, H-5, H-8, H-9), 2.32 (s, 3H, SC(O)C*H*_3_), 3.54 (t, *J* = 7.9 Hz, 2H, H-1), 5.08 (m, 2H, H-6, H-10), 5.21 (tq, *J* = 7.9, 1.1 Hz, 1H, H-2); ^13^C NMR (125.77 MHz, CDCl3) δ 16.0 (*C*H_3_ at C-3), 16.2 (*C*H_3_ at C-7), 17.7 (*C*H_3_ at C-11), 25.9 (C-12), 26.3 (C-9), 26.7 (C-5), 27.3 (C-1), 30.4 (SC(O)*C*H_3_), 39.5 (C-4), 39.7 (C-8), 118.4 (C-2), 123.7 (C-10), 124.3 (C-6), 131.3 (C-11), 135.3 (C-7), 140.2 (C-3), 196.1 (S*C*(O)CH_3_). HRMS (ESI) calcd. for C_17_H_28_OS [M + Na]^+^ 303.1759; found 303.1757.

*(2E,6E)-3,7,11-Trimethyldodeca-2,6,10-triene-1-thiol* (**51**). A solution of **49** (840.3 mg, 3.0 mmol) in anhydrous tetrahydrofuran (10 mL) cooled at 0 °C was treated with portionwise with lithium aluminum hydride (170 mg, 4.5 mmol) as described for the preparation of **50** to obtain 421.8 mg of **51** (59% yield) as a colorless oil: ^1^H NMR (500.13 MHz, CDCl_3_) δ 1.42 (t, *J* = 7.1 Hz, 1H, S*H*), 1.62 (s, 6H, C-12, C*H*_3_ at C-3), 1.68 (s, 3H, C*H*_3_ at C-7), 1.71 (s, 3H, C*H*_3_ at C-11), 1.99–2.14 (m, 8H, H-4, H-5, H-8, H-9), 3.18 (t, *J* = 7.2 Hz, 2H, H-1), 5.12 (m, 2H, H-6, H-10), 5.37 (tq, *J* = 7.8, 1.2 Hz, 1H, H-2); ^13^C NMR (125.77 MHz, CDCl3) δ 15.8 (*C*H_3_ at C-3), 16.0 (*C*H_3_ at C-7), 17.7 (*C*H_3_ at C-11), 22.1 (C-1), 25.7 (C-12), 26.3 (C-9), 26.7 (C-5), 39.4 (C-4), 39.7 (C-8), 123.3 (C-2), 123.7 (C-10), 124.3 (C-6), 131.3 (C-11), 135.3 (C-7), 137.5 (C-3). HRMS (ESI) calcd. for C_15_H_27_S [M + H]^+^ 239.1833; found 239.1820.

*Tetraethyl 1-[((2E,6E)-3,7,11-trimethyldodeca-2,6,10-triene-1-thio)ethyl]-1,1-bisphosphonate* (**53**). A solution of **21** (303 mg, 1.0 mmol) in anhydrous methylene chloride (10 mL) was treated with triethylamine (112 mg, 154 μL, 1.1 mmol) and **51** (241 mg, 1.0 mmol) as described for the preparation of **22**. The solvent was evaporated and the residue was purified by column chromatography (silica gel) employing EtOAc as eluent to yield 412.3 mg (76% yield) of **53** as a colorless oil: *R*_f_ 0.50 (EtOAc–methanol, 9:1); ^1^H NMR (500.13 MHz, CDCl_3_) δ 1.35 (t, *J* = 7.1 Hz, 12H, CH_2_C*H*_3_), 1.36 (t, *J* = 7.0 Hz, 12H, CH_2_C*H′*_3_), 1.60 (s, 6H, C-15, C*H*_3_ at C-6), 1.68 (s, 6H, C*H*_3_ at C-10, C*H*_3_ at C-14), 1.96–2.10 (m, 8H, H-7, H-8, H-11, H-12), 2.61 (tt, *J* = 23.8, 6.1 Hz, 1H, H-1), 3.02 (dt, *J* = 18.2, 6.1 Hz, 2H, H-2), 3.22 (d, *J* = 7.7 Hz, 2H, H-4), 4.20 (m, 8H, C*H*_2_CH_3_), 5.10 (m, 2H, H-9, H-13), 5.26 (tq, *J* = 7.6, 1.0 Hz, 1H, H-5); ^13^C NMR (125.77 MHz, CDCl3) δ 16.2 (CH_3_ at C-6), 16.3 (CH_3_ at C-10), 16.4 (d, *J* = 6.1 Hz, CH_2_*C*H_3_), 17.7 (CH_3_ at C-14), 25.7 (C-15), 27.3 (t, *J* = 5.0 Hz, C-2), 26.5 (C-12), 26.7 (C-8), 30.7 (C-4), 38.9 (t, *J* = 131.6 Hz, C-1), 39.6 (C-7), 39.7 (C-11), 62.7 (d, *J* = 6.7 Hz, *C*H_2_CH_3_), 62.8 (d, *J* = 6.6 Hz, *C*′H_2_CH_3_), 119.8 (C-5), 123.8 (C-13), 124.3 (C-9), 131.3 (C-14), 135.3 (C-10), 139.5 (C-6); ^31^P NMR (202.46 MHz, CDCl_3_) δ 21.47. HRMS (ESI) calcd. for C_25_H_48_O_6_SP_2_ [M + Na]^+^ 561.2545; found 561.2534.

*1-[((2E,6E)-3,7,11-Trimethyldodeca-2,6,10-triene-1-thio)ethyl]-1,1-bisphosphonic Acid* (**45**). A solution of **53** (375 mg, 0.7 mmol), 2,4,6-collidine (843 mg, 920 µL, 7.0 mmol) in anhydrous methylene chloride (10 mL) was treated with bromotrimethylsilane (1.07 g, 0.9 mL, 7.0 mmol) under an argon atmosphere. The reaction mixture was stirred at room temperature for 48 h. Then, methanol (1.0 mL) was added and the reaction mixture was treated as depicted for the preparation of **44**. The residue was purified by reversed-phase column chromatography eluting with a mixture of water–acetonitrile (2:1) to give 170 mg (52% yield) of **45** as an amorphous solid: ^1^H NMR (500.13 MHz, D_2_O-CD_3_OD) δ 1.61 (s, 6H, C-15, C*H*_3_ at C-6), 1.69 (s, 3H, C*H*_3_ at C-10) 1.72 (s, 3H, C*H*_3_ at C-14), 1.96–2.17 (m, 9H, H-1, H-7, H-8, H-11, H-12), 3.06 (dt, *J* = 14.6, 7.1 Hz, 2H, H-2), 3.31 (d, *J* = 7.9 Hz, 2H, H-4), 5.14 (m, 2H, H-9, H-13), 5.26 (tq, *J* = 7.8, 1.0 Hz, 1H, H-5); ^13^C NMR (125.77 MHz, D_2_O-CD_3_OD) δ 16.1 (*C*H_3_ at C-6), 16.3 (*C*H_3_ at C-10), 17.9 (*C*H_3_ at C-14), 24.3 (C-15), 25.9 (C-12), 30.2 (t, *J* = 3.7 Hz, C-2), 26.9 (C-12), 27.0 (C-8), 31.4 (C-4), 41.4 (t, *J* = 109.4 Hz, C-1), 40.09 (C-7), 40.11 (C-11), 120.9 (C-5), 125.23 (C-13), 125.22 (C-9), 133.4 (C-14), 136.7 (C-10), 140.6 (C-6); ^31^P NMR (202.46 MHz, D_2_O-CD_3_OD) δ 18.16 ppm. HRMS (ESI) calcd. for C_17_H_29_O_6_SP_2_Na_4_ [M + H]^+^ 515.0751; found 515.0737.

*Acetylation Reaction. General Procedure*. Tetraethyl 2-(*N*-*n*-alkylamino)ethyl bisphosphonates **12**–**14**, **61**–**65** (15 mmol) were treated individually with acetic anhydride (3.0 mL) and pyridine (3.0 mL). The mixture was stirred at room temperature overnight. The solvent was evaporated and each residue was purified by column chromatography (silica gel) eluting with CH_2_Cl_2_–methanol (99:1) to produce the acetylated products **67**–**74**.

*Tetraethyl 2-(N-n-propylacetamido)ethyl-1,1-bisphosphonate* (**67**): 66% yield; colorless oil; ^1^H NMR (500.13 MHz, CDCl_3_) δ 0.90 (t, *J* = 7.4 Hz, 3H, H-6), 1.33 (t, *J* = 7.0 Hz, 6H, CH_2_C*H*_3_), 1.34 (t, *J* = 7.0 Hz, 6H, CH_2_C*H*′_3_), 1.55 (p, *J* = 7.1 Hz, 2H, H-5), 2.06 (s, 3H, C(O)C*H*_3_), 3.38 (t, *J* = 7.7 Hz, 1H, H-4), 3.46 (tt, *J* = 22.6, 7.5 Hz, 1H, H-1), 3.74 (ddd, *J* = 14.7, 11.2, 7.4 Hz, 2H, H-2), 4.17 (m, 8H, C*H*_2_CH_3_); ^13^C NMR (125.77 MHz, CDCl_3_) δ 11.2 (C-6), 16.30 (d, *J* = 4.1 Hz, CH_2_*C*H_3_), 16.36 (d, *J* = 4.1 Hz, CH_2_*C*H_3_), 21.6 ((C(O)*C*H_3_), 22.2 (C-5), 34.1 (t, *J* = 130.1 Hz, C-1), 44.6 (t, *J* = 3.3 Hz, C-2), 52.7 (C-4), 62.4 (d, *J* = 6.8 Hz, *C*H_2_CH_3_), 63.0 (d, *J* = 6.7 Hz, *C*H_2_CH_3_), 171.1 (*C*(O)CH_3_); ^31^P NMR (202.46 MHz, CDCl_3_) δ 21.48.

*Tetraethyl 2-(N-n-butylacetamido)ethyl-1,1-bisphosphonate* (**68**): 51% yield; colorless oil; ^1^H NMR (500.13 MHz, CDCl_3_) δ 0.95 (t, *J* = 7.3 Hz, 3H, H-7), 1.34 (t, *J* = 7.0 Hz, 6H, CH_2_C*H*_3_), 1.35 (t, *J* = 7.1 Hz, 6H, CH_2_C*H*′_3_), 1.53 (p, *J* = 7.1 Hz, 2H, H-5), 2.07 (s, 3H, C(O)C*H*_3_), 3.41 (t, *J* = 7.8 Hz, 1H, H-4), 3.44 (tt, *J* = 22.8, 7.4 Hz, 1H, H-1), 3.74 (ddd, *J* = 14.7, 11.1, 7.4 Hz, 2H, H-2), 4.19 (m, 8H, C*H*_2_CH_3_); ^13^C NMR (125.77 MHz, CDCl_3_) δ 13.8 (C-7), 16.27 (d, *J* = 4.1 Hz, CH_2_*C*H_3_), 16.33 (d, *J* = 4.1 Hz, CH_2_*C*H_3_), 20.0 (C-6), 21.6 ((C(O)*C*H_3_), 31.1 (C-5), 34.1 (t, *J* = 130.1 Hz, C-1), 44.6 (t, *J* = 3.3 Hz, C-2), 50.6 (C-4), 62.4 (d, *J* = 6.8 Hz, *C*H_2_CH_3_), 62.9 (d, *J* = 6.8 Hz, *C*H_2_CH_3_), 171.0 (*C*(O)CH_3_); ^31^P NMR (202.46 MHz, CDCl_3_) δ 21.47.

*Tetraethyl 2-(N-n-penylacetamido)ethyl-1,1-bisphosphonate* (**69**): 47% yield, colorless oil; ^1^H NMR (500.13 MHz, CDCl_3_) δ 0.91 (t, *J* = 7.2 Hz, 3H, H-8), 1.26 (m, 2H, C*H*_2_), 1.34 (t, *J* = 7.1 Hz, 6H, CH_2_C*H*_3_), 1.35 (t, *J* = 7.1 Hz, 6H, CH_2_C*H*′_3_), 1.53 (p, *J* = 7.7 Hz, 2H, H-5), 2.07 (s, 3H, C(O)C*H*_3_), 3.41 (t, *J* = 7.7 Hz, 1H, H-4), 3.44 (tt, *J* = 22.8, 7.4 Hz, 1H, H-1), 3.74 (ddd, *J* = 14.7, 11.1, 7.4 Hz, 2H, H-2), 4.19 (m, 8H, C*H*_2_CH_3_); ^13^C NMR (125.77 MHz, CDCl_3_) δ 13.9 (C-8), 16.3 (d, *J* = 4.2 Hz, CH_2_*C*H_3_), 16.4 (d, *J* = 4.2 Hz, CH_2_*C*H_3_), 21.6 ((C(O)*C*H_3_), 22.4 (C-7), 28.7 (C-6), 28.9 (C-5), 34.1 (t, *J* = 130.0 Hz, C-1), 44.6 (t, *J* = 3.4 Hz, C-2), 50.9 (C-4), 62.4 (d, *J* = 6.8 Hz, *C*H_2_CH_3_), 62.9 (d, *J* = 6.7 Hz, *C*H_2_CH_3_), 171.0 (*C*(O)CH_3_); ^31^P NMR (202.46 MHz, CDCl_3_) δ 21.47.

*Tetraethyl 2-(N-n-hexylacetamido)ethyl-1,1-bisphosphonate* (**70**): 51% yield, colorless oil; ^1^H NMR (500.13 MHz, CDCl_3_) δ 0.87 (t, *J* = 6.9 Hz, 3H, H-9), 1.29 (m, 6H, C*H*_2_), 1.34 (t, *J* = 7.0 Hz, 6H, CH_2_C*H*_3_), 1.35 (t, *J* = 7.1 Hz, 6H, CH_2_C*H*′_3_), 1.52 (m, 2H, H-5), 2.07 (s, 3H, C(O)C*H*_3_), 3.41 (t, *J* = 7.7 Hz, 1H, H-4), 3.44 (tt, *J* = 22.8, 7.4 Hz, 1H, H-1), 3.74 (ddd, *J* = 14.7, 11.1, 7.4 Hz, 2H, H-2), 4.18 (m, 8H, C*H*_2_CH_3_); ^13^C NMR (125.77 MHz, CDCl_3_) δ 14.0 (C-9), 16.2 (d, *J* = 4.3 Hz, CH_2_*C*H_3_), 16.4 (d, *J* = 4.1 Hz, CH_2_*C*H_3_), 21.6 ((C(O)*C*H_3_), 22.6 (C-8), 26.5 (C-6), 29.0 (C-5), 31.5 (C-7), 34.1 (t, *J* = 130.0 Hz, C-1), 44.6 (t, *J* = 3.2 Hz, C-2), 50.9 (C-4), 62.4 (d, *J* = 6.8 Hz, *C*H_2_CH_3_), 62.9 (d, *J* = 6.7 Hz, *C*H_2_CH_3_), 171.0 (*C*(O)CH_3_); ^31^P NMR (202.46 MHz, CDCl_3_) δ 21.48.

*Tetraethyl 2-(N-n-heptylacetamido)ethyl-1,1-bisphosphonate* (**71**): 66% yield; colorless oil; ^1^H NMR (500.13 MHz, CDCl_3_) δ 0.89 (t, *J* = 7.1 Hz, 3H, H-10), 1.29 (m, 8H, C*H*_2_), 1.34 (t, *J* = 7.1 Hz, 6H, CH_2_C*H*_3_), 1.35 (t, *J* = 7.1 Hz, 6H, CH_2_C*H*′_3_), 1.52 (p, *J* = 7.5 Hz, 2H, H-5), 2.07 (s, 3H, C(O)C*H*_3_), 3.41 (t, *J* = 7.7 Hz, 1H, H-4), 3.44 (tt, *J* = 23.1, 7.4 Hz, 1H, H-1), 3.74 (ddd, *J* = 14.7, 11.1, 7.4 Hz, 2H, H-2), 4.18 (m, 8H, C*H*_2_CH_3_); ^13^C NMR (125.77 MHz, CDCl_3_) δ 14.0 (C-10), 16.3 (d, *J* = 4.4 Hz, CH_2_*C*H_3_), 16.4 (d, *J* = 4.1 Hz, CH_2_*C*H_3_), 21.6 ((C(O)*C*H_3_), 22.6 (C-9), 26.8 (C-6), 29.03 (C-5), 29.04 (C-7), 31.5 (C-8), 34.1 (t, *J* = 130.0 Hz, C-1), 44.6 (C-2), 50.9 (C-4), 62.4 (d, *J* = 6.6 Hz, *C*H_2_CH_3_), 62.9 (d, *J* = 6.8 Hz, *C*H_2_CH_3_), 171.0 (*C*(O)CH_3_); ^31^P NMR (202.46 MHz, CDCl_3_) δ 21.49.

*Tetraethyl 2-(N-n-octylacetamido)ethyl-1,1-bisphosphonate* (**72**): 50% yield; colorless oil; ^1^H NMR (500.13 MHz, CDCl_3_) δ 0.89 (t, *J* = 7.0 Hz, 3H, H-11), 1.29 (m, 10H, C*H*_2_), 1.34 (t, *J* = 7.1 Hz, 6H, CH_2_C*H*_3_), 1.35 (t, *J* = 7.1 Hz, 6H, CH_2_C*H*′_3_), 1.52 (p, *J* = 7.4 Hz, 2H, H-5), 2.07 (s, 3H, C(O)C*H*_3_), 3.41 (t, *J* = 7.8 Hz, 1H, H-4), 3.44 (tt, *J* = 22.9, 7.4 Hz, 1H, H-1), 3.74 (ddd, *J* = 14.7, 11.1, 7.4 Hz, 2H, H-2), 4.19 (m, 8H, C*H*_2_CH_3_); ^13^C NMR (125.77 MHz, CDCl_3_) δ 14.0 (C-11), 16.2 (d, *J* = 3.9 Hz, CH_2_*C*H_3_), 16.3 (d, *J* = 3.9 Hz, CH_2_*C*H_3_), 21.5 ((C(O)*C*H_3_), 22.5 (C-10), 26.7 (C-6), 28.9 (C-8), 29.1 (C-5), 29.2 (C-7), 31.6 (C-9), 34.0 (t, *J* = 130.0 Hz, C-1), 44.5 (t, *J* = 3.2 Hz, C-2), 50.8 (C-4), 62.3 (d, *J* = 6.8 Hz, *C*H_2_CH_3_), 62.8 (d, *J* = 6.6 Hz, *C*H_2_CH_3_), 170.9 (*C*(O)CH_3_); ^31^P NMR (202.46 MHz, CDCl_3_) δ 21.44.

*Tetraethyl 2-(N-n-nonylacetamido)ethyl-1,1-bisphosphonate* (**73**): 57% yield; colorless oil; ^1^H NMR (500.13 MHz, CDCl_3_) δ 0.84 (t, *J* = 6.9 Hz, 3H, H-12), 1.23 (m, 12H, C*H*_2_), 1.30 (t, *J* = 7.1 Hz, 6H, CH_2_C*H*_3_), 1.31 (t, *J* = 7.1 Hz, 6H, CH_2_C*H*′_3_), 1.49 (p, *J* = 7.1 Hz, 2H, H-5), 2.03 (s, 3H, C(O)C*H*_3_), 3.37 (t, *J* = 7.8 Hz, 1H, H-4), 3.40 (tt, *J* = 23.0, 7.4 Hz, 1H, H-1), 3.70 (ddd, *J* = 14.8, 11.2, 7.4 Hz, 2H, H-2), 4.15 (m, 8H, C*H*_2_CH_3_); ^13^C NMR (125.77 MHz, CDCl_3_) δ 14.0 (C-12), 16.2 (d, *J* = 4.0 Hz, CH_2_*C*H_3_), 16.3 (d, *J* = 3.9 Hz, CH_2_*C*H_3_), 21.5 ((C(O)*C*H_3_), 22.5 (C-11), 26.7 (C-6), 29.0 (C-8), 29.1 (C-5), 29.3 (C-7), 29.4 (C-9), 31.8 (C-10), 34.0 (t, *J* = 130.1 Hz, C-1), 44.5 (t, *J* = 3.3 Hz, C-2), 50.9 (C-4), 62.3 (d, *J* = 6.9 Hz, *C*H_2_CH_3_), 62.9 (d, *J* = 6.7 Hz, *C*H_2_CH_3_), 170.9 (*C*(O)CH_3_); ^31^P NMR (202.46 MHz, CDCl_3_) δ 21.45.

*Tetraethyl 2-(N-n-decylacetamido)ethyl-1,1-bisphosphonate* (**74**): 73% yield; colorless oil; ^1^H NMR (500.13 MHz, CDCl_3_) δ 0.85 (t, *J* = 6.9 Hz, 3H, H-13), 1.23 (m, 14H, C*H*_2_), 1.31 (t, *J* = 7.0 Hz, 6H, CH_2_C*H*_3_), 1.32 (t, *J* = 7.1 Hz, 6H, CH_2_C*H*′_3_), 1.49 (p, *J* = 7.1 Hz, 2H, H-5), 2.04 (s, 3H, C(O)C*H*_3_), 3.38 (t, *J* = 7.5 Hz, 1H, H-4), 3.44 (tt, *J* = 23.2, 7.6 Hz, 1H, H-1), 3.71 (ddd, *J* = 14.6, 11.2, 7.5 Hz, 2H, H-2), 4.15 (m, 8H, C*H*_2_CH_3_); ^13^C NMR (125.77 MHz, CDCl_3_) δ 14.0 (C-13), 16.27 (d, *J* = 6.8 Hz, CH_2_*C*H_3_), 16.30 (d, *J* = 6.6 Hz, CH_2_*C*H_3_), 21.6 ((C(O)*C*H_3_), 22.6 (C-12), 26.8 (C-6), 29.0 (C-8), 29.2 (C-5), 29.3 (C-7), 29.4 (C-9), 29.5 (C-10), 31.8 (C-11), 34.0 (t, *J* = 130.0 Hz, C-1), 44.5 (t, *J* = 3.3 Hz, C-2), 50.9 (C-4), 62.4 (d, *J* = 6.9 Hz, *C*H_2_CH_3_), 62.9 (d, *J* = 6.7 Hz, *C*H_2_CH_3_), 171.0 (*C*(O)CH_3_); ^31^P NMR (202.46 MHz, CDCl_3_) δ 21.46.

*Fluorination Reaction. General Procedure.* Sodium hydride (10.5 mmol, 60% in mineral oil) was washed with anhydrous hexane under an argon atmosphere, and then anhydrous tetrahydrofuran (10 mL) was added. The mixture, cooled at 0 °C, was treated with the corresponding tetraethyl 2-(*N*-*n*-alkylacetamido)ethyl-1,1-bisphosphonate such as **67**–**74** (10.0 mmol) in anhydrous tetrahydrofuran (10 mL). The solution was stirred at 0 °C for 15 min, then, the mixture was allowed to reach room temperature and stirred for additional 60 min. The mixture was cooled at 0 °C and Selectfluor (12.5 mmol) was added followed by addition of *N*,*N*-dimethylformamide (5.0 mL). The reaction mixture was allowed to reach room temperature and stirred for 4 h. The mixture was partitioned between methylene chloride (30 mL) and brine (30 mL). The aqueous phase was extracted with methylene chloride (2 × 30 mL). The combined organic layers were washed with brine (5 × 30 mL), water (2 × 25 mL), dried (MgSO_4_), and the solvent was evaporated. The residue was purified by column chromatography eluting with a mixture of EtOAc–methanol (49:1) to produce the corresponding 1-fluoro tetraethyl esters **75**–**82**.

*Tetraethyl 2-(N-n-propylacetamido)ethyl-1-fluoro-1,1-bisphosphonate* (**75**): 50% yield; ^1^H NMR (500.13 MHz, CDCl_3_) δ 0.88 (t, *J* = 7.3 Hz, 3H, H-6), 0.91 (t, *J* = 7.4 Hz, 3H, H-6′), 1.38 (t, *J* = 7.0 Hz, 12H, CH_2_C*H*_3_), 1.39 (dt, *J* = 6.9, 3.3 Hz, 12H, CH_2_C*H*′_3_), 1.53 (sext, *J* = 7.6 Hz, 2H, H-5), 1.60 (sext, *J* = 7.5 Hz, 2H, H-5′), 2.14 (s, 3H, C(O)C*H*_3_), 2.21 (s, 3H, C(O)C*H*_3_), 3.45 (t, *J* = 7.8 Hz, 2H, H-4), 3.47 (t, *J* = 7.9 Hz, 2H, H-4′), 4.10 (dd, *J* = 10.1, 8.8 Hz, 2H, H-2), 4.15 (dd, *J* = 10.2, 8.8 Hz, 2H, H-2′), 4.27 (m, 8H, C*H*_2_CH_3_), two rotamers; ^13^C NMR (125.77 MHz, CDCl_3_) δ 11.1 (C-6), 11.3 (C-6′), 16.3 (t, *J* = 3.3 Hz, CH_2_*C*H_3_), 16.4 (t, *J* = 2.3 Hz, CH_2_*C*H′_3_), 19.8 (C-5), 21.2 (C-5′), 21.4 (C(O)*C*H_3_), 21.9 (d, *J* = 3.5 Hz, C(O)*C*H′_3_), 44.9 (d, *J* = 17.9 Hz, C-2), 48.4 (d, *J* = 3.5 Hz, C-4), 48.9 (d, *J* = 18.3 Hz, C-2′), 50.9 (d, *J* = 4.2 Hz, C-2′), 64.2 (m, *C*H_2_CH_3_), 64.7 (t, *J* = 3.4 Hz, *C*H_2_CH_3_), 171.3 (*C*(O)CH_3_), 171.6 (*C*(O)′CH_3_), two rotamers; ^31^P NMR (202.46 MHz, CDCl_3_) δ 11.70 (d, *J* = 70.2 Hz), 11.90 (d, *J* = 77.4 Hz), two rotamers; ^19^F NMR (470.59 MHz, CDCl_3_) δ −192.55 (t, *J* = 77.4 Hz), −192.80 (t, *J* = 71.3 Hz), two rotamers.

*Tetraethyl 2-(N-n-butylacetamido)ethyl-1-fluoro-1,1-bisphosphonate* (**76**): 65% yield; colorless oil; ^1^H NMR (500.13 MHz, CDCl_3_) δ 0.91 (t, *J* = 7.3 Hz, 3H, H-7), 0.94 (t, *J* = 7.4 Hz, 3H, H-7′), 1.30 (m, 2H, H-6), 1.37 (t, *J* = 6.8 Hz, 12H, CH_2_C*H*_3_), 1.38 (dt, *J* = 6.8, 3.3 Hz, 12H, CH_2_C*H*′_3_), 1.49 (p, *J* = 7.7 Hz, 2H, H-5), 1.54 (p, *J* = 7.8 Hz, 2H, H-5′), 2.12 (s, 3H, C(O)C*H*_3_), 2.20 (s, 3H, C(O)C*H*_3_), 3.46 (t, *J* = 7.9 Hz, 2H, H-4), 3.49 (t, *J* = 7.9 Hz, 2H, H-4′), 4.10 (dd, *J* = 10.5, 8.5 Hz, 2H, H-2), 4.14 (dd, *J* = 10.3, 8.9 Hz, 2H, H-2′), 4.27 (m, 8H, C*H*_2_CH_3_), two rotamers; ^13^C NMR (125.77 MHz, CDCl_3_) δ 13.75 (C-7), 13.84 (C-7′), 16.39 (t, *J* = 3.2 Hz, CH_2_*C*H_3_), 16.44 (t, *J* = 2.6 Hz, CH_2_*C*H′_3_), 20.0 (C-6), 20.1 (C-6′), 21.5 (C(O)*C*H_3_), 22.0 (d, *J* = 3.6 Hz, C(O)*C*H′_3_), 28.7 (C-5), 30.1 (C-5′), 44.9 (d, *J* = 18.1 Hz, C-2), 46.5 (d, *J* = 3.3 Hz, C-4), 49.1 (d, *J* = 4.2 Hz, C-4′), 49.9 (d, *J* = 18.1 Hz, C-2′), 64.2 (m, *C*H_2_CH_3_), 64.7 (m, *C*H_2_CH_3_), 171.2 (*C*(O)CH_3_), 171.6 (*C*(O)′CH_3_), two rotamers; ^31^P NMR (202.46 MHz, CDCl_3_) δ 11.70 (d, *J* = 70.2 Hz), 11.90 (d, *J* = 77.2 Hz), two rotamers; ^19^F NMR (470.59 MHz, CDCl_3_) δ −192.46 (t, *J* = 77.5 Hz), −192.81 (t, *J* = 71.4 Hz), two rotamers. HRMS (ESI) calcd. for (C_16_H_34_O_7_NFP_2_Na) [M + Na]^+^ 456.1692; found 456.1700.

*Tetraethyl 2-(N-n-pentylacetamido)ethyl-1-fluoro-1,1-bisphosphonate* (**77**): 75% yield; colorless oil; ^1^H NMR (500.13 MHz, CDCl_3_) δ 0.89 (t, *J* = 7.5 Hz, 3H, H-8), 0.90 (t, *J* = 7.5 Hz, 3H, H-8′), 1.24 (m, 2H, C*H*_2_), 1.37 (t, *J* = 6.7 Hz, 12H, CH_2_C*H*_3_), 1.38 (dt, *J* = 6.5, 2.6 Hz, 12H, CH_2_C*H*′_3_), 1.51 (p, *J* = 7.6 Hz, 2H, H-5), 1.55 (p, *J* = 7.7 Hz, 2H, H-5′), 2.12 (s, 3H, C(O)C*H*_3_), 2.20 (s, 3H, C(O)C*H*_3_), 3.47 (t, *J* = 7.9 Hz, 2H, H-4), 4.10 (t, *J* = 9.6 Hz, 2H, H-2), 4.15 (t, *J* = 9.1 Hz, 2H, H-2′), 4.27 (m, 8H, C*H*_2_CH_3_), two rotamers; ^13^C NMR (125.77 MHz, CDCl_3_) δ 13.86 (C-8), 13.90 (C-8′), 16.36 (m, CH_2_*C*H_3_), 21.4 (C(O)*C*H_3_), 21.88 (d. *J* = 3.5 Hz, C(O)*C*H′_3_), 22.3 (C-7), 22.4 (C-7′), 26.1 (C-6), 27.6 (C-6′), 28.8 (C-5), 29.0 (C-5′), 44.8 (d, *J* = 18.5 Hz, C-2), 46.7 (d, *J* = 3.3 Hz, C-4), 49.2 (d, *J* = 4.2 Hz, C-4′), 49.8 (d, *J* = 18.2 Hz, C-2′), 64.1 (m, *C*H_2_CH_3_), 64.6 (m, *C*H_2_CH_3_), 95.8 (dt, *J* = 153.2, 109.3 Hz, 1H, H-1), 97.3 (dt, *J* = 153.0, 103.1 Hz, 1H, H-1′), 171.1 (*C*(O)CH_3_), 171.4 (*C*(O)′CH_3_), two rotamers; ^31^P NMR (202.46 MHz, CDCl_3_) δ 11.66 (d, *J* = 71.8 Hz), 11.87 (d, *J* = 78.0 Hz), two rotamers; ^19^F NMR (470.59 MHz, CDCl_3_) δ −192.56 (t, *J* = 77.4 Hz), −192.82 (t, *J* = 71.2 Hz); two rotamers.

*Tetraethyl 2-(N-n-hexylacetamido)ethyl-1-fluoro-1,1-bisphosphonate* (**78**): 36% yield; colorless oil; ^1^H NMR (500.13 MHz, CDCl_3_) δ 0.87 (t, *J* = 6.9 Hz, 3H, H-9), 0.89 (t, *J* = 6.9 Hz, 3H, H-9′), 1.29 (m, 6H, C*H*_2_), 1.37 (t, *J* = 6.9 Hz, 12H, CH_2_C*H*_3_), 1.38 (dt, *J* = 6.9, 2.7 Hz, 12H, CH_2_C*H*′_3_), 1.49 (p, *J* = 7.5 Hz, 2H, H-5), 1.54 (p, *J* = 7.8 Hz, 2H, H-5′), 2.12 (s, 3H, C(O)C*H*_3_), 2.19 (s, 3H, C(O)C*H*_3_), 3.46 (t, *J* = 7.9 Hz, 2H, H-4), 4.09 (t, *J* = 9.4 Hz, 2H, H-2), 4.09 (t, *J* = 9.4 Hz, 2H, H-2′), 4.26 (m, 8H, C*H*_2_CH_3_), two rotamers; ^13^C NMR (125.77 MHz, CDCl_3_) δ 13.95 (C-9), 14.00 (C-9′), 16.39 (t, *J* = 3.2 Hz, CH_2_*C*H_3_), 16.44 (t, *J* = 2.6 Hz, CH_2_*C*H′_3_), 21.5 (C(O)*C*H_3_), 22.0 (d, *J* = 3.5 Hz, C(O)*C*H′_3_), 22.5 (C-8), 22.6 (C-8′), 26.56 (C-6), 26.58 (C-6′), 28.0 (C-5), 31.5 (C-7), 31.6 (C-7′), 44.9 (d, *J* = 18.2 Hz, C-2), 46.8 (d, *J* = 3.2 Hz, C-4), 49.3 (d, *J* = 4.3 Hz, C-4′), 49.9 (d, *J* = 18.4 Hz, C-2′), 62.4 (d, *J* = 6.8 Hz, *C*H_2_CH_3_), 62.9 (d, *J* = 6.7 Hz, *C*H_2_CH_3_), 171.1 (*C*(O)CH_3_), 171.5 (*C*(O)′CH_3_), two rotamers; ^31^P NMR (202.46 MHz, CDCl_3_) δ 11.71 (d, *J* = 69.7 Hz), 11.91 (d, *J* = 77.1 Hz), two rotamers; ^19^F NMR (470.59 MHz, CDCl_3_) δ −192.50 (t, *J* = 77.6 Hz), −192.78 (t, *J* = 71.2 Hz); two rotamers. HRMS (ESI) calcd. for (C_18_H_39_O_7_NFP_2_) [M + H]^+^ 462.2188; found 462.2197.

*Tetraethyl 2-(N-n-heptylacetamido)ethyl-1-fluoro-1,1-bisphosphonate* (**79**): 53% yield; colorless oil; ^1^H NMR (500.13 MHz, CDCl_3_) δ 0.87 (t, *J* = 6.4 Hz, 3H, H-10), 0.88 (t, *J* = 6.9 Hz, 3H, H-10′), 1.27 (m, 8H, C*H*_2_), 1.36 (t, *J* = 7.0 Hz, 12H, CH_2_C*H*_3_), 1.38 (dt, *J* = 6.8, 3.4 Hz, 12H, CH_2_C*H*′_3_), 1.49 (p, *J* = 7.6 Hz, 2H, H-5), 1.54 (p, *J* = 7.8 Hz, 2H, H-5′), 2.11 (s, 3H, C(O)C*H*_3_), 2.19 (s, 3H, C(O)C*H*_3_), 3.46 (t, *J* = 7.9 Hz, 2H, H-4), 4.09 (dd, *J* = 10.3, 8.6 Hz, 2H, H-2), 4.13 (t, *J* = 10.2, 8.7 Hz, 2H, H-2′), 4.27 (m, 8H, C*H*_2_CH_3_), two rotamers; ^13^C NMR (125.77 MHz, CDCl_3_) δ 13.99 (C-10), 14.02 (C-10′), 16.3 (t, *J* = 3.2 Hz, CH_2_*C*H_3_), 16.4 (t, *J* = 2.4 Hz, CH_2_*C*H′_3_), 21.5 (C(O)*C*H_3_), 21.9 (d, *J* = 3.5 Hz, C(O)*C*H′_3_), 22.50 (C-9), 22.53 (C-9′), 26.6 (C-6), 26.7 (C-6′), 26.9 (C-7), 28.0 (C-5), 31.69 (C-8), 31.74 (C-8′), 44.9 (d, *J* = 17.9 Hz, C-2), 46.8 (d, *J* = 3.2 Hz, C-4), 49.3 (d, *J* = 4.3 Hz, C-4′), 49.9 (d, *J* = 18.3 Hz, C-2′), 64.2 (m, *C*H_2_CH_3_), 95.9 (dt, *J* = 153.1, 102.8 Hz, 1H, H-1), 97.4 (dt, *J* = 153.1, 102.8 Hz, 1H, H-1′), 171.2 (*C*(O)CH_3_), 171.5 (*C*(O)′CH_3_), two rotamers; ^31^P NMR (202.46 MHz, CDCl_3_) δ 11.69 (d, *J* = 72.1 Hz), 11.89 (d, *J* = 78.4 Hz), two rotamers; ^19^F NMR (470.59 MHz, CDCl_3_) δ −192.52 (t, *J* = 77.5 Hz), −192.79 (t, *J* = 71.3 Hz); two rotamers.

*Tetraethyl 2-(N-n-octylacetamido)ethyl-1-fluoro-1,1-bisphosphonate* (**80**): 52% yield; colorless oil; ^1^H NMR (500.13 MHz, CDCl_3_) δ 0.87 (t, *J* = 7.1 Hz, 3H, H-11), 0.88 (t, *J* = 6.9 Hz, 3H, H-11′), 1.27 (m, 10H, C*H*_2_), 1.37 (t, *J* = 7.0 Hz, 12H, CH_2_C*H*_3_), 1.38 (dt, *J* = 6.8, 3.4 Hz, 12H, CH_2_C*H*′_3_), 1.50 (p, *J* = 7.6 Hz, 2H, H-5), 1.54 (p, *J* = 7.9 Hz, 2H, H-5′), 2.12 (s, 3H, C(O)C*H*_3_), 2.19 (s, 3H, C(O)C*H*_3_), 3.46 (t, *J* = 8.2 Hz, 2H, H-4), 4.09 (dd, *J* = 12.0, 7.2 Hz, 2H, H-2), 4.15 (t, *J* = 10.2, 8.8 Hz, 2H, H-2′), 4.27 (m, 8H, C*H*_2_CH_3_), two rotamers; ^13^C NMR (125.77 MHz, CDCl_3_) δ 14.01 (C-11), 14.02 (C-11′), 16.4 (m, CH_2_*C*H_3_), 21.5 (C(O)*C*H_3_), 21.9 (d, *J* = 3.5 Hz, C(O)*C*H′_3_), 22.55 (C-10), 22.58 (C-10′), 26.6 (C-6), 26.8 (C-6′), 26.9 (C-7), 28.0 (C-5), 29.1 (C-8), 29.3 (C-8′), 31.74 (C-9), 31.79 (C-9′), 44.9 (d, *J* = 18.0 Hz, C-2), 46.8 (d, *J* = 3.3 Hz, C-4), 49.3 (d, *J* = 4.2 Hz, C-4′), 49.9 (d, *J* = 18.4 Hz, C-2′), 64.2 (m, *C*H_2_CH_3_), 95.9 (dt, *J* = 153.1, 109.2 Hz, 1H, H-1), 97.4 (dt, *J* = 153.1, 103.3 Hz, 1H, H-1′), 171.2 (*C*(O)CH_3_), 171.5 (*C*(O)′CH_3_), two rotamers; ^31^P NMR (202.46 MHz, CDCl_3_) δ 11.69 (d, *J* = 72.1 Hz), 11.89 (d, *J* = 79.6 Hz), two rotamers; ^19^F NMR (470.59 MHz, CDCl_3_) δ −192.51 (t, *J* = 77.5 Hz), −192.78 (t, *J* = 71.3 Hz); two rotamers.

*Tetraethyl 2-(N-n-nonylacetamido)ethyl-1-fluoro-1,1-bisphosphonate* (**81**): 59% yield; colorless oil; ^1^H NMR (500.13 MHz, CDCl_3_) δ 0.875 (t, *J* = 7.0 Hz, 3H, H-11), 0.882 (t, *J* = 6.9 Hz, 3H, H-11′), 1.26 (m, 12H, C*H*_2_), 1.37 (t, *J* = 7.0 Hz, 12H, CH_2_C*H*_3_), 1.38 (dt, *J* = 6.9, 3.3 Hz, 12H, CH_2_C*H*′_3_), 1.50 (p, *J* = 7.6 Hz, 2H, H-5), 1.54 (p, *J* = 7.7 Hz, 2H, H-5′), 2.12 (s, 3H, C(O)C*H*_3_), 2.19 (s, 3H, C(O)C*H*_3_), 3.46 (t, *J* = 7.9 Hz, 2H, H-4), 3.48 (t, *J* = 8.5 Hz, 2H, H-4′), 4.09 (dd, *J* = 10.0, 8.9 Hz, 2H, H-2), 4.15 (t, *J* = 10.2, 8.8 Hz, 2H, H-2′), 4.27 (m, 8H, C*H*_2_CH_3_), two rotamers; ^13^C NMR (125.77 MHz, CDCl_3_) δ 14.0 (C-12), 14.1 (C-12′), 16.4 (m, CH_2_*C*H_3_), 21.5 (C(O)*C*H_3_), 21.9 (d, *J* = 3.6 Hz, C(O)*C*H′_3_), 22.59 (C-11), 22.61 (C-11′), 26.6 (C-6), 26.8 (C-6′), 26.9 (C-7), 28.0 (C-5), 29.16 (C-8), 29.21 (C-8′), 29.28 (C-7′) 29.39 (C-5′), 29.46 (C-9), 29.50 (C-9′), 31.78 (C-10), 31.81 (C-10′), 44.9 (d, *J* = 17.9 Hz, C-2), 46.8 (d, *J* = 3.4 Hz, C-4), 49.3 (d, *J* = 4.3 Hz, C-4′), 49.9 (d, *J* = 18.2 Hz, C-2′), 64.2 (m, *C*H_2_CH_3_), 64.7 (t, *J* = 3.2 Hz, *C*H_2_CH_3_), 95.9 (dt, *J* = 153.0, 109.0 Hz, 1H, H-1), 97.4 (dt, *J* = 153.0, 103.0 Hz, 1H, H-1′), 171.2 (*C*(O)CH_3_), 171.5 (*C*(O)′CH_3_), two rotamers; ^31^P NMR (202.46 MHz, CDCl_3_) δ 11.70 (d, *J* = 77.9 Hz), 11.91 (d, *J* = 77.9 Hz), two rotamers; ^19^F NMR (470.59 MHz, CDCl_3_) δ −192.50 (t, *J* = 77.4 Hz), −192.77 (t, *J* = 71.3 Hz); two rotamers.

*Tetraethyl 2-(N-n-decylacetamido)ethyl-1-fluoro-1,1-bisphosphonate* (**82**): 54% yield; colorless oil; ^1^H NMR (500.13 MHz, CDCl_3_) δ 0.877 (t, *J* = 7.0 Hz, 3H, H-13), 0.881 (t, *J* = 6.8 Hz, 3H, H-13′), 1.26 (m, 14H, C*H*_2_), 1.37 (t, *J* = 7.0 Hz, 12H, CH_2_C*H*_3_), 1.38 (dt, *J* = 6.9, 3.1 Hz, 12H, CH_2_C*H*′_3_), 1.50 (p, *J* = 7.3 Hz, 2H, H-5), 1.54 (p, *J* = 7.6 Hz, 2H, H-5′), 2.12 (s, 3H, C(O)C*H*_3_), 2.19 (s, 3H, C(O)C*H*_3_), 3.46 (t, *J* = 7.9 Hz, 2H, H-4), 3.48 (t, *J* = 8.2 Hz, 2H, H-4′), 4.09 (dd, *J* = 10.4, 8.6 Hz, 2H, H-2), 4.15 (t, *J* = 10.1, 8.8 Hz, 2H, H-2′), 4.27 (m, 8H, C*H*_2_CH_3_), two rotamers; ^13^C NMR (125.77 MHz, CDCl_3_) δ 14.0 (C-13), 16.4 (m, CH_2_*C*H_3_), 21.5 (C(O)*C*H_3_), 21.9 (d, *J* = 3.9 Hz, C(O)*C*H′_3_), 22.61 (C-12), 22.61 (C-12′), 26.6 (C-6), 26.8 (C-6′), 26.9 (C-7), 28.0 (C-5), 29.23 (C-8), 29.25 (C-8′), 29.27 (C-7′) 29.39 (C-5′), 29.45 (C-9), 29.50 (C-10), 29.54 (C-10′), 31.81 (C-11), 31.83 (C-11′), 44.9 (d, *J* = 18.6 Hz, C-2), 46.8 (d, *J* = 3.4 Hz, C-4), 49.3 (d, *J* = 4.5 Hz, C-4′), 49.9 (d, *J* = 18.4 Hz, C-2′), 64.2 (m, *C*H_2_CH_3_), 64.7 (t, *J* = 3.2 Hz, *C*H_2_CH_3_), 95.9 (dt, *J* = 153.0, 109.0 Hz, 1H, H-1), 97.4 (dt, *J* = 153.0, 103.1 Hz, 1H, H-1′), 171.2 (*C*(O)CH_3_), 171.5 (*C*(O)′CH_3_), two rotamers; ^31^P NMR (202.46 MHz, CDCl_3_) δ 11.70 (d, *J* = 71.2 Hz), 11.90 (d, *J* = 77.7 Hz), two rotamers; ^19^F NMR (470.59 MHz, CDCl_3_) δ −192.50 (t, *J* = 77.5 Hz), −192.78 (t, *J* = 71.2 Hz); two rotamers.

*Hydrolysys. General Procedure*. Esters **75**–**82** were treated, in independent experiments, with a concentrated aqueous solution of hydrochloric acid (2.0 mL). The resulting mixtures were refluxed for 24 h. The solvent was evaporated and the residues were crystallized from water–ethanol.

*1-[(n-Prop-1-ylamino)ethyl]-1-fluoro-1,1-bisphosphonic Acid* (**54**): 54% yield; white solid; m.p. 161 °C; ^1^H NMR (500.13 MHz, D_2_O) δ 0.91 (t, *J* = 7.5 Hz, 3H, H-6), 1.65 (sext, *J* = 7.4 Hz, 2H, H-5), 3.04 (t, *J* = 7.3 Hz, 2H, H-4), 3.60 (m, 2H, H-2); ^13^C NMR (125.77 MHz, D_2_O) δ 10.4 (C-6), 19.5 (C-5), 49.6 (d, *J* = 16.1, C-2), 50.2 (C-4); ^31^P NMR (202.46 MHz, D_2_O) δ 8.89 (d, *J* = 65.4 Hz); ^19^F NMR (470.59 MHz, D_2_O) δ −193.32 (t, *J* = 65.5 Hz). HRMS (ESI) calcd. for (C_5_H_15_O_6_NFP_2_) [M + H]^+^ 266.0359; found 266.0359.

*1-[(n-But-1-ylamino)ethyl]-1-fluoro-1,1-bisphosphonic Acid* (**55**): 70% yield; white solid; m.p. 153–154 °C; ^1^H NMR (500.13 MHz, D_2_O) δ 0.85 (t, *J* = 7.4 Hz, 3H, H-7), 1.32 (sext, *J* = 7.5 Hz, 2H, H-6), 1.61 (p, *J* = 7.5 Hz, 2H, H-5), 3.07 (t, *J* = 7.4 Hz, 2H, H-4), 3.59 (m, 2H, H-2); ^13^C NMR (125.77 MHz, D_2_O) δ 12.7 (C-7), 19.0 (C-6), 27.5 (C-5), 48.1 (C-4), 49.1 (dt, *J* = 18.1, 2.3 Hz, C-2); ^31^P NMR (202.46 MHz, D_2_O) δ 8.84 (d, *J* = 65.5 Hz); ^19^F NMR (470.59 MHz, D_2_O) δ −193.39 (t, *J* = 65.6 Hz). HRMS (ESI) calcd. for (C_6_H_17_O_6_NFP_2_) [M + H]^+^ 280.0515; found 280.0514.

*1-[(n-Pent-1-ylamino)ethyl]-1-fluoro-1,1-bisphosphonic Acid* (**56**): 65% yield; white solid; m.p. 164–165 °C; ^1^H NMR (500.13 MHz, D_2_O) δ 0.81 (t, *J* = 7.1 Hz, 3H, H-8), 1.28 (m, 4H, C*H*_2_), 1.63 (p, *J* = 7.3 Hz, 2H, H-5), 3.06 (t, *J* = 7.4 Hz, 2H, H-4), 3.59 (p, *J* = 10.1 Hz, 2H, H-2); ^13^C NMR (125.77 MHz, D_2_O) δ 13.0 (C-8), 21.4 (C-7), 25.1 (C-5), 27.7 (C-7), 48.3 (C-4), 49.1 (d, *J* = 18.3 Hz, C-2); ^31^P NMR (202.46 MHz, D_2_O) δ 8.93 (d, *J* = 47.3 Hz); ^19^F NMR (470.59 MHz, D_2_O) δ −193.32 (t, *J* = 65.5 Hz). HRMS (ESI) calcd. for (C_7_H_19_O_6_NFP_2_) [M + H]^+^ 294.0672; found 294.0667.

*1-[(n-Hex-1-ylamino)ethyl]-1-fluoro-1,1-bisphosphonic Acid* (**57**): 34% yield; white solid; ^1^H NMR (500.13 MHz, D_2_O) δ 0.77 (t, *J* = 7.1 Hz, 3H, H-9), 1.22 (m, 4H, C*H*_2_), 1.28 (m, 2H, C*H*_2_), 1.61 (p, *J* = 7.5 Hz, 2H, H-5), 3.05 (t, *J* = 7.5 Hz, 2H, H-4), 3.58 (p, *J* = 10.6 Hz, 2H, H-2); ^13^C NMR (125.77 MHz, D_2_O) δ 13.1 (C-9), 21.6 (C-8), 25.2 (C-6), 25.4 (C-5), 30.3 (C-7), 49.1 (dt, *J* = 18.1, 2.3 Hz, C-2), 92.2 (dt, *J* = 184.1, 139.4 Hz, C-1); ^31^P NMR (202.46 MHz, D_2_O) δ 8.91 (d, *J* = 59.7 Hz); ^19^F NMR (470.59 MHz, D_2_O) δ −193.54 (t, *J* = 65.9 Hz). HRMS (ESI) calcd. for (C_8_H_21_O_6_NFP_2_) [M + H]^+^ 308.0823; found 308.0822.

*1-[(n-Hept-1-ylamino)ethyl]-1-fluoro-1,1-bisphosphonic Acid* (**58**): 56% yield; white solid; m.p. 135–136 °C; ^1^H NMR (500.13 MHz, D_2_O) δ 0.76 (t, *J* = 7.1 Hz, 3H, H-10), 1.20 (m, 4H, C*H*_2_), 1.27 (m, 4H, C*H*_2_), 1.61 (p, *J* = 7.4 Hz, 2H, H-5), 3.06 (t, *J* = 7.7 Hz, 2H, H-4), 3.59 (p, *J* = 10.7 Hz, 2H, H-2); ^13^C NMR (125.77 MHz, D_2_O) δ 13.2 (C-10), 21.8 (C-9), 25.4 (C-6), 25.5 (C-7), 27.8 (C-5), 30.7 (C-8), 48.4 (C-4), 49.0 (dt, *J* = 17.9, 2.3 Hz, C-2), 92.1 (dt, *J* = 184.2 Hz, 139.6 Hz, C-1); ^31^P NMR (202.46 MHz, CDCl_3_) δ 8.90 (d, *J* = 60.8 Hz); ^19^F NMR (470.59 MHz, D_2_O) −193.65 (t, *J* = 66.0 Hz). HRMS (ESI) calcd. for (C_9_H_23_O_6_NFP_2_) [M + H]^+^ 322.0985; found 322.0969.

*1-[(n-Oct-1-ylamino)ethyl]-1-fluoro-1,1-bisphosphonic Acid* (**59**): 31% yield; white solid; m.p. >270 °C; ^1^H NMR (500.13 MHz, D_2_O) δ 0.74 (t, *J* = 6.9 Hz, 3H, H-11), 1.19 (m, 10H, C*H*_2_), 1.59 (p, *J* = 7.4 Hz, 2H, H-5), 3.02 (t, *J* = 7.6 Hz, 2H, H-4), 3.57 (p, *J* = 10.2 Hz, 2H, H-2); ^13^C NMR (125.77 MHz, D_2_O) δ 13.4 (C-11), 22.0 (C-10), 25.4 (C-6), 25.6 (C-7), 28.1 (C-8), 28.2 (C-5), 31.0 (C-9), 48.2 (C-4), 48.9 (d, *J* = 18.1 Hz, C-2), 92.1 (dt, *J* = 184.6 Hz, 139.8 Hz, C-1); ^31^P NMR (202.46 MHz, CDCl_3_) δ 8.87 ppm (d, *J* = 61.0 Hz); ^19^F NMR (470.59 MHz, D_2_O) δ −193.71 (t, *J* = 66.1 Hz).

*1-[(n-Non-1-ylamino)ethyl]-1-fluoro-1,1-bisphosphonic Acid* (**60**). White solid; m.p. 147 °C; ^1^H NMR (500.13 MHz, D_2_O) δ 0.77 (t, *J* = 7.1 Hz, 3H, H-12), 1.19 (m, 12H, C*H*_2_), 1.62 (p, *J* = 6.67 Hz, 2H, H-5), 3.06 (t, *J* = 7.1 Hz, 2H, H-4), 3.59 (m, 2H, H-2); ^13^C NMR (125.77 MHz, D_2_O) δ 13.4 (C-12), 22.0 (C-11), 25.4 (C-6), 25.5 (C-7), 28.1 (C-9), 28.3 (C-8), 28.4 (C-5), 31.1 (C-10), 48.4 (C-4), 49.1 (d, *J* = 18.1 Hz, C-2), 92.3 (dt, *J* = 184.9 Hz, 139.4 Hz, C-1); ^31^P NMR (202.46 MHz, CDCl_3_) δ 8.83 ppm (d, *J* = 65.7 Hz); ^19^F NMR (470.59 MHz, D_2_O) δ −193.34 (t, *J* = 65.6 Hz). HRMS (ESI) calcd. for (C_11_H_27_O_6_NFP_2_) [M + H]^+^ 350.1298; found 350.1306.

*1-[(n-Dec-1-ylamino)ethyl]-1-fluoro-1,1-bisphosphonic Acid* (**61**): 54% yield; White solid; m.p. 151 °C; ^1^H NMR (500.13 MHz, D_2_O) δ 0.78 (t, *J* = 6.7 Hz, 3H, H-13), 1.20 (m, 10H, C*H*_2_), 1.27 (m, 4H, C*H*_2_), 1.64 (p, *J* = 7.3 Hz, 2H, H-5), 3.06 (t, *J* = 7.0 Hz, 2H, H-4), 3.62 (m, 2H, H-2); ^13^C NMR (125.77 MHz, D_2_O) δ 13.4 (C-13), 22.0 (C-12), 25.4 (C-6), 25.5 (C-7), 28.1 (C-10), 28.4 (C-8,C-9), 28.6 (C-5), 31.1 (C-11), 48.4 (C-4), 49.1 (dt, *J* = 18.1, 2.3 Hz, C-2); ^31^P NMR (202.46 MHz, CDCl_3_) δ 8.75 ppm (d, *J* = 65.6 Hz); ^19^F NMR (470.59 MHz, D_2_O) δ −199.16 (t, *J* = 65.8 Hz). HRMS (ESI) calcd. for (C_12_H_23_O_6_NFP_2_) [M + H]^+^ 364.1454; found 364.1443.

## 5. Drug Screening

### 5.1. T. cruzi Amastigote Assays

These experiments were done as reported using tdTomato labeled trypomastigotes with the modifications described by Recher et al., 2013 [38]. ED_50_ values were determined by non-linear regression analysis using SigmaPlot.

### 5.2. T. gondii Tachyzoites Assays

Experiments on *T. gondii* tachyzoites were carried out as described previously using *T. gondii* tachyzoites expressing red fluorescent protein with the modifications described by Recher et al., 2013 [38]. Plates were read with covered lids, and both excitation (544 nm) and emission (590 nm) were read from the bottom.

### 5.3. Cytotoxicity for Vero cells

The cytotoxicity was tested using the Alamar Blue™ assay as described by Recher et al., 2013 [38].

### 5.4. TcFPPS and TgFPPS assays and product analysis

The enzymatic activity of the target enzymes was performed according to our previous studies as described for Szajnman et al., 2008 [34].

## 6. Conclusions

In this paper, we have described the synthesis and biological evaluation of isoprenoid and linear 1,1-bisphosphonates against *T. cruzi* and *T. gondii* cells as well as the target enzymes *Tc*FPPS and *Tg*FPPS. The title compounds exhibited marginal antiparasitic activity. However, the biological data here presented provide insights concerning structure–activity relationship. Clearly, the simple replacement of the hydrogen atom by a fluorine atom had a dramatic effect on the biological activity, giving rise to inactive compounds.

## Figures and Tables

**Table 1:** Biological activity of bisphosphonates previously prepared in our laboratory against *T. cruzi* (amastigotes), *T. gondii* (tachyzoites), *Tc*FPPS, and *Tg*FPPS.

Compound	*T. cruzi* Growth ED_50_ (µM)	*Tc*FPPS IC_50_ (µM)	*T. gondii* Growth ED_50_ (µM)	*Tg*FPPS IC_50_ (µM)
**10**	>10.0	>1.0	2.67 ± 11.93	0.035
**11**	>10.0	>1.0	>10.0	0.060
**12**	0.84	0.49	9.4	0.14
**13**	10.0	0.058	>50.0	0.095
**14**	0.67	0.81	6.23	0.093
**15**	>10.0	>10.0	2.0	0.039
**16**	>20.0	0.097	1.8	0.021
**17**	>20.0	>10.0	5.0	0.009
**18**	>20.0	0.040	7.0	0.013

**Table 2:** Biological activity of bisphosphonates against *T. cruzi* (amastigotes), *T. gondii* (tachyzoites), *Tc*FPPS, *Tg*FPPS, *human* FPPS (*Hh*FPPS) and Vero cells ^‡^.

Compound	*T. cruzi* Growth ED_50_ (µM)	*Tc*FPPS IC_50_ (µM)	*T. gondii* Growth ED_50_ (µM)	*Tg*FPPS IC_50_ (µM)	*Hh*FPPS IC_50_ (µM)	Cytotoxicity ED_50_ (µM)
**19**	>20	4.5 ± 2.8	22.28 ± 11.93	0.183 ± 0.063	6.6 ± 2.4	>200
**44**	>10.0	NT	>10.0	0.313 ± 0.027	NT	NT
**45**	>10.0	NT	>10.0	NT	NT	NT
**54**	>10.0	NT	NT	NT	NT	NT
**55**	>10.0	0.097 ± 0.020	>10.0	0.091 ± 0.032	NT	>200
**56**	>10.0	NT	>10.0	NT	NT	NT
**57**	>10.0	NT	>10.0	NT	NT	NT
**58**	>10.0	NT	>10.0	NT	NT	NT
**59**	>10.0	NT	4.008 ± 1.191	NT	NT	161.7 ± 12.4
**60**	cytotoxic †	NT		NT	NT	15.0
**61**	cytotoxic †	NT		NT	NT	20.0
**benznidazole**	1.80 ± 0.39					

^‡^ Maximum concentration tested. NT = not tested. † visually.

## Supplementary Materials

Copies of the ^1^H NMR, ^13^C NMR, ^31^P NMR and ^19^F NMR spectra of the target molecules and the corresponding intermediates are included as supporting information. They are available online at http://www.mdpi.com/1420-3049/22/1/82/s1.
