# Supplementary material for: Antiparasitic Activity of Sulfur- and Fluorine-Containing Bisphosphonates against Trypanosomatids and Apicomplexan Parasites
Source: Molecules. 2017 Jan 4;22(1):82. doi: 10.3390/molecules22010082 (PMC6155738; doi:10.3390/molecules22010082)
Supplement: Supplementary file 1 [file molecules-22-00082-s001.pdf]

Supplementary Materials: Antiparasitic Activity of Sulfur- and Fluorine-Containing Bisphosphonates against Trypanosomatids and Apicomplexan Parasites

Tamila Galaka, Mariana Ferrer Casal, Melissa Storey, Catherine Li, María N. Chao, Sergio H. Szajnman, Roberto Docampo, Silvia N. J. Moreno, and Juan B. Rodriguez

List of Contents **Page**

^{1}H NMR spectrum of compound **22**.	S4
^{13}C NMR spectrum of compound **22**.	S4
^{31}P NMR spectrum of compound **22**.	S5
^{1}H NMR spectrum of compound **19**.	S5
^{13}C NMR spectrum of compound **19**.	S6
^{31}P NMR spectrum of compound **19**.	S6
^{1}H NMR spectrum of compound **25**.	S7
^{13}C NMR spectrum of compound **25**	S7
^{1}H NMR spectrum of compound **27**	S8
^{13}C NMR spectrum of compound **27**	S8
^{1}H NMR spectrum of compound **29**	S9
^{13}C NMR spectrum of compound **29**	S9
^{1}H NMR spectrum of compound **30**	S10
^{13}C NMR spectrum of compound **30**	S10
^{1}H NMR spectrum of compound **31**	S11
^{13}C NMR spectrum of compound **31**	S11
^{1}H NMR spectrum of compound **32**	S12
^{13}C NMR spectrum of compound **32**	S12
^{1}H NMR spectrum of compound **33**	S13
^{13}C NMR spectrum of compound **33**	S13
^{1}H NMR spectrum of compound **34**	S14
^{13}C NMR spectrum of compound **34**	S14
^{31}P NMR spectrum of compound **34**	S15
^{1}H NMR spectrum of compound **36**	S15
^{13}C NMR spectrum of compound **36**	S16
^{1}H NMR spectrum of compound **37**	S16
^{1}H NMR spectrum of compound **38**	S17
^{13}C NMR spectrum of compound **38**	S17
^{1}H NMR spectrum of compound **42**	S18
^{13}C NMR spectrum of compound **42**	S18
^{1}H NMR spectrum of compound **43**	S19
^{13}C NMR spectrum of compound **43**	S19
^{31}P NMR spectrum of compound **43**	S20
^{1}H NMR spectrum of compound **46**	S20
^{13}C NMR spectrum of compound **46**	S21

^{31}P NMR spectrum of compound **46** S21
^{1}H NMR spectrum of compound **48** S22
^{13}C NMR spectrum of compound **48** S22
^{1}H NMR spectrum of compound **50** S23
^{13}C NMR spectrum of compound **50** S23
^{1}H NMR spectrum of compound **52** S24
^{13}C NMR spectrum of compound **52** S24
^{31}P NMR spectrum of compound **52** S25
^{1}H NMR spectrum of compound **44** S25
^{13}C NMR spectrum of compound **44** S26
^{31}P NMR spectrum of compound **44** S26
^{1}H NMR spectrum of compound **49** S27
^{13}C NMR spectrum of compound **49** S27
^{1}H NMR spectrum of compound **51** S28
^{13}C NMR spectrum of compound **51** S28
^{1}H NMR spectrum of compound **53** S29
^{13}C NMR spectrum of compound **53** S29
^{31}P NMR spectrum of compound **53** S30
^{1}H NMR spectrum of compound **45** S30
^{13}C NMR spectrum of compound **45** S31
^{31}P NMR spectrum of compound **45** S31
^{1}H NMR spectrum of compound **67** S32
^{13}C NMR spectrum of compound **67** S32
^{31}P NMR spectrum of compound **67** S33
^{1}H NMR spectrum of compound **68** S33
^{13}C NMR spectrum of compound **68** S34
^{31}P NMR spectrum of compound **68** S34
^{1}H NMR spectrum of compound **69** S35
^{13}C NMR spectrum of compound **69** S35
^{31}P NMR spectrum of compound **69** S36
^{1}H NMR spectrum of compound **70** S36
^{13}C NMR spectrum of compound **70** S37
^{31}P NMR spectrum of compound **70** S37
^{1}H NMR spectrum of compound **71** S38
^{13}C NMR spectrum of compound **71** S38
^{31}P NMR spectrum of compound **71** S39
^{1}H NMR spectrum of compound **72** S39
^{13}C NMR spectrum of compound **72** S40
^{31}P NMR spectrum of compound **72** S40
^{1}H NMR spectrum of compound **73** S41
^{13}C NMR spectrum of compound **73** S41
^{31}P NMR spectrum of compound **73** S42
^{1}H NMR spectrum of compound **74** S42
^{13}C NMR spectrum of compound **74** S43
^{31}P NMR spectrum of compound **74** S43

^{1}H NMR spectrum of compound **75** S44
^{13}C NMR spectrum of compound **75** S44
^{31}P NMR spectrum of compound **75** S45
^{19}F NMR spectrum of compound **75** S45
^{1}H NMR spectrum of compound **76** S46
^{13}C NMR spectrum of compound **76** S46
^{31}P NMR spectrum of compound **76** S47
^{19}F NMR spectrum of compound **76** S47
^{1}H NMR spectrum of compound **77** S48
^{13}C NMR spectrum of compound **77** S48
^{31}P NMR spectrum of compound **77** S49
^{19}F NMR spectrum of compound **77** S49
^{1}H NMR spectrum of compound **78** S50
^{13}C NMR spectrum of compound **78** S50
^{31}P NMR spectrum of compound **78** S51
^{19}F NMR spectrum of compound **78** S51
^{1}H NMR spectrum of compound **79** S52
^{13}C NMR spectrum of compound **79** S52
^{31}P NMR spectrum of compound **79** S53
^{19}F NMR spectrum of compound **79** S53
^{1}H NMR spectrum of compound **80** S54
^{13}C NMR spectrum of compound **80** S54
^{31}P NMR spectrum of compound **80** S55
^{19}F NMR spectrum of compound **80** S55
^{1}H NMR spectrum of compound **81** S56
^{13}C NMR spectrum of compound **81** S56
^{31}P NMR spectrum of compound **81** S57
^{19}F NMR spectrum of compound **81** S57
^{1}H NMR spectrum of compound **82** S58
^{13}C NMR spectrum of compound **82** S58
^{31}P NMR spectrum of compound **82** S59
^{19}F NMR spectrum of compound **82** S59
^{1}H NMR spectrum of compound **54** S60
^{13}C NMR spectrum of compound **54** S60
^{31}P NMR spectrum of compound **54** S61
^{19}F NMR spectrum of compound **54** S61
^{1}H NMR spectrum of compound **55** S62
^{13}C NMR spectrum of compound **55** S62
^{31}P NMR spectrum of compound **55** S63
^{19}F NMR spectrum of compound **55** S63
^{1}H NMR spectrum of compound **56** S64
^{13}C NMR spectrum of compound **56** S64
^{31}P NMR spectrum of compound **56** S65
^{19}F NMR spectrum of compound **56** S65
^{1}H NMR spectrum of compound **57** S66

^{13}C NMR spectrum of compound **57** S66
^{31}P NMR spectrum of compound **57** S67
^{19}F NMR spectrum of compound **57** S67
^{1}H NMR spectrum of compound **58** S68
^{13}C NMR spectrum of compound **58** S68
^{31}P NMR spectrum of compound **58** S69
^{19}F NMR spectrum of compound **58** S69
^{1}H NMR spectrum of compound **59** S70
^{13}C NMR spectrum of compound **59** S70
^{31}P NMR spectrum of compound **59** S71
^{19}F NMR spectrum of compound **59** S71
^{1}H NMR spectrum of compound **60** S72
^{13}C NMR spectrum of compound **60** S72
^{31}P NMR spectrum of compound **60** S73
^{19}F NMR spectrum of compound **60** S73
^{1}H NMR spectrum of compound **61** S74
^{13}C NMR spectrum of compound **61** S74
^{31}P NMR spectrum of compound **61** S75
^{19}F NMR spectrum of compound **61** S75

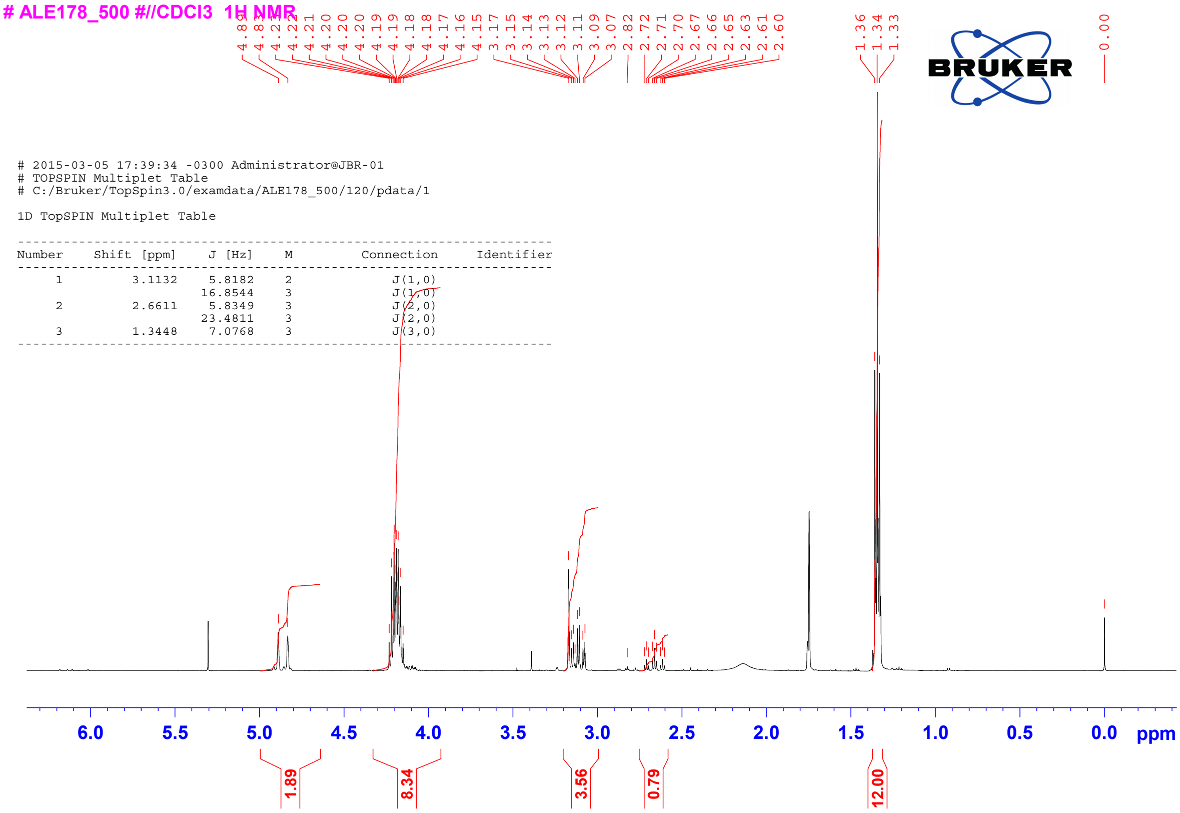

^{1}H NMR spectrum of compound **22**.

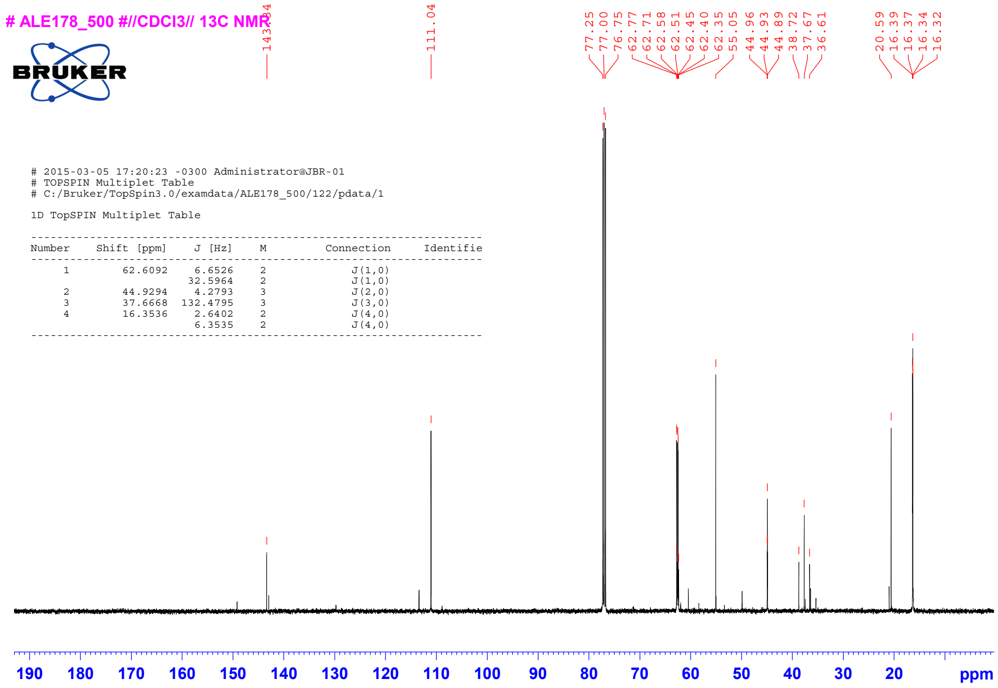

^{13}C NMR spectrum of compound **22**.

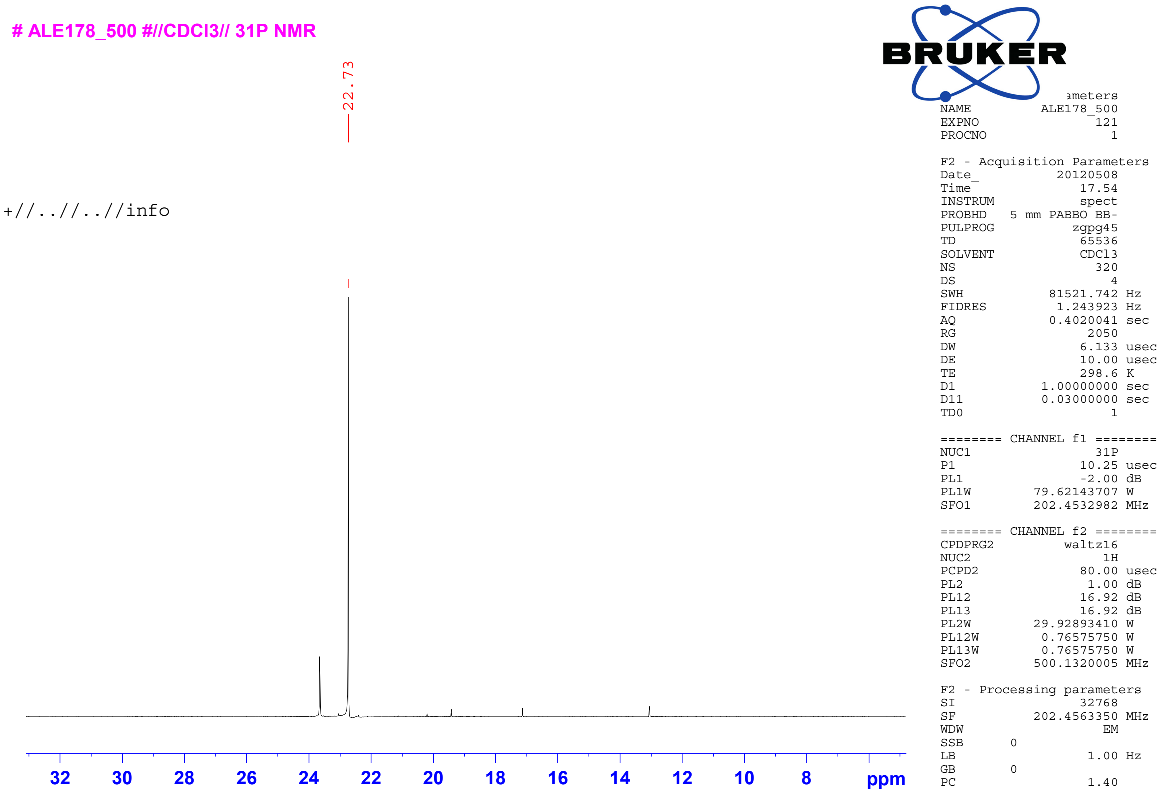

^{31}P NMR spectrum of compound **22**.

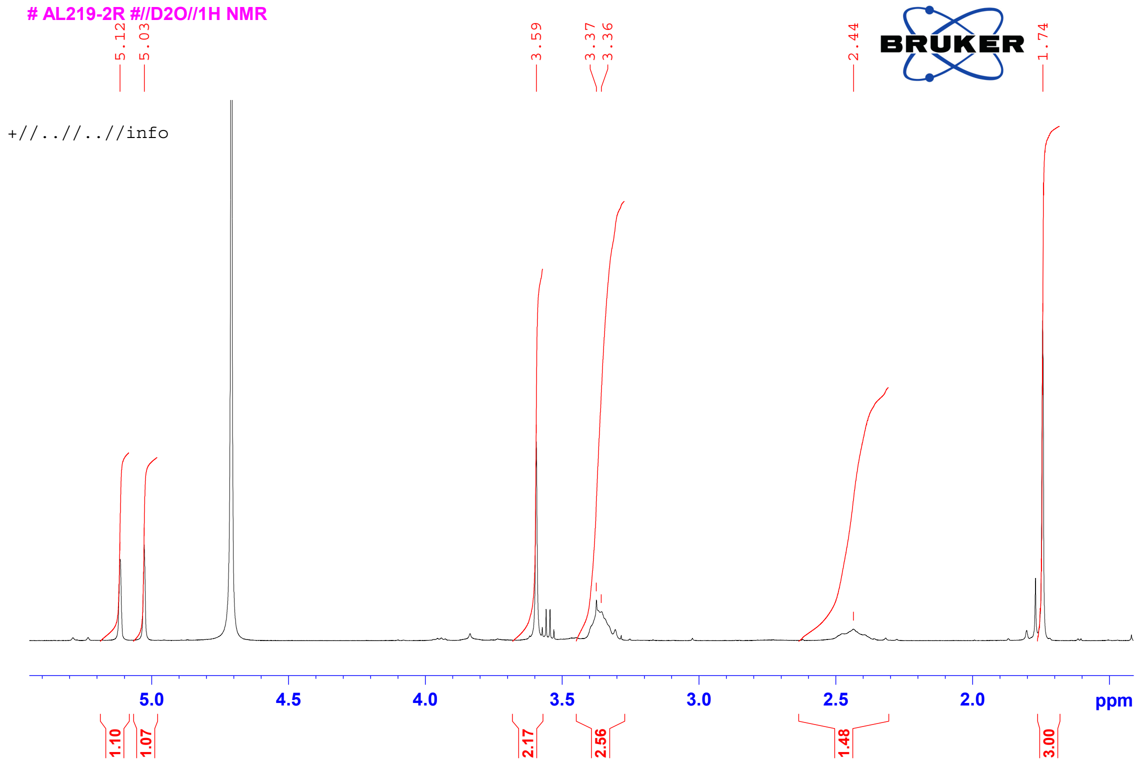

^{1}H NMR spectrum of compound **19**.

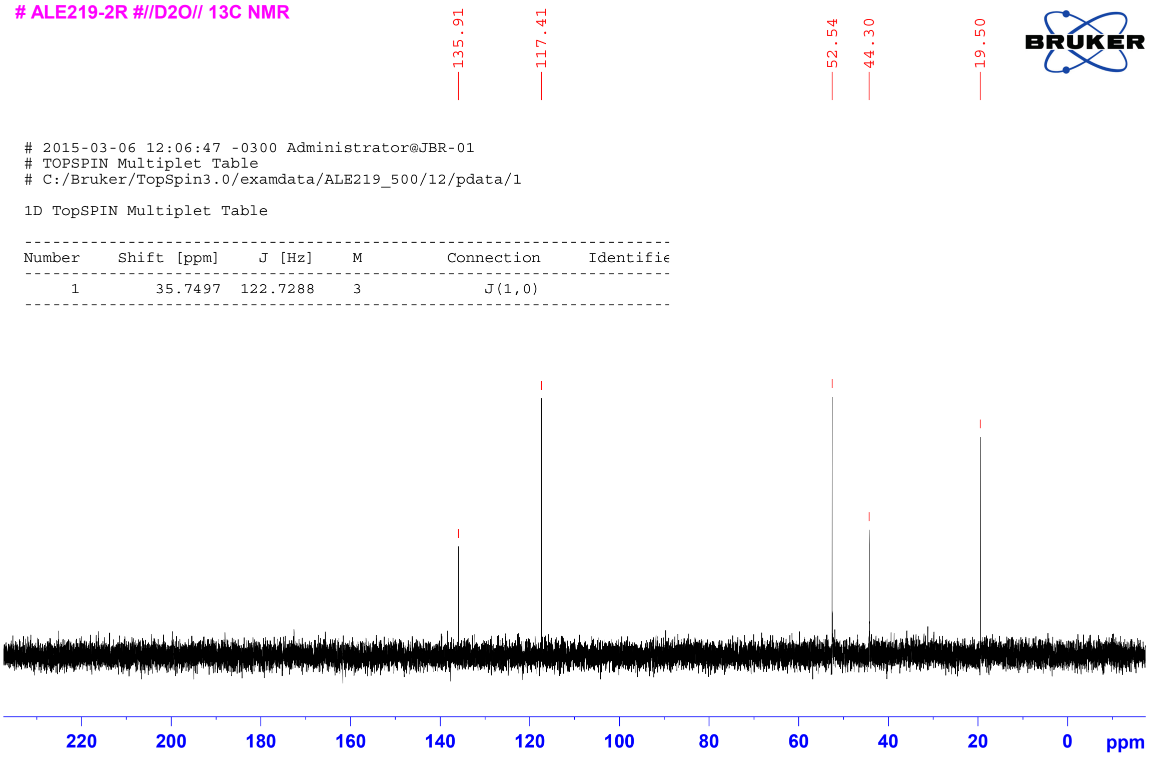

^{13}C NMR spectrum of compound **19**.

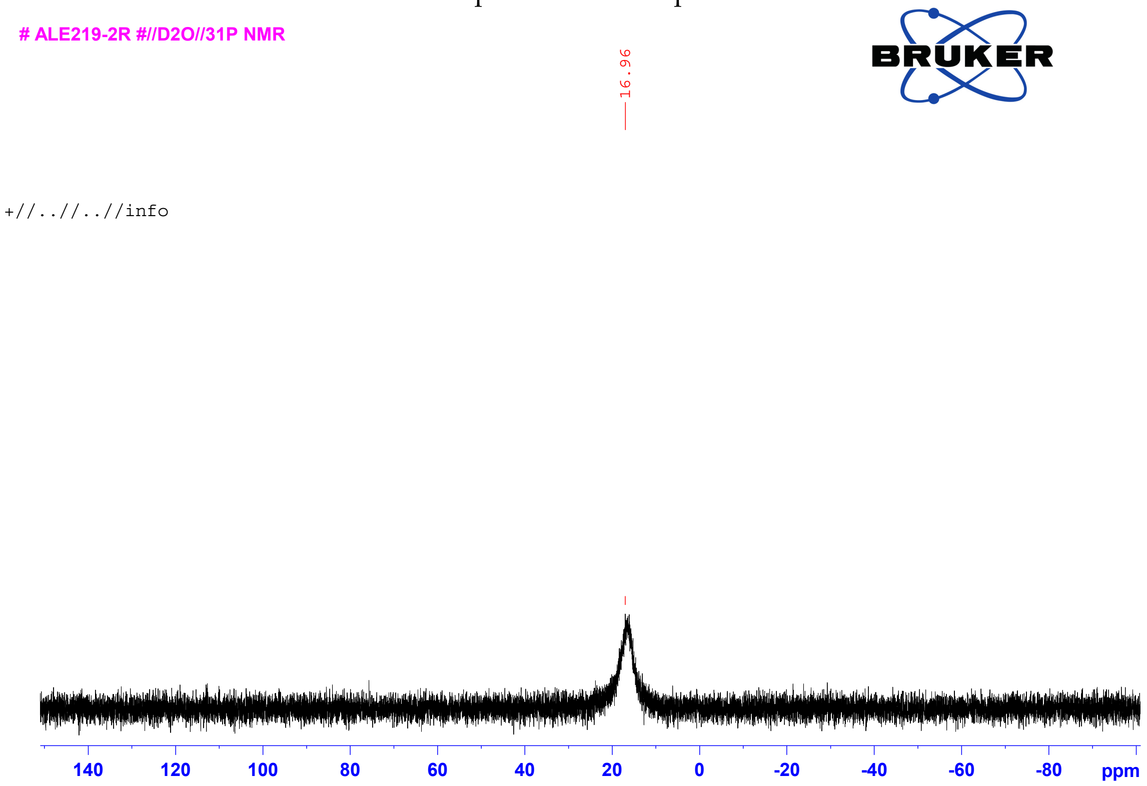

^{31}P NMR spectrum of compound **19**.

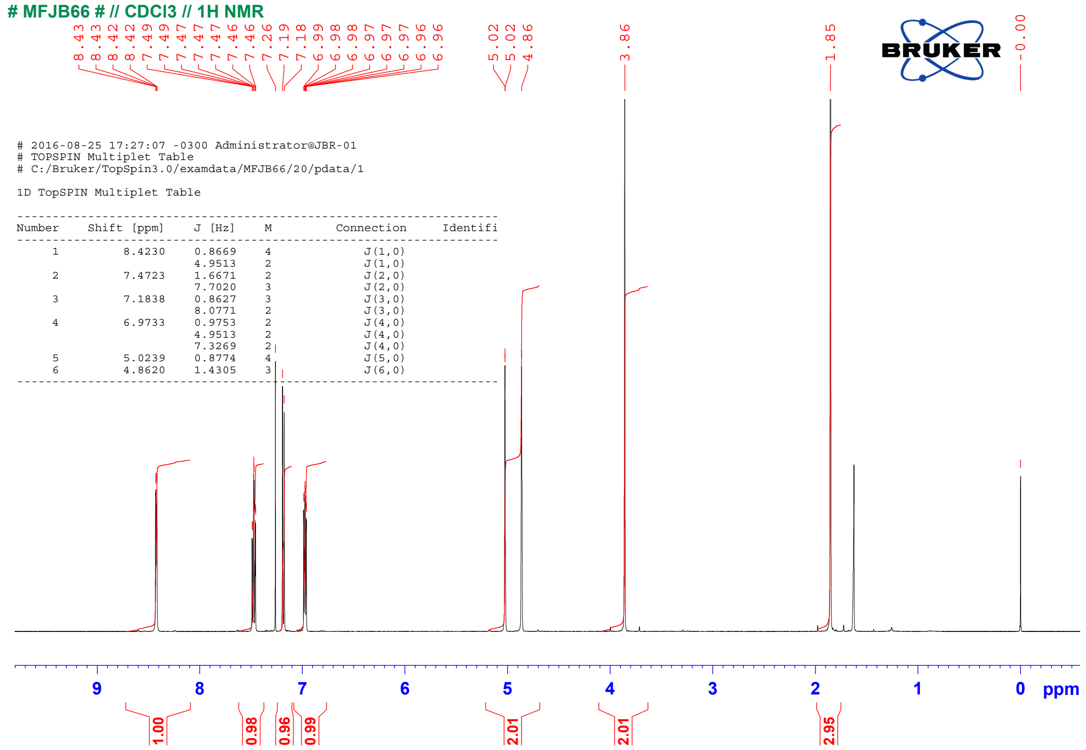

^{1}H NMR spectrum of compound **25**.

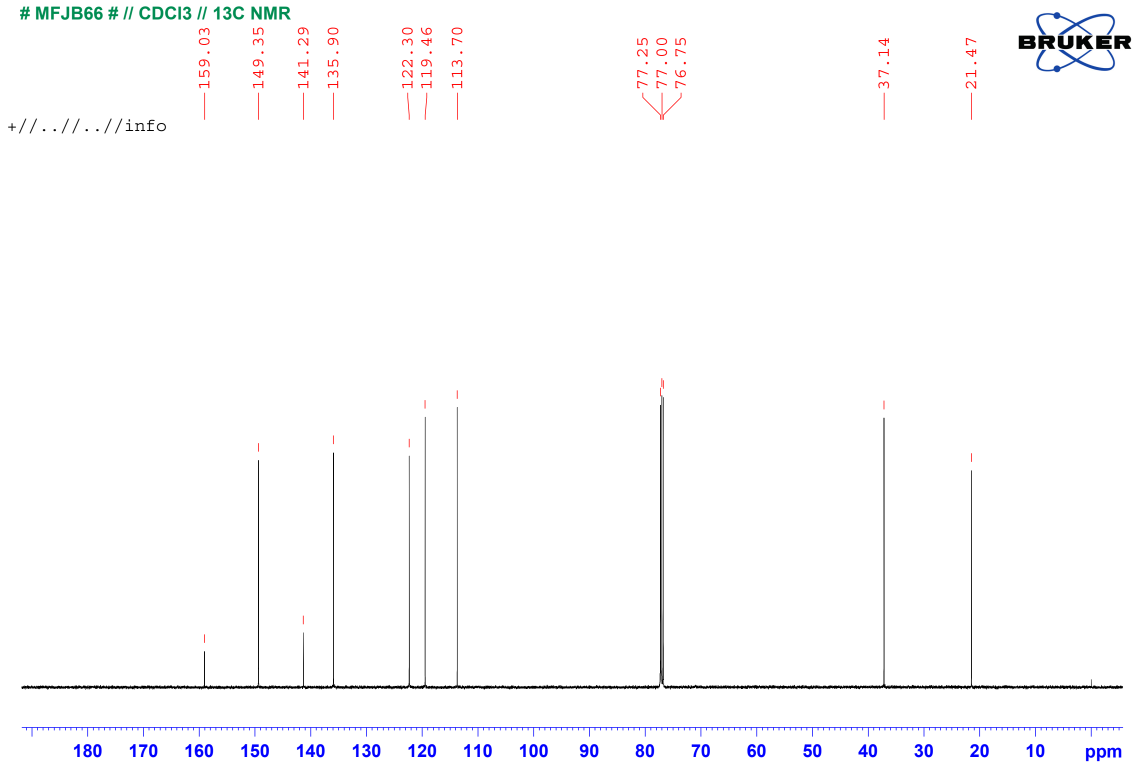

^{13}C NMR spectrum of compound **25**.

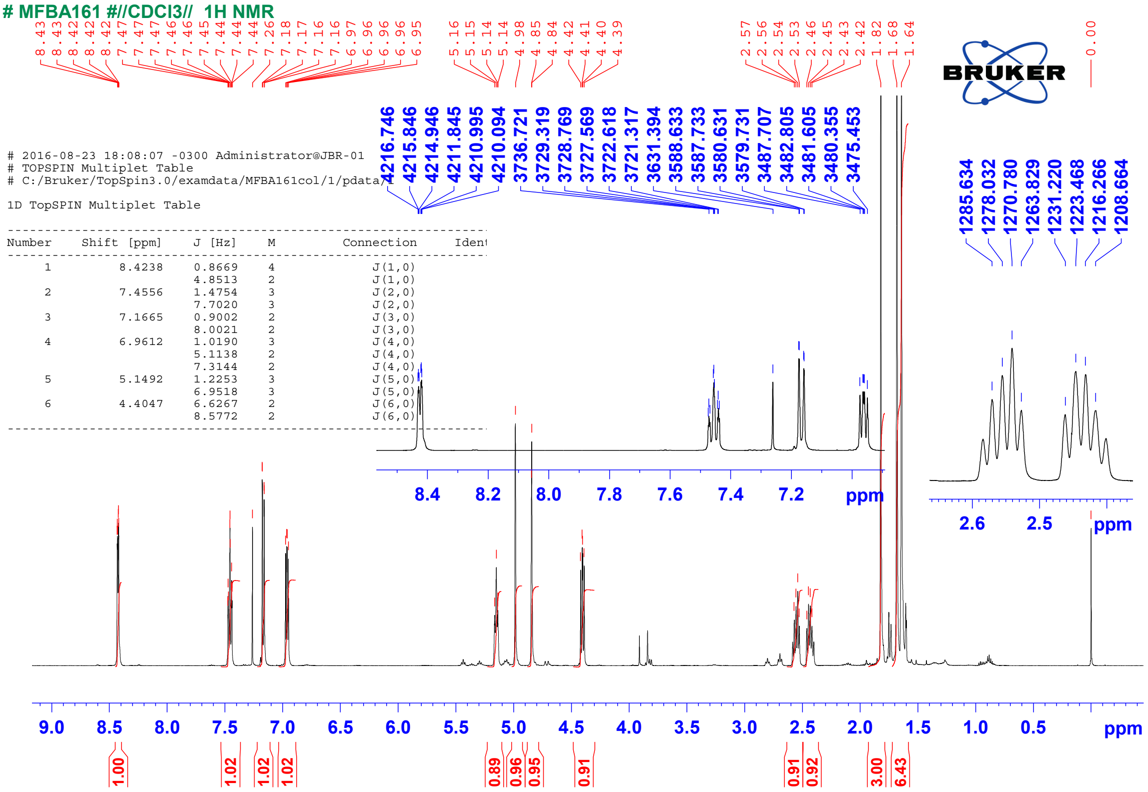

^{1}H NMR spectrum of compound **27**.

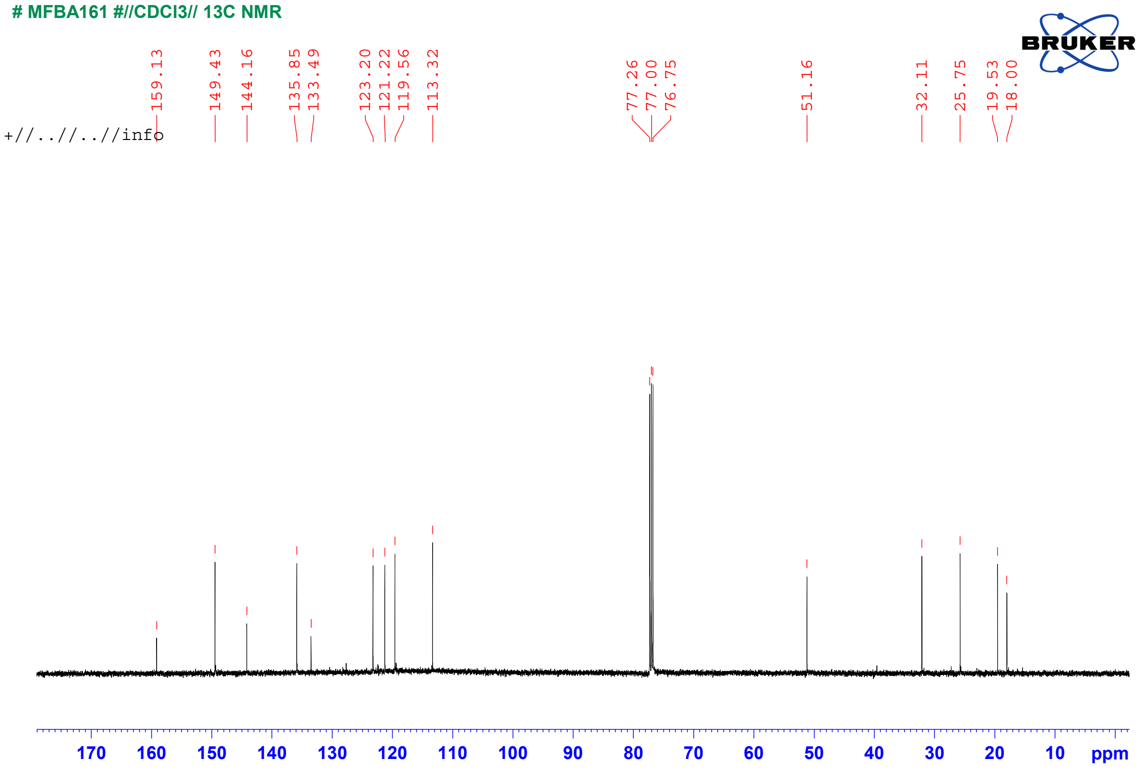

^{13}C NMR spectrum of compound **27**.

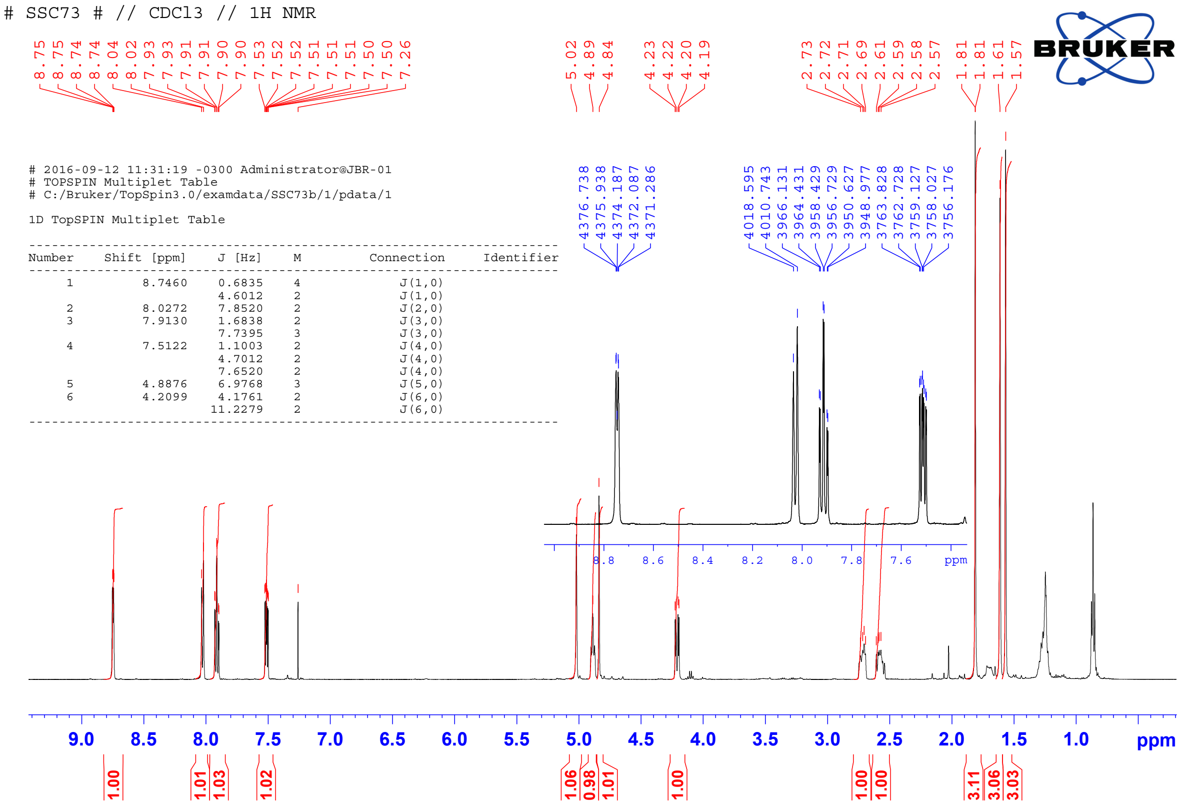

^{1}H NMR spectrum of compound **29**.

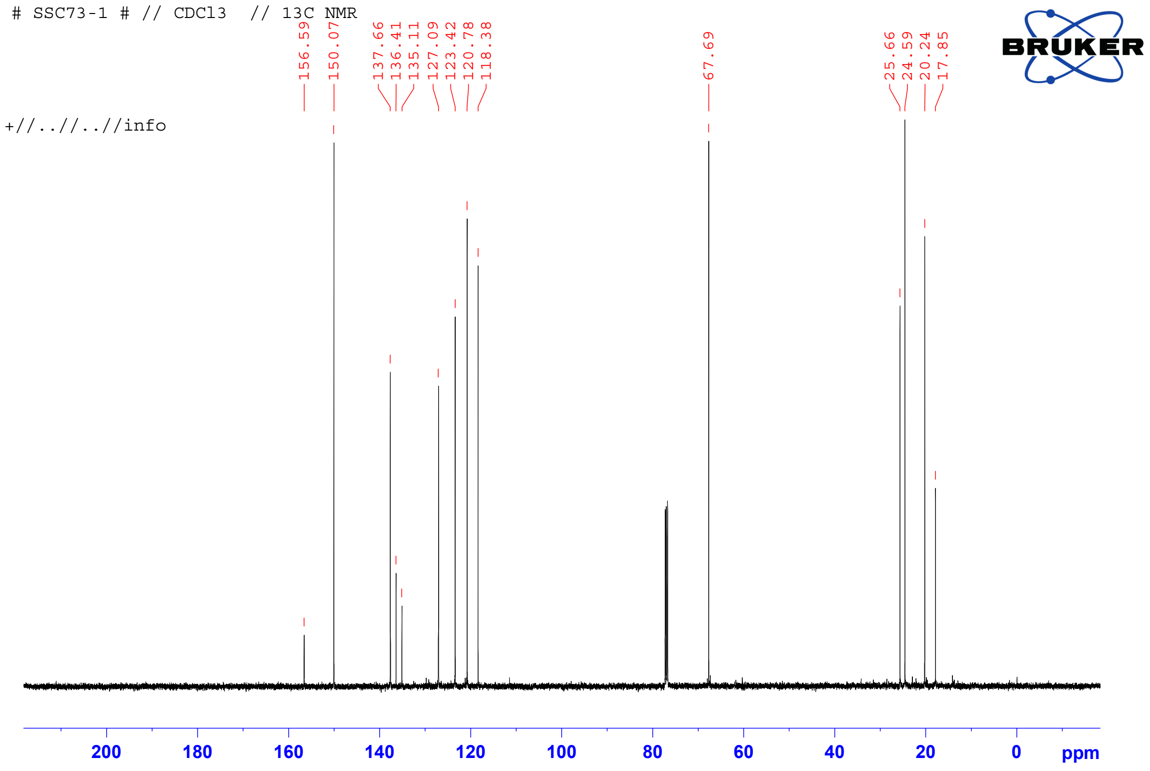

^{13}C NMR spectrum of compound **29**.

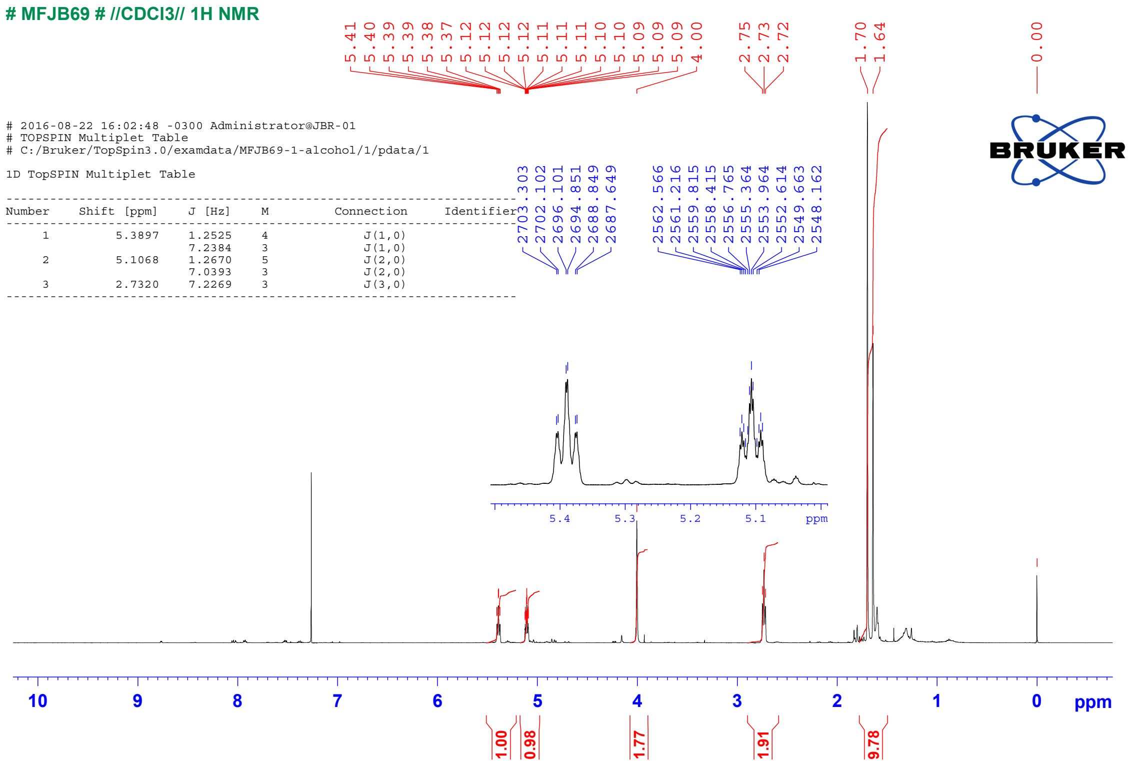

^{1}H NMR spectrum of compound **30**.

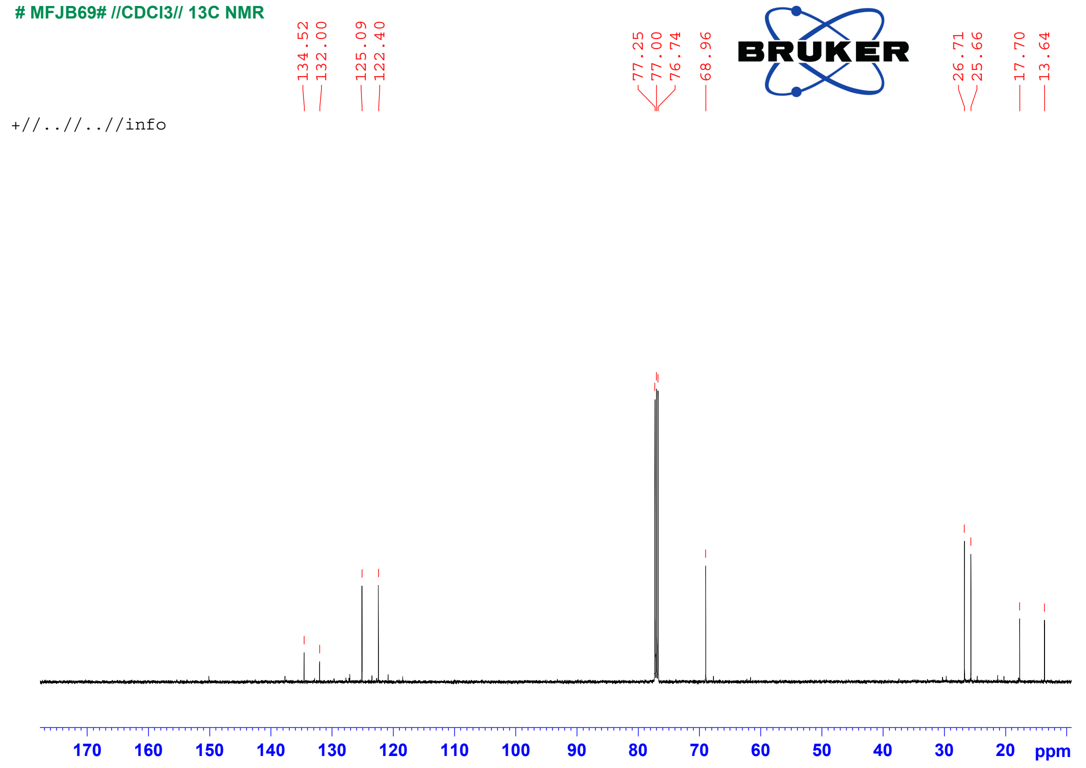

^{13}C NMR spectrum of compound **30**.

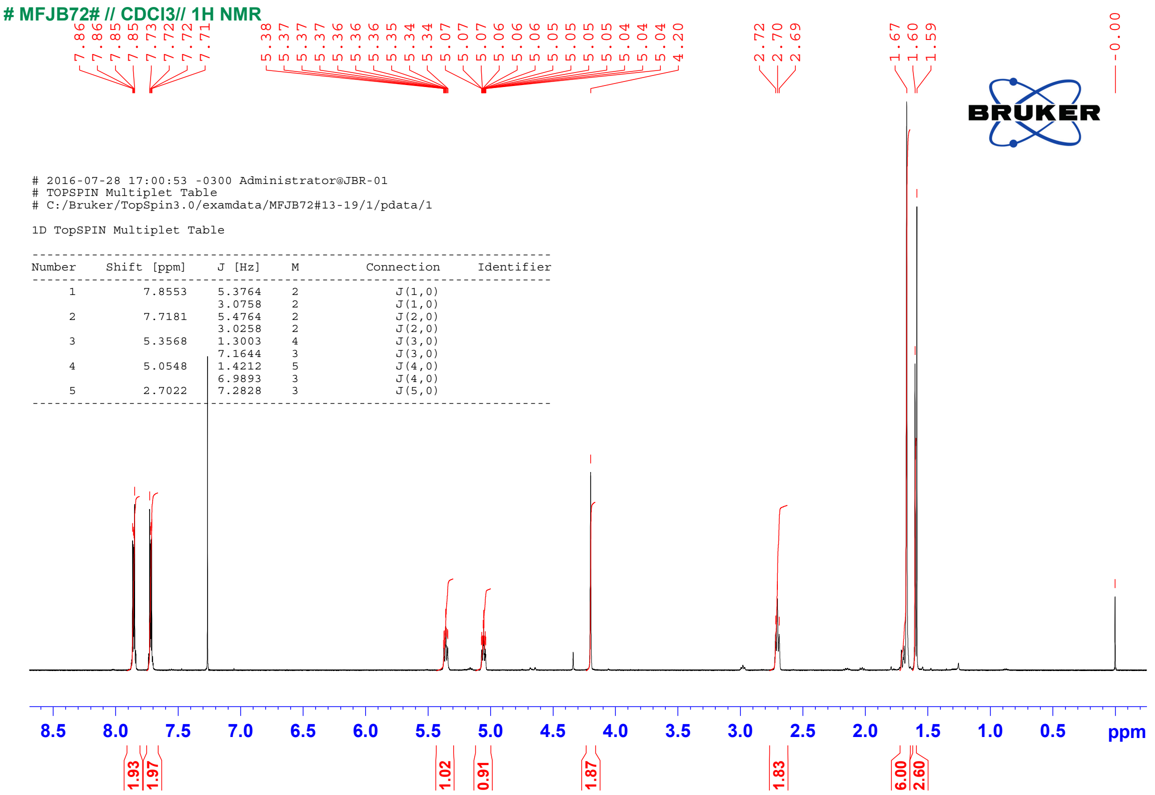

^{1}H NMR spectrum of compound **31**.

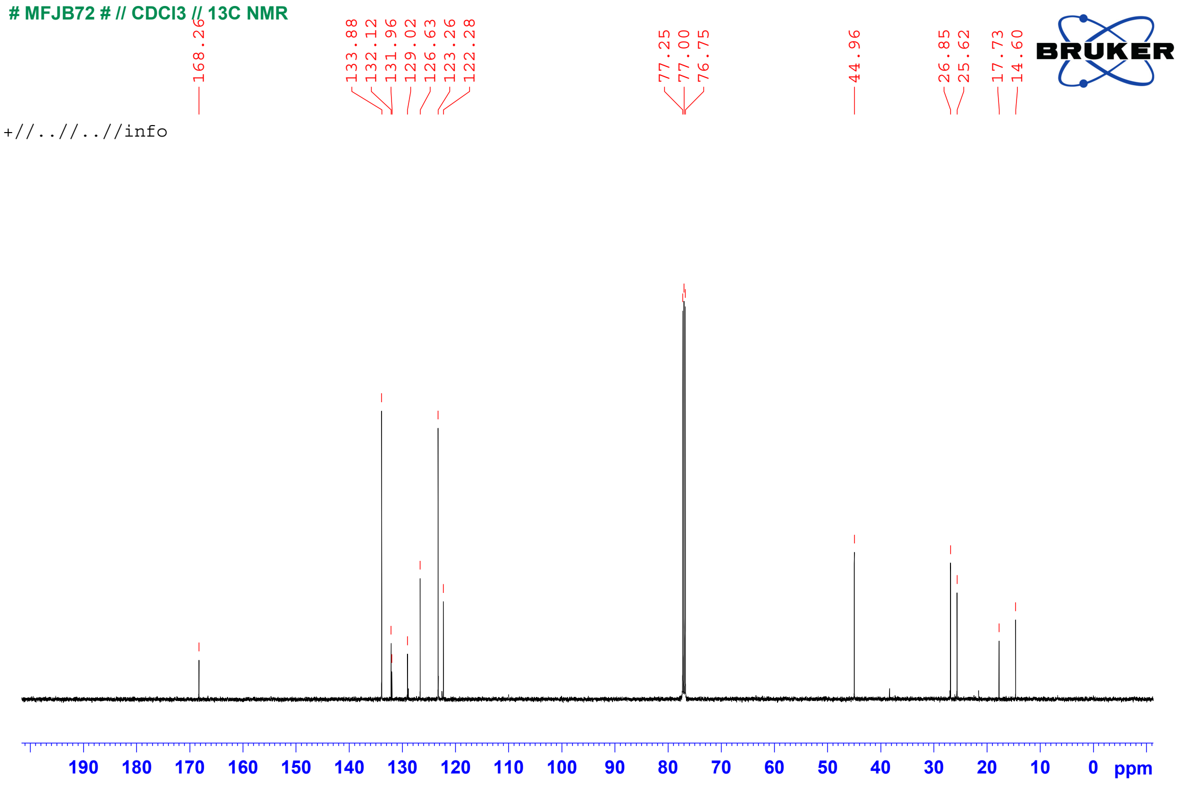

^{13}C NMR spectrum of compound **31**.

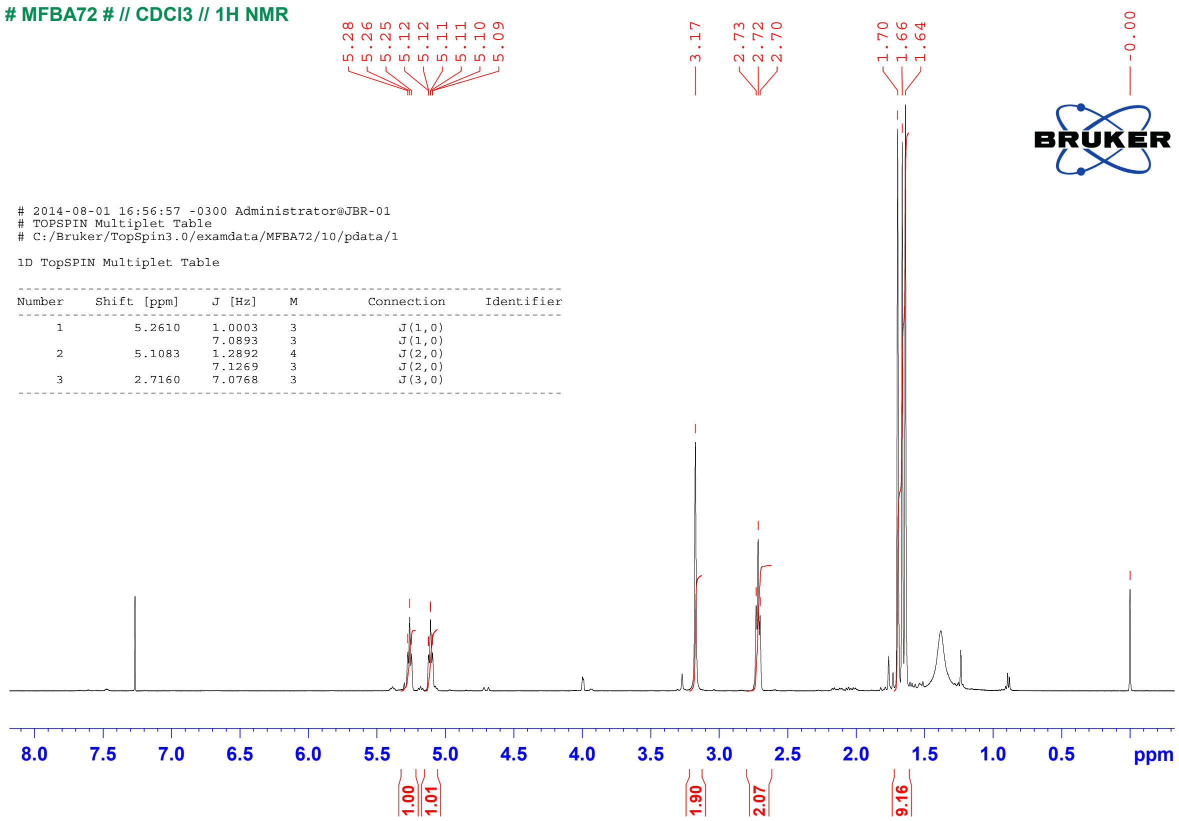

^{1}H NMR spectrum of compound **32**.

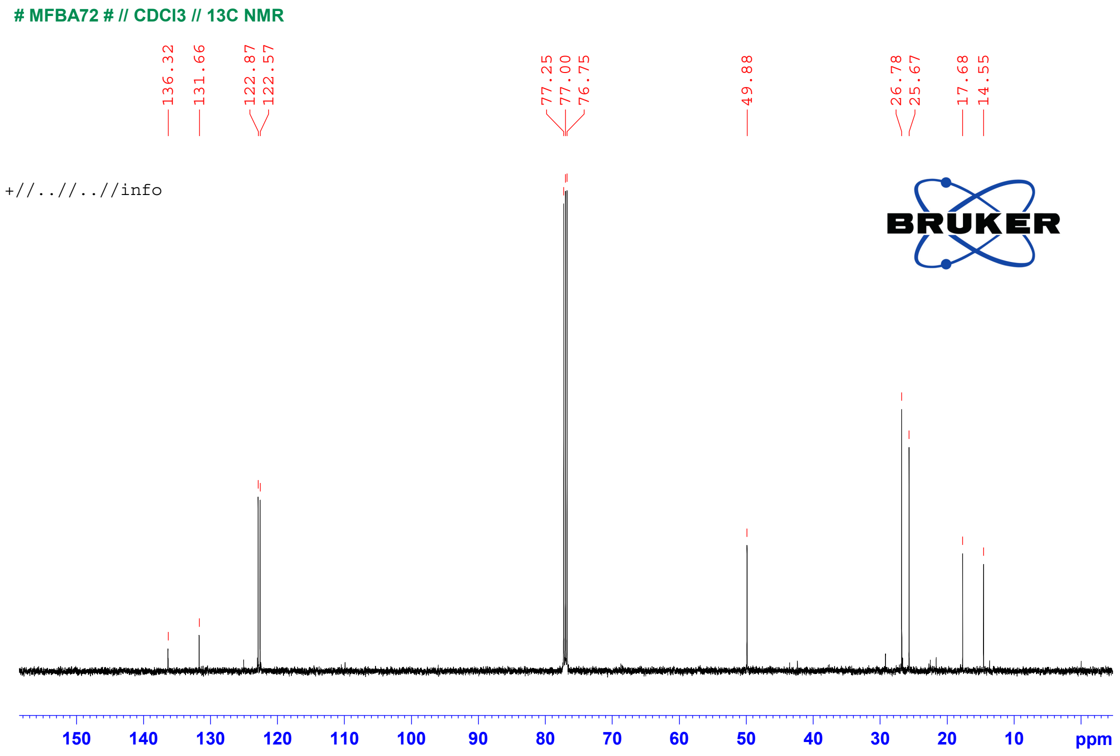

^{13}C NMR spectrum of compound **32**.

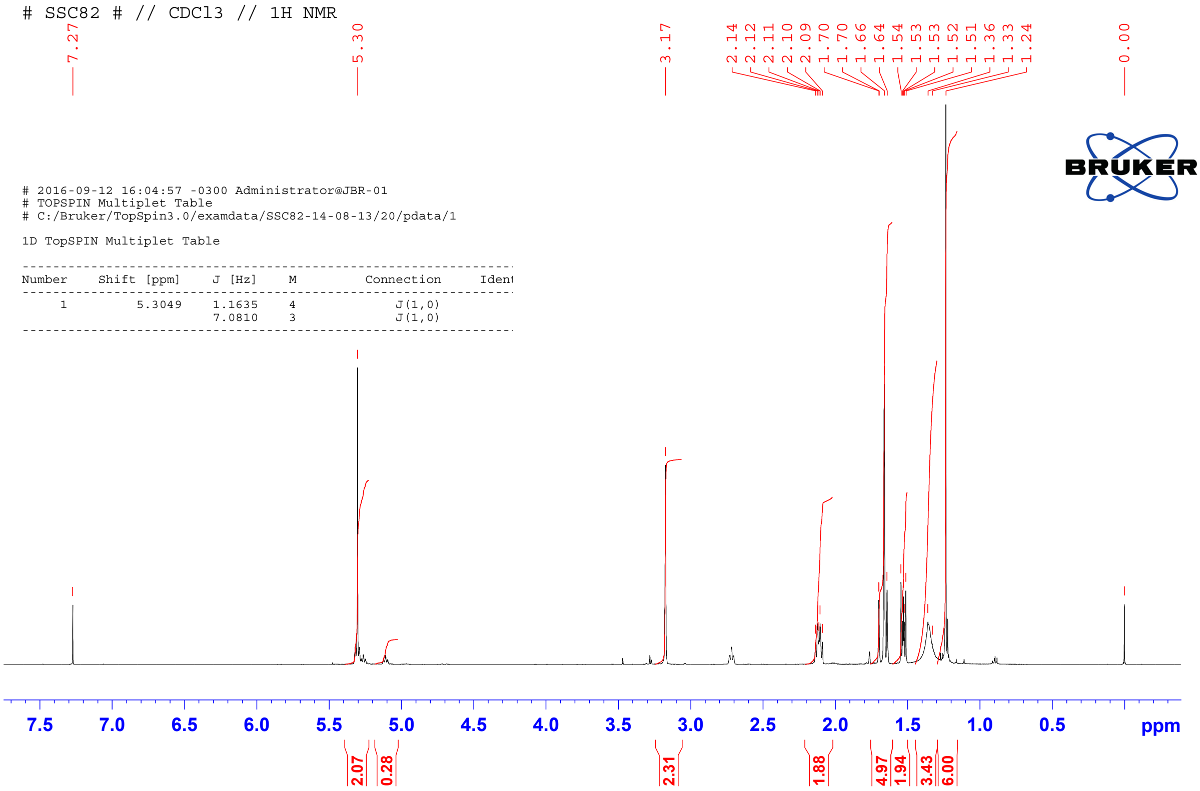

^{1}H NMR spectrum of compound **33**.

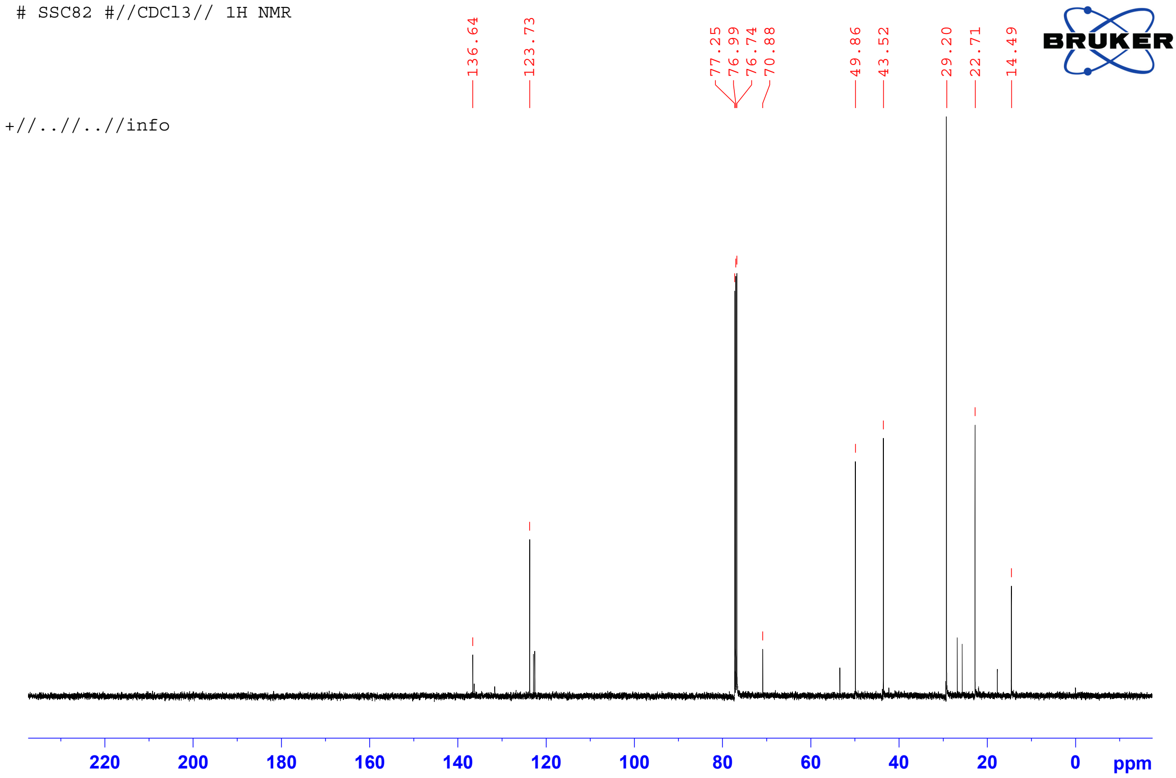

^{13}C NMR spectrum of compound **33**.

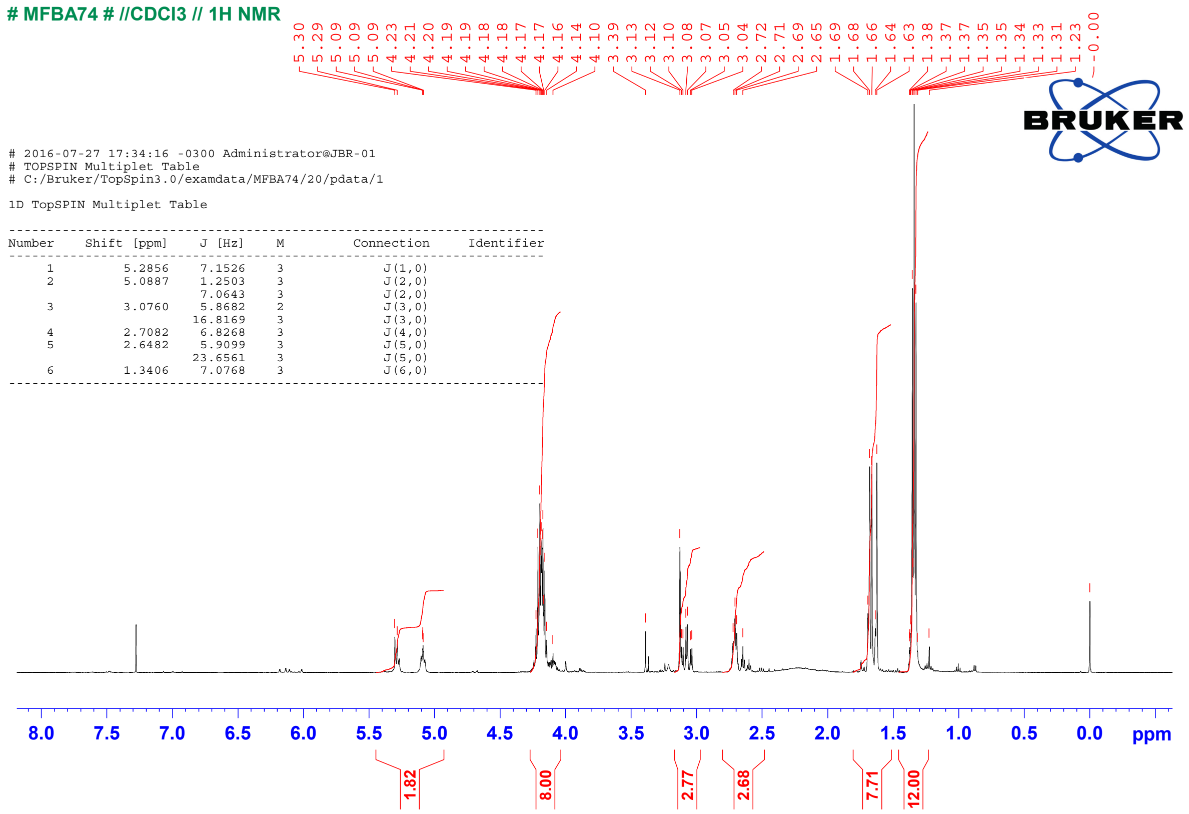

^1H NMR spectrum of compound **34**.

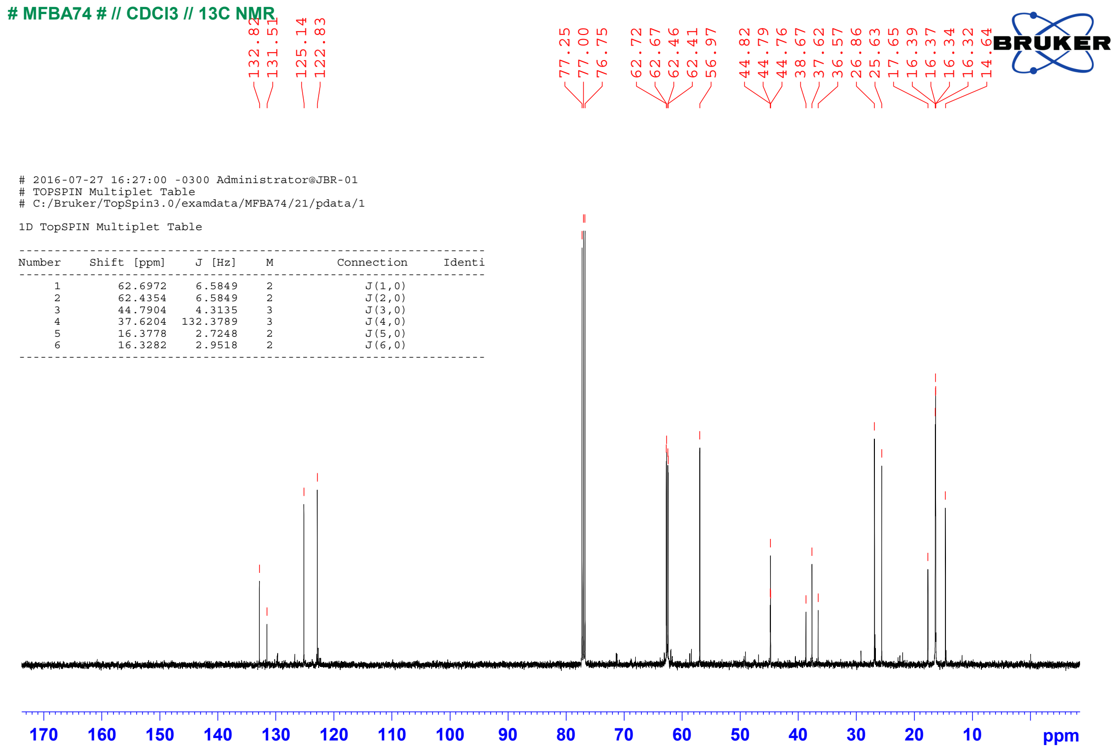

^{13}C NMR spectrum of compound **34**.

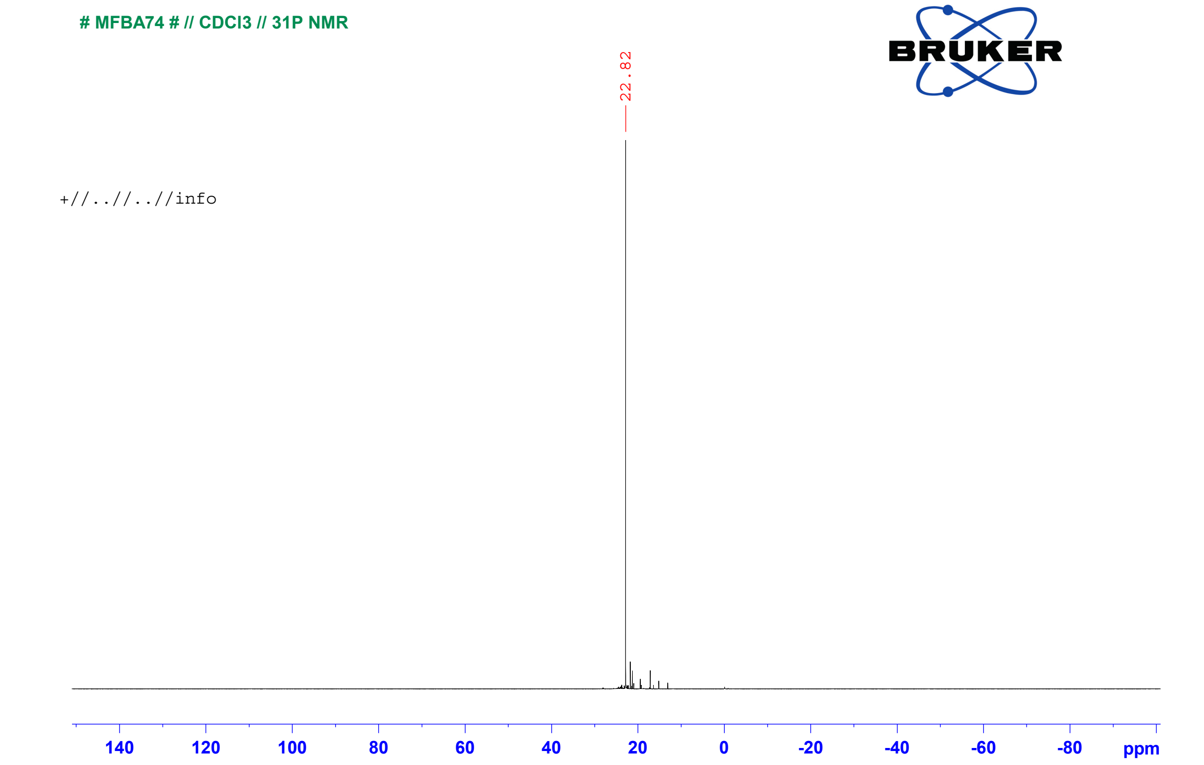

^{31}P NMR spectrum of compound **34**.

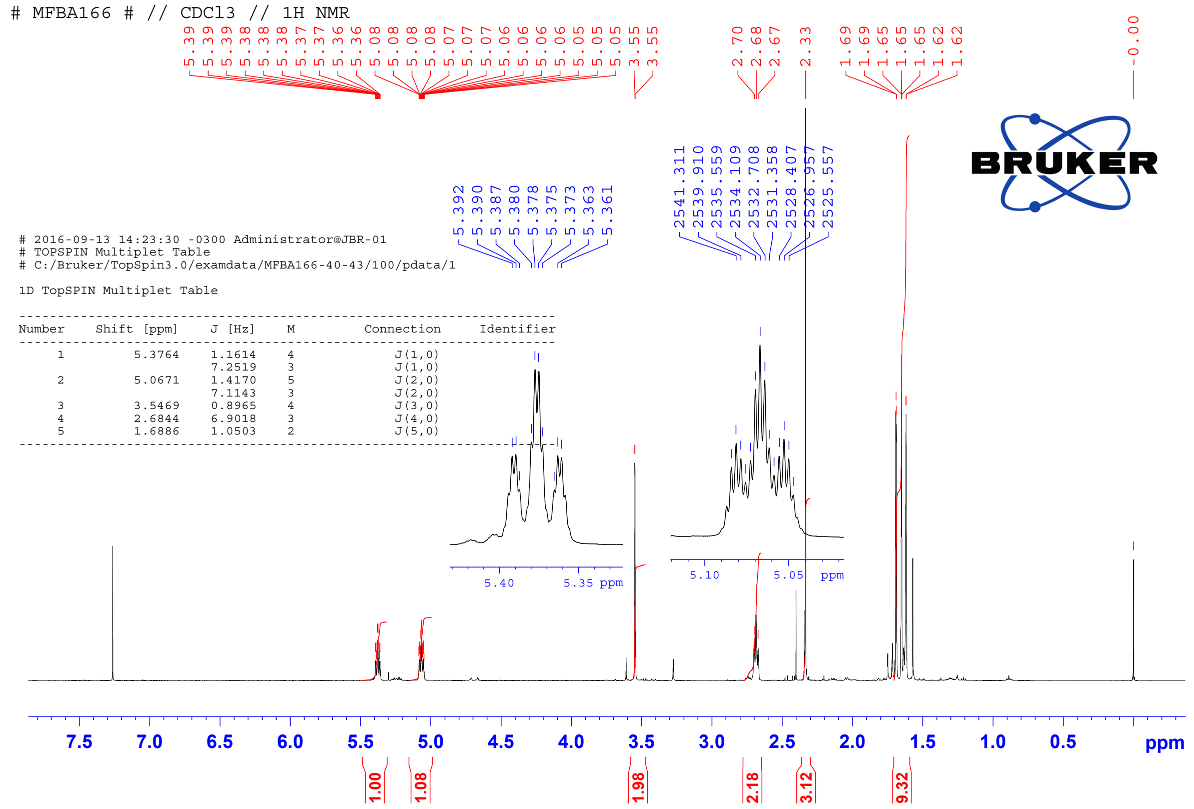

^{1}H NMR spectrum of compound **36**.

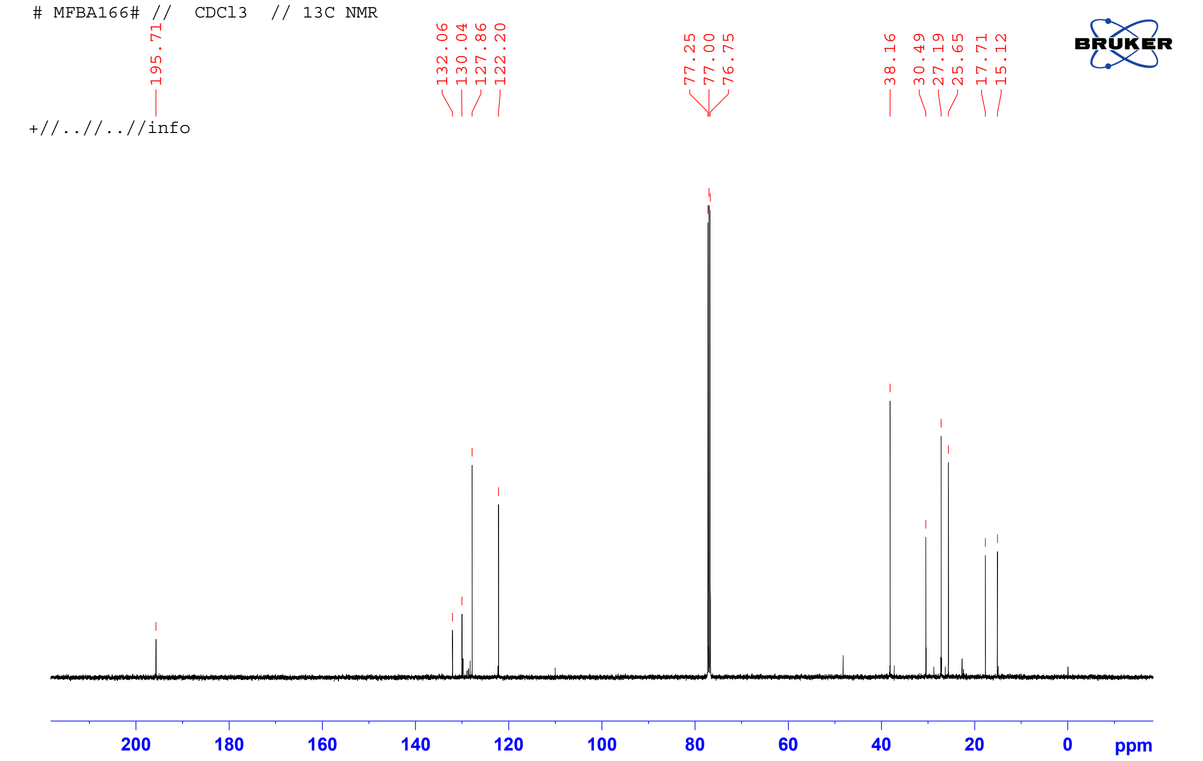

^{13}C NMR spectrum of compound **36**.

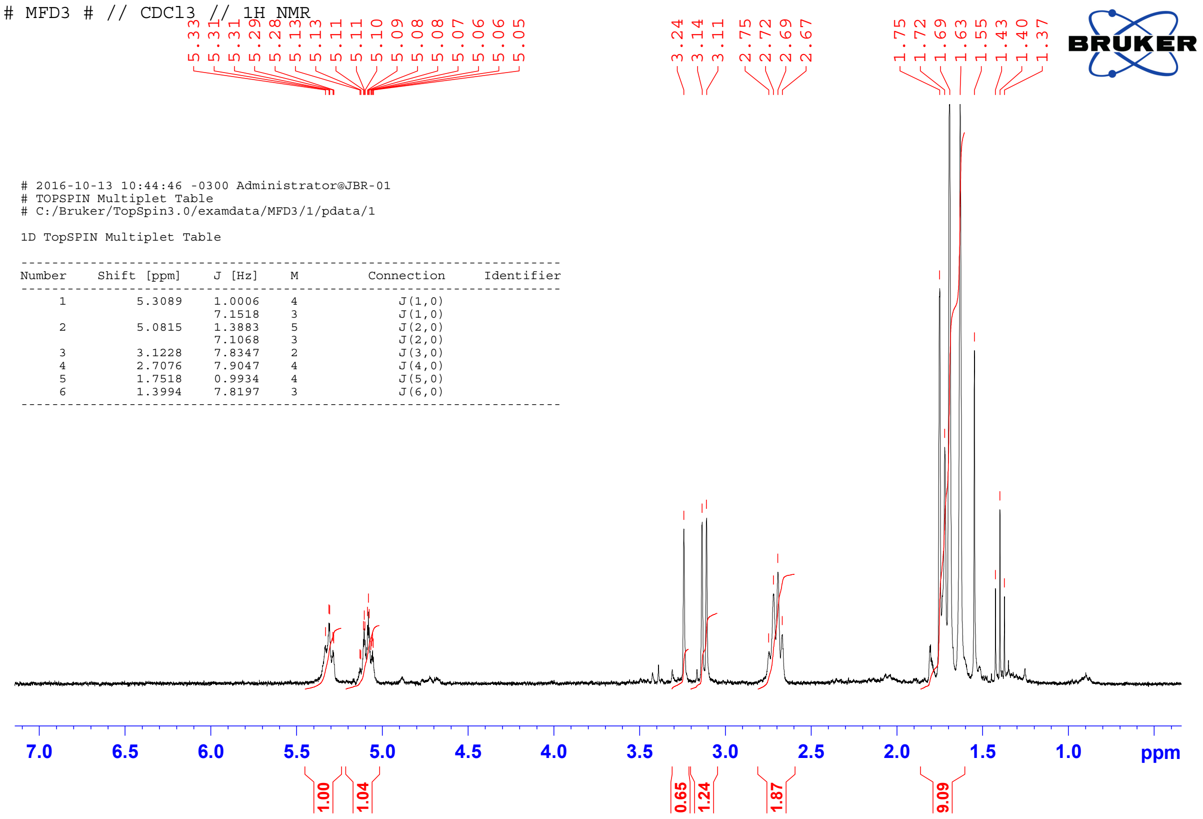

^{1}H NMR spectrum of compound **37**.

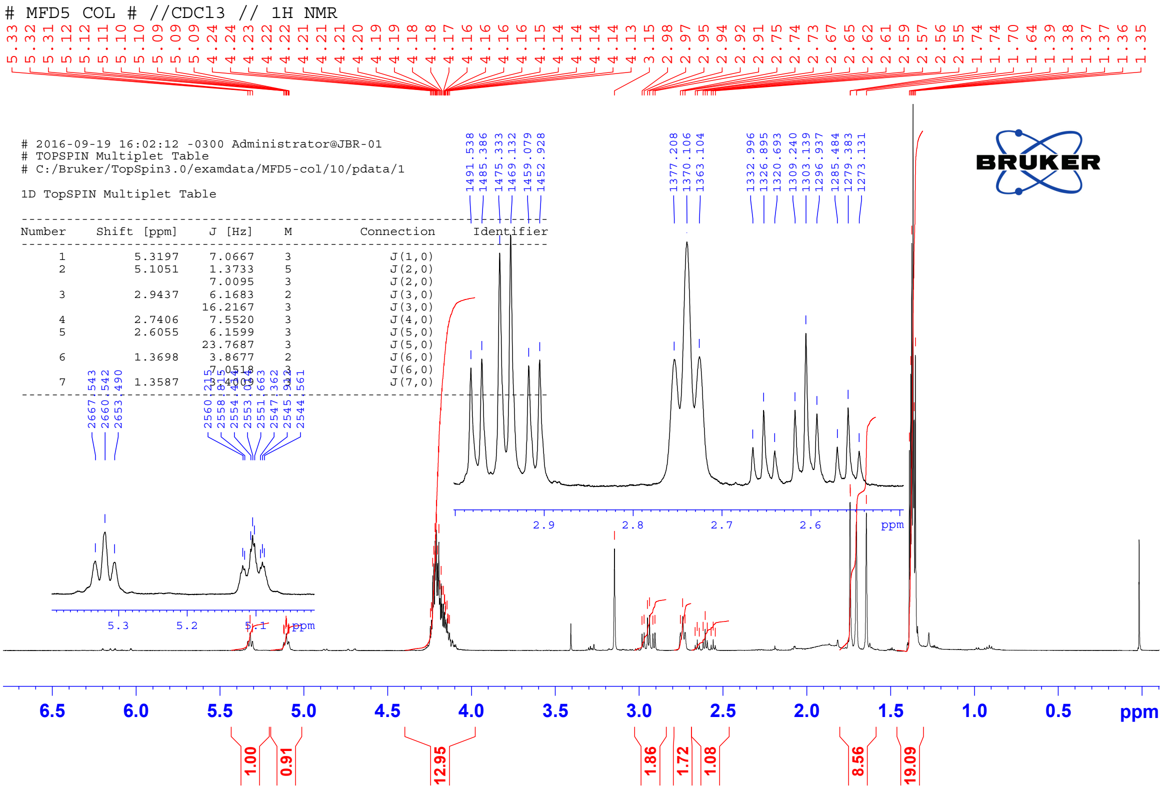

^1H NMR spectrum of compound **38**.

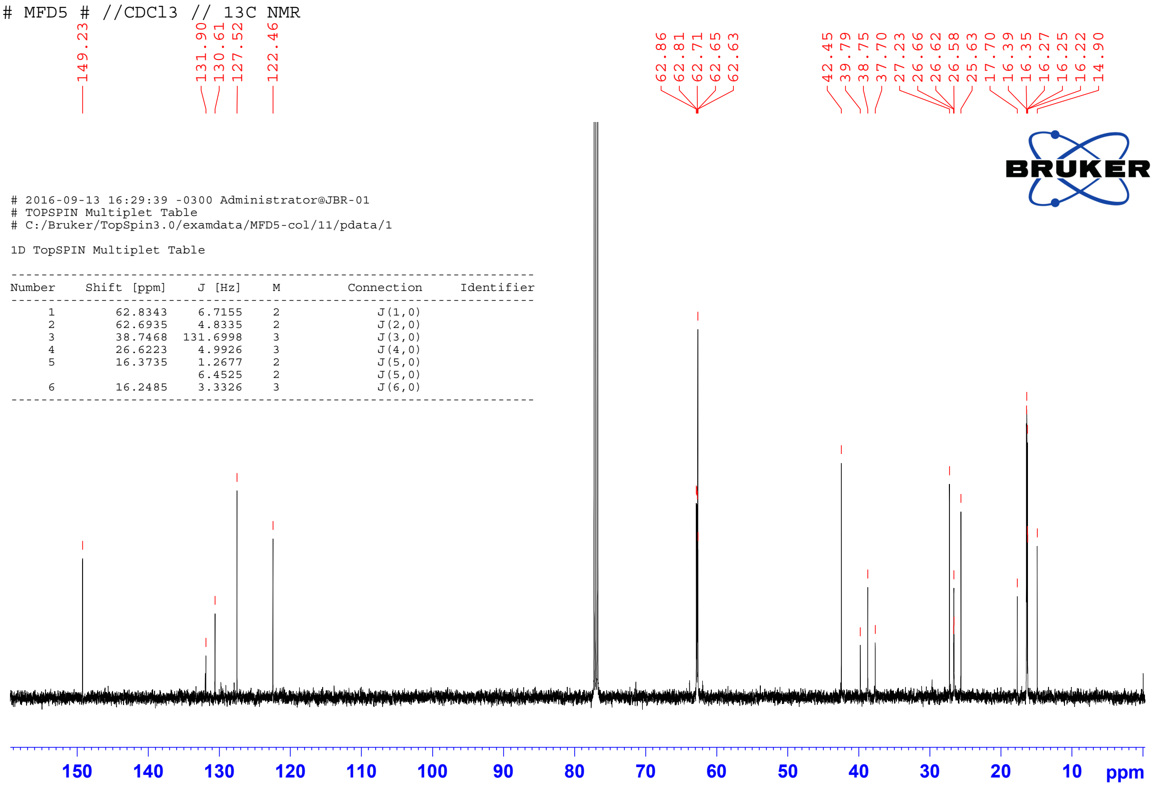

^{13}C NMR spectrum of compound **38**.

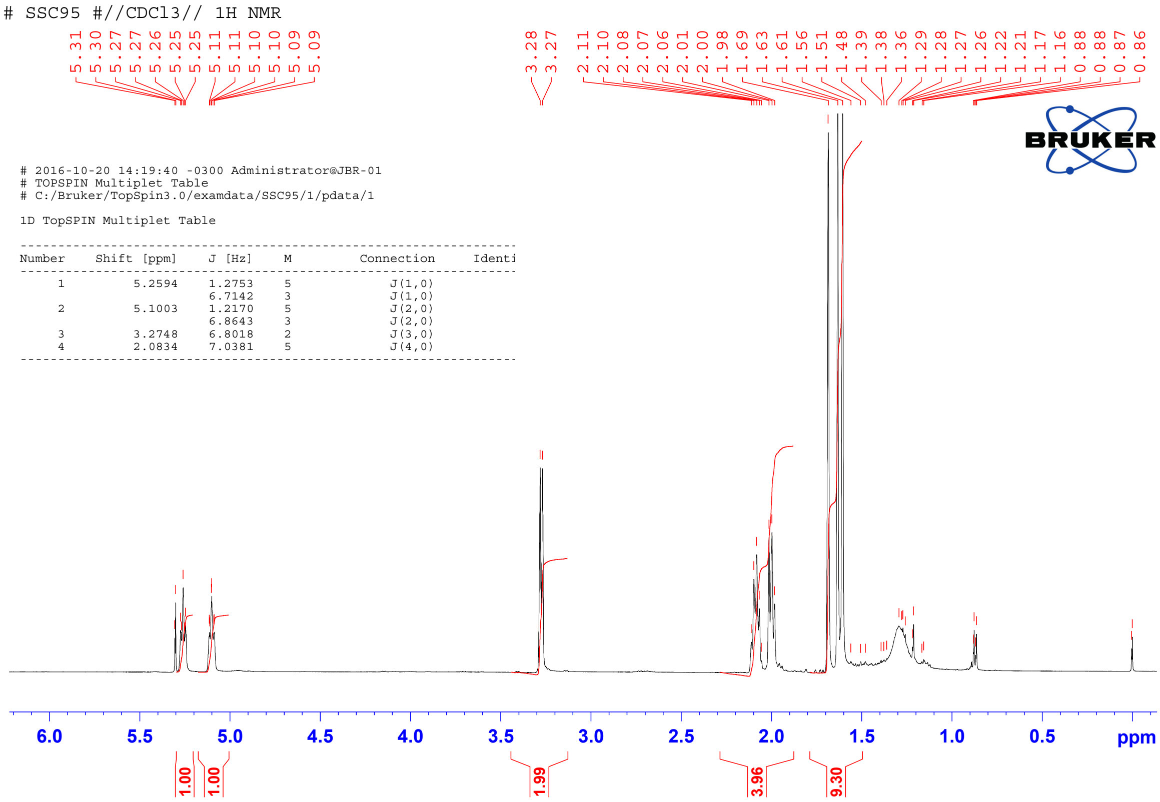

^{1}H NMR spectrum of compound **42**.

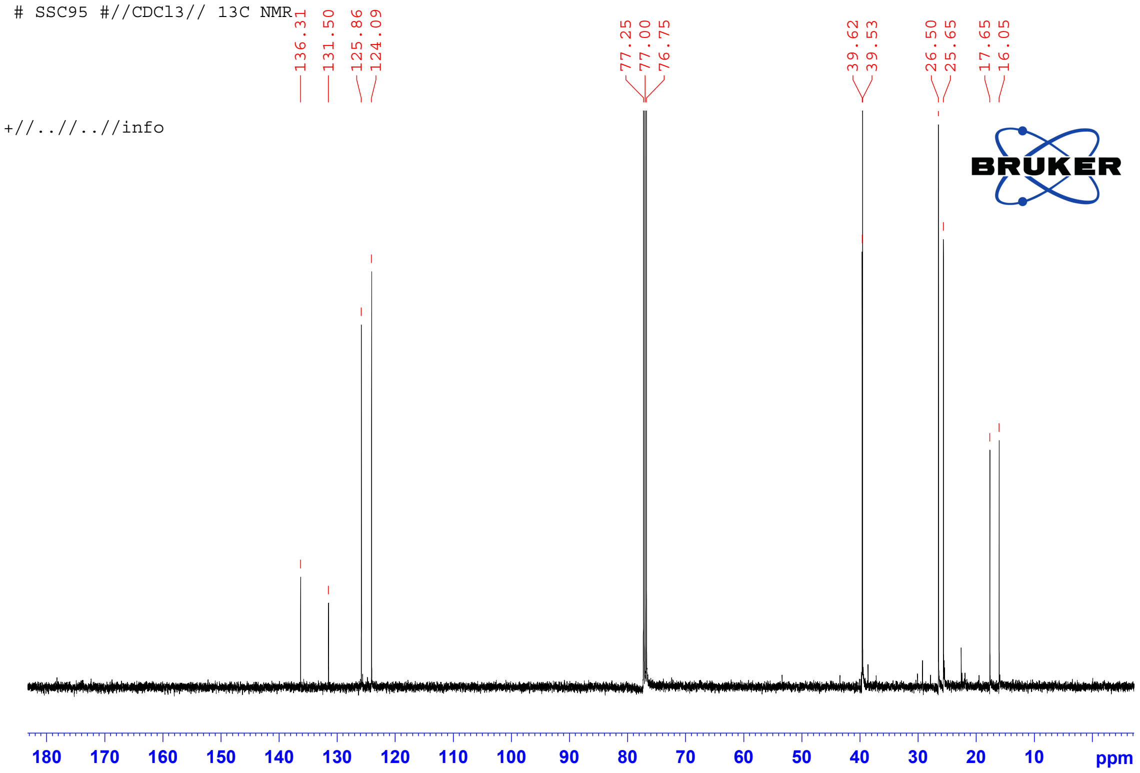

^{13}C NMR spectrum of compound **42**.

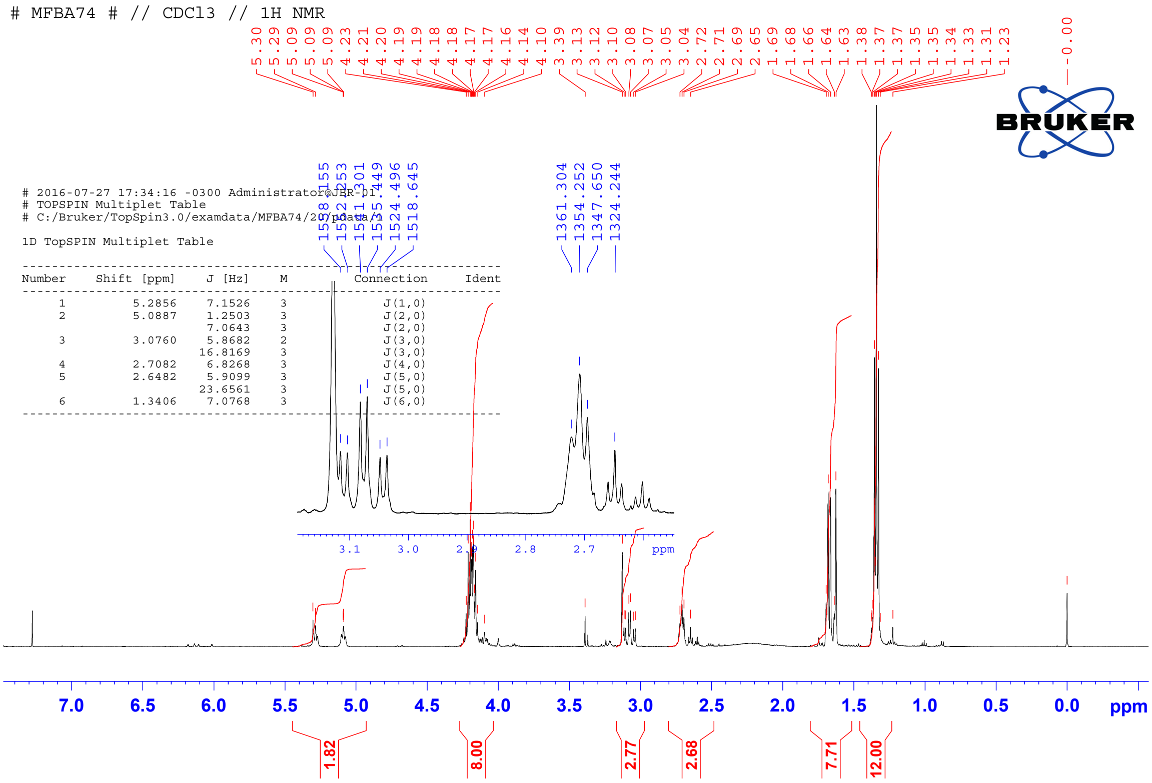

^{1}H NMR spectrum of compound **43**.

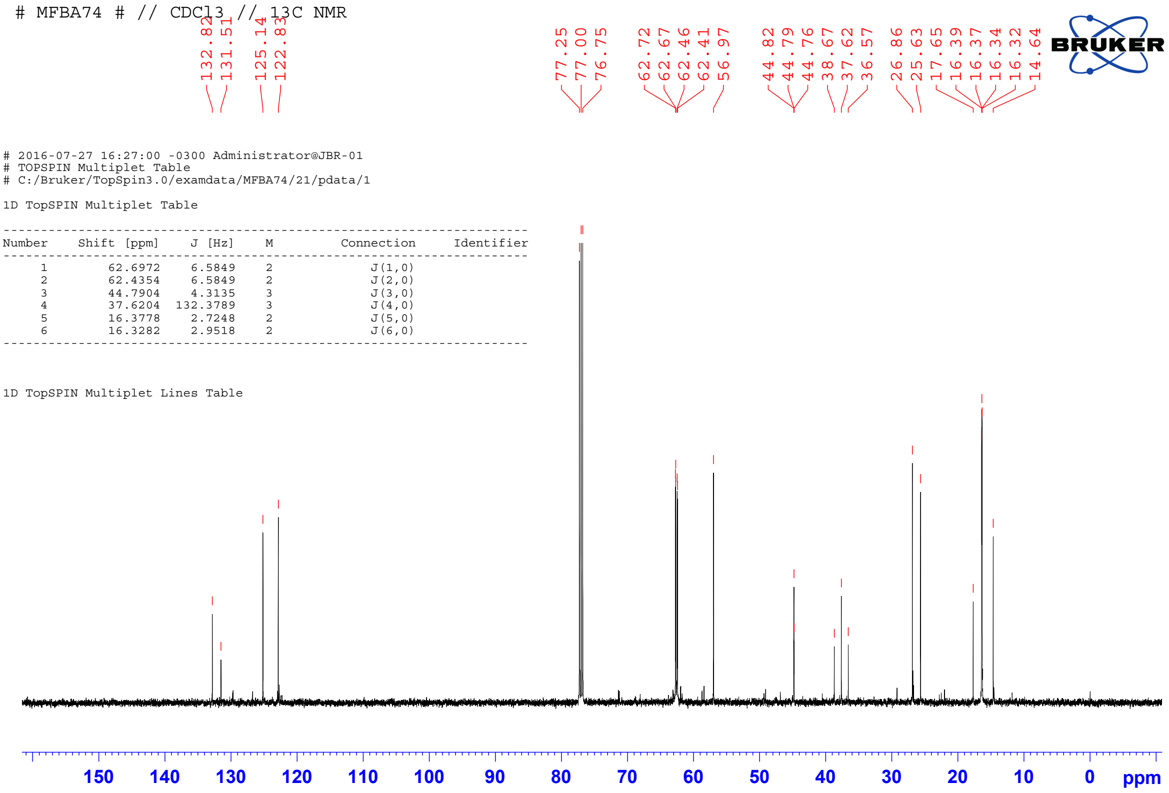

^{13}C NMR spectrum of compound **43**.

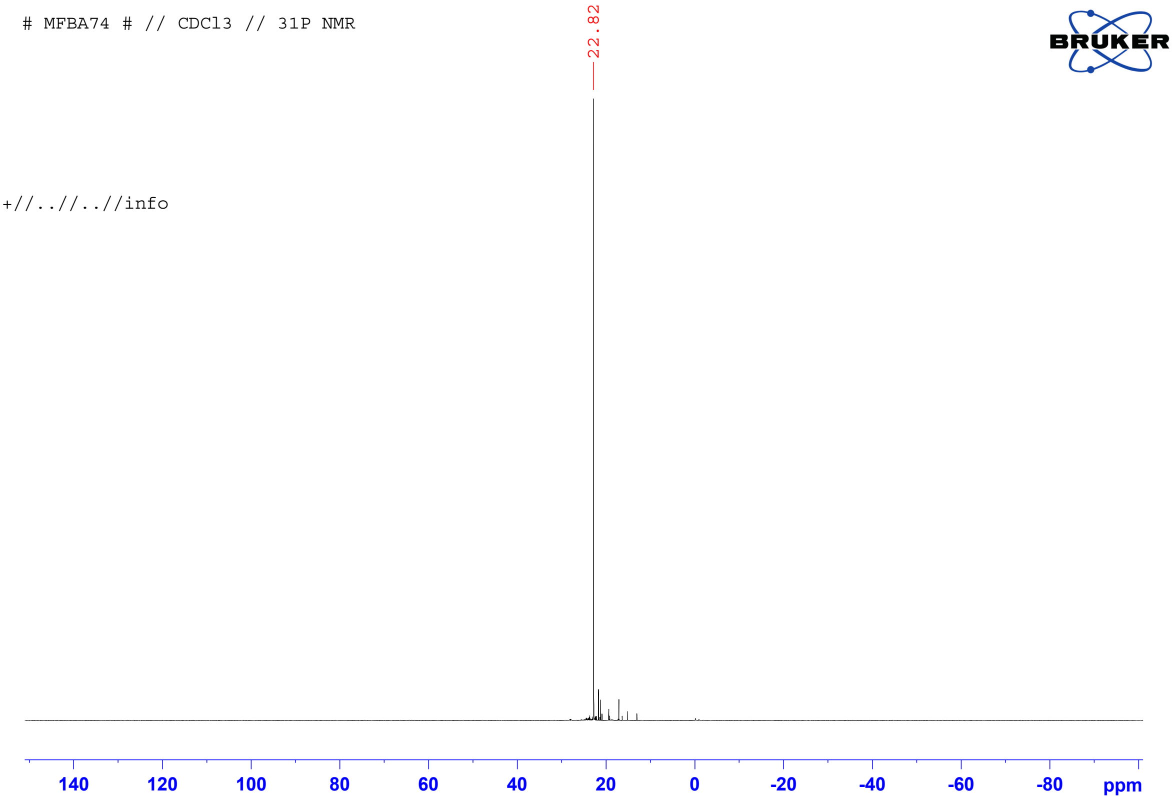

^{31}P NMR spectrum of compound **43**.

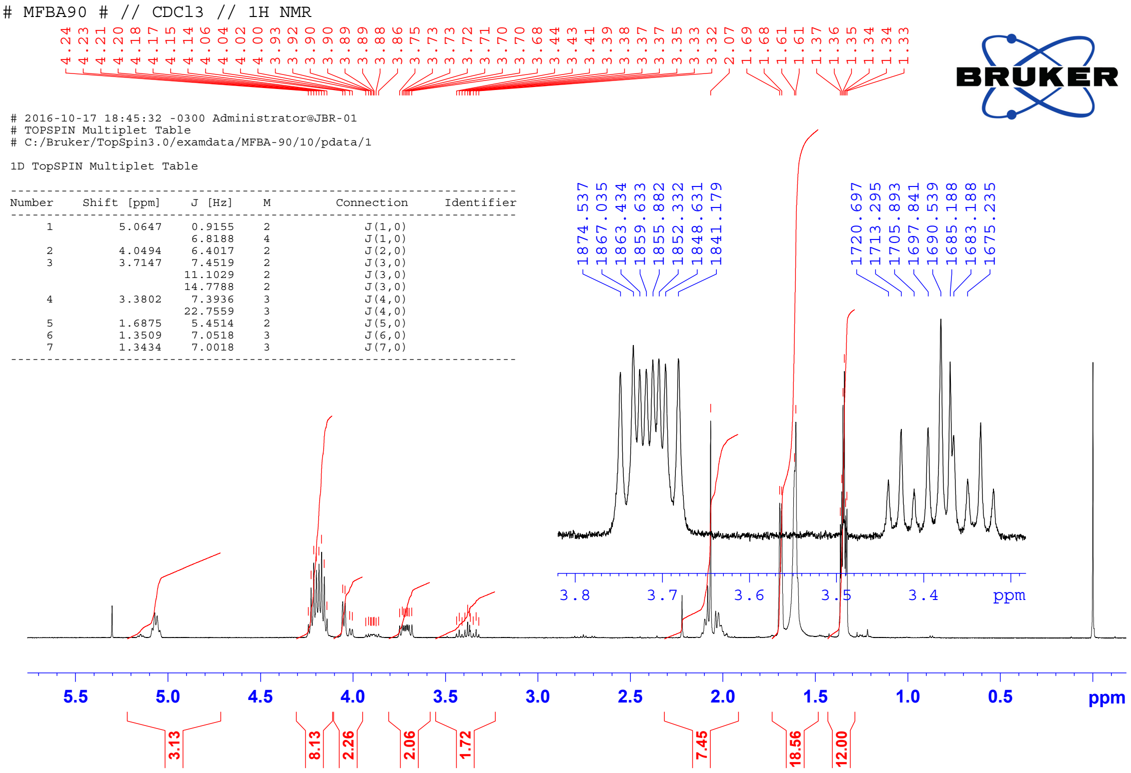

Number	Shift [ppm]	J [Hz]	M	Connection	Identifier
1	5.0647	0.9155	2	J(1,0)	
		6.8188	4	J(1,0)	
2	4.0494	6.4017	2	J(2,0)	
3	3.7147	7.4519	2	J(3,0)	
		11.1029	2	J(3,0)	
		14.7788	2	J(3,0)	
4	3.3802	7.3936	3	J(4,0)	
		22.7559	3	J(4,0)	
5	1.6875	5.4514	2	J(5,0)	
6	1.3509	7.0518	3	J(6,0)	
7	1.3434	7.0018	3	J(7,0)	

^{1}H NMR spectrum of compound **46**.

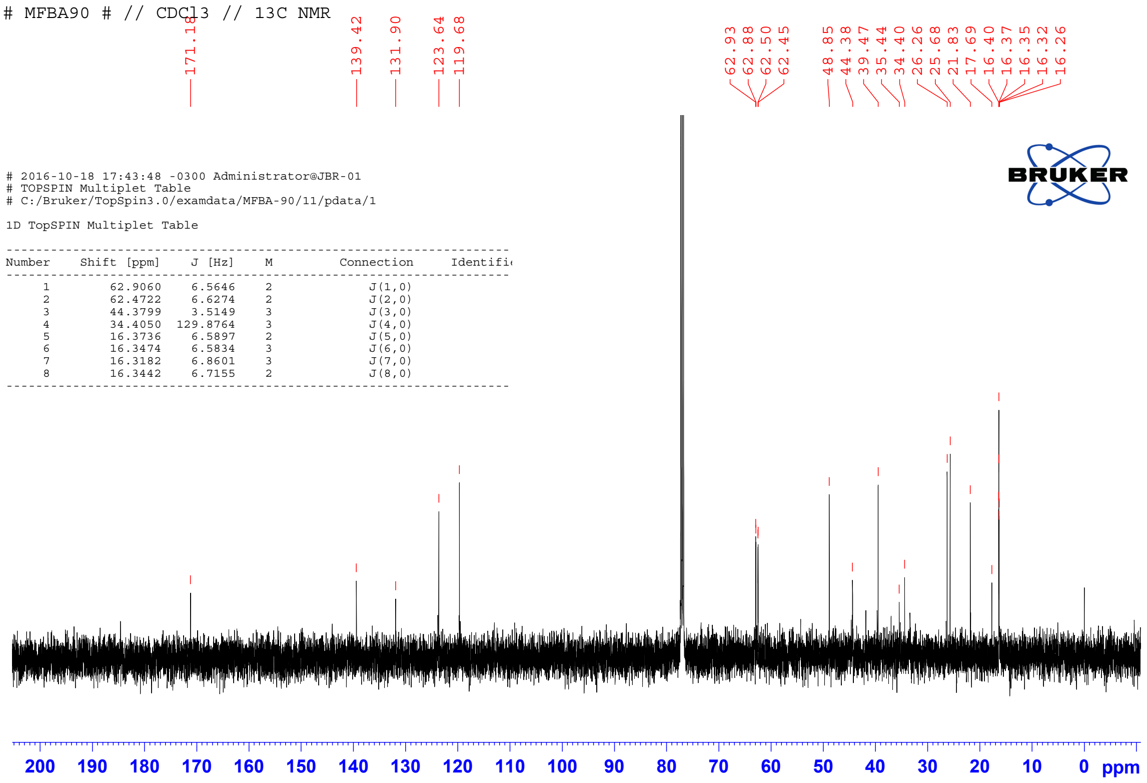

^{13}C NMR spectrum of compound **46**.

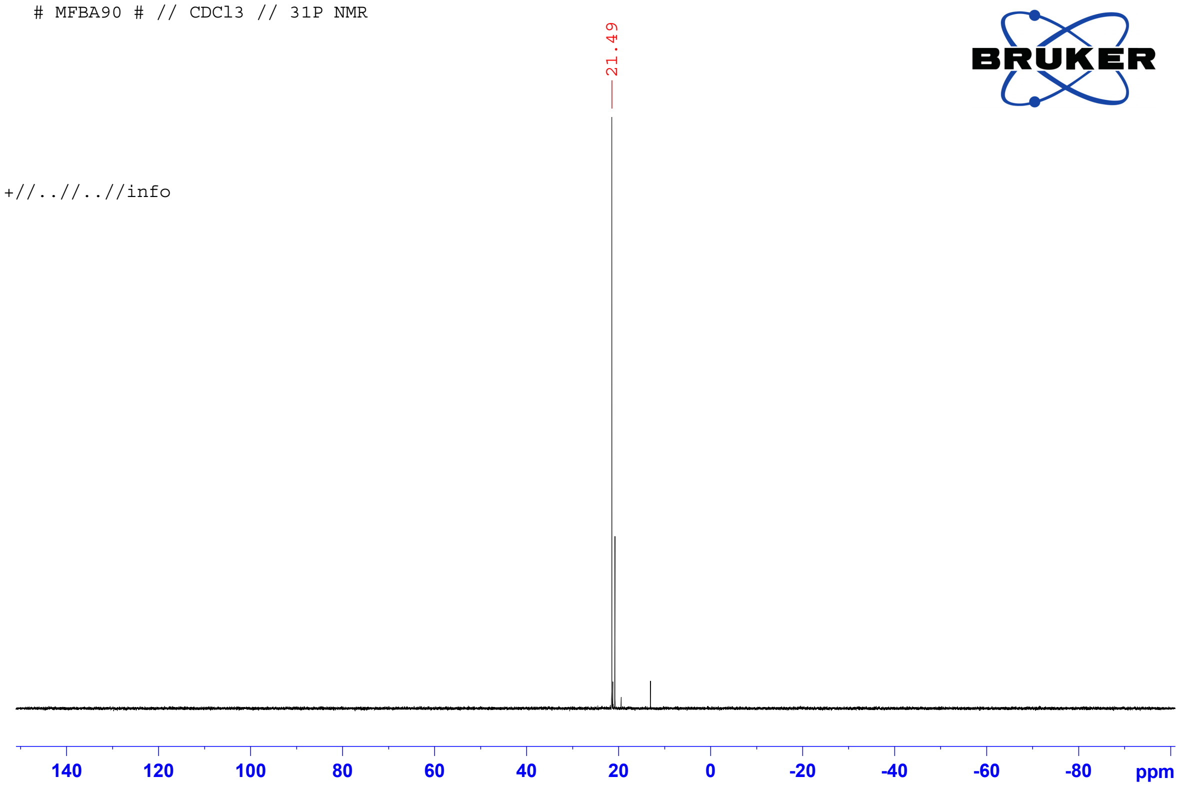

^{31}P NMR spectrum of compound **46**.

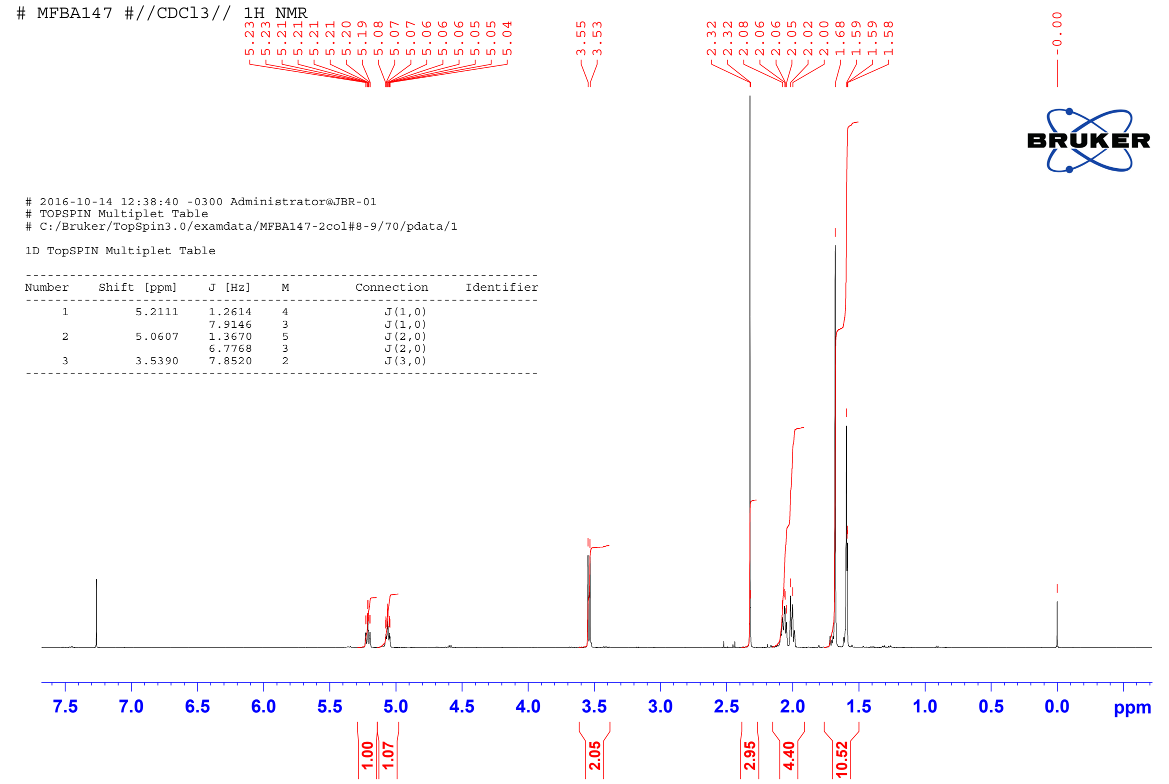

^{1}H NMR spectrum of compound **48**.

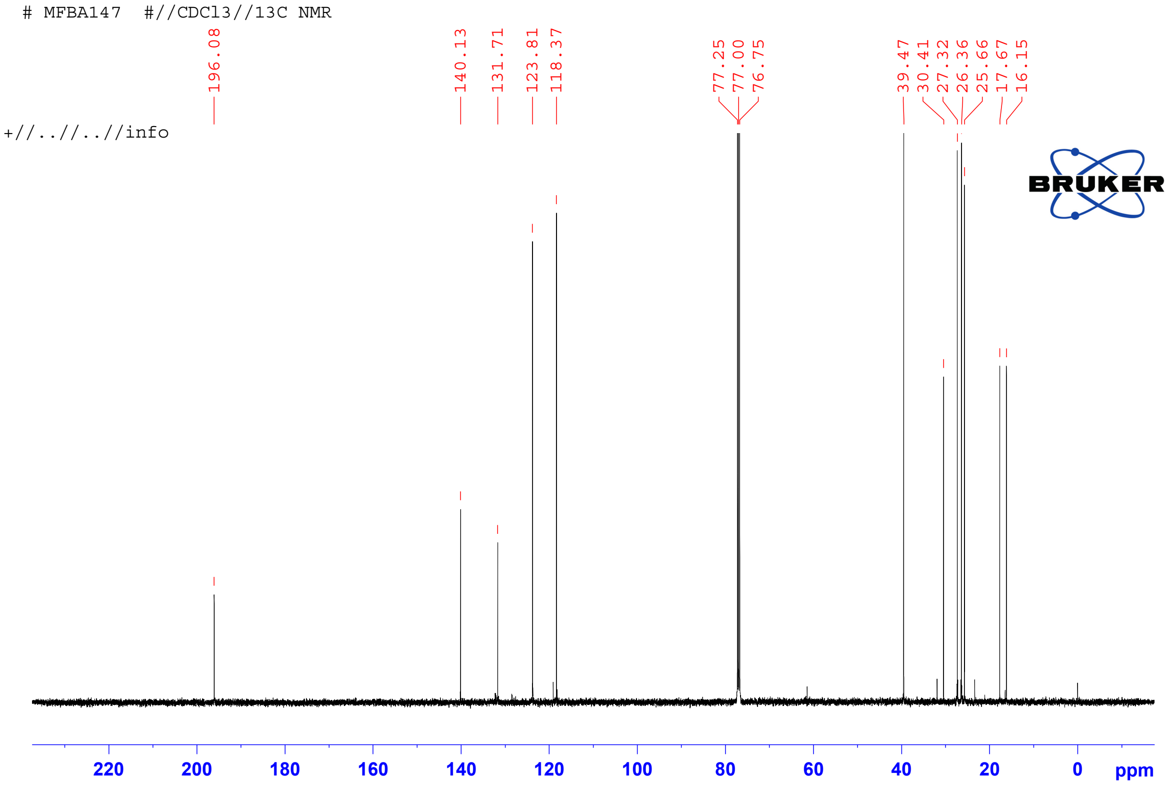

^{13}C NMR spectrum of compound **48**.

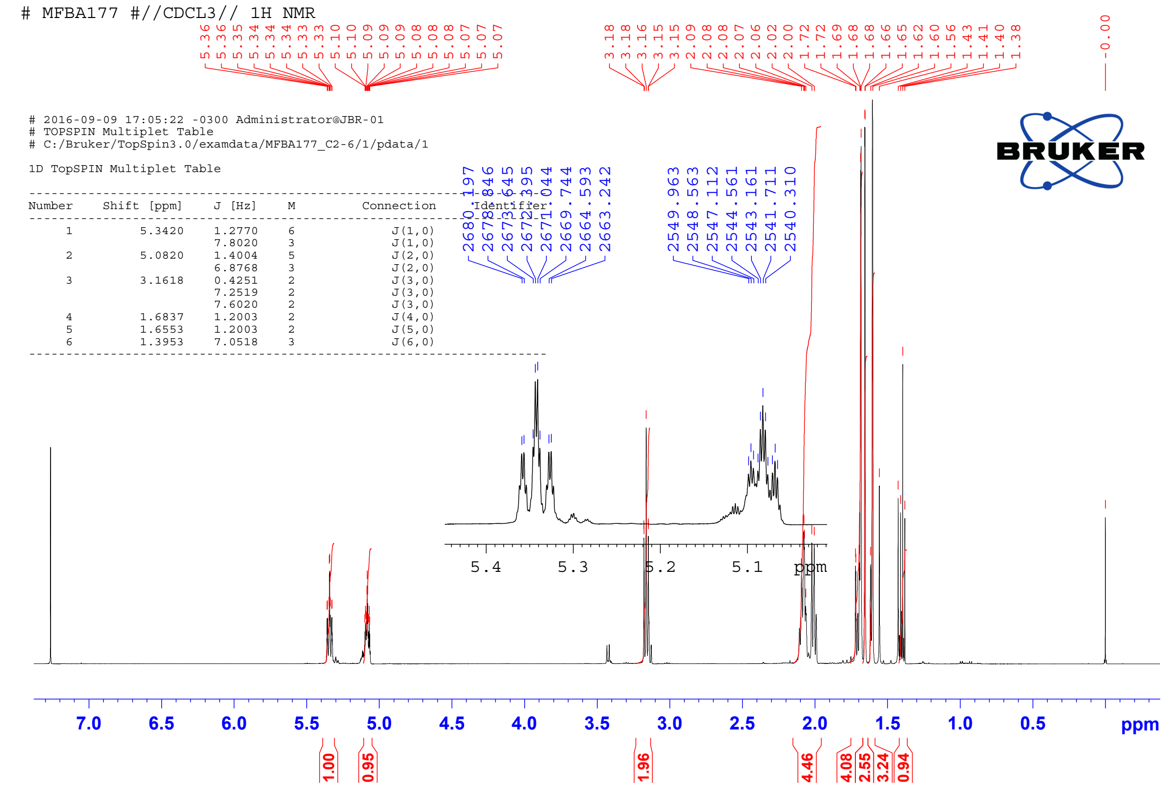

^{1}H NMR spectrum of compound **50**.

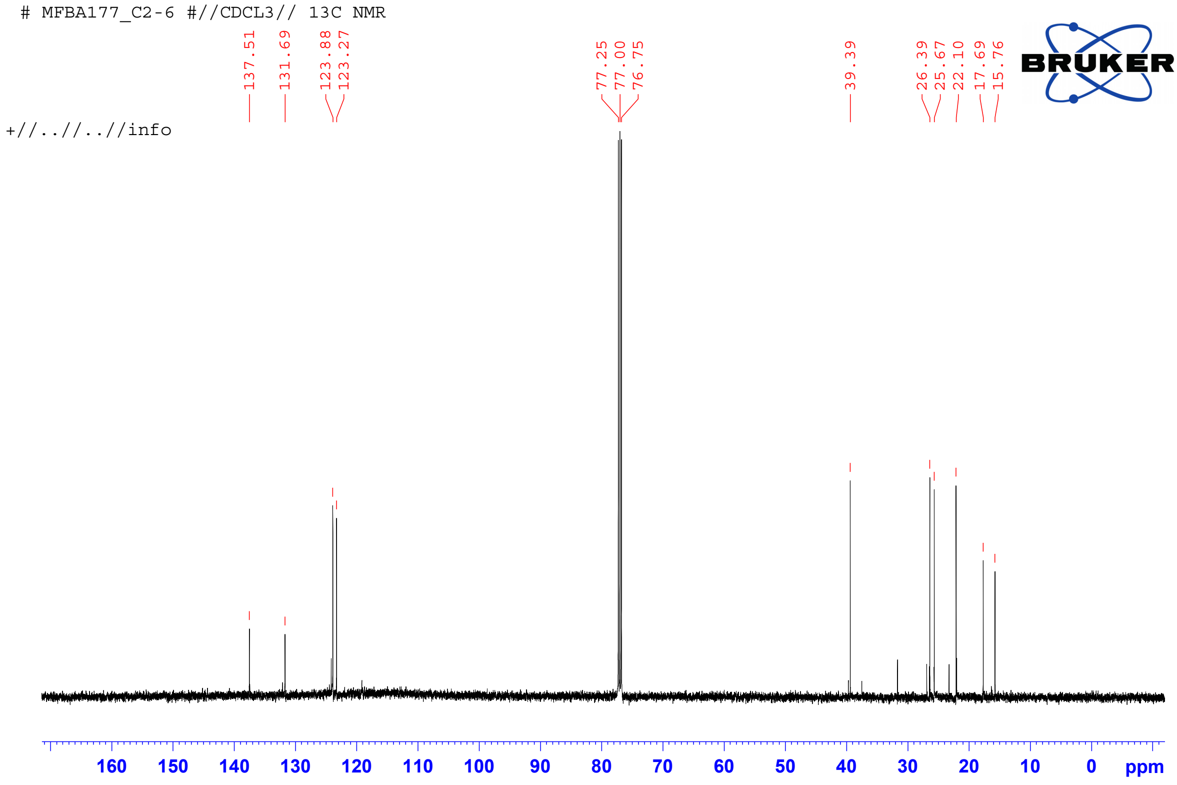

^{13}C NMR spectrum of compound **50**.

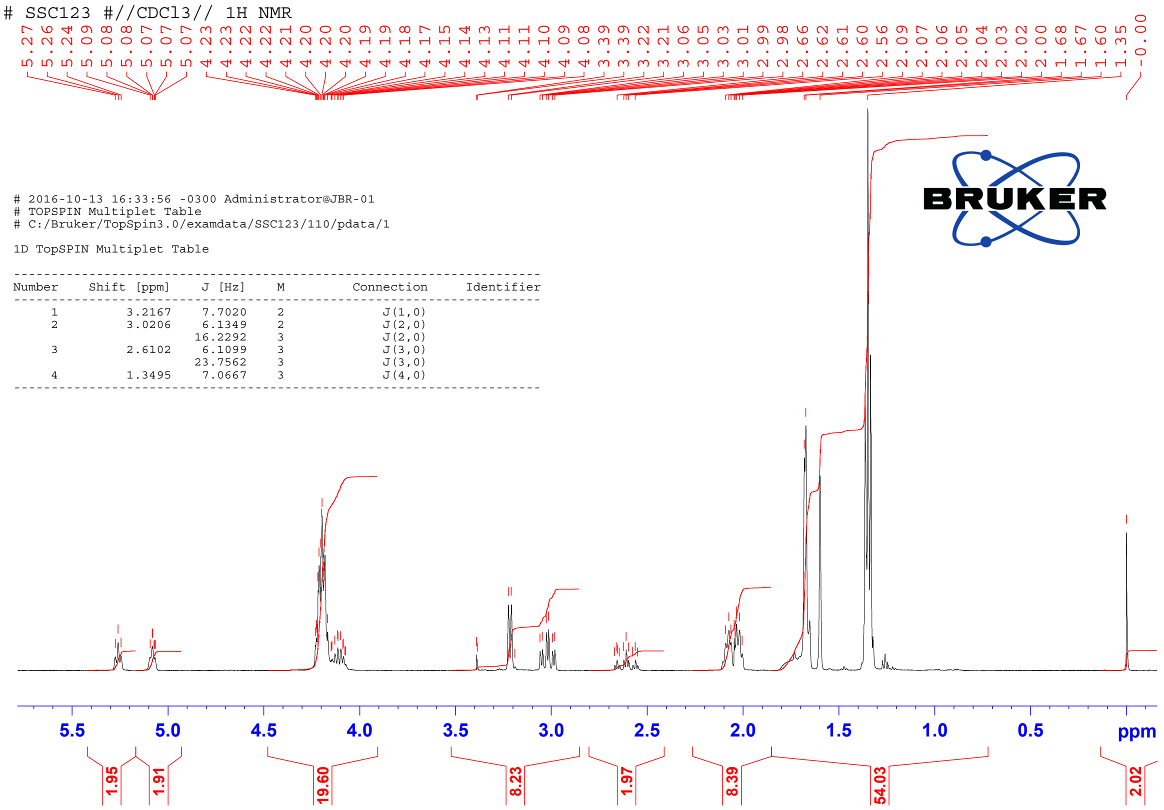

^{1}H NMR spectrum of compound **52**.

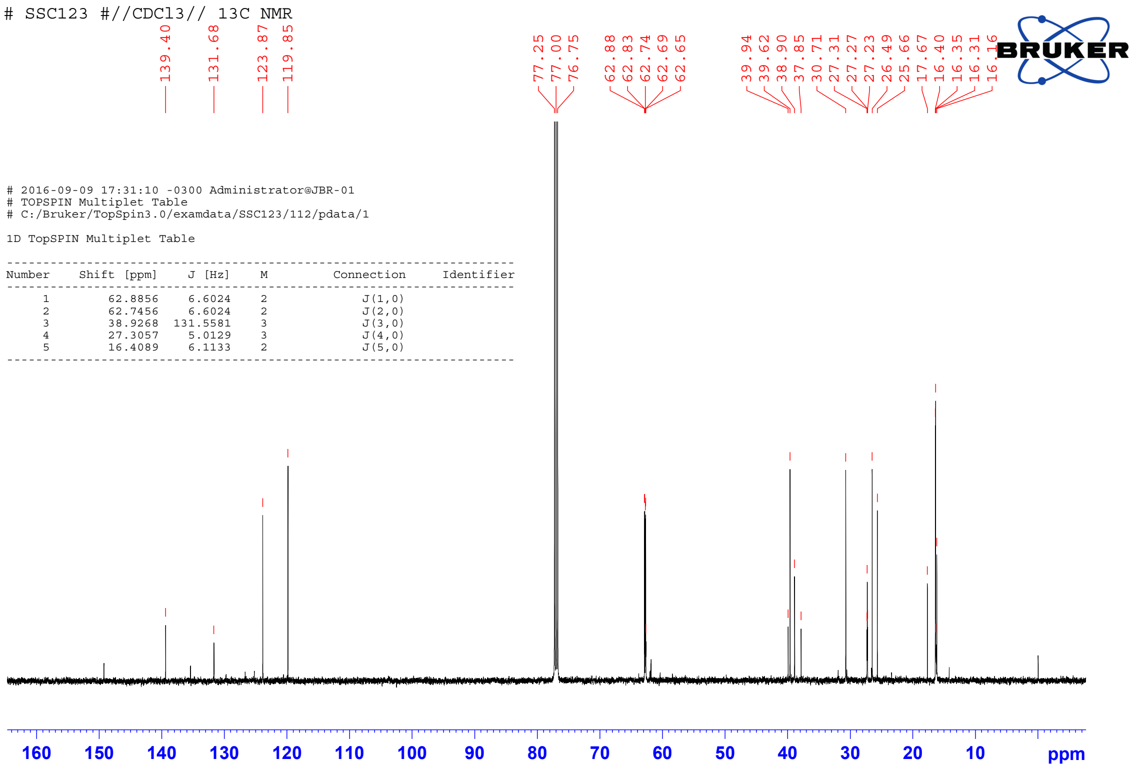

^{13}C NMR spectrum of compound **52**.

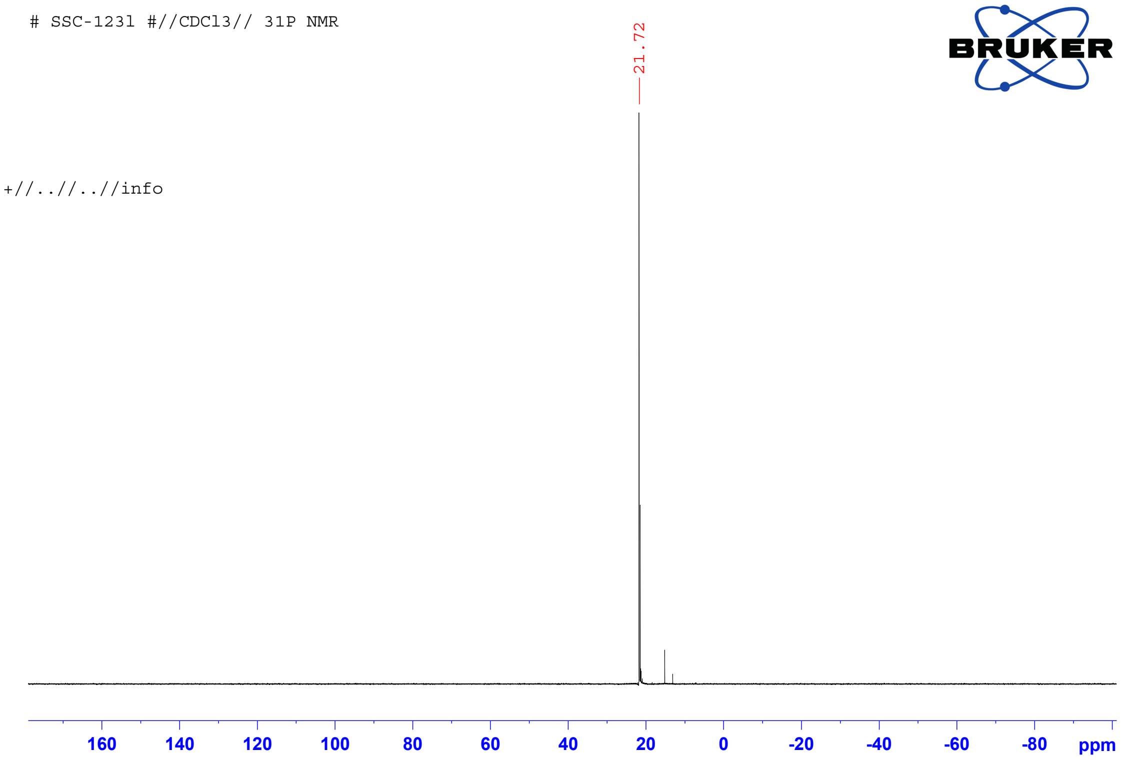

^{31}P NMR spectrum of compound **52**.

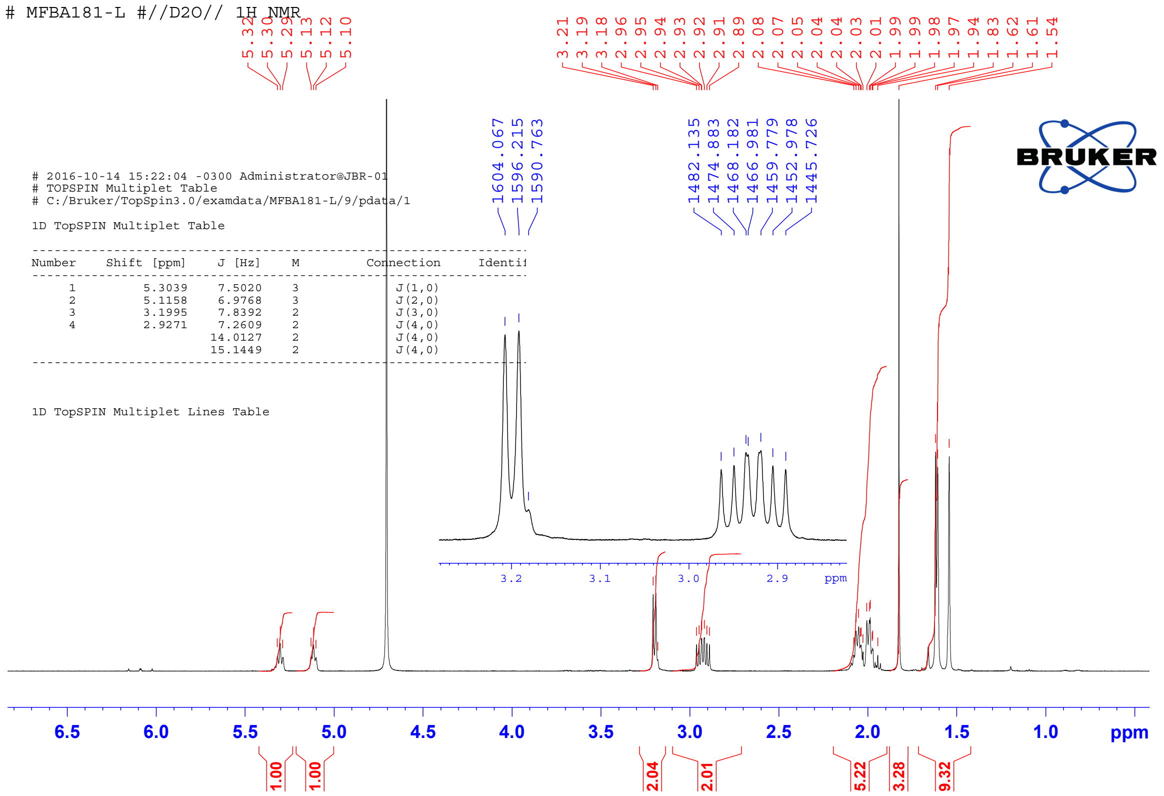

^{1}H NMR spectrum of compound **44**.

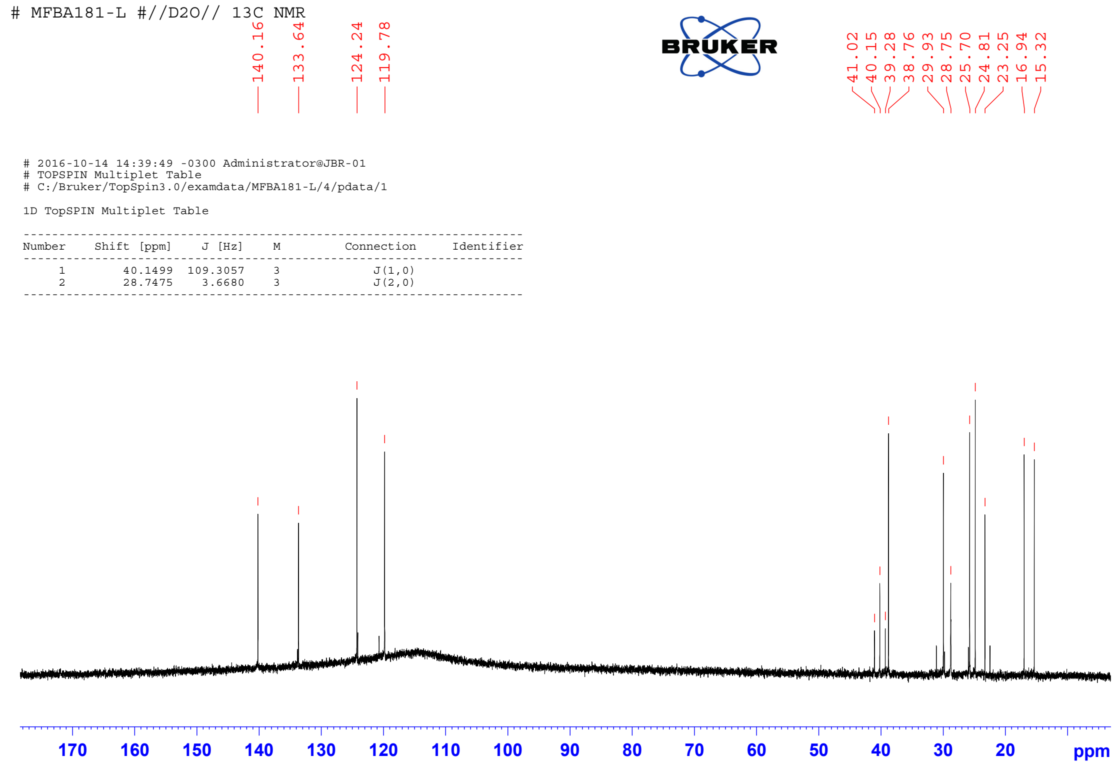

^{13}C NMR spectrum of compound **44**.

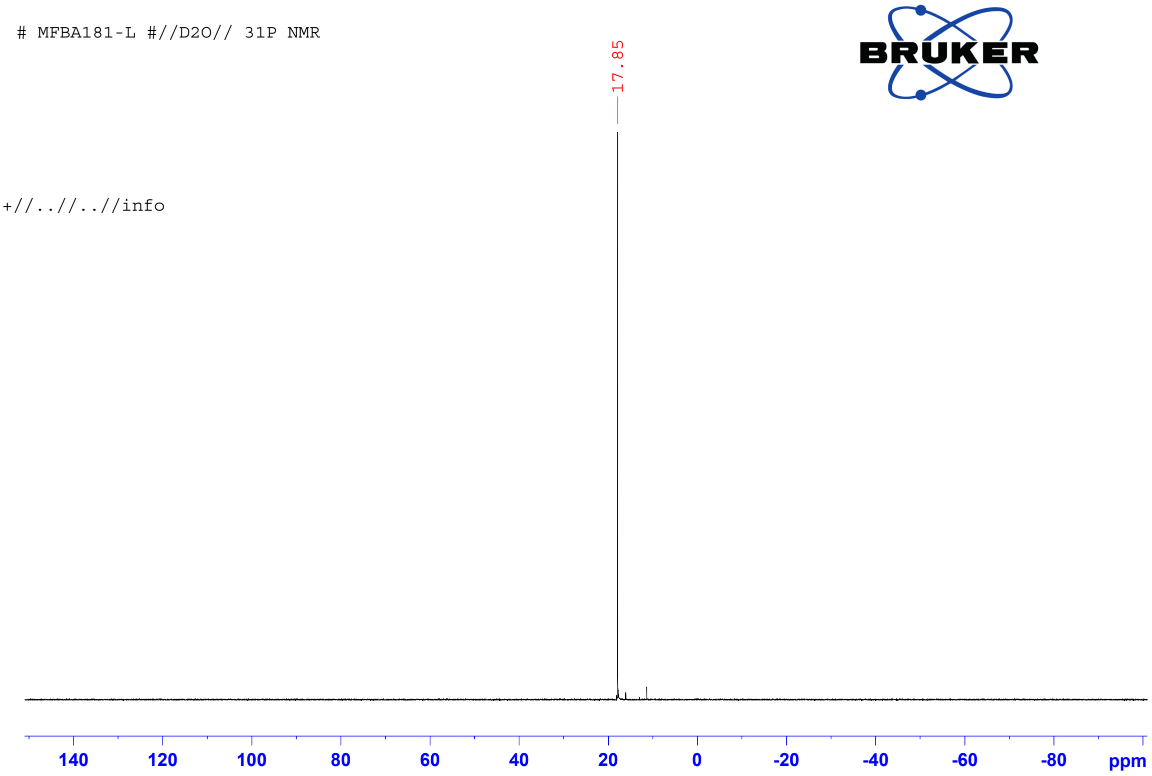

^{31}P NMR spectrum of compound **44**.

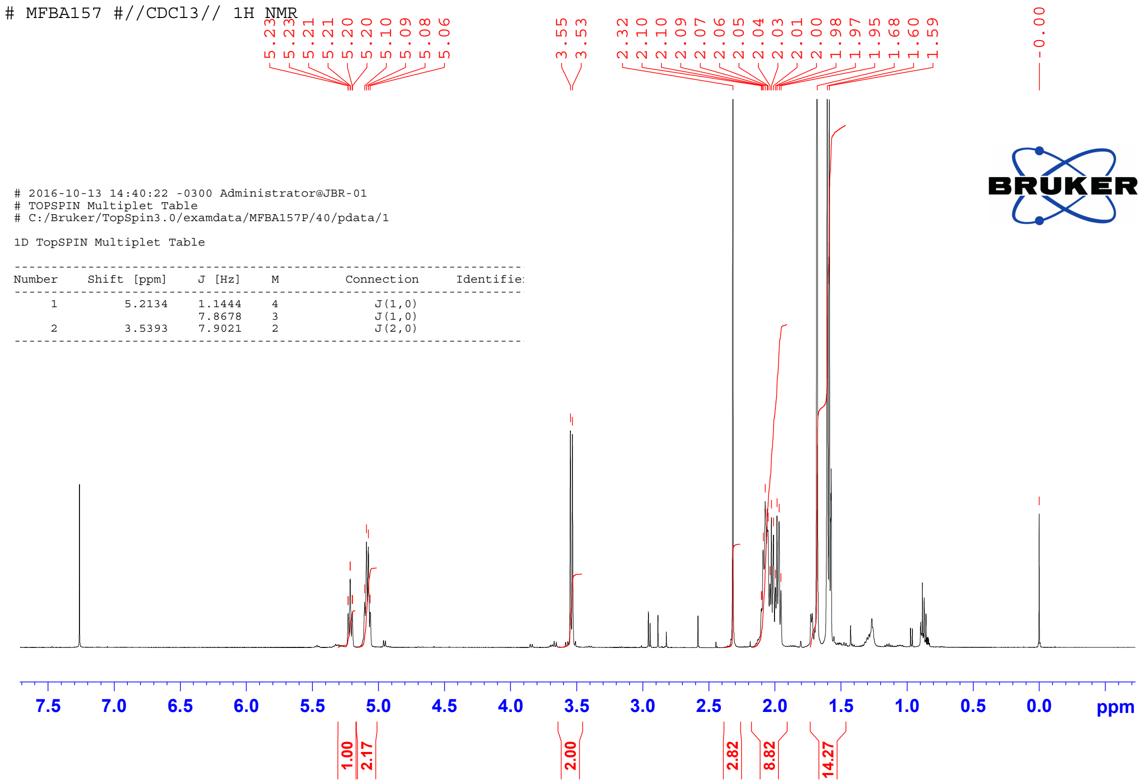

^{1}H NMR spectrum of compound **49**.

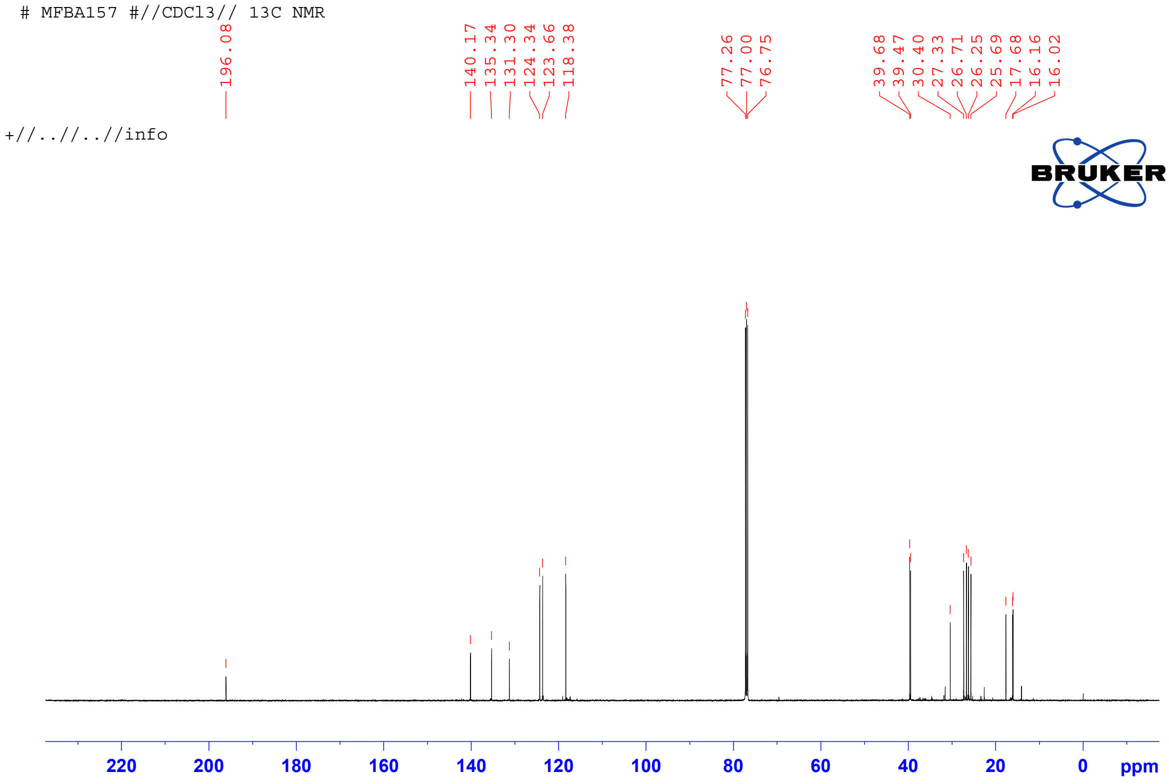

^{13}C NMR spectrum of compound **49**.

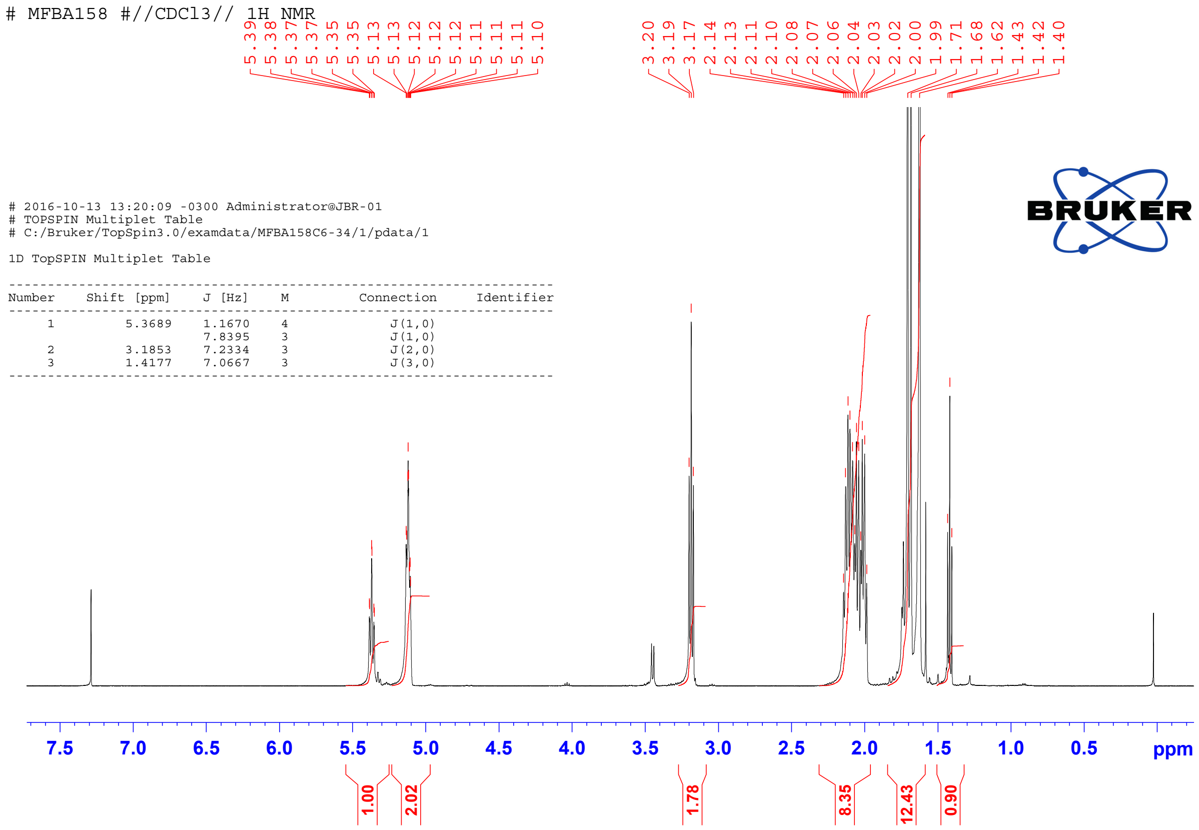

^{1}H NMR spectrum of compound **51**.

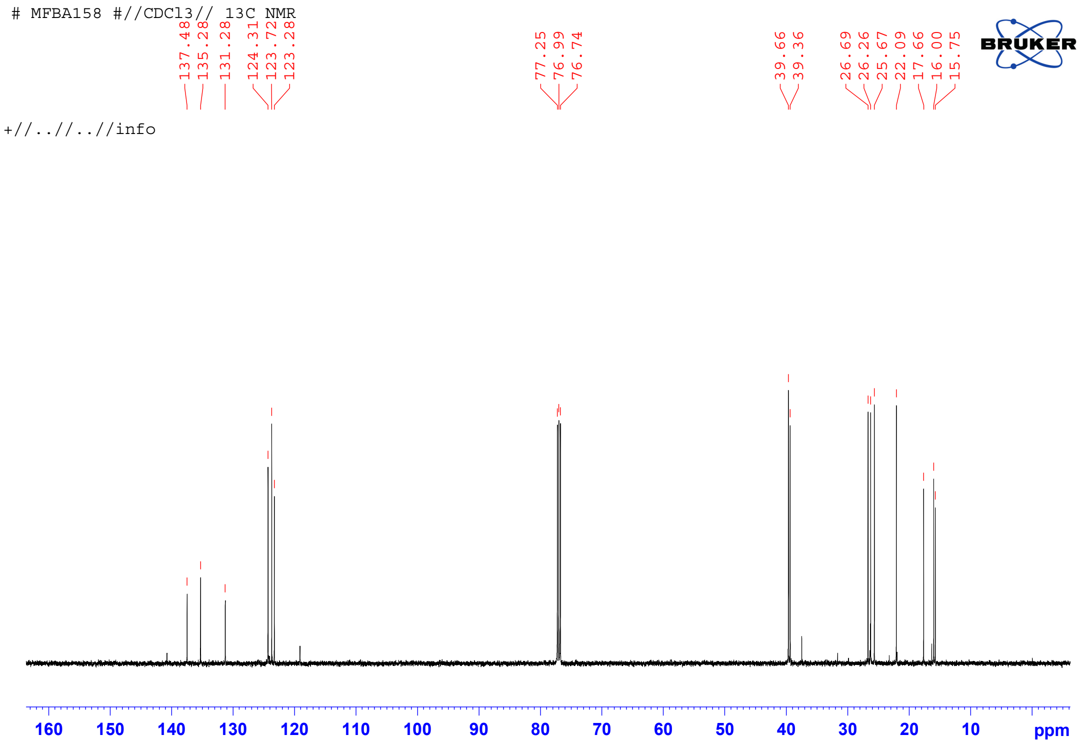

^{13}C NMR spectrum of compound **51**.

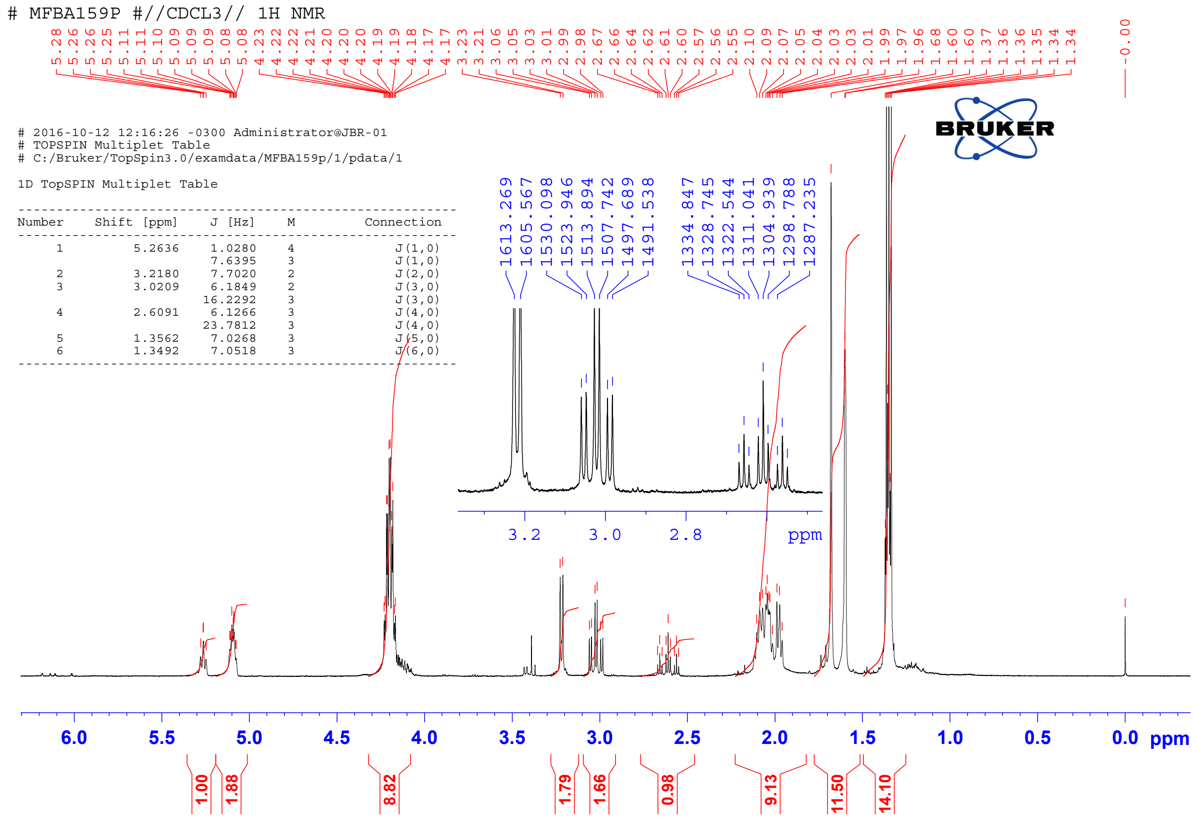

^{1}H NMR spectrum of compound **53**.

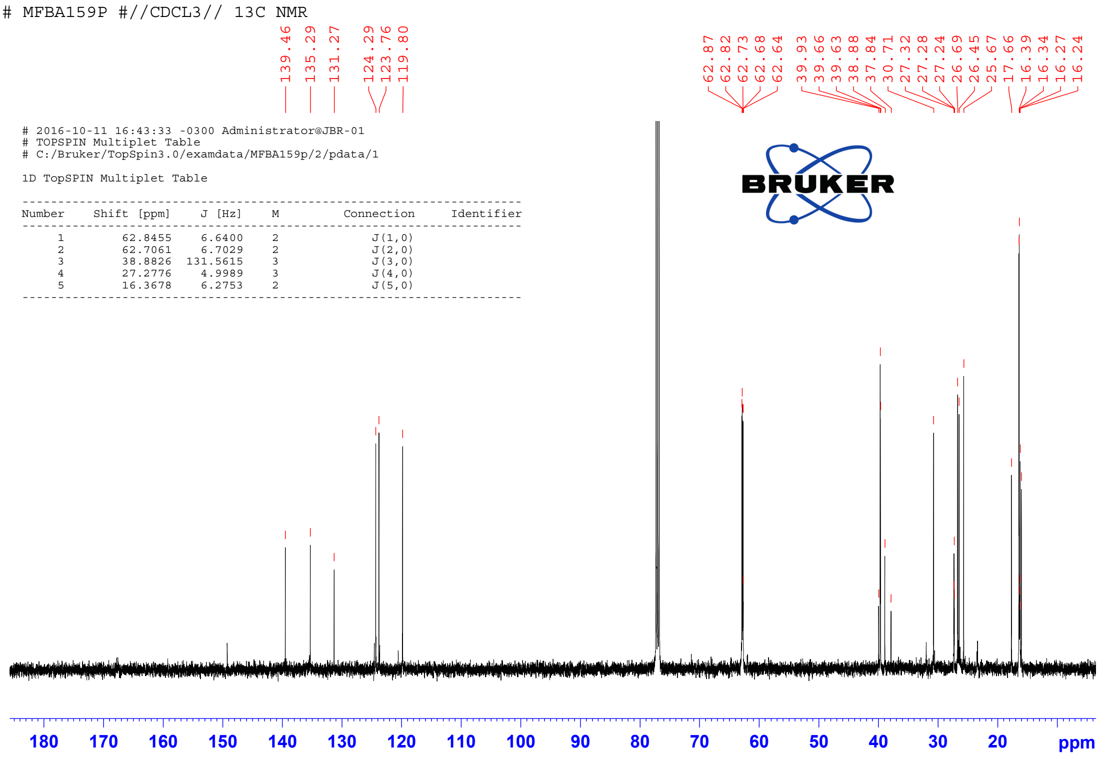

^{13}C NMR spectrum of compound **53**.

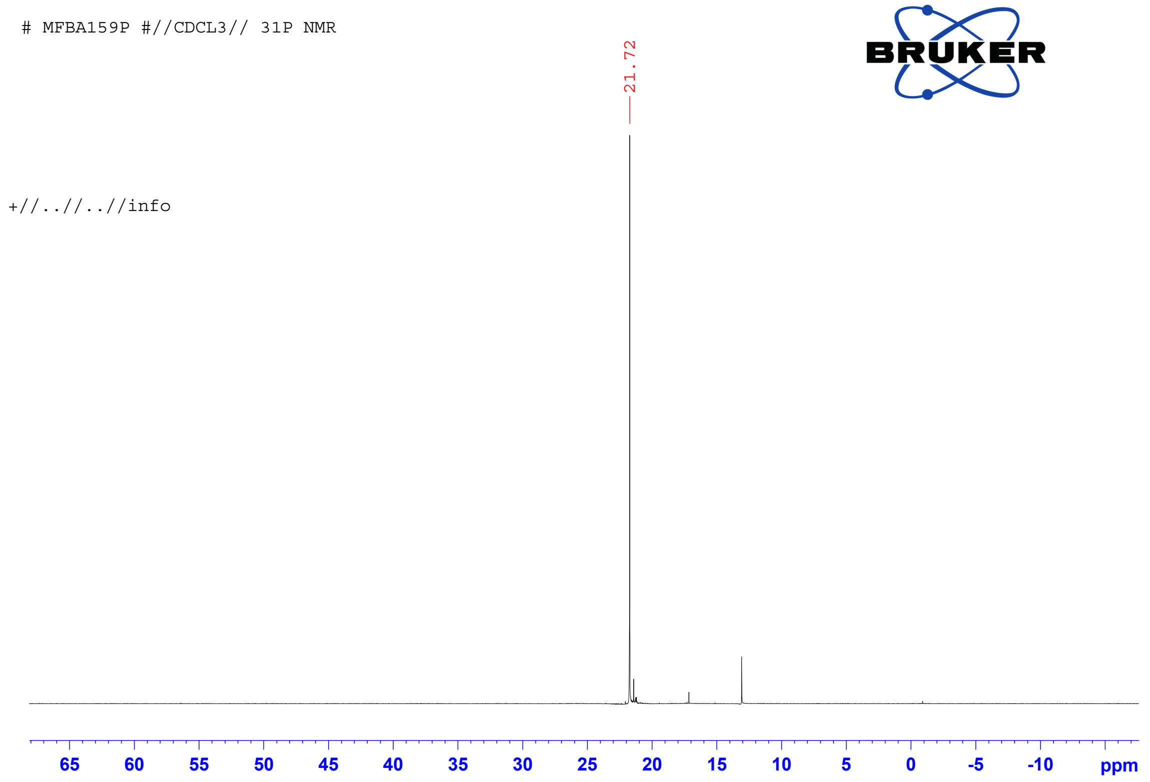

^{31}P NMR spectrum of compound **53**.

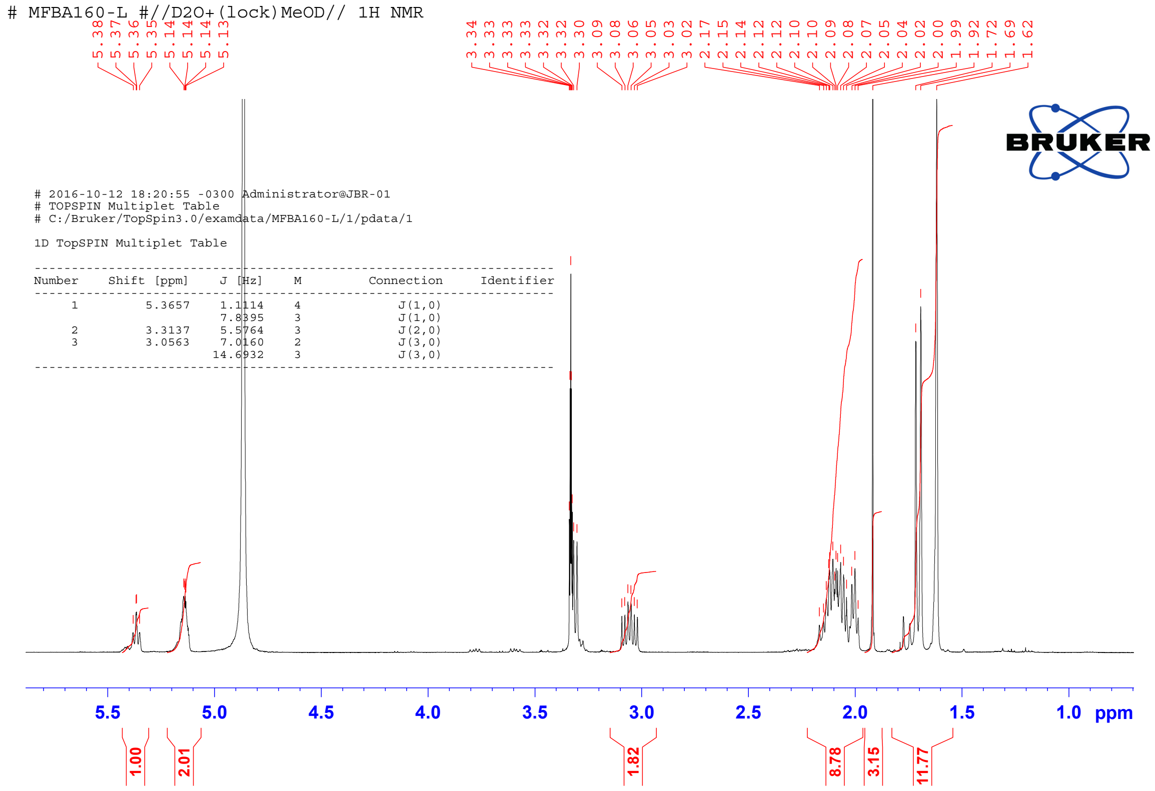

^{1}H NMR spectrum of compound **45**.

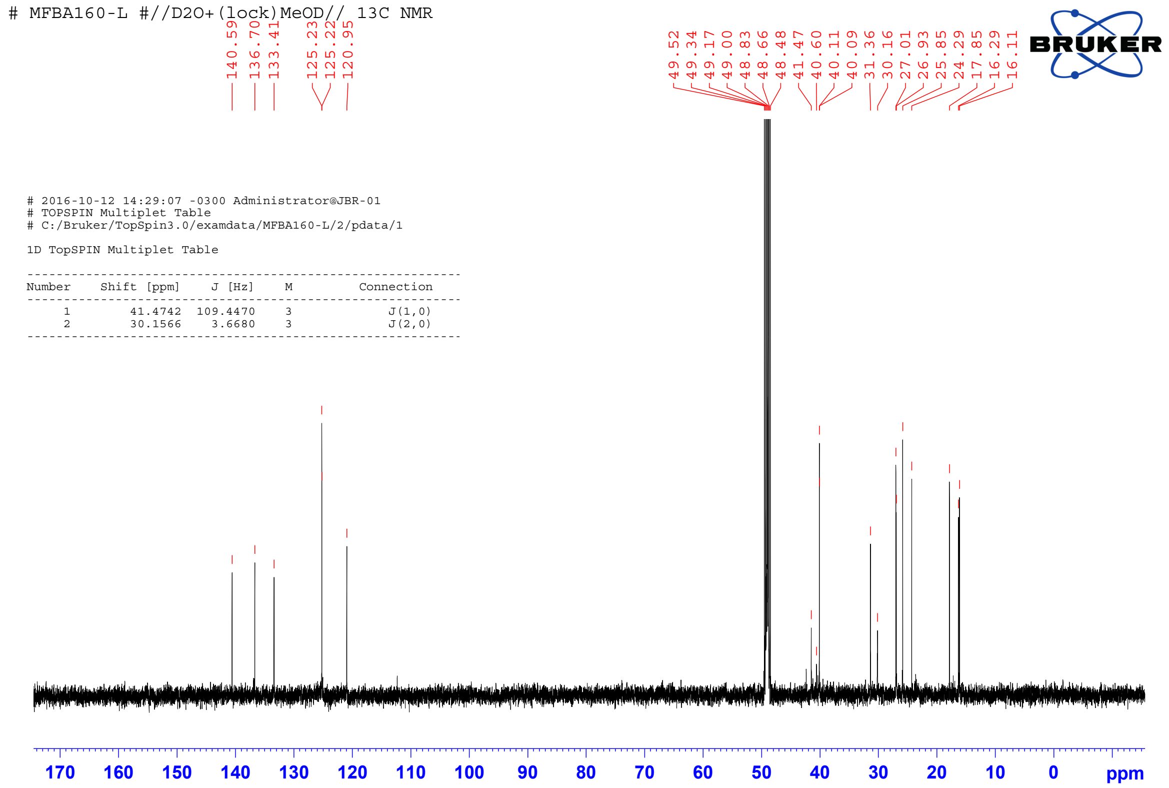

^{13}C NMR spectrum of compound **45**.

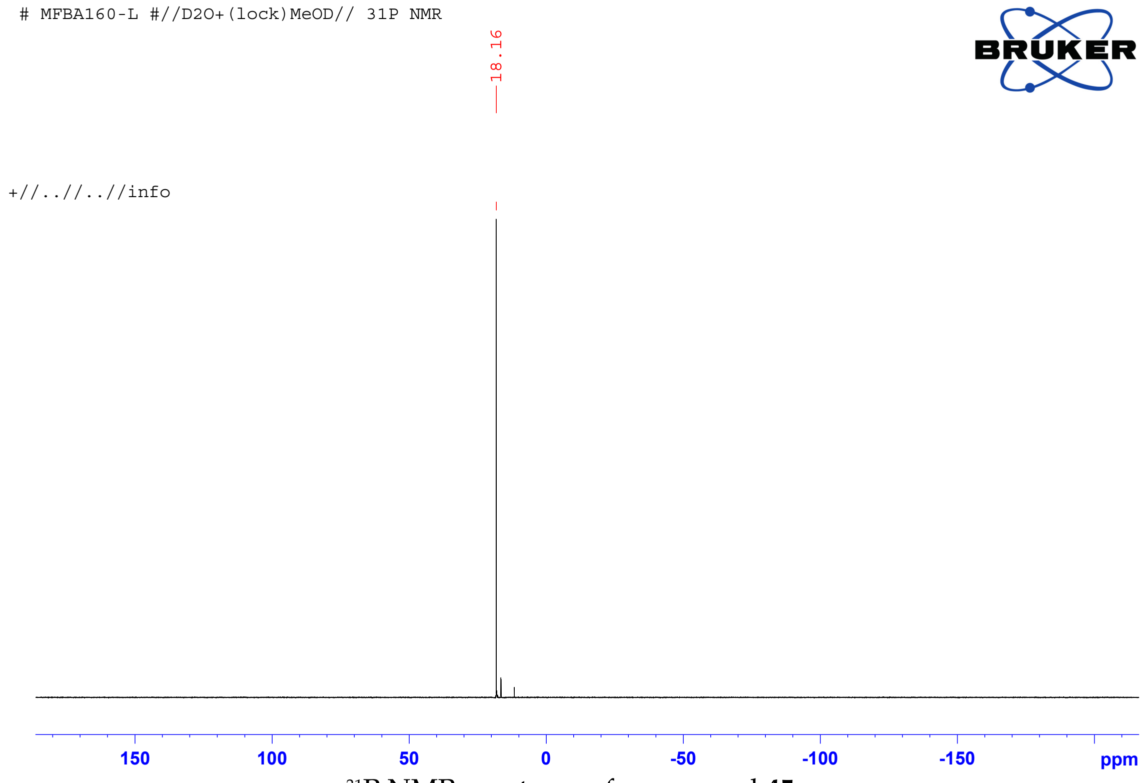

^{31}P NMR spectrum of compound **45**.

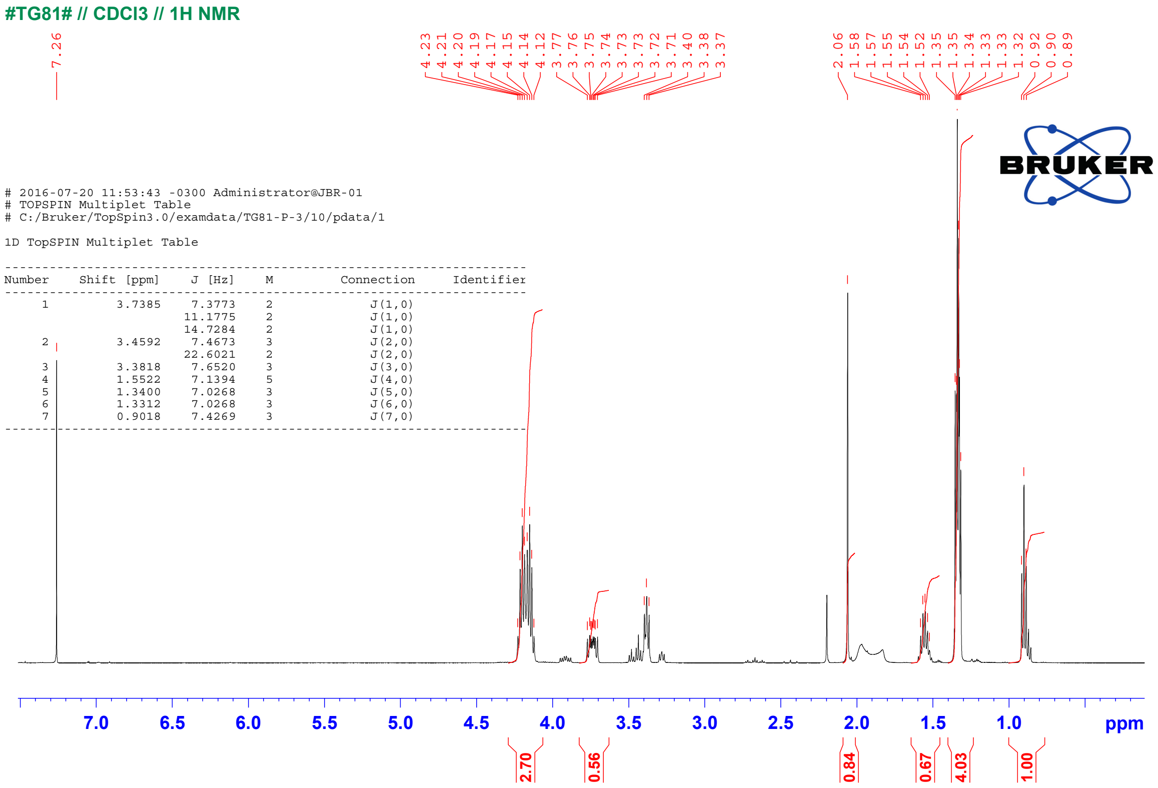

^{1}H NMR spectrum of compound **67**.

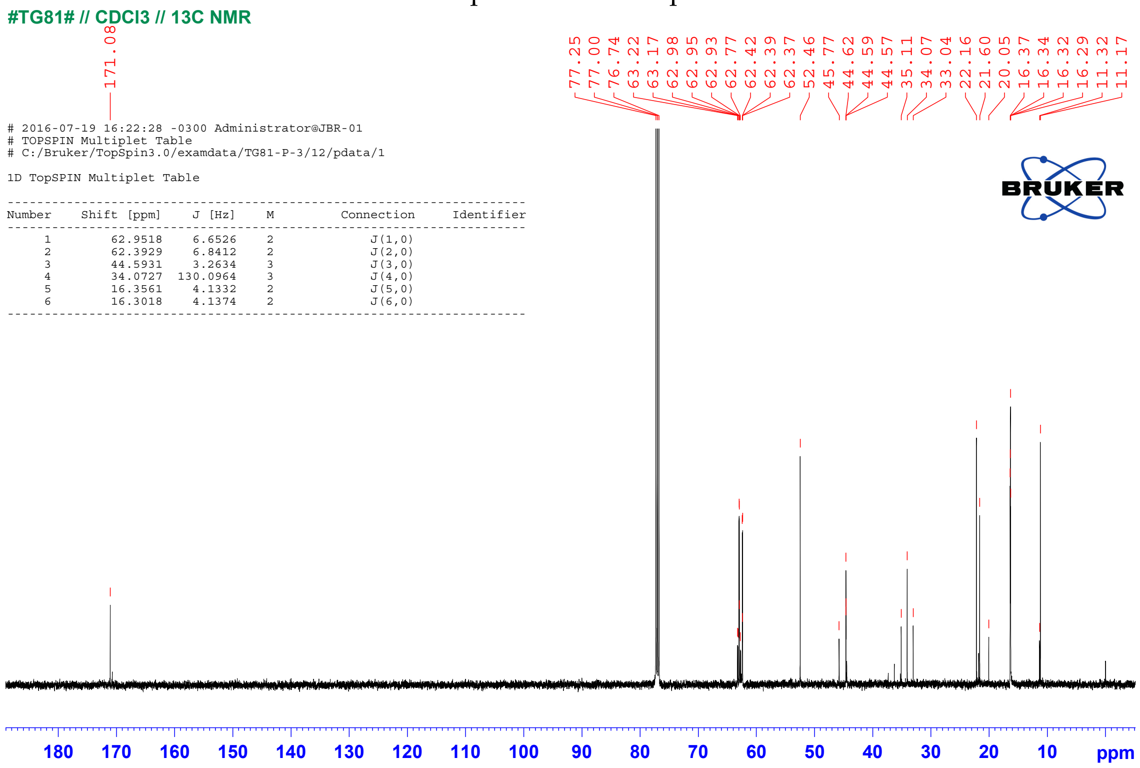

^{13}C NMR spectrum of compound **67**.

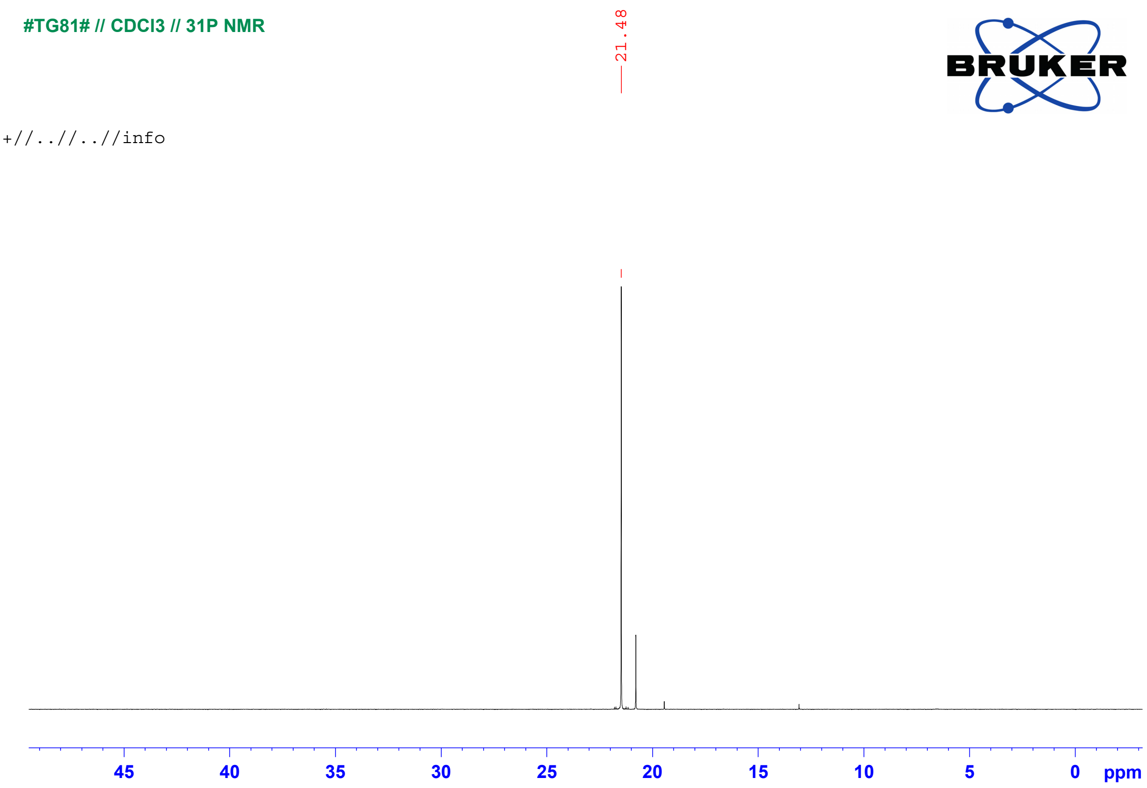

^{31}P NMR spectrum of compound **67**.

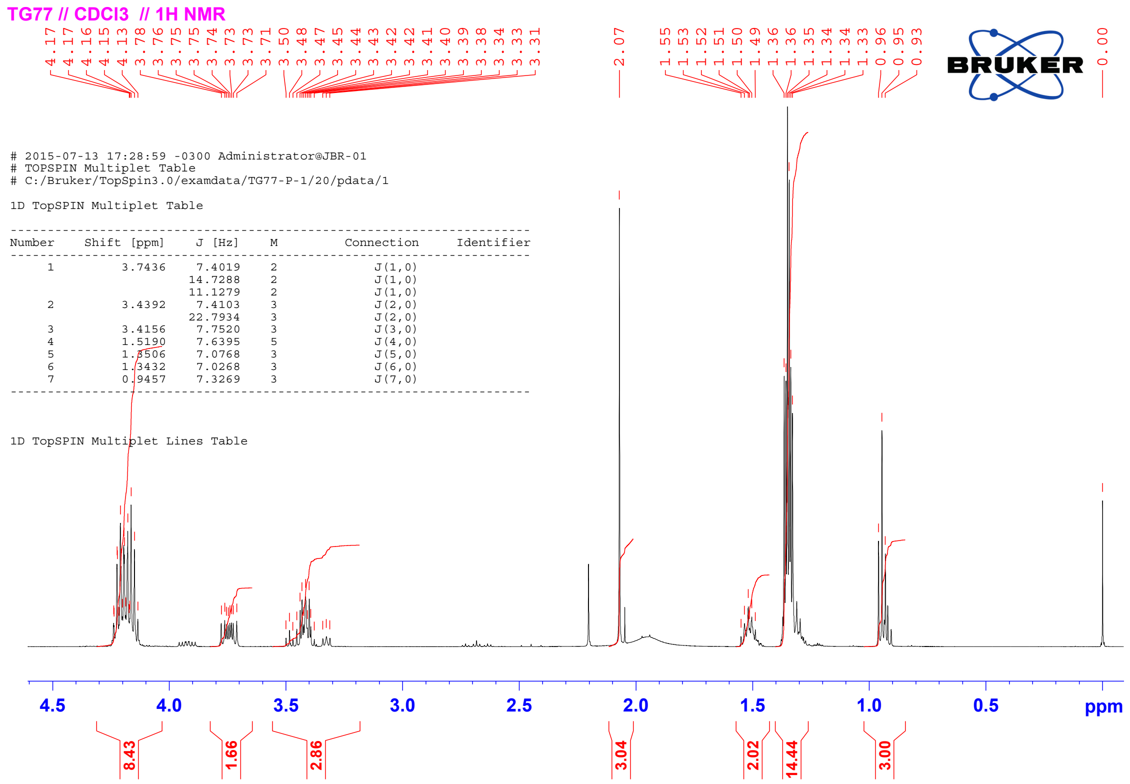

^{1}H NMR spectrum of compound **68**.

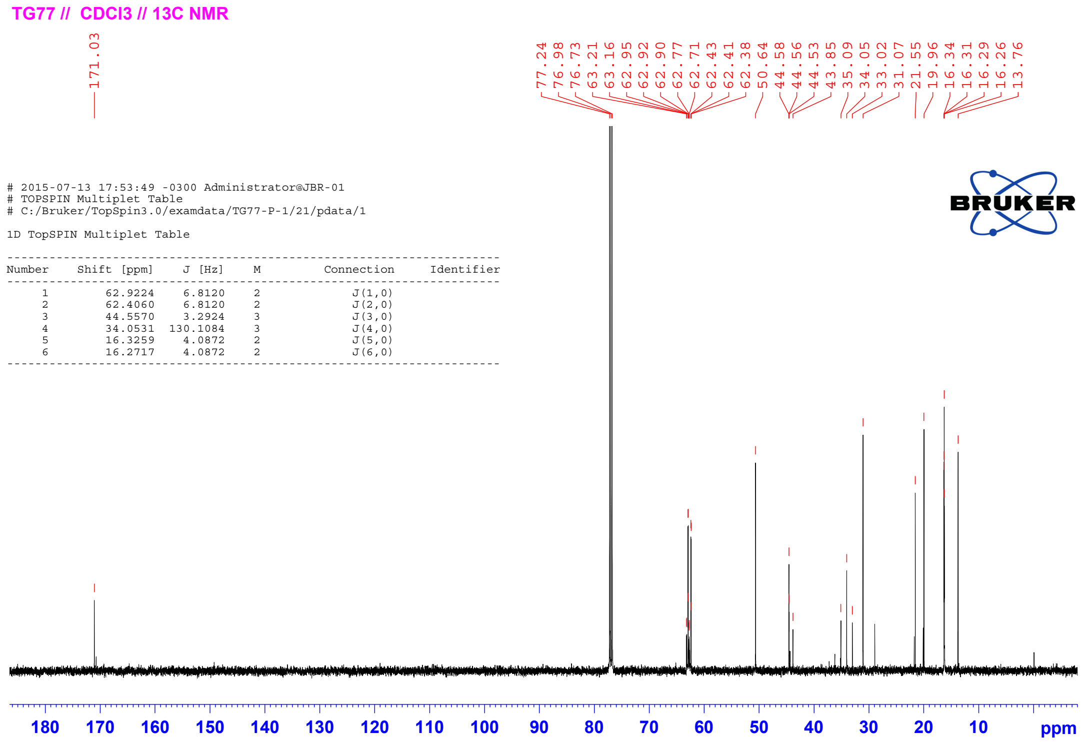

^{13}C NMR spectrum of compound **68**.

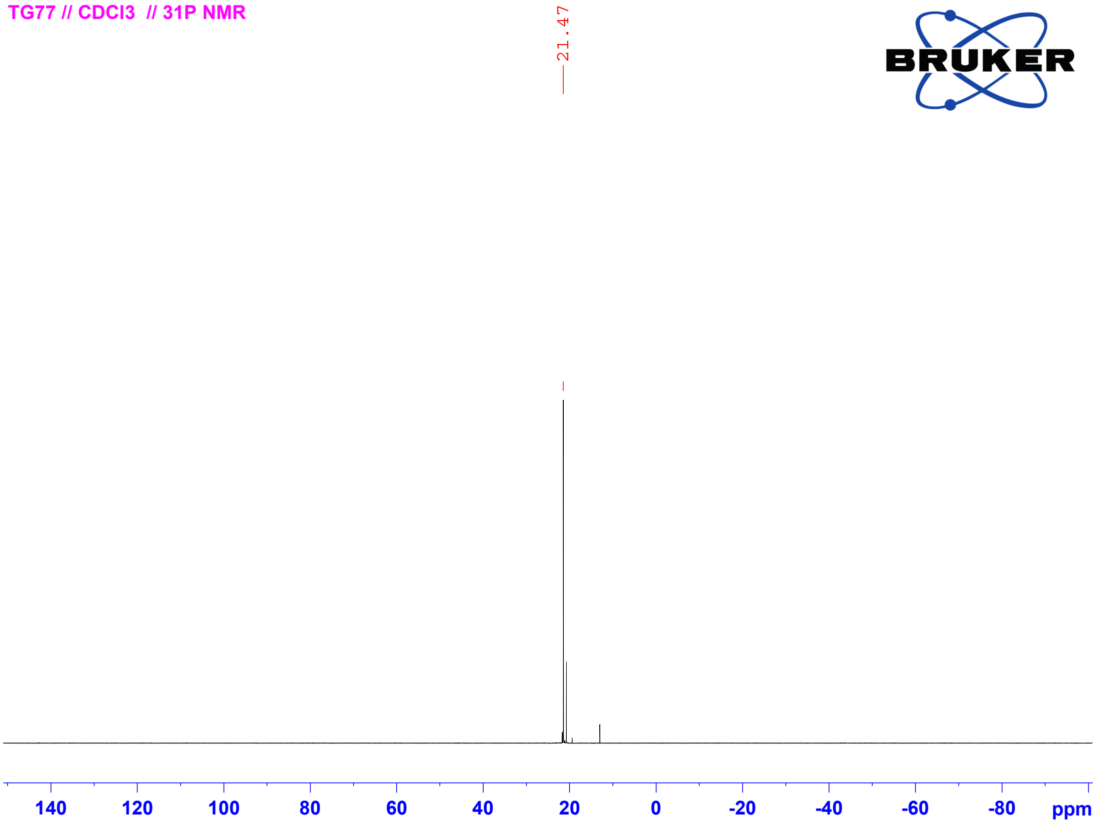

^{31}P NMR spectrum of compound **68**.

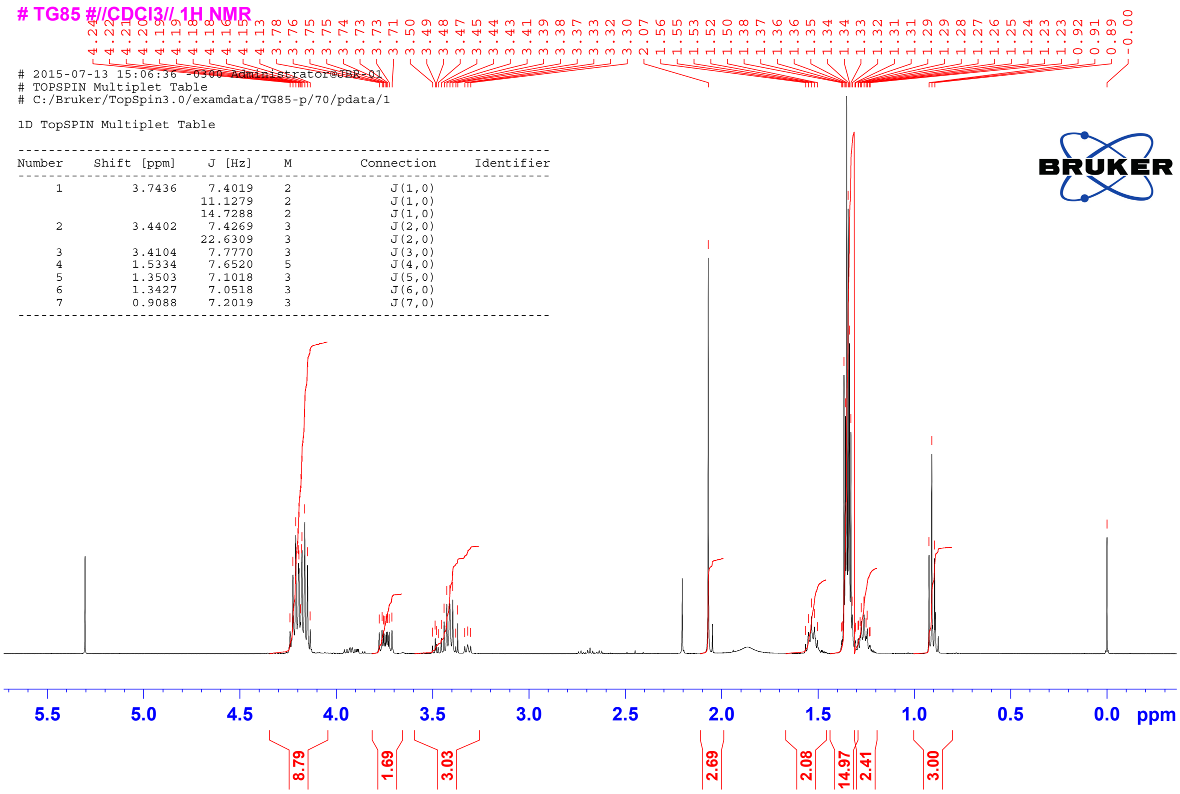

^{1}H NMR spectrum of compound **69**.

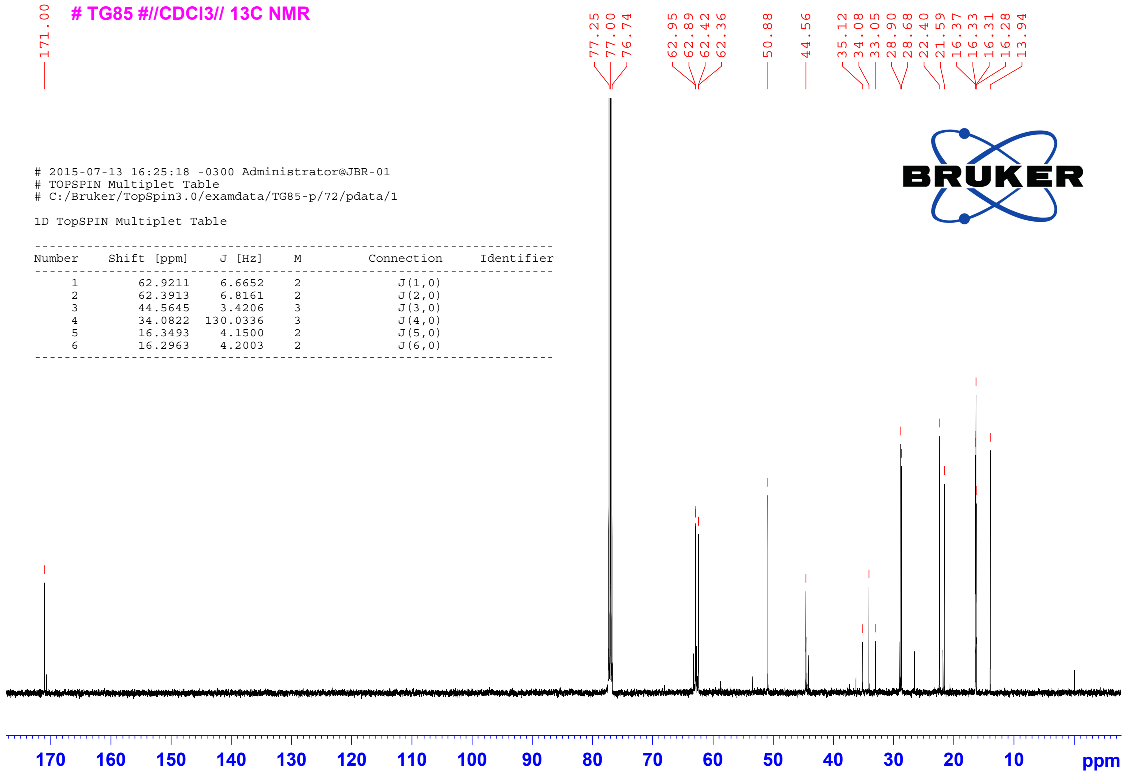

^{13}C NMR spectrum of compound **69**.

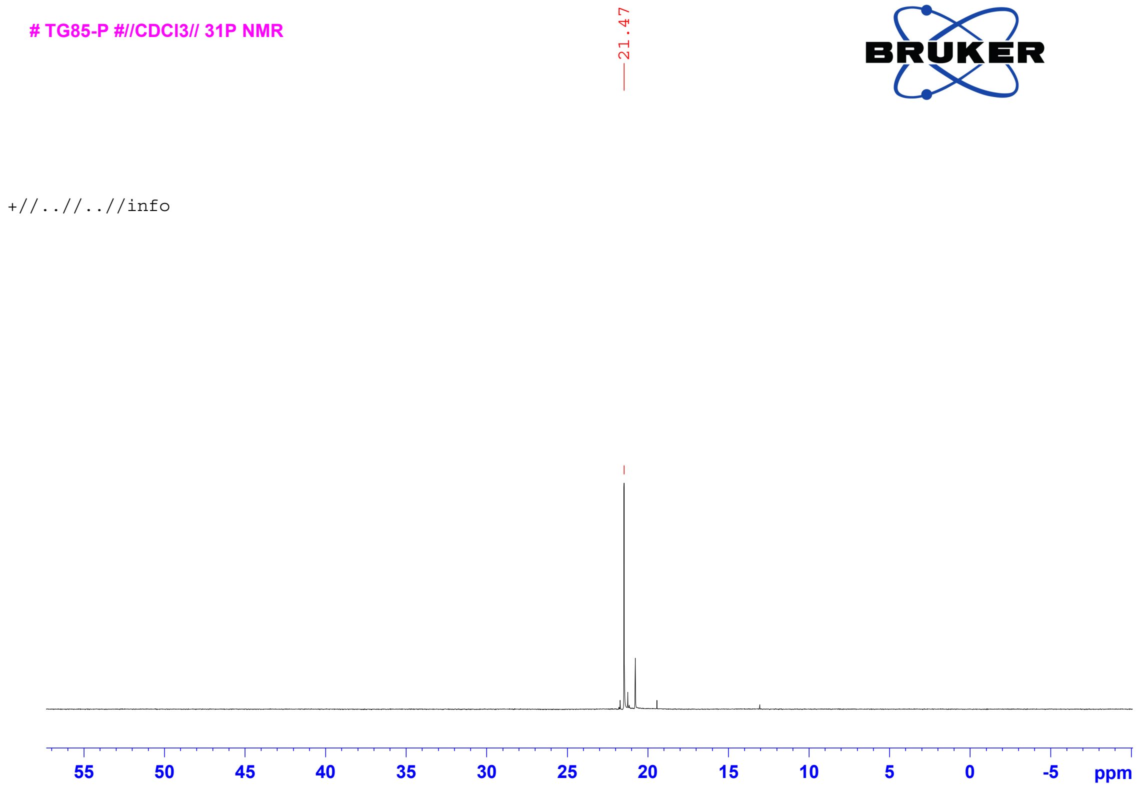

^{31}P NMR spectrum of compound **69**.

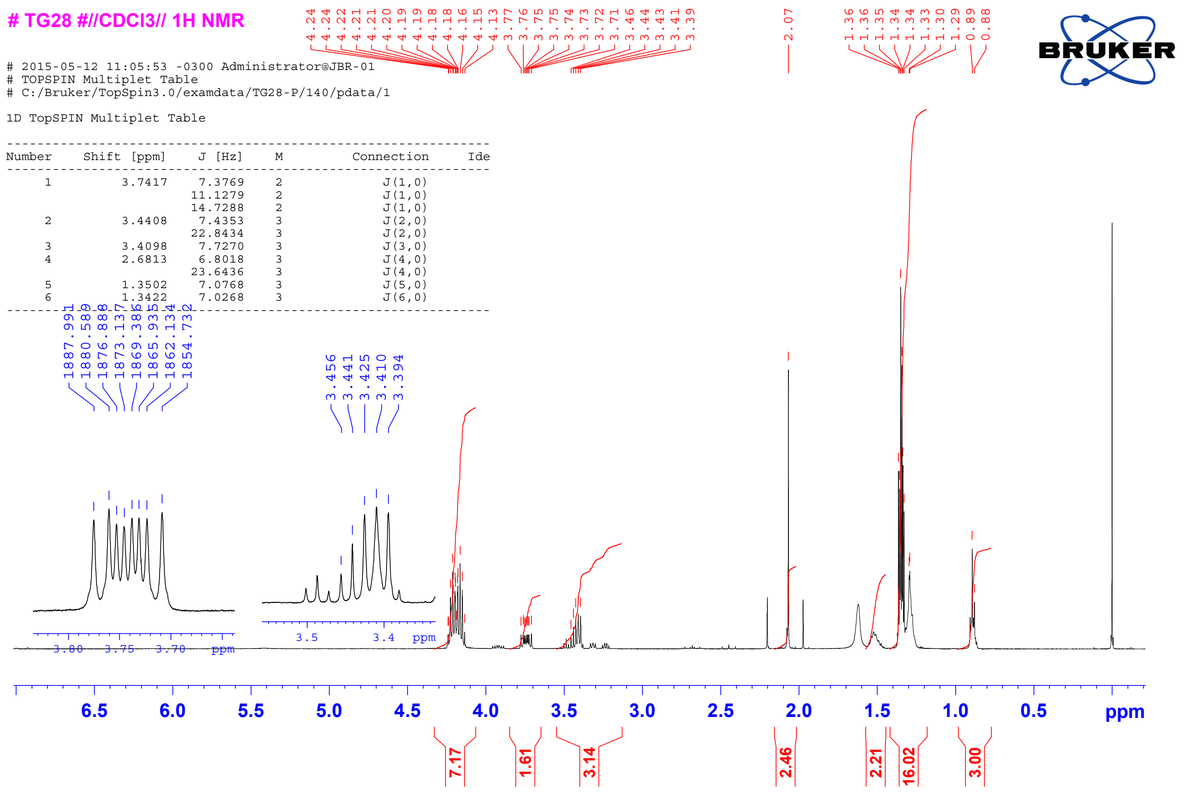

Number	Shift [ppm]	J [Hz]	M	Connection	Ide
1	3.7417	7.3769	2	J(1,0)	
		11.1279	2	J(1,0)	
		14.7288	2	J(1,0)	
2	3.4408	7.4353	3	J(2,0)	
		22.8434	3	J(2,0)	
3	3.4098	7.7270	3	J(3,0)	
4	2.6813	6.8018	3	J(4,0)	
		23.6436	3	J(4,0)	
5	1.3502	7.0768	3	J(5,0)	
6	1.3422	7.0268	3	J(6,0)	

^{1}H NMR spectrum of compound **70**.

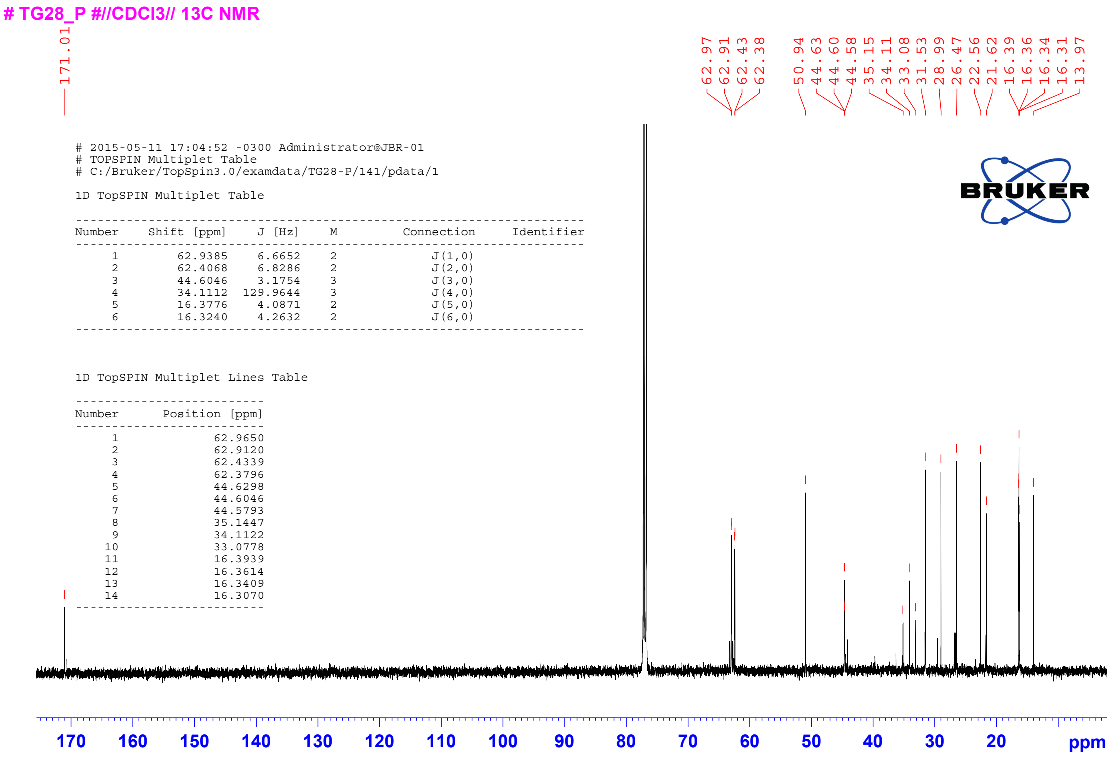

^{13}C NMR spectrum of compound **70**.

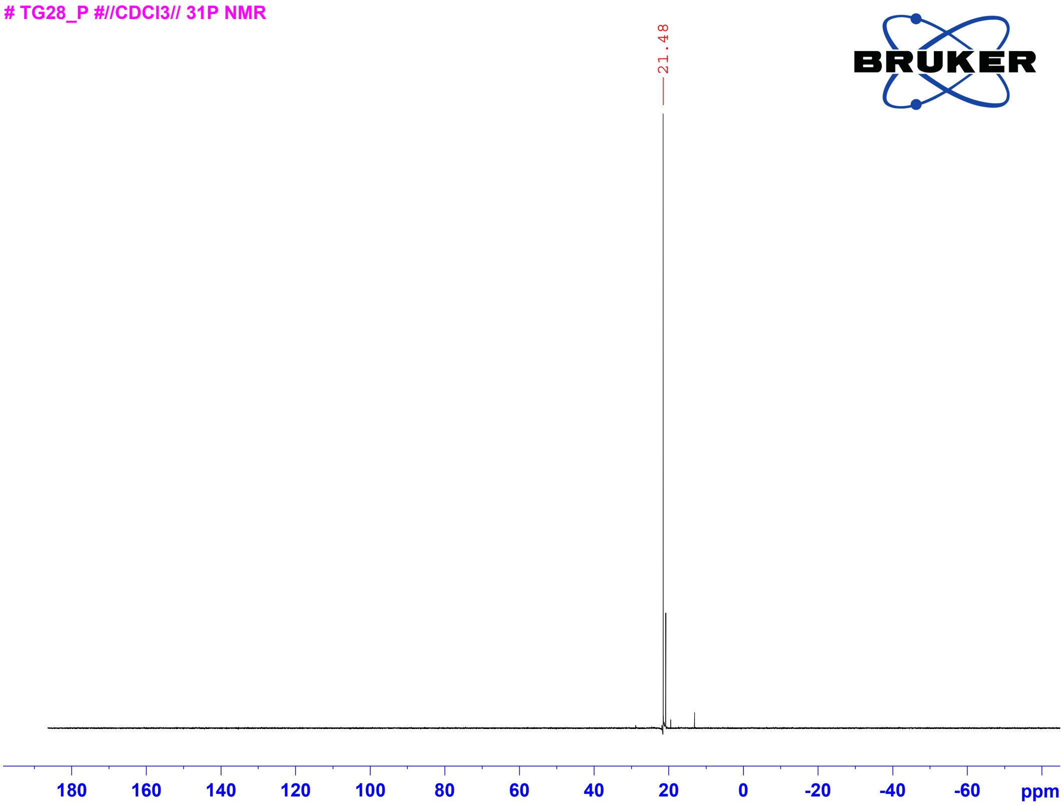

^{31}P NMR spectrum of compound **70**.

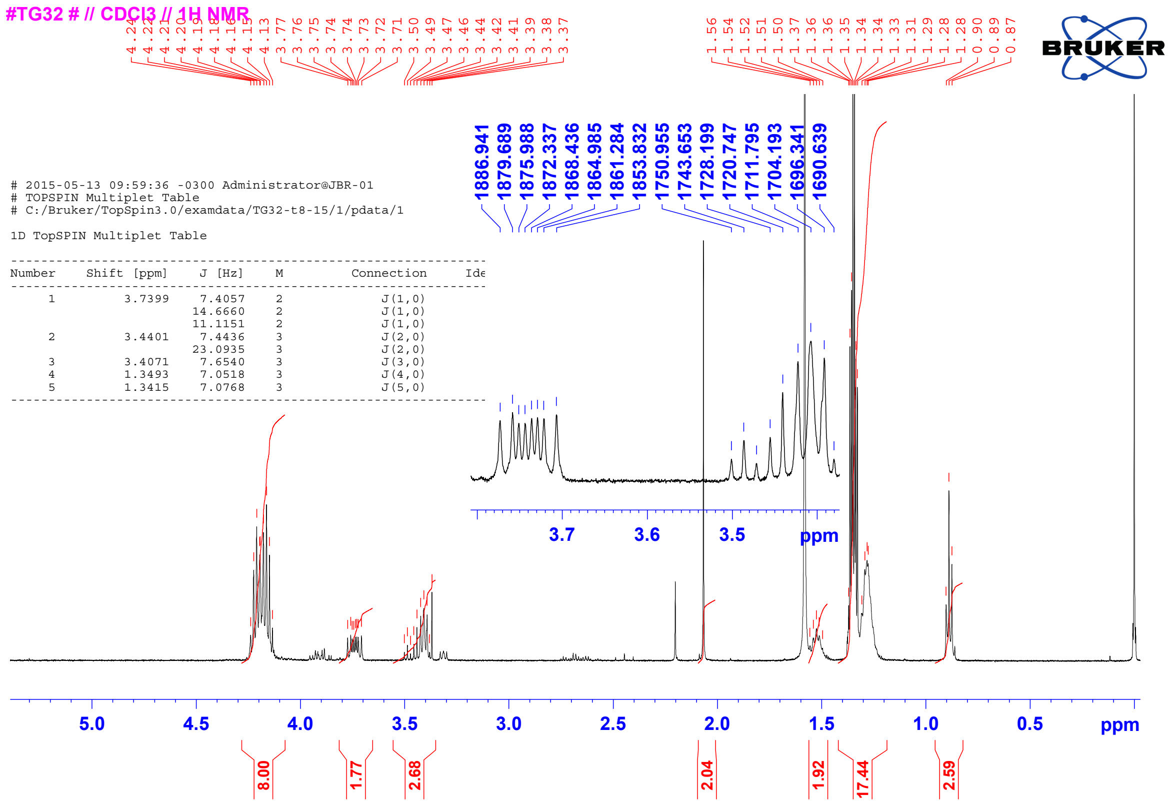

^{1}H NMR spectrum of compound **71**.

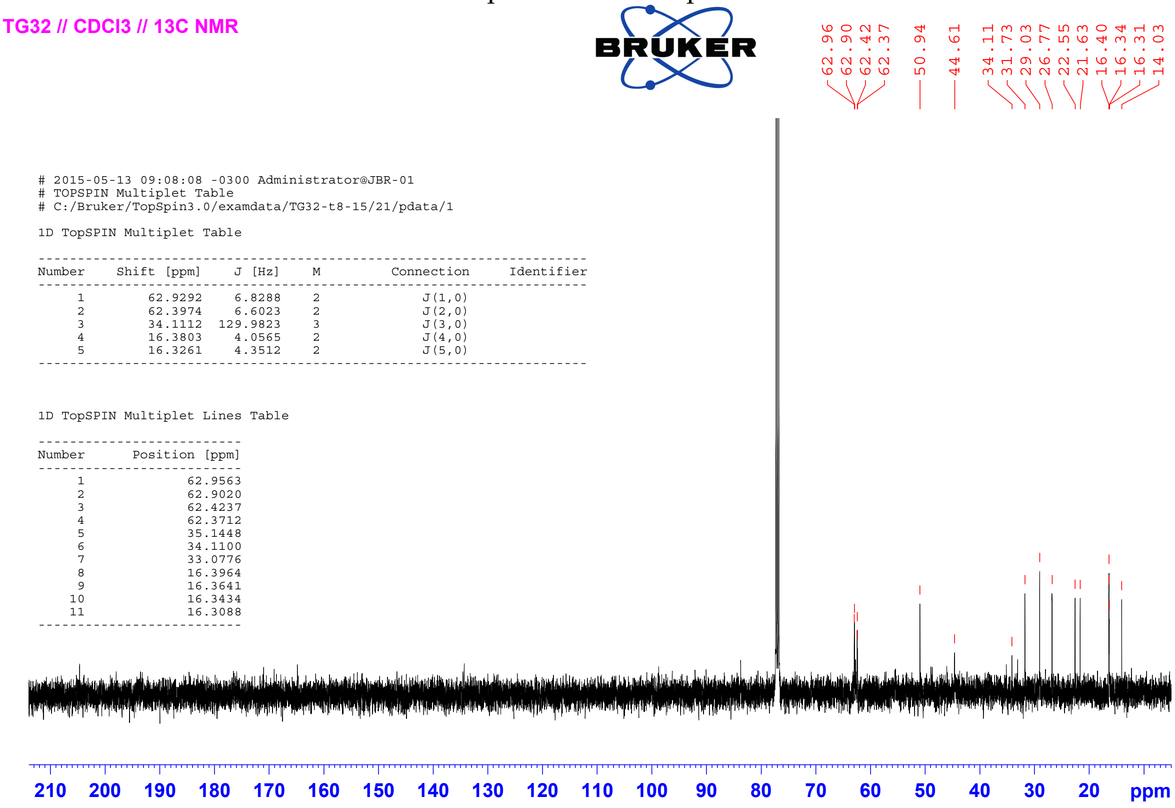

^{13}C NMR spectrum of compound **71**.

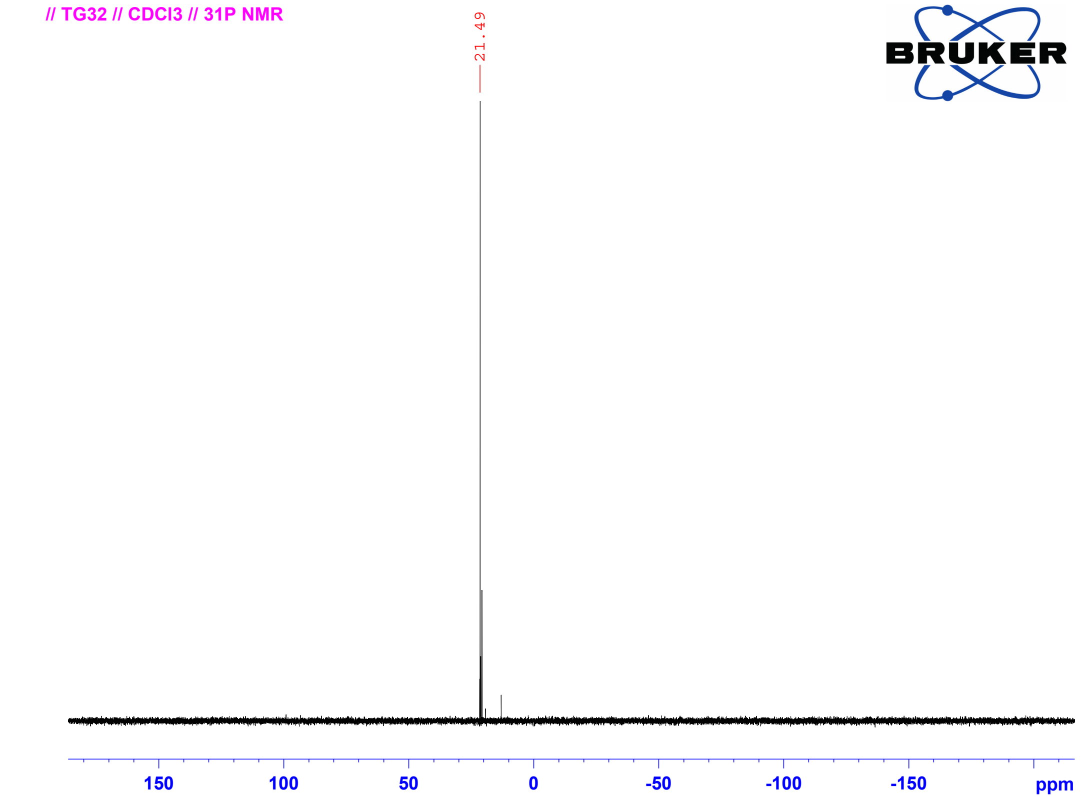

^{31}P NMR spectrum of compound **71**.

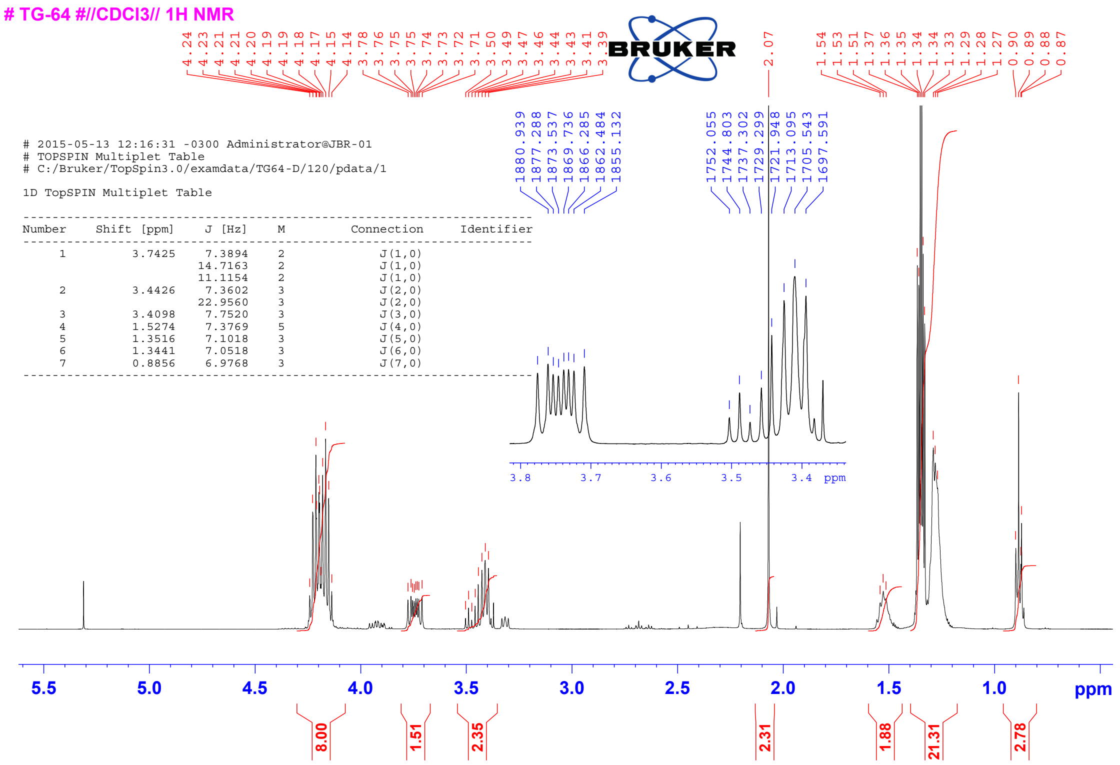

^{1}H NMR spectrum of compound **72**.

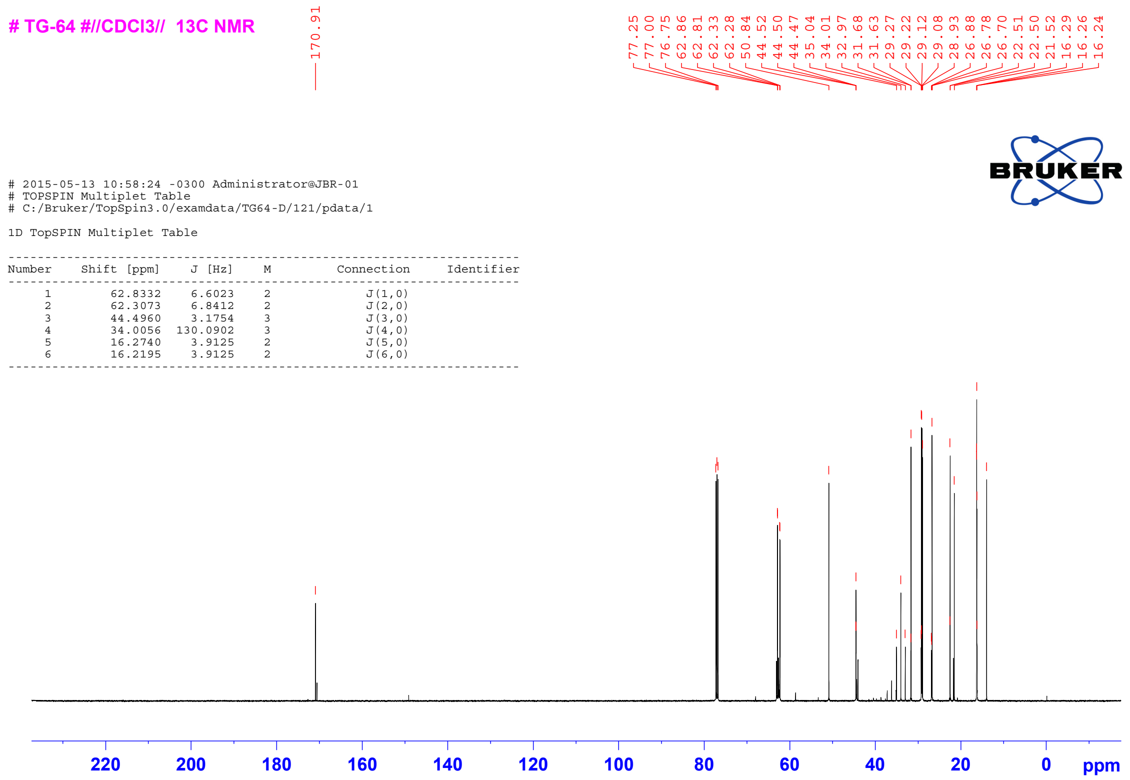

^{13}C NMR spectrum of compound **72**.

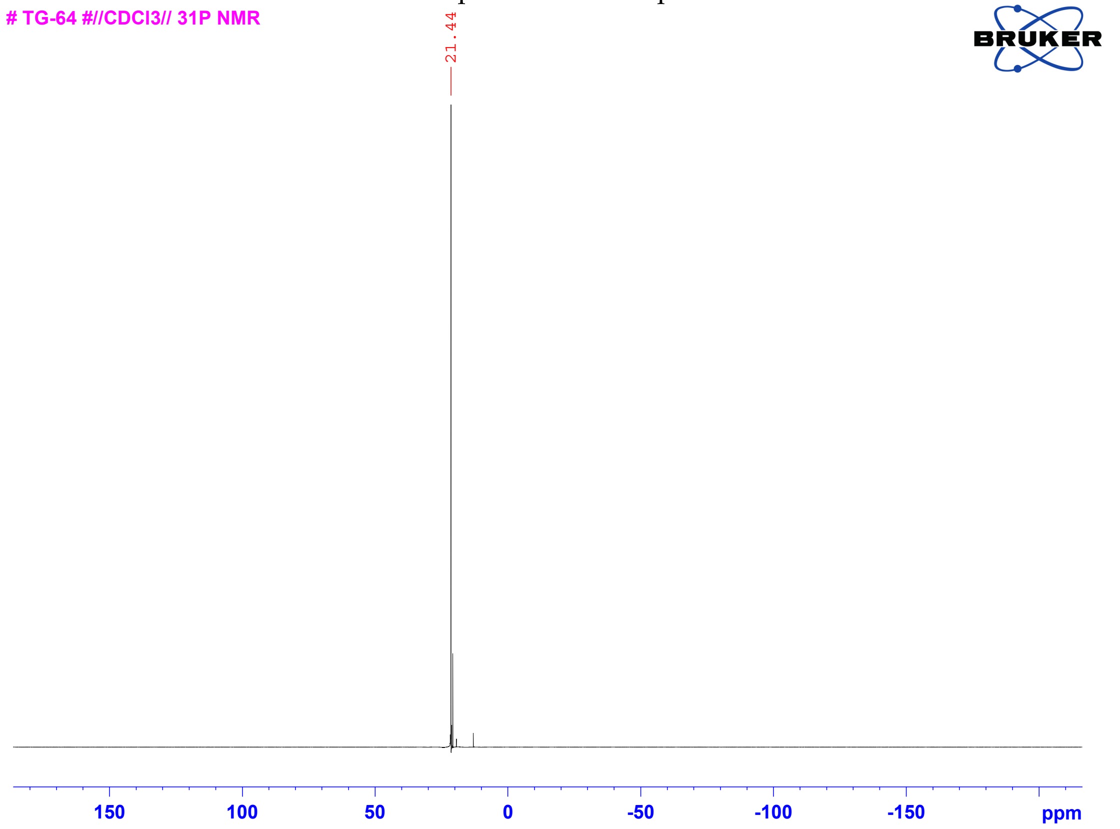

^{31}P NMR spectrum of compound **72**.

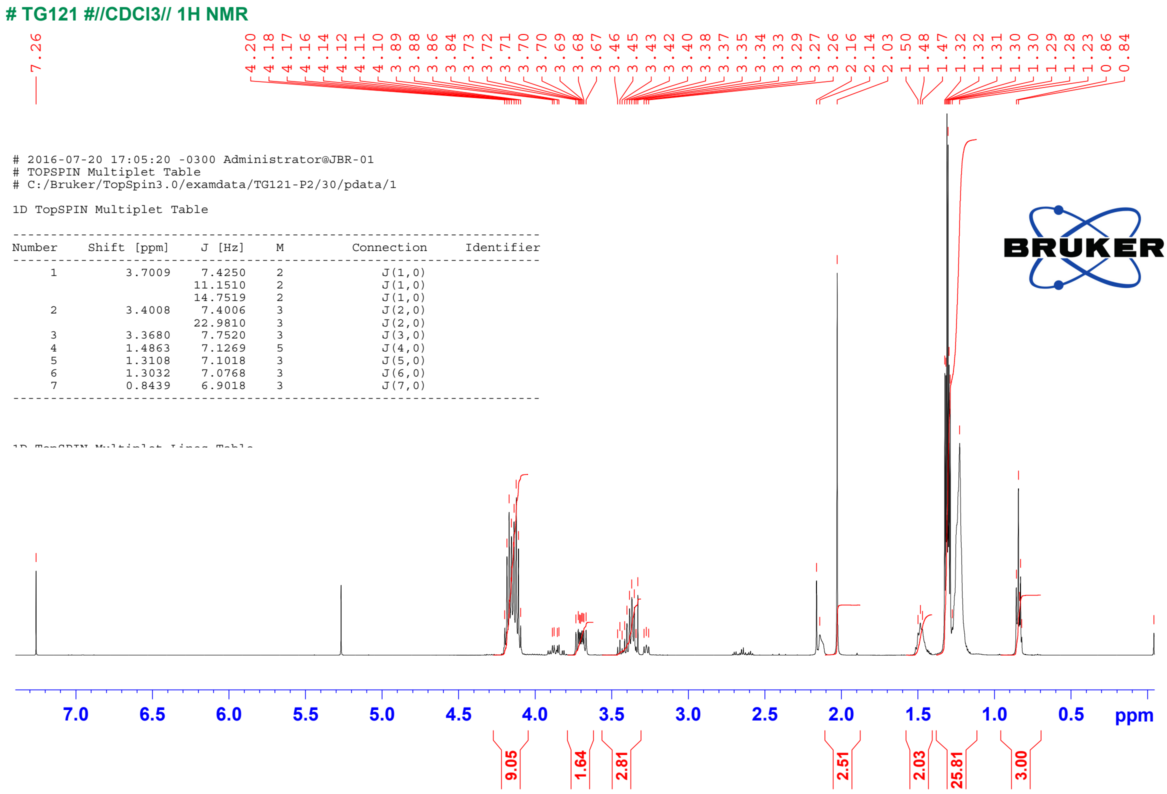

^{1}H NMR spectrum of compound **73**.

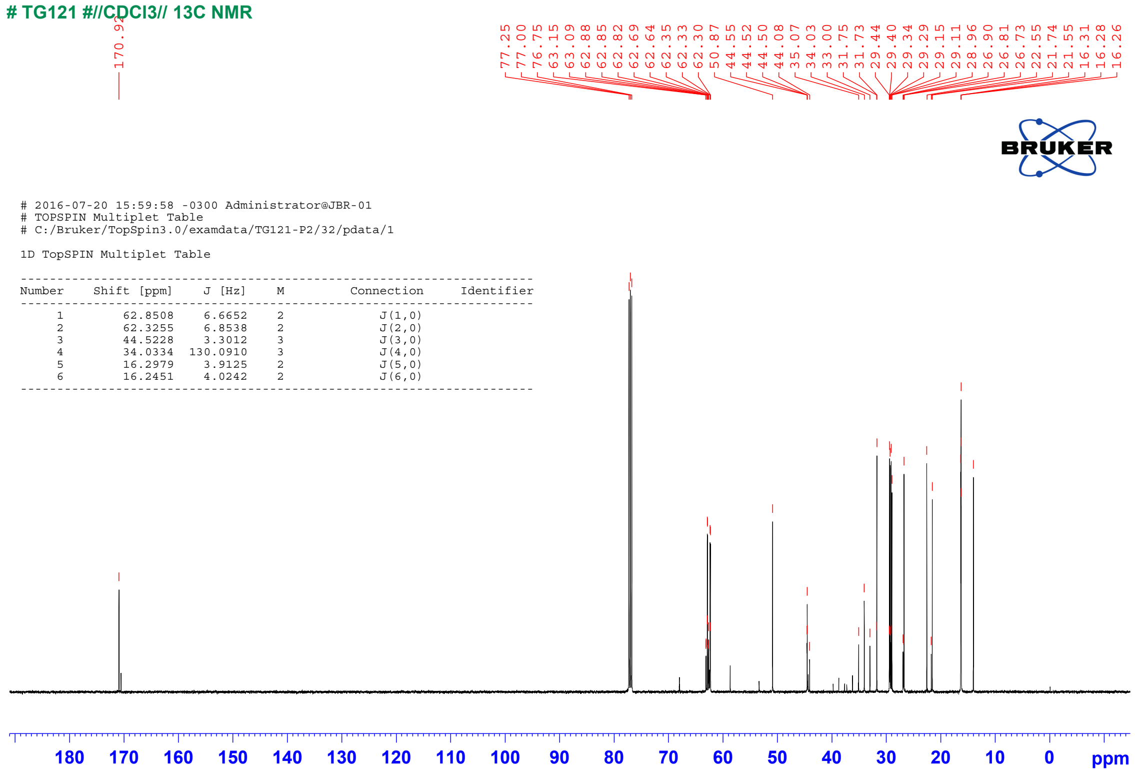

^{13}C NMR spectrum of compound **73**.

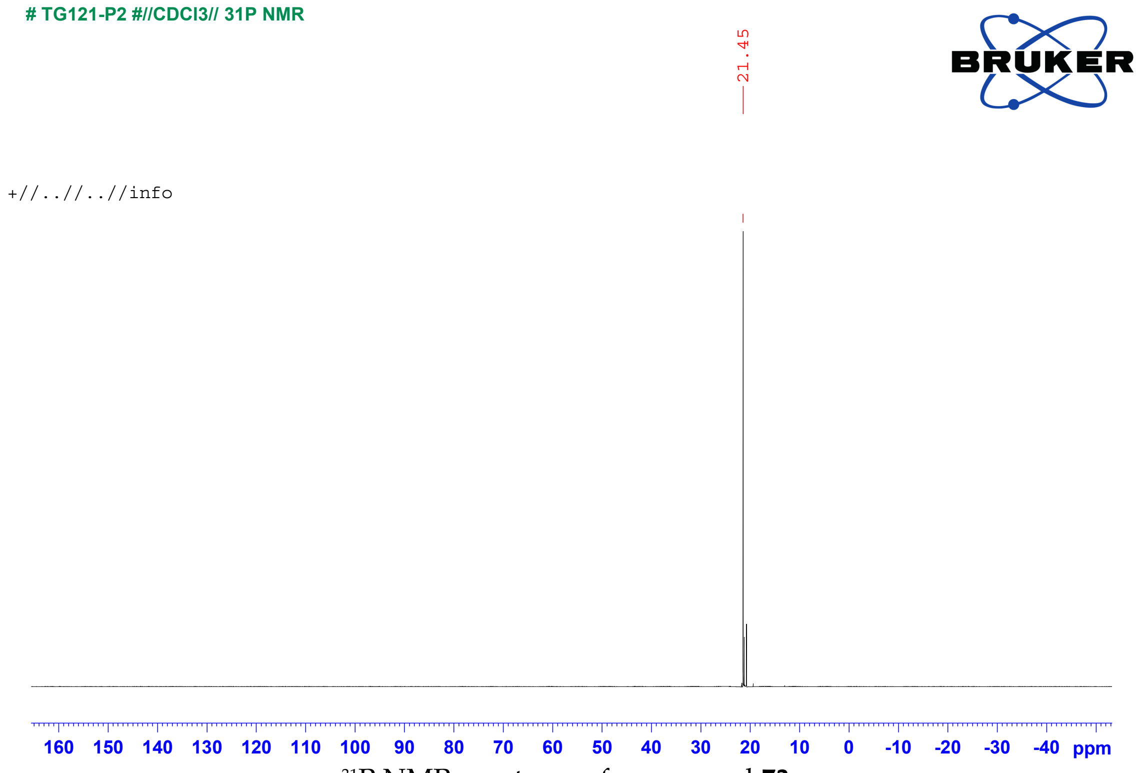

^{31}P NMR spectrum of compound **73**.

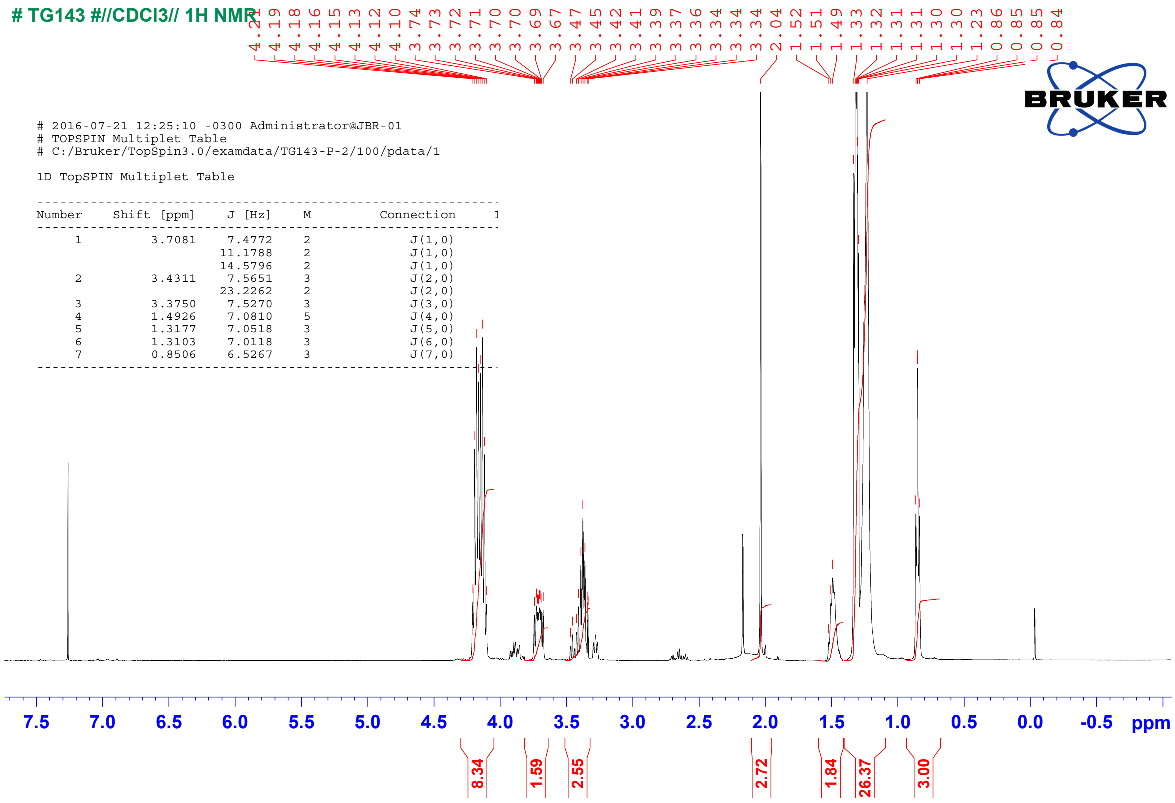

^{1}H NMR spectrum of compound **74**.

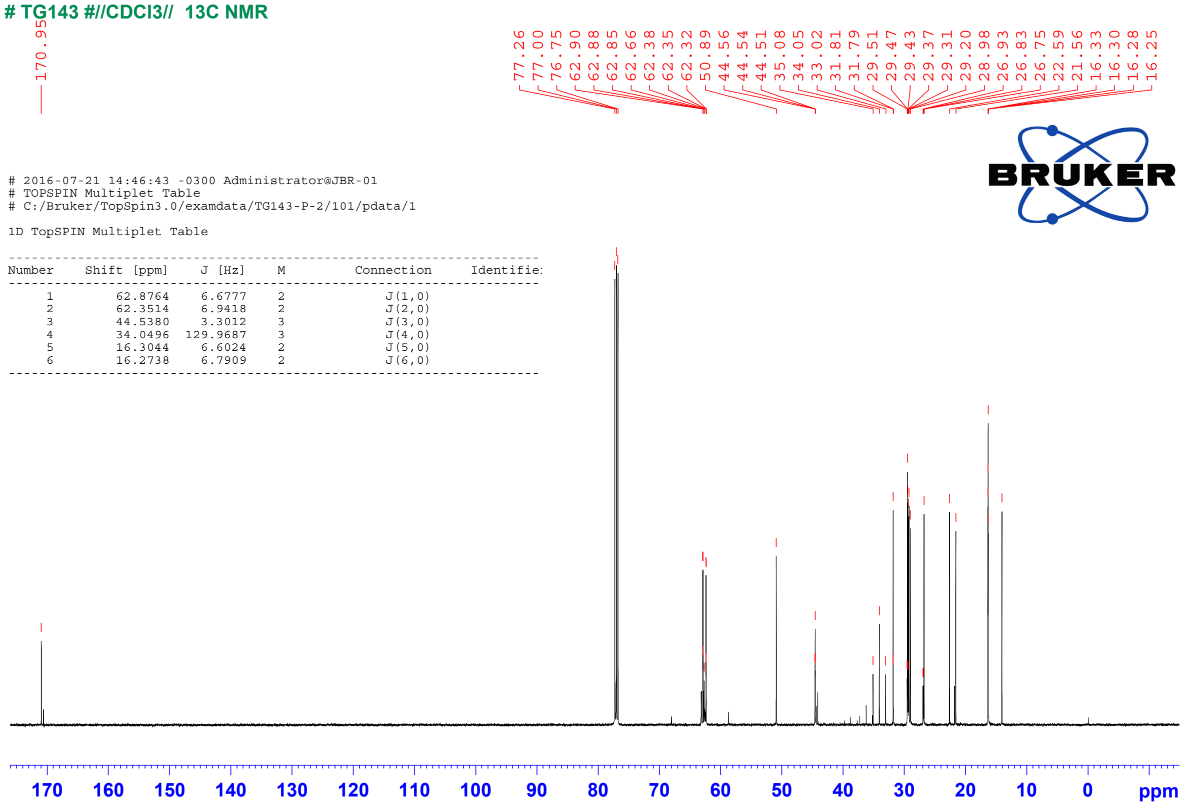

^{13}C NMR spectrum of compound **74**.

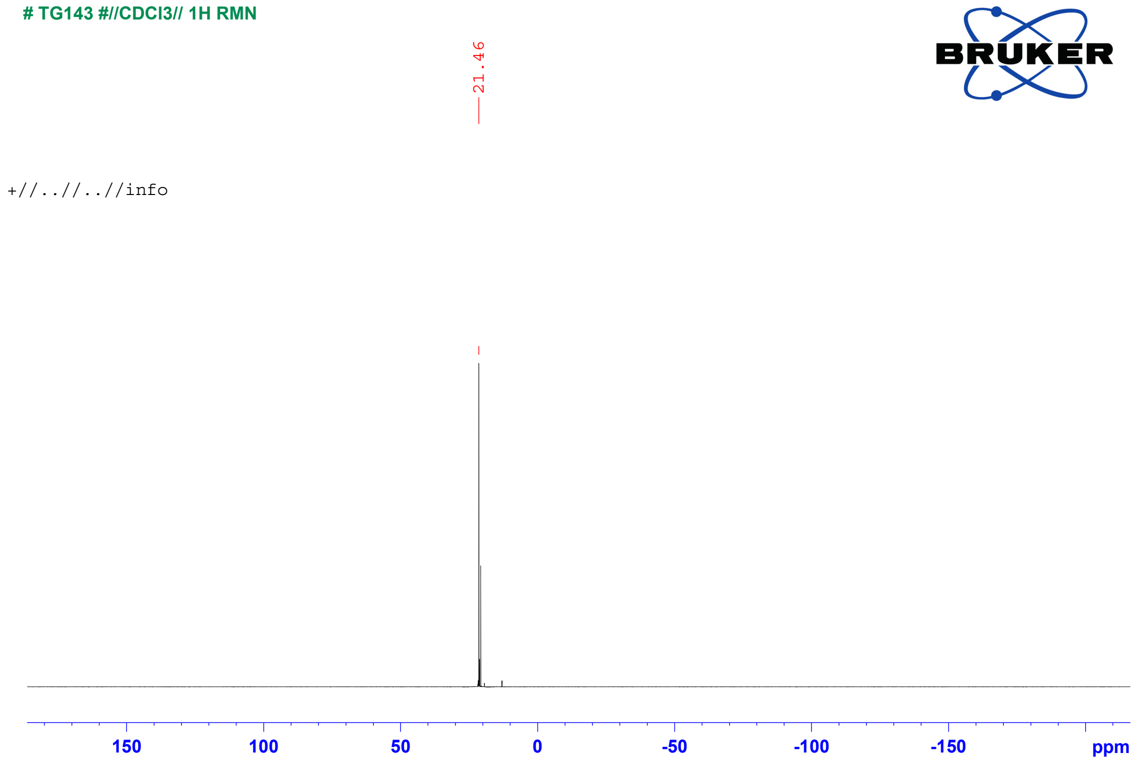

^{31}P NMR spectrum of compound **74**.

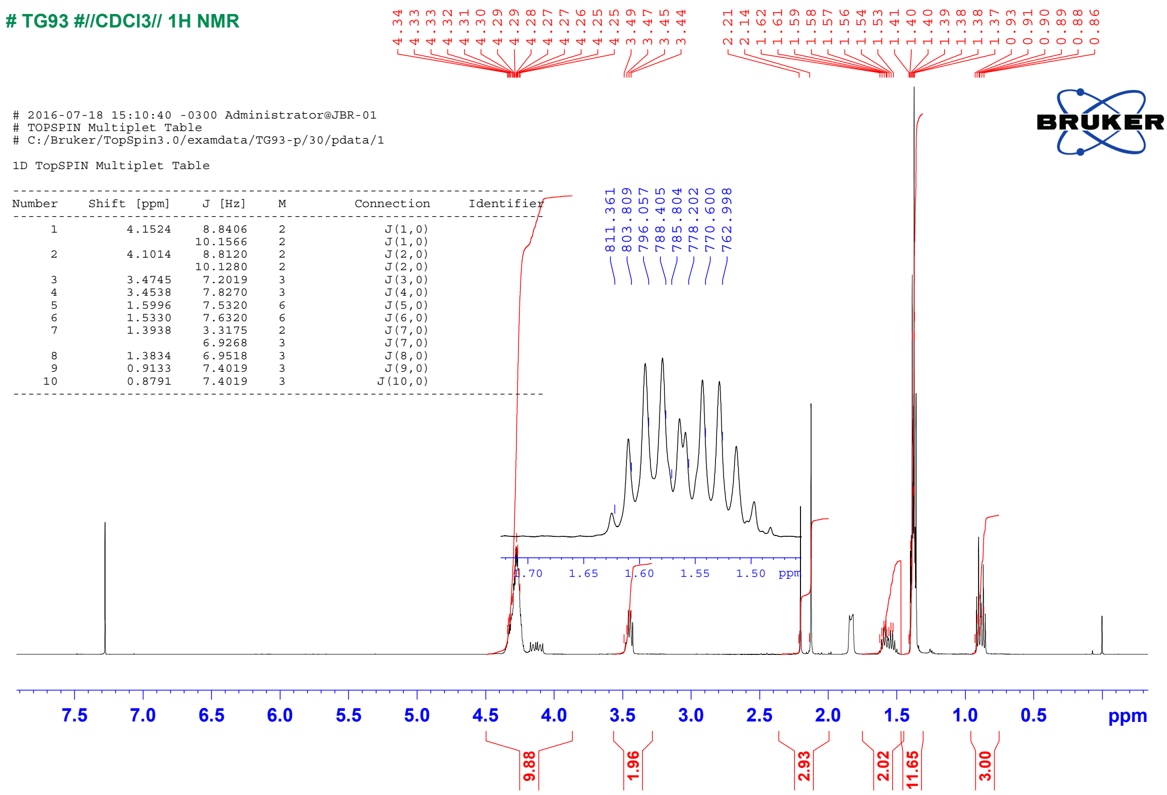

^{1}H NMR spectrum of compound **75**.

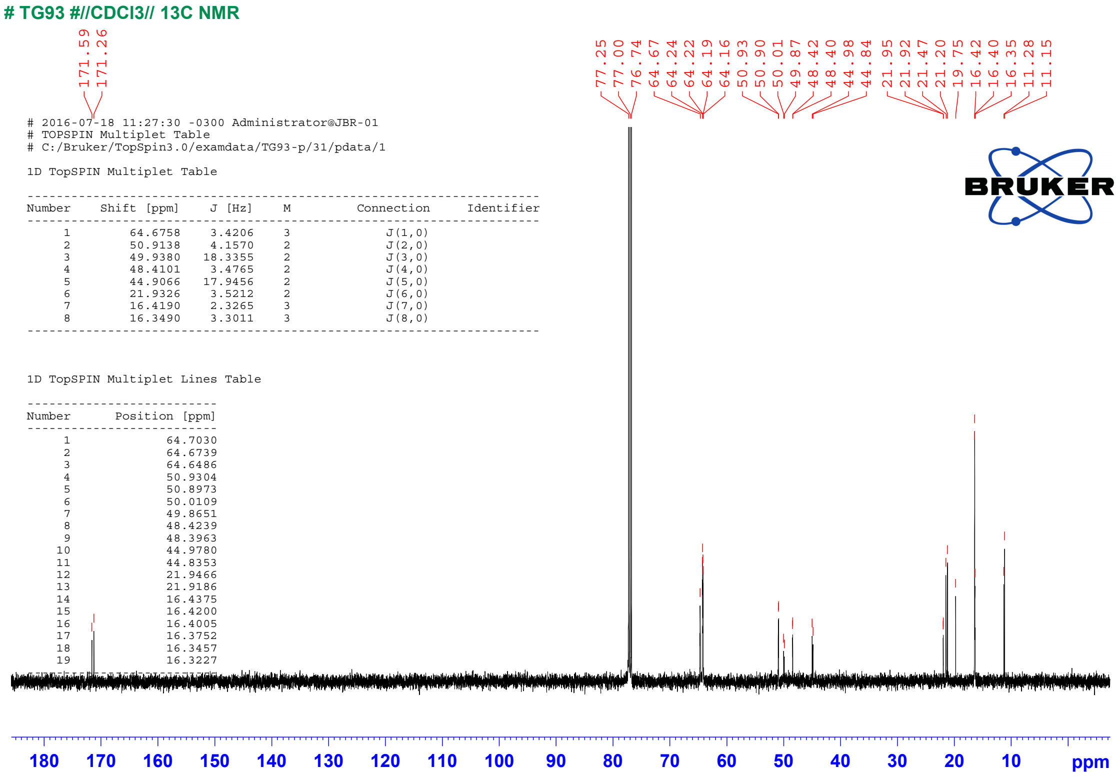

^{13}C NMR spectrum of compound **75**.

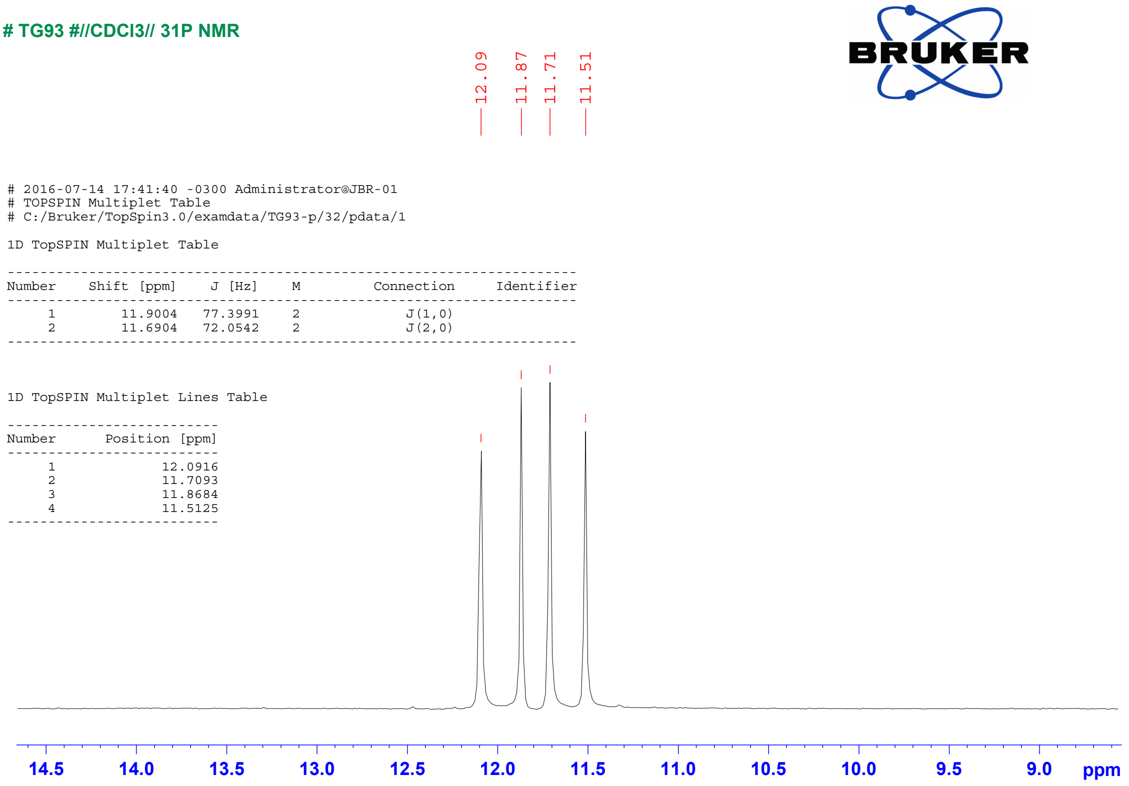

^{31}P NMR spectrum of compound **75**.

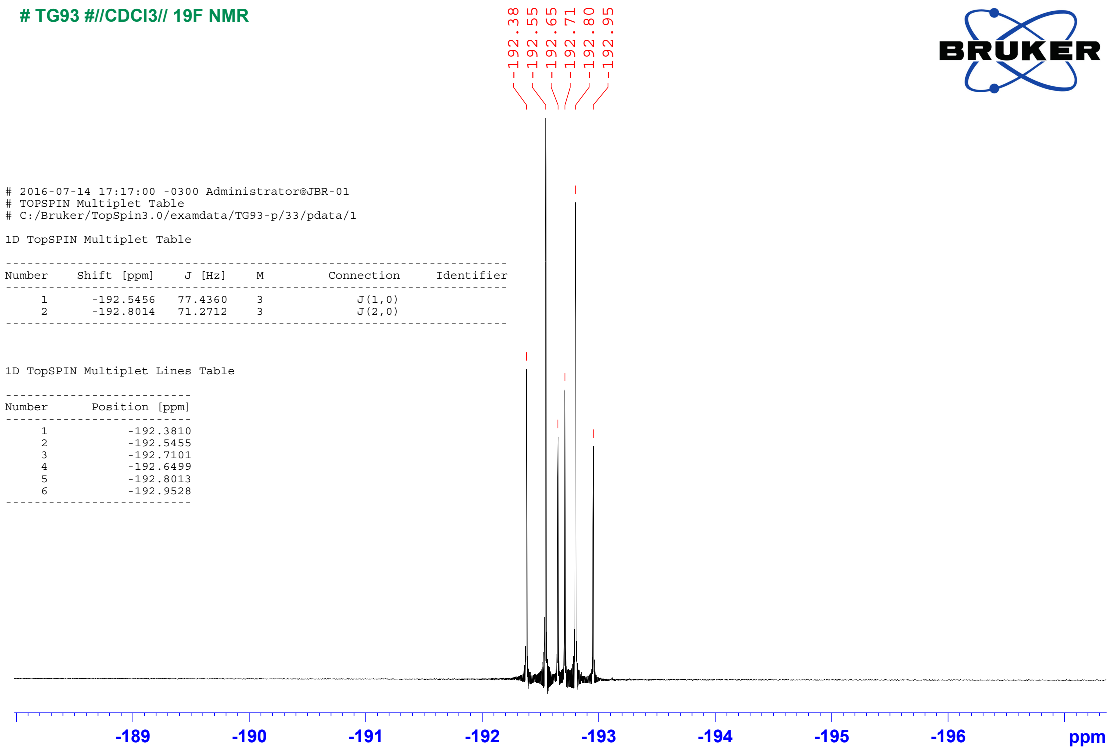

^{19}F NMR spectrum of compound **75**.

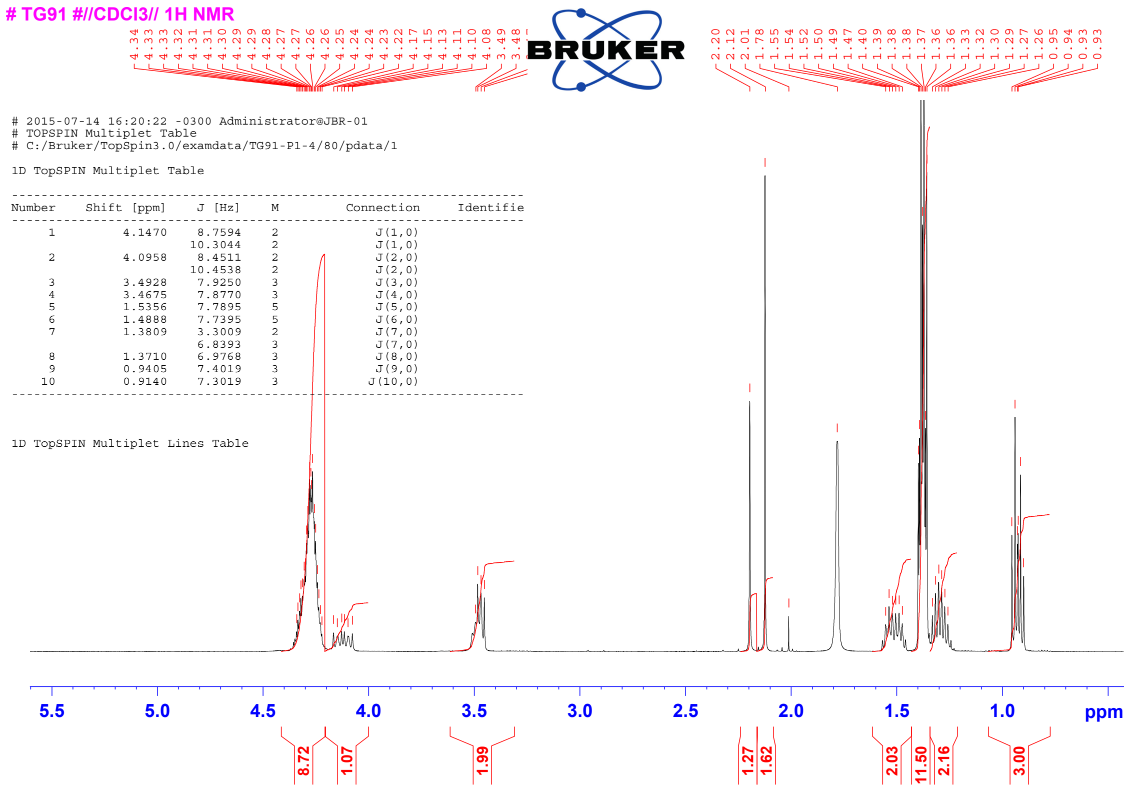

^{1}H NMR spectrum of compound **76**.

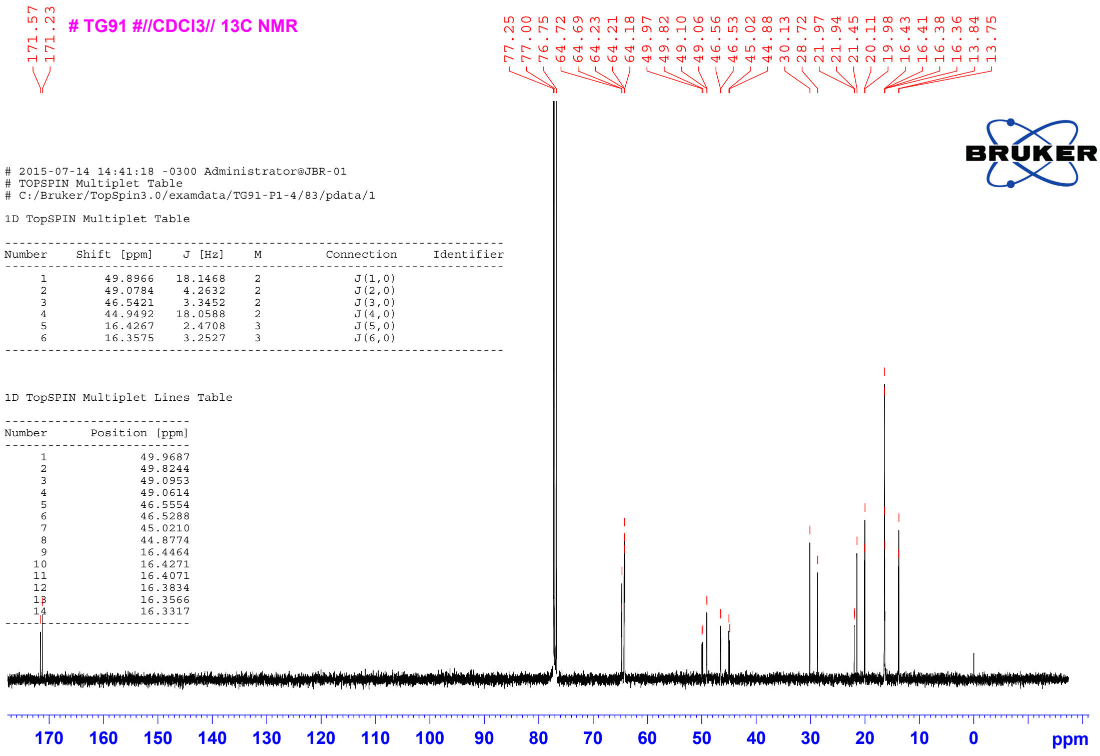

^{13}C NMR spectrum of compound **76**.

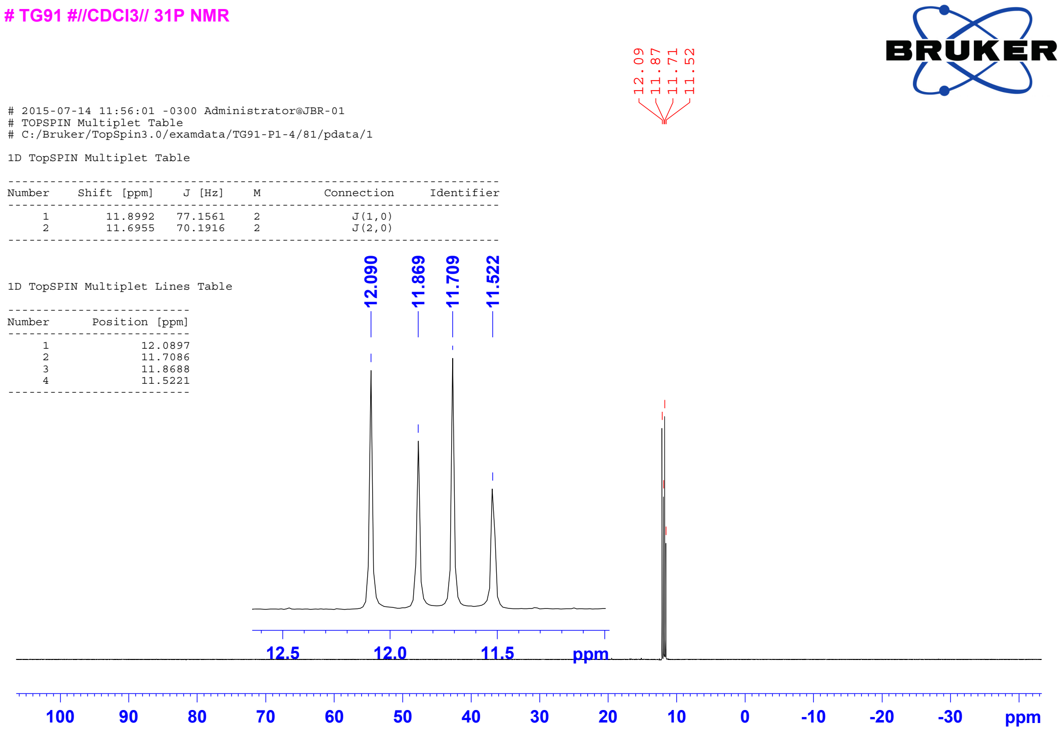

^{31}P NMR spectrum of compound **76**.

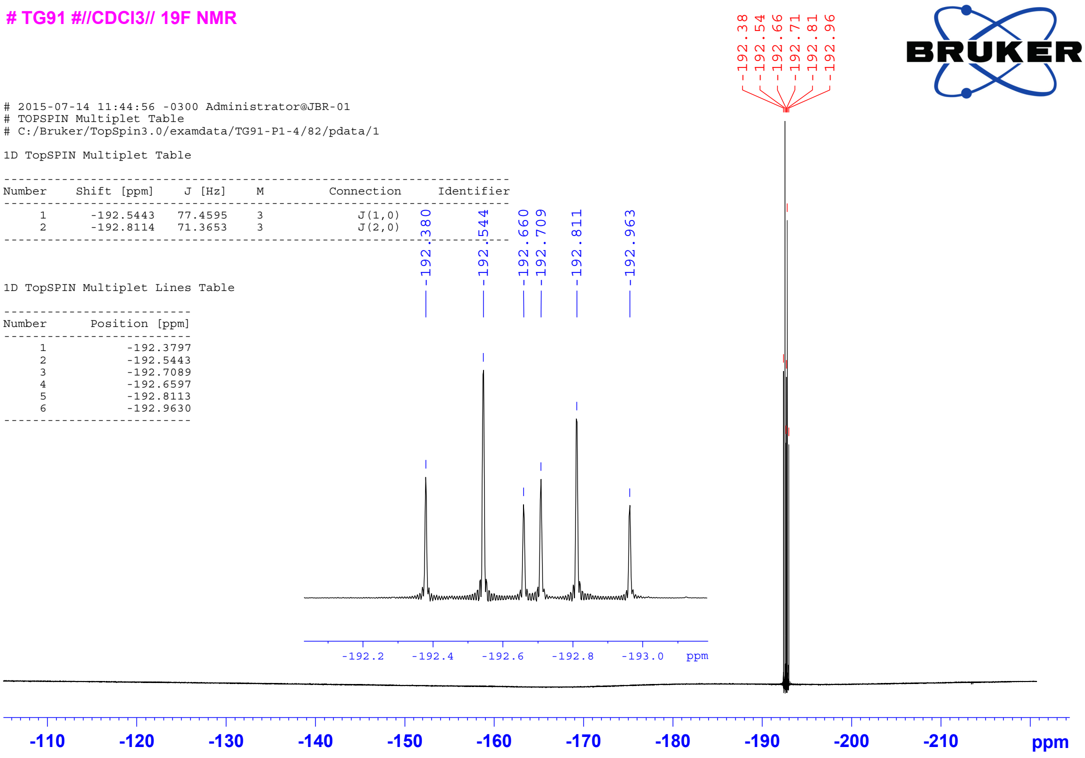

^{19}F NMR spectrum of compound **76**.

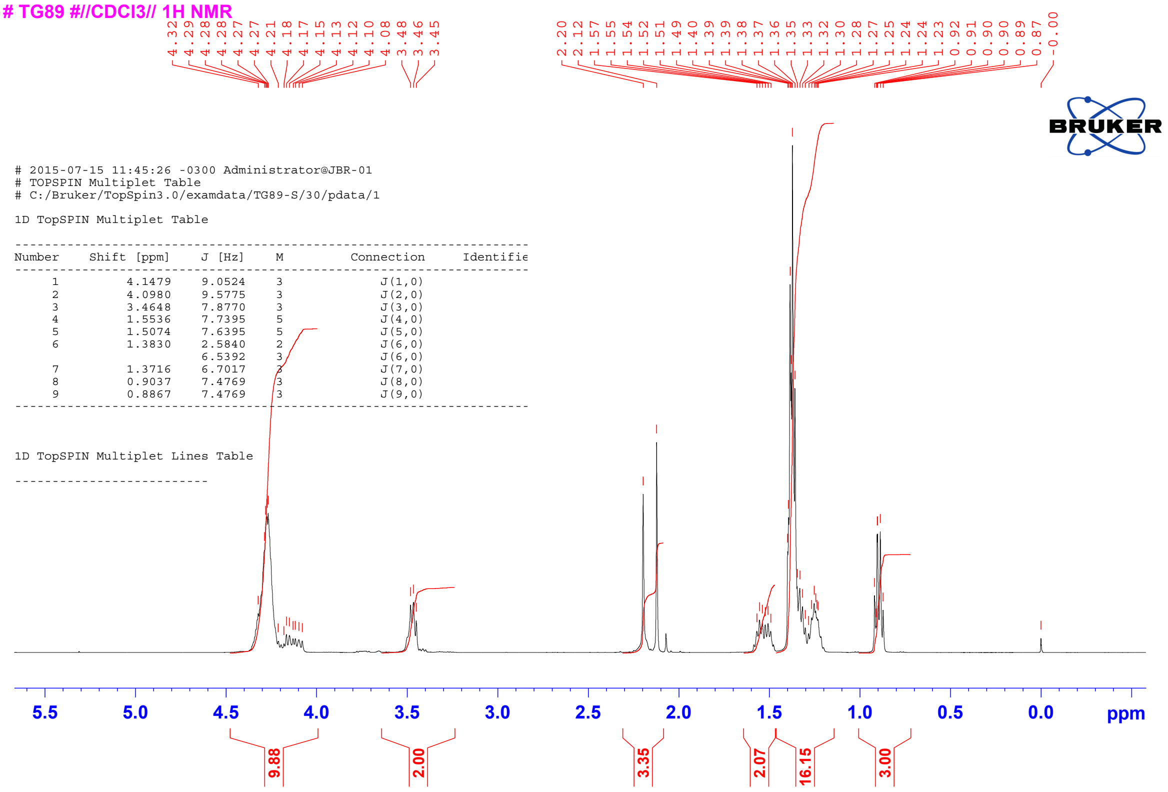

^{1}H NMR spectrum of compound **77**.

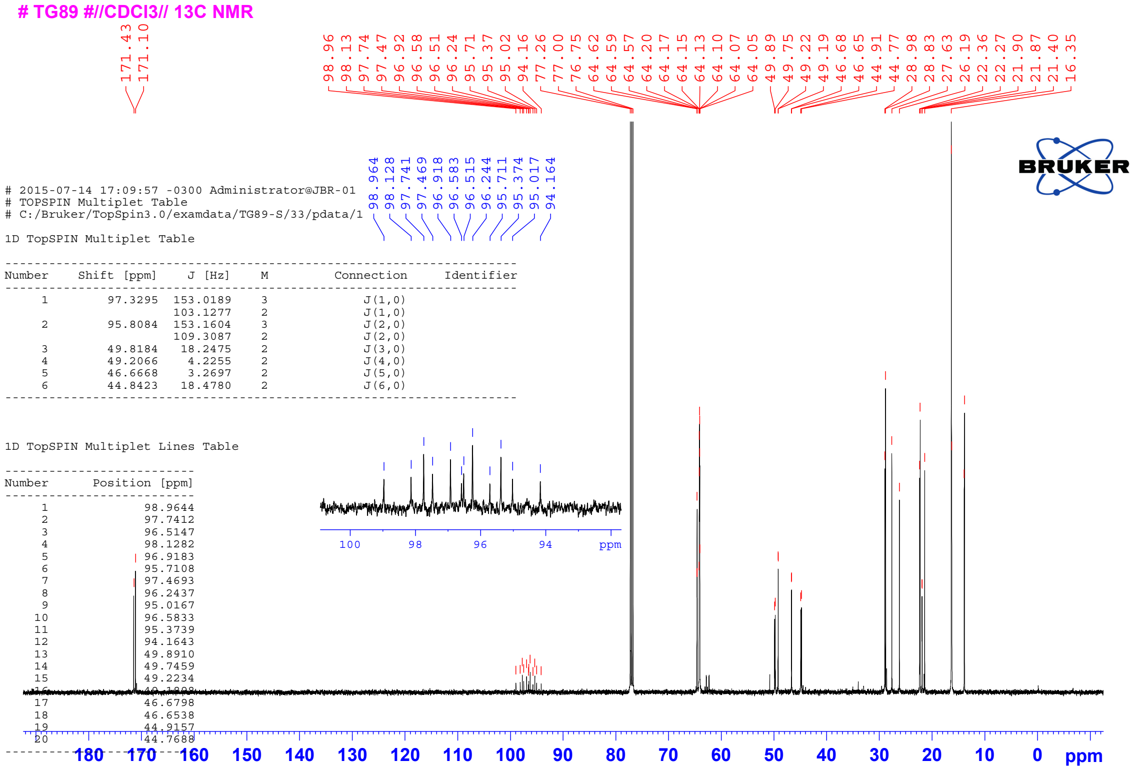

^{13}C NMR spectrum of compound **77**.

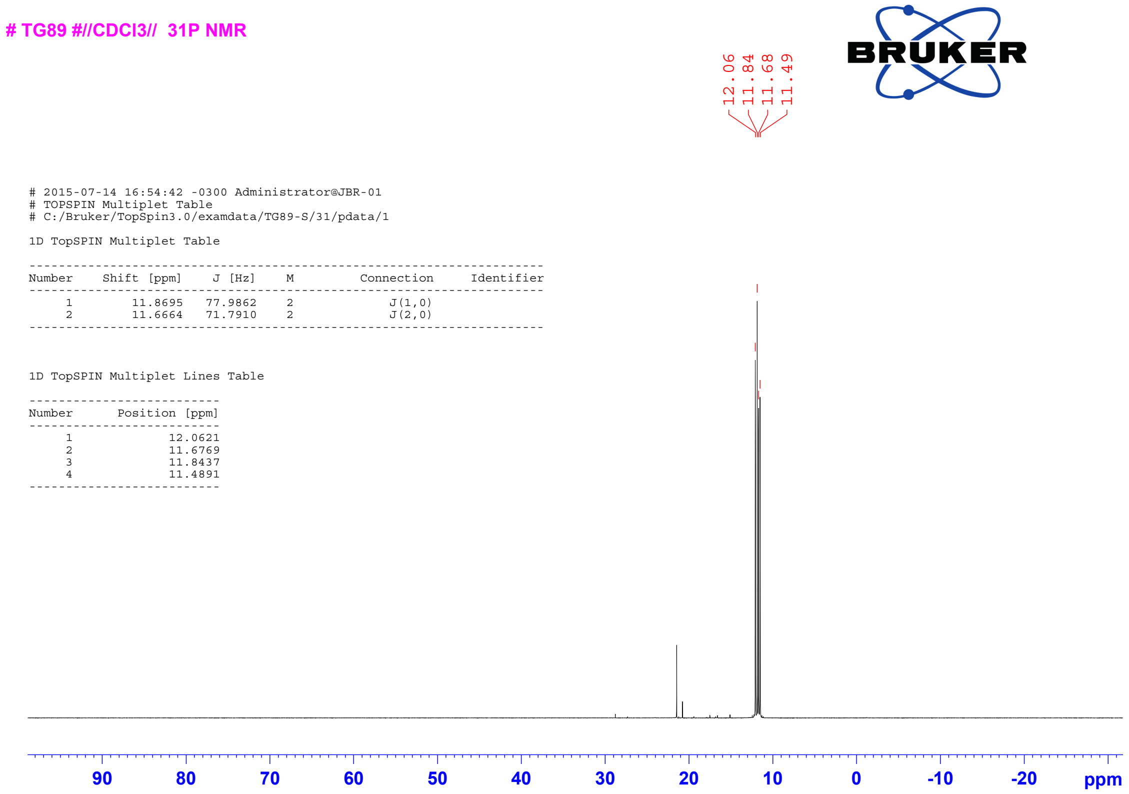

^{31}P NMR spectrum of compound **77**.

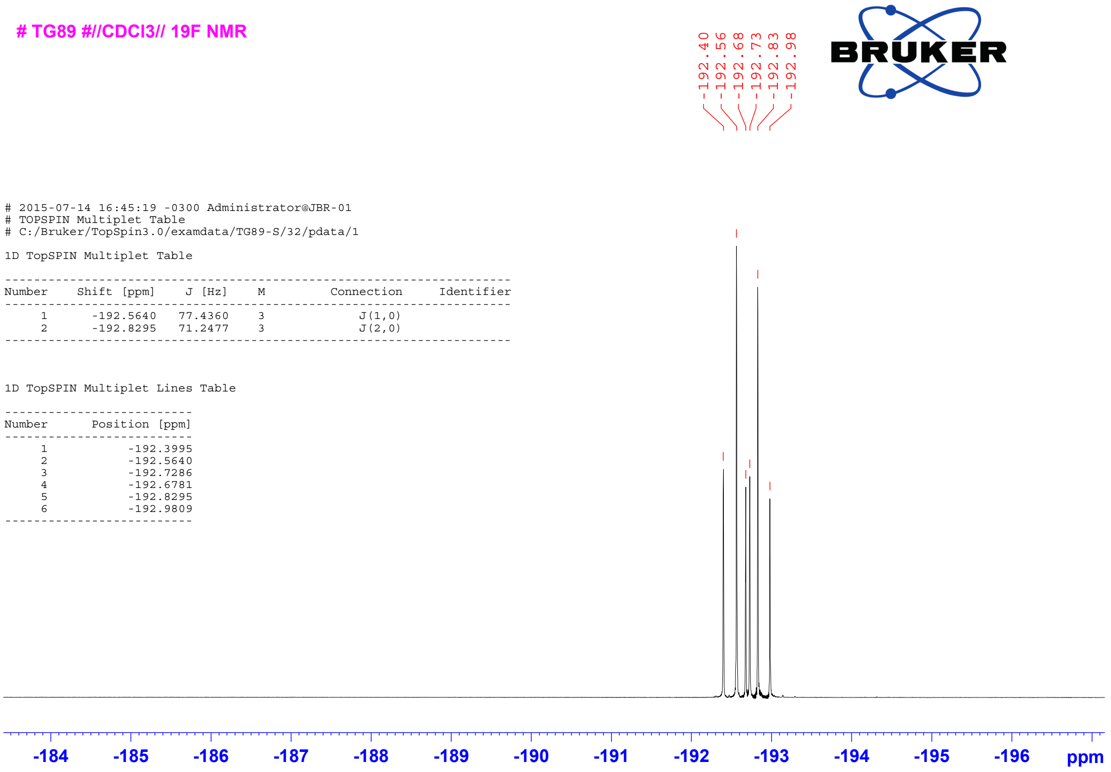

^{19}F NMR spectrum of compound **77**.

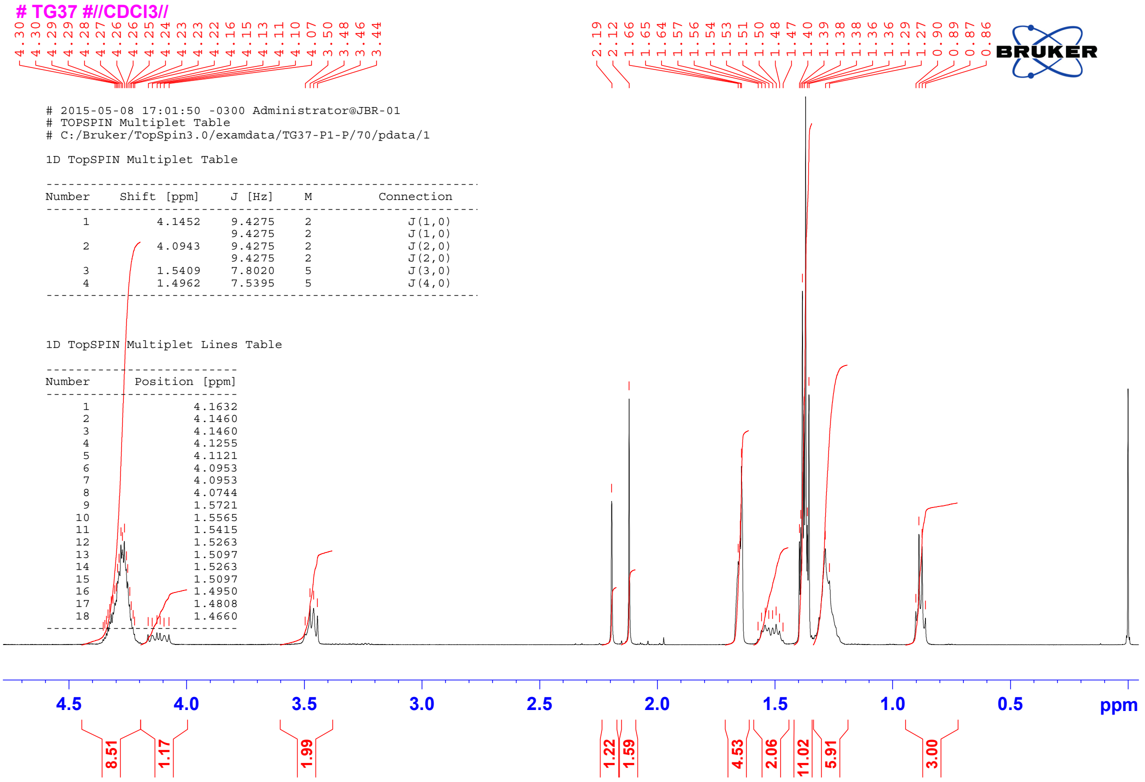

^{1}H NMR spectrum of compound **78**.

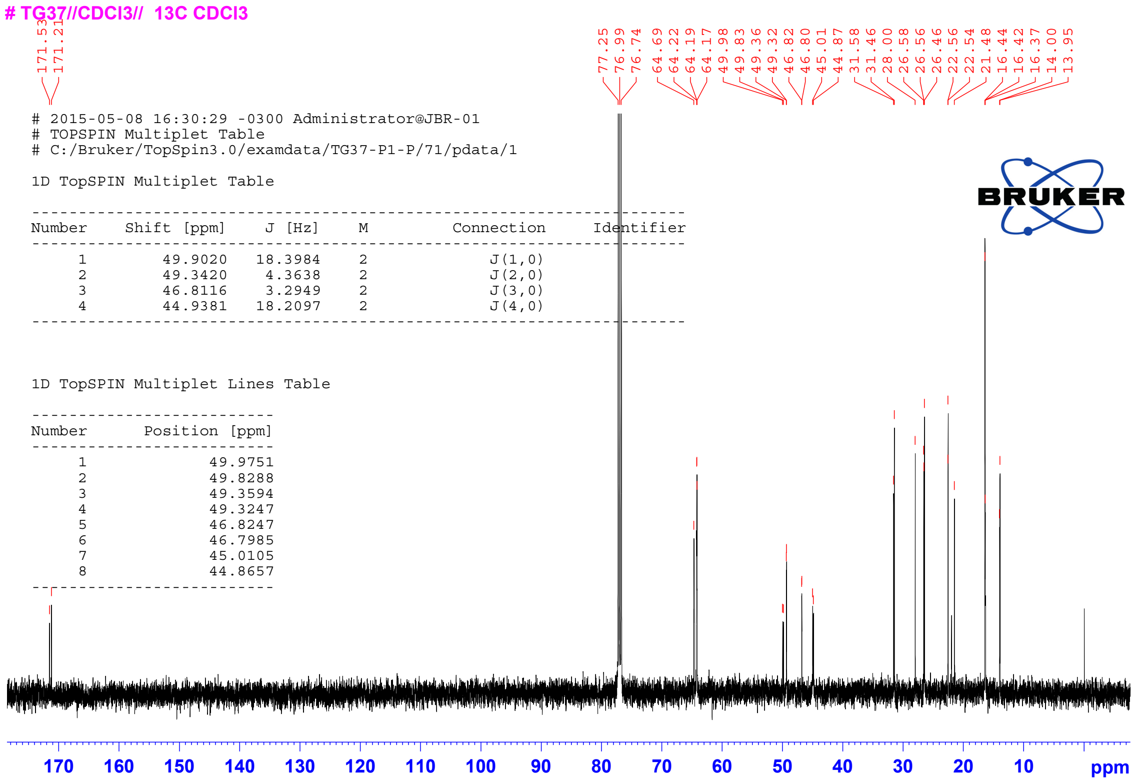

^{13}C NMR spectrum of compound **78**.

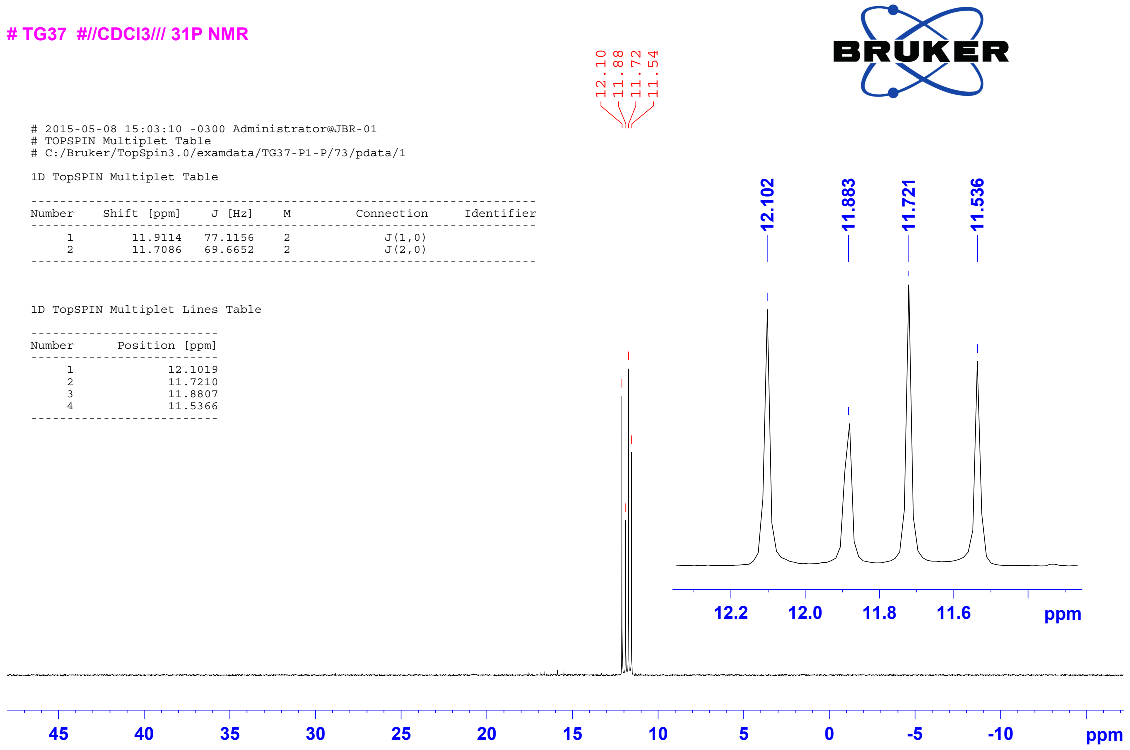

^{31}P NMR spectrum of compound **78**.

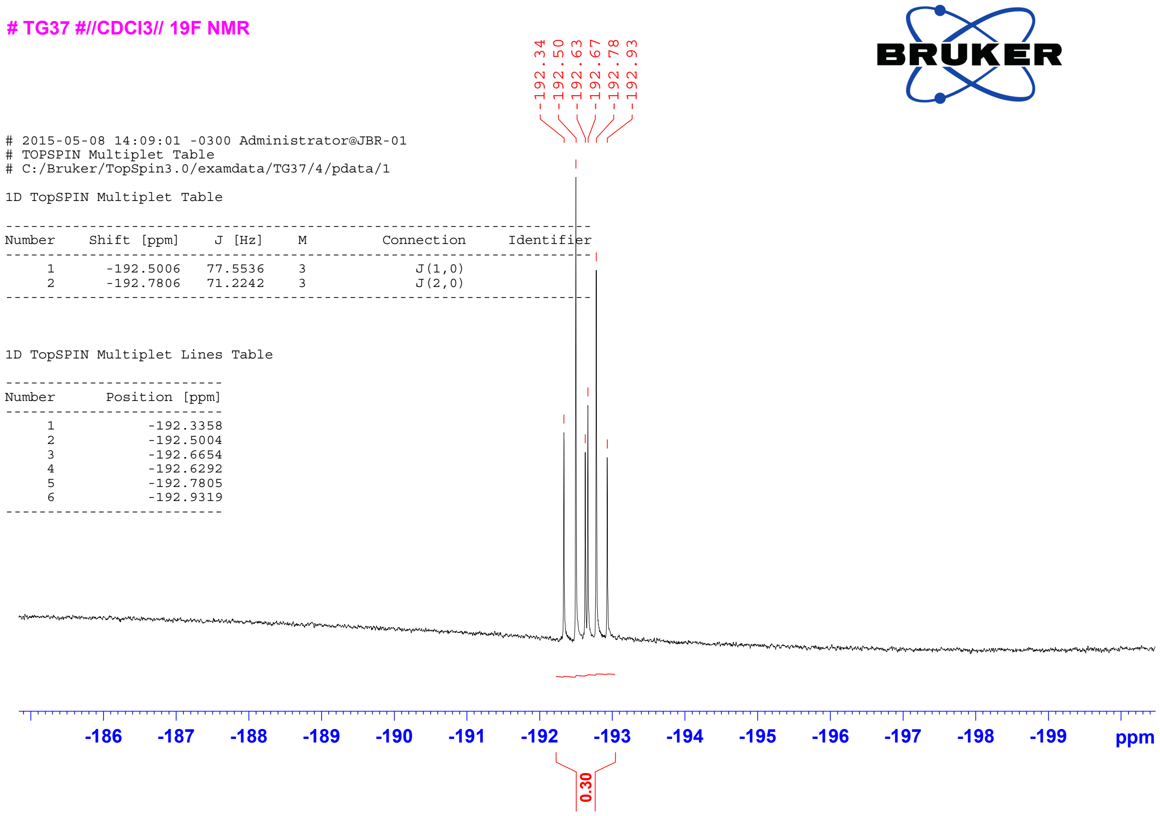

^{19}F NMR spectrum of compound **78**.

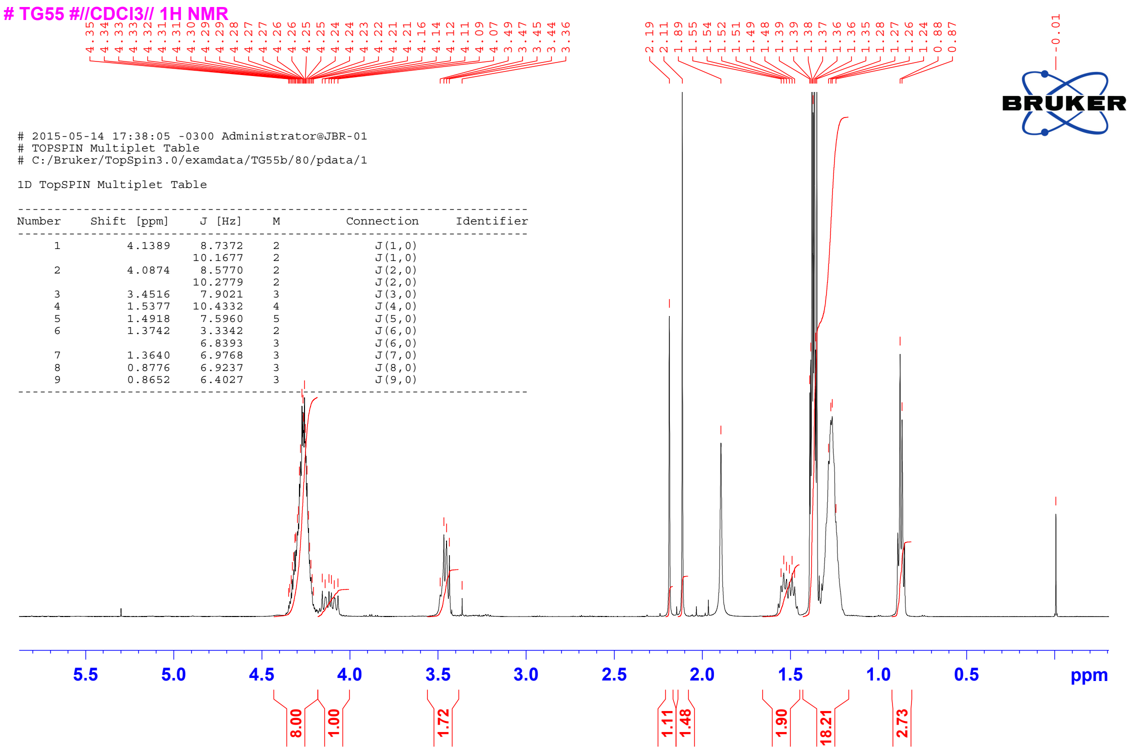

^{1}H NMR spectrum of compound **79**.

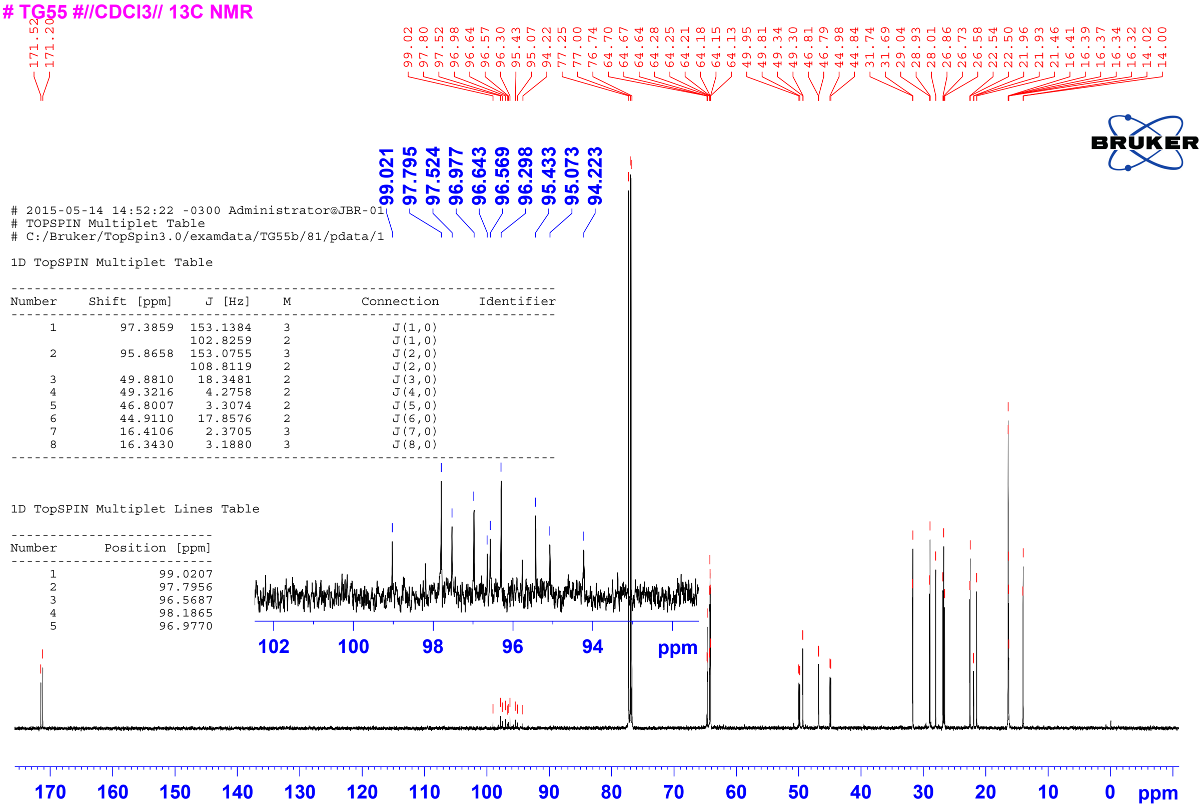

^{13}C NMR spectrum of compound **79**.

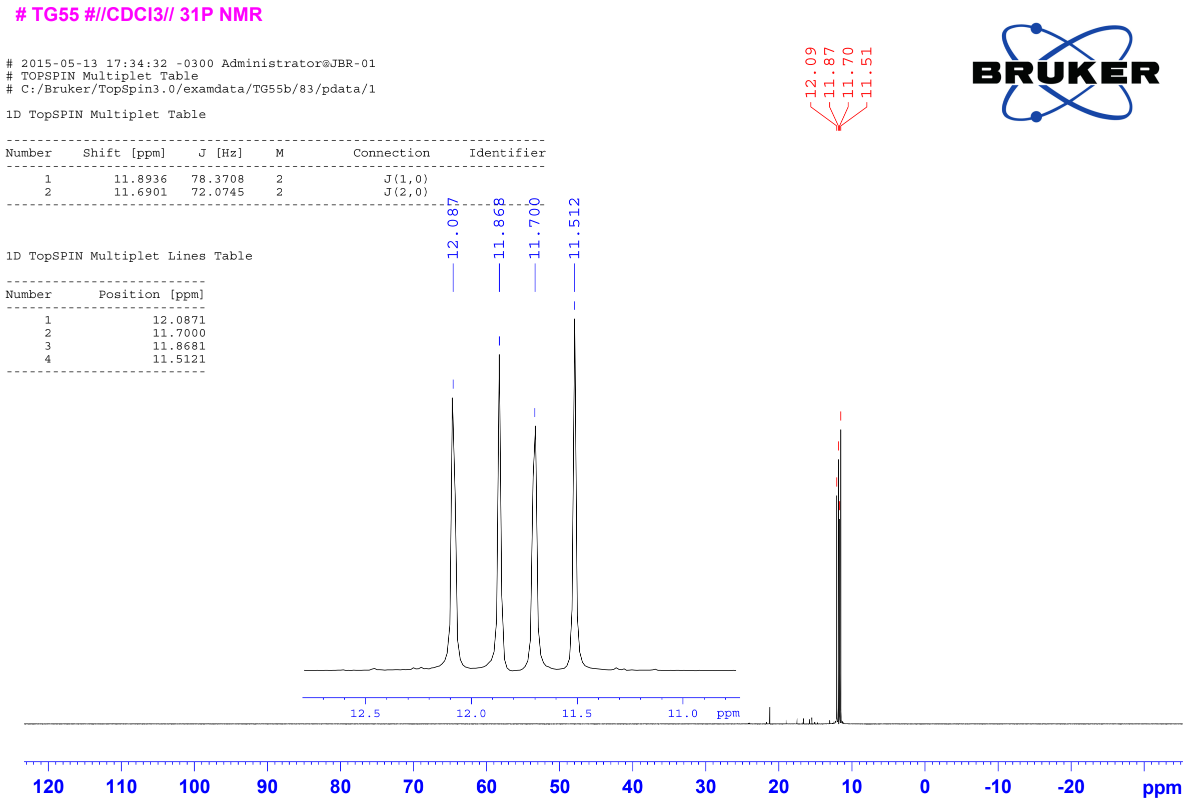

^{31}P NMR spectrum of compound **79**.

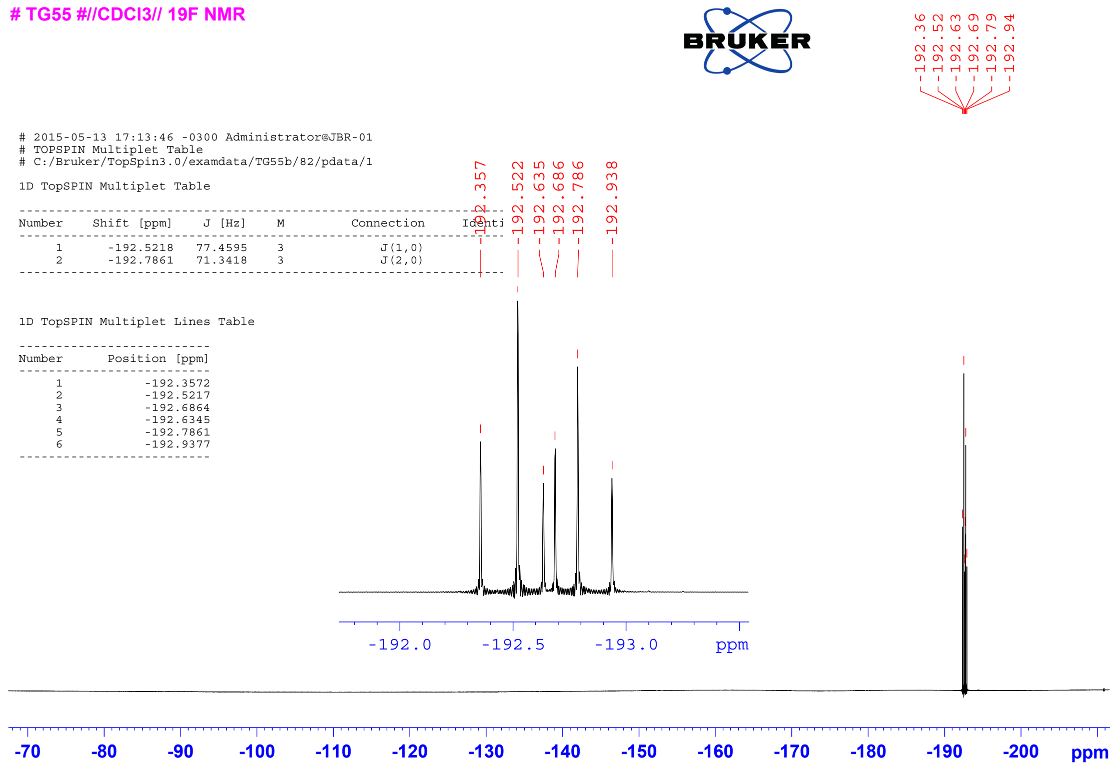

^{19}F NMR spectrum of compound **79**.

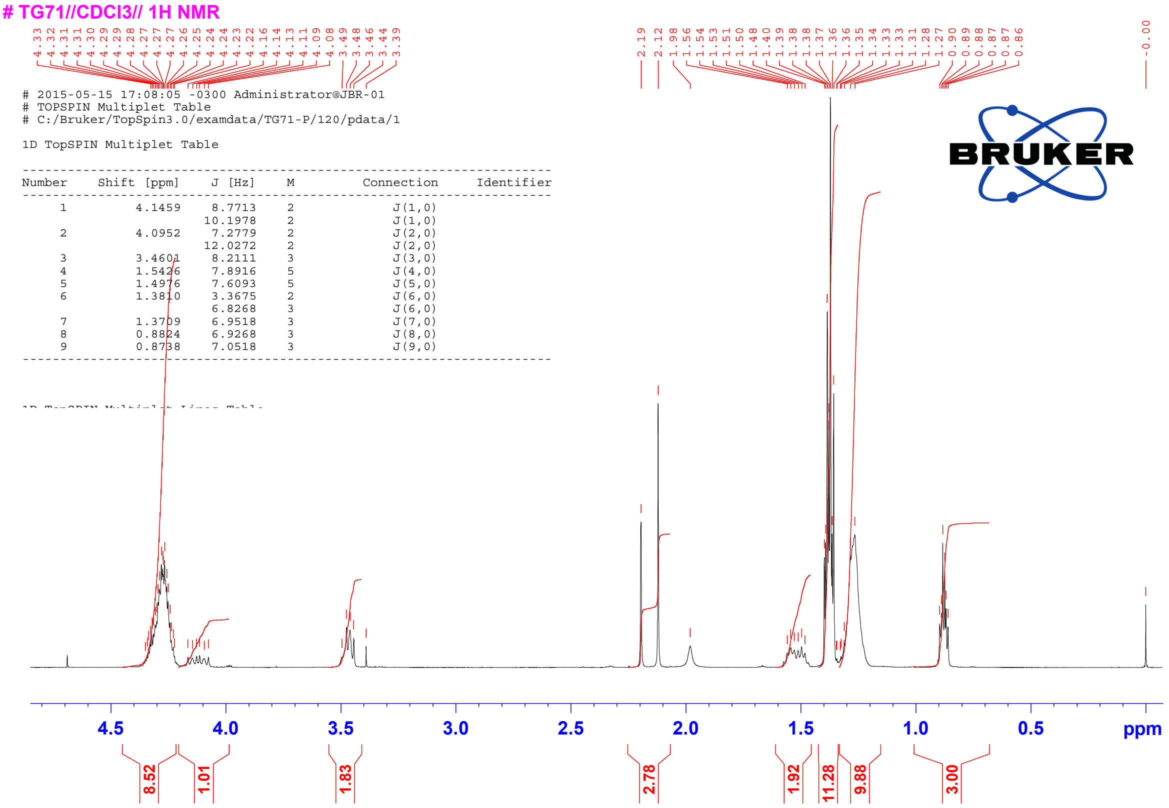

^{1}H NMR spectrum of compound **80**.

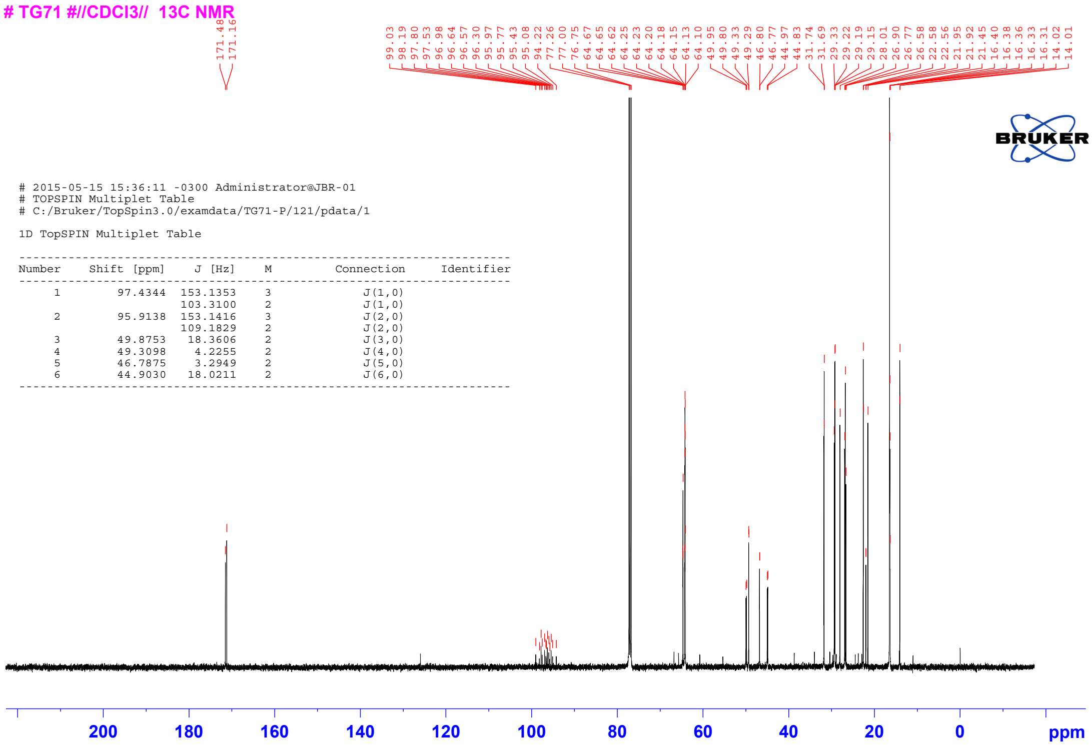

^{13}C NMR spectrum of compound **80**.

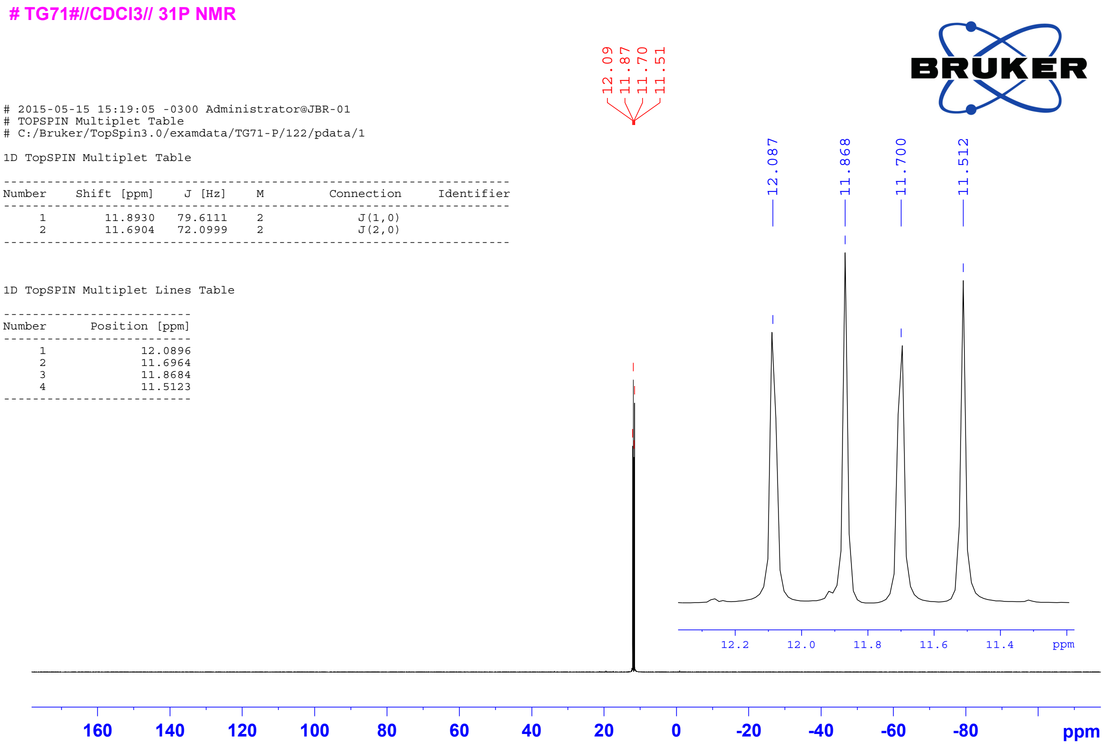

^{31}P NMR spectrum of compound **80**.

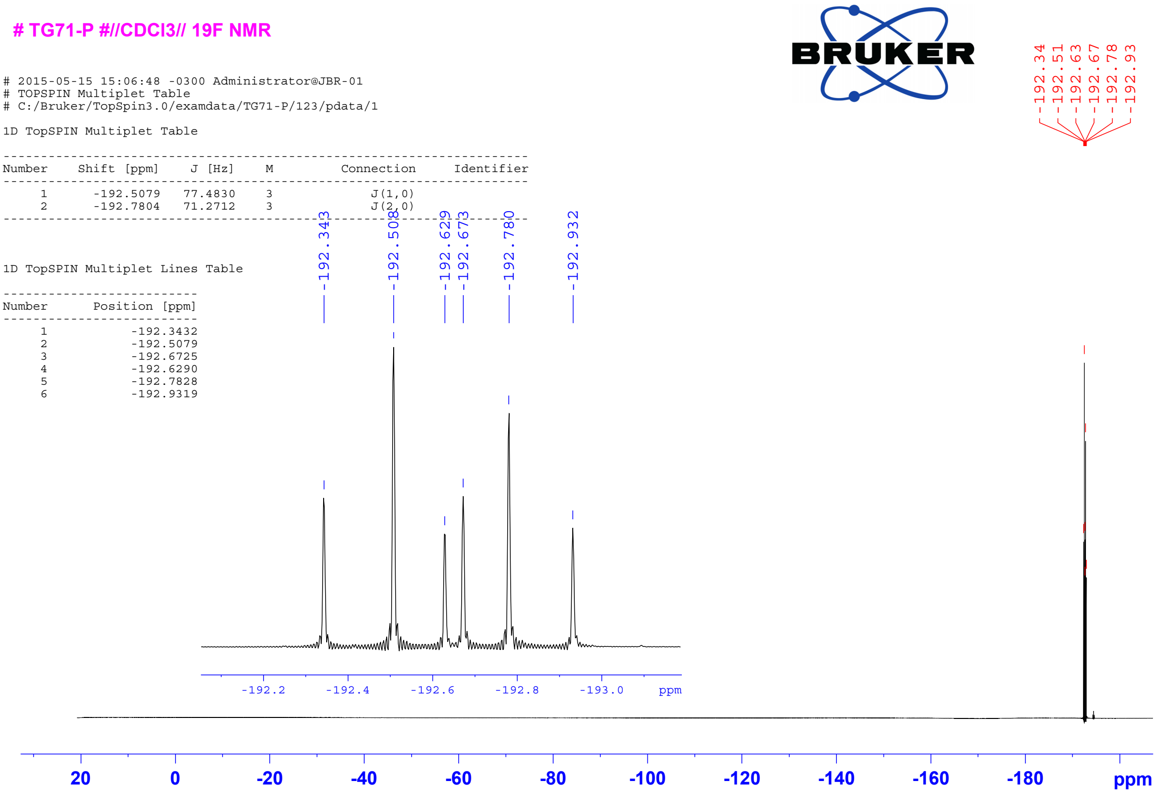

^{19}F NMR spectrum of compound **80**.

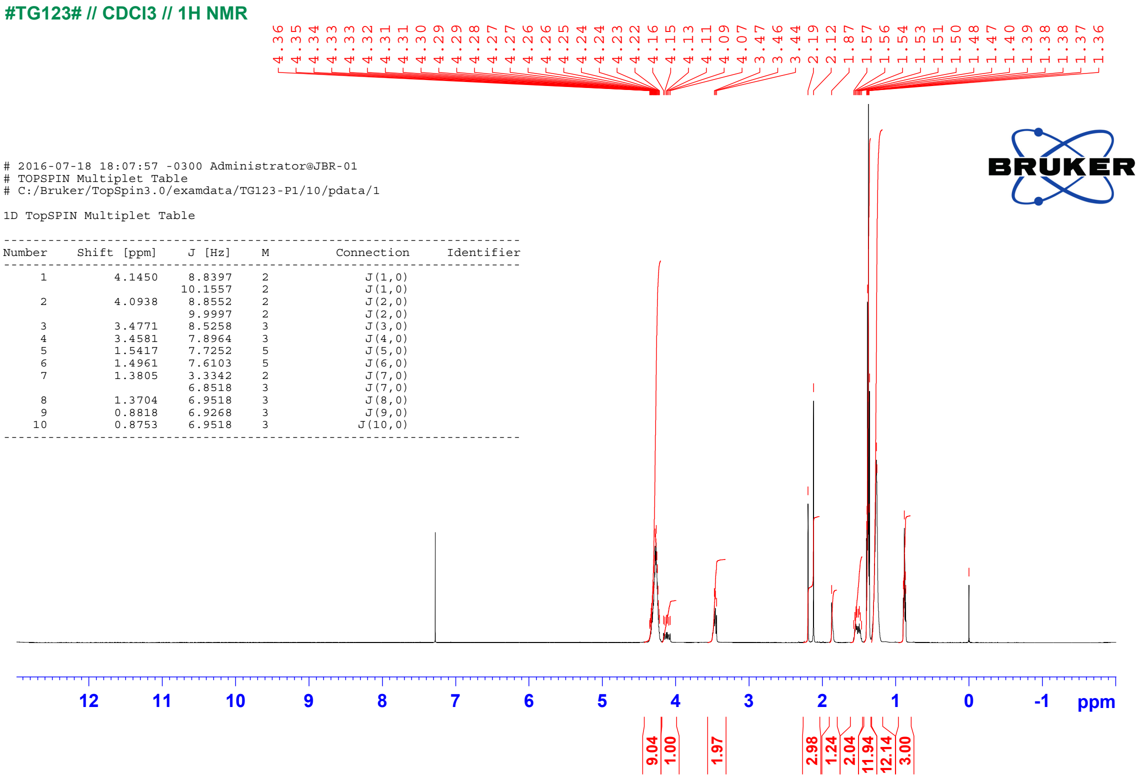

^{1}H NMR spectrum of compound **81**.

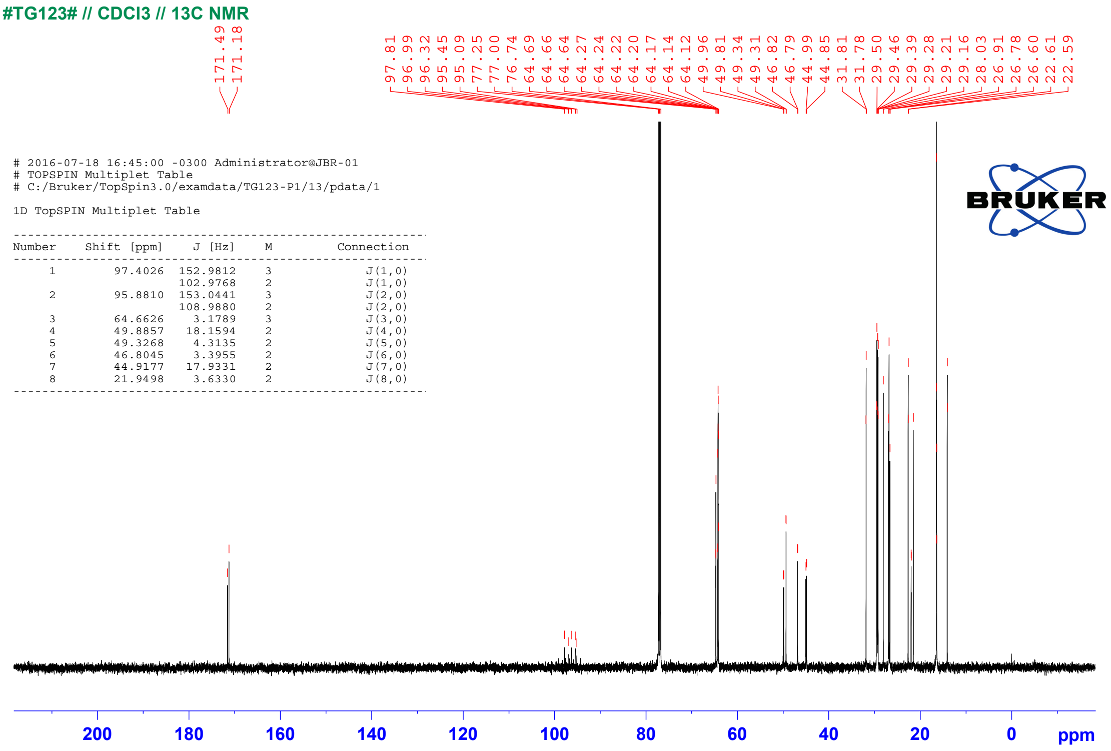

^{13}C NMR spectrum of compound **81**.

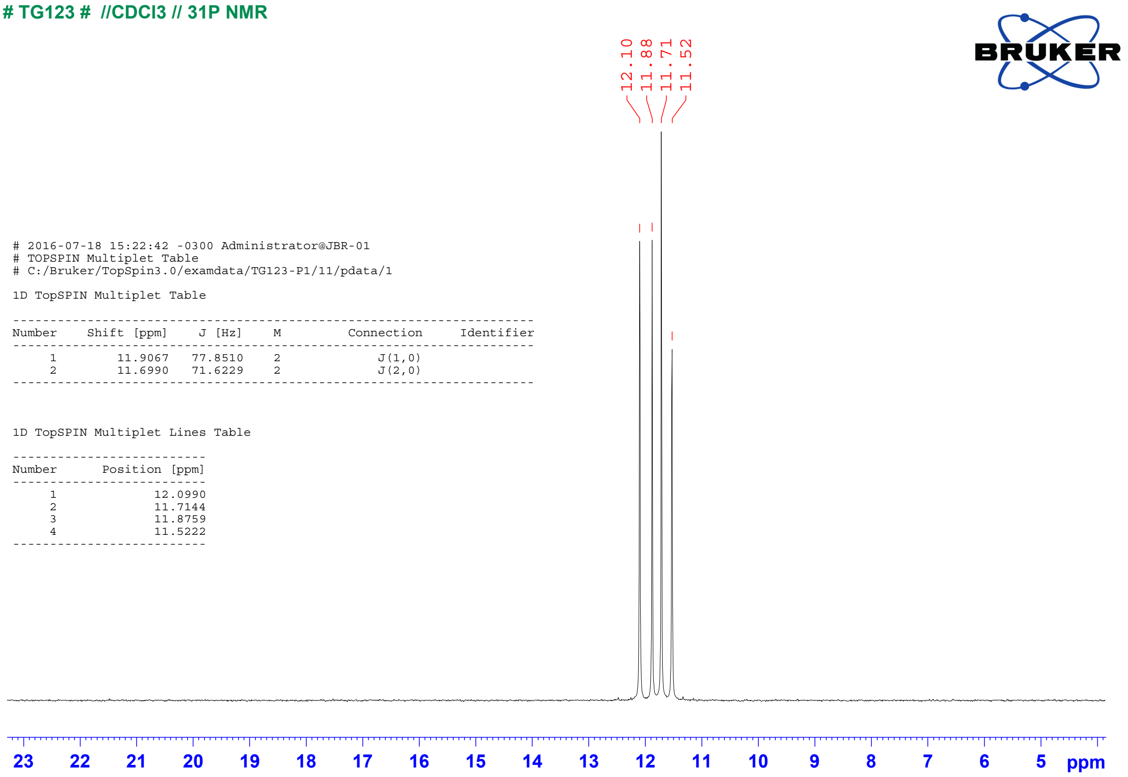

^{31}P NMR spectrum of compound **81**.

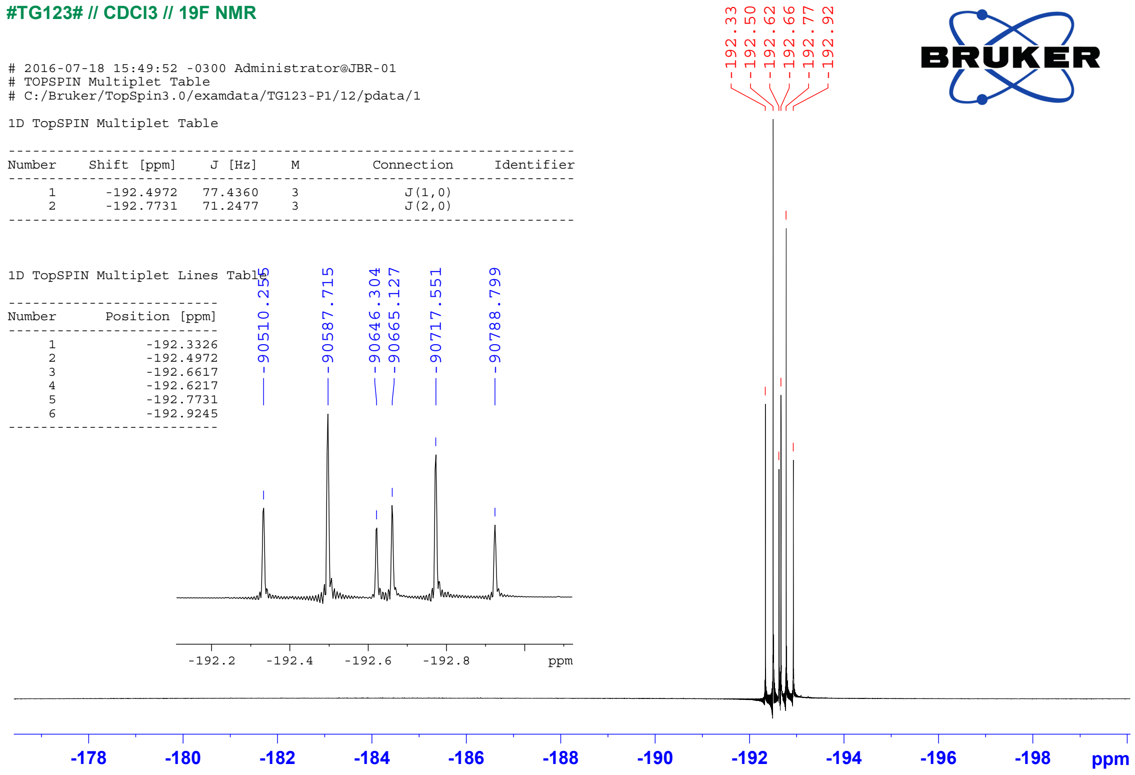

^{19}F NMR spectrum of compound **81**.

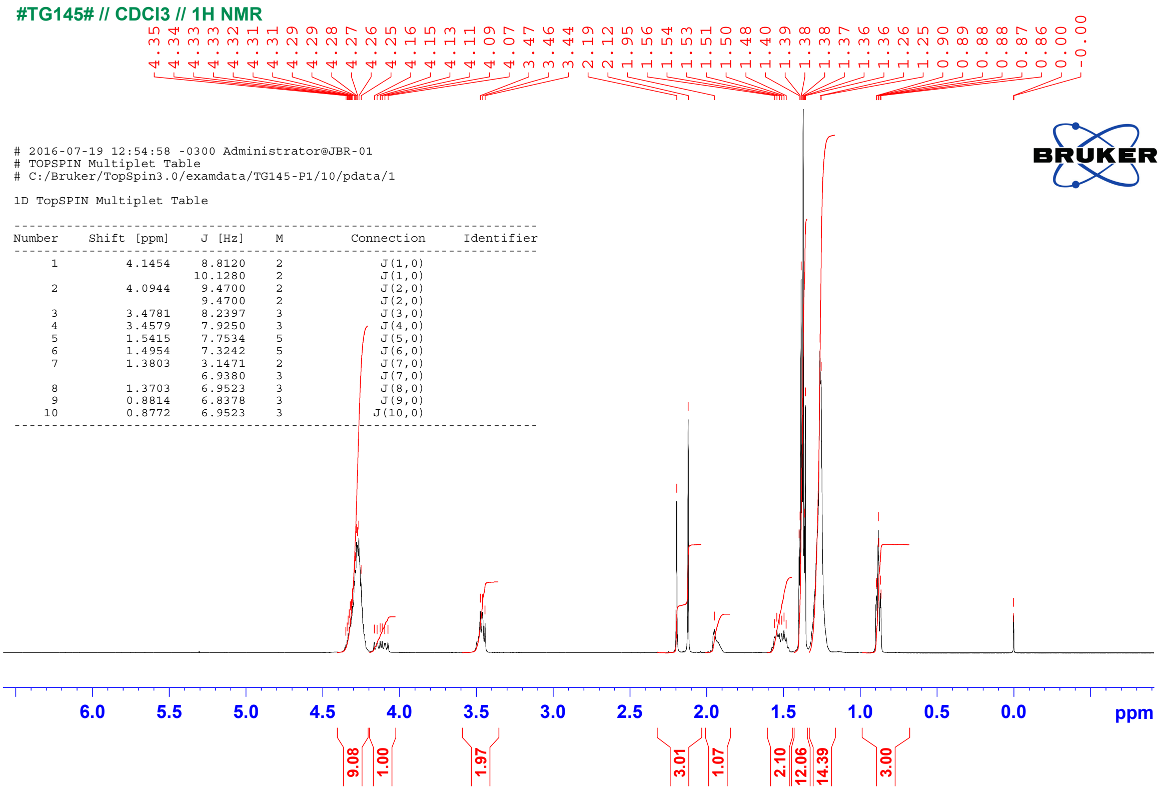

^{1}H NMR spectrum of compound **82**.

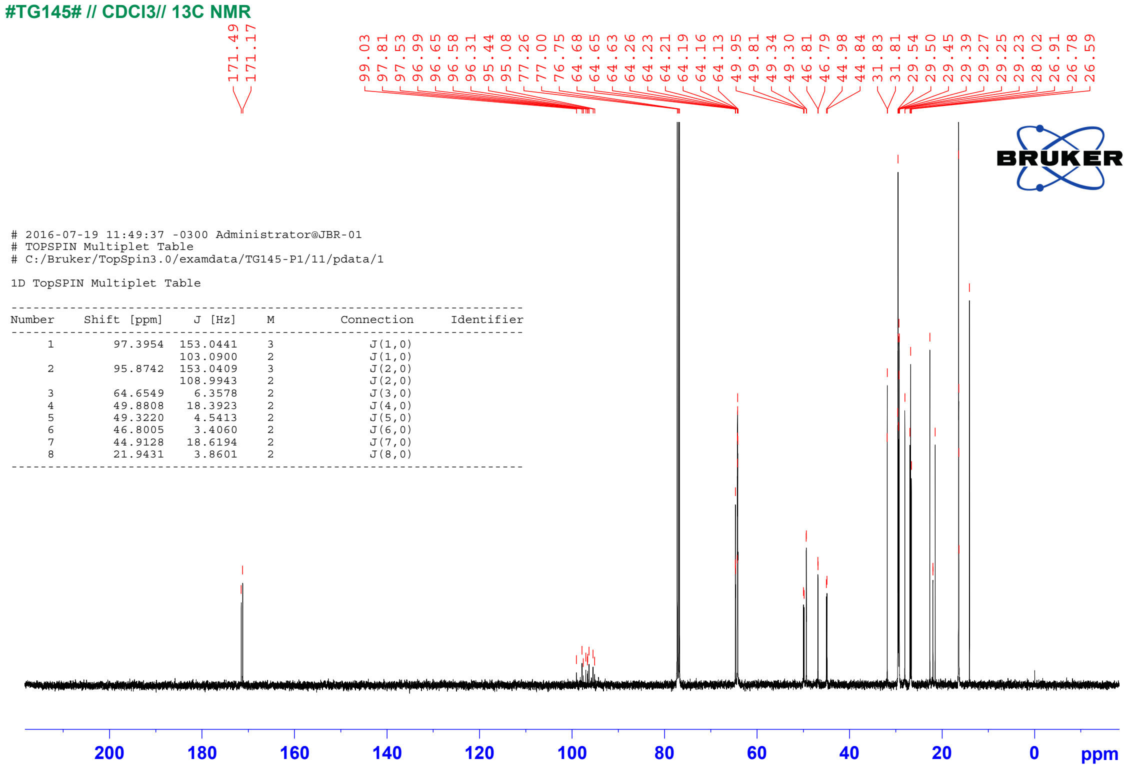

^{31}P NMR spectrum of compound **82**.

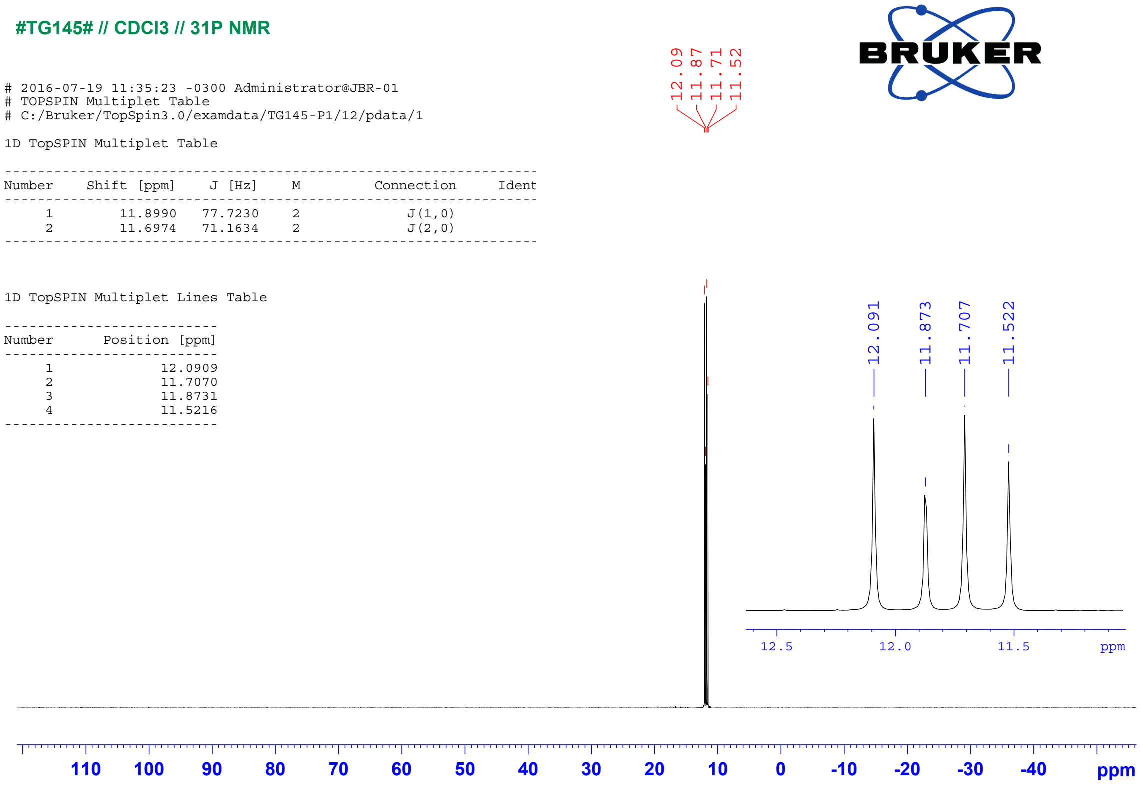

^{31}P NMR spectrum of compound **82**.

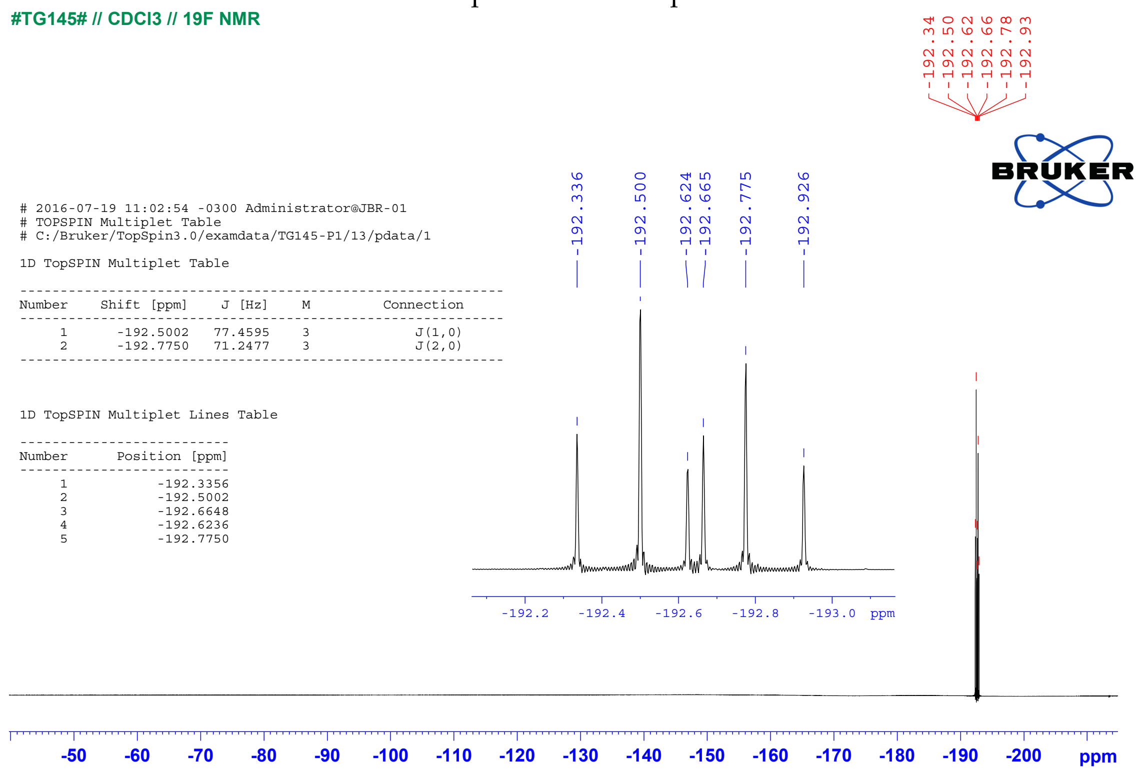

^{19}F NMR spectrum of compound **82**.

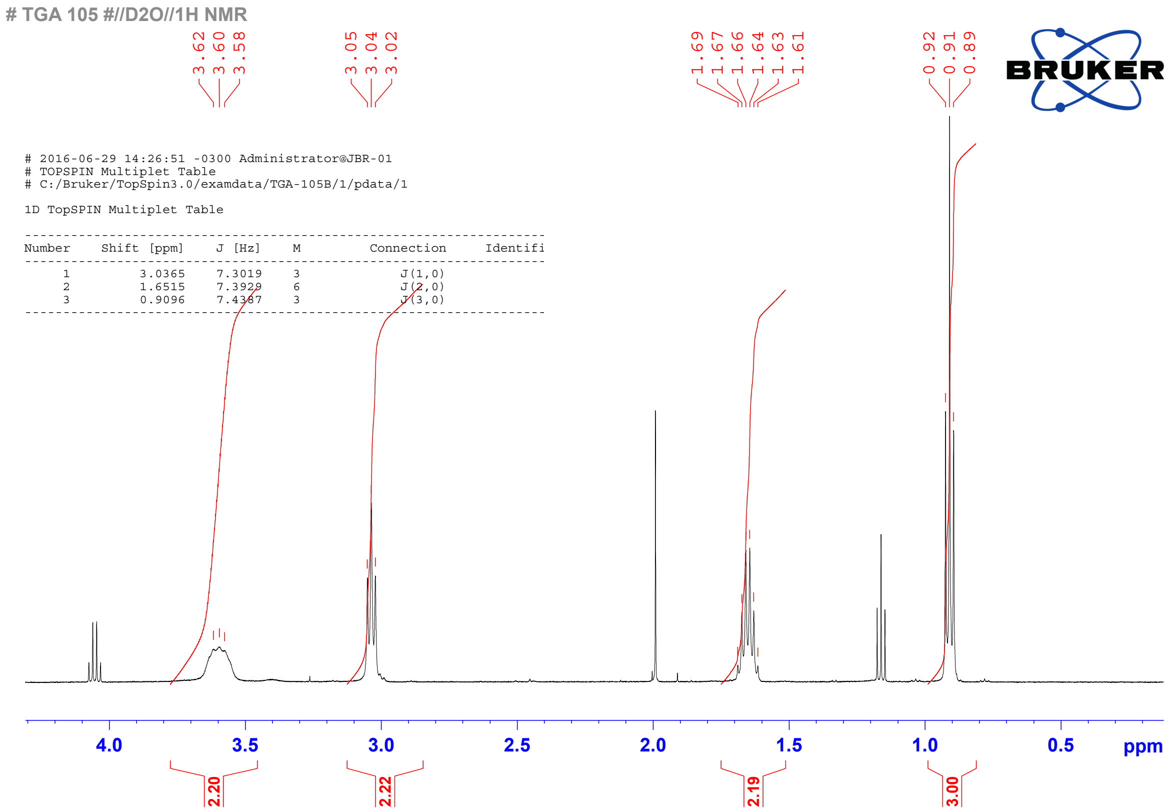

^{1}H NMR spectrum of compound **54**.

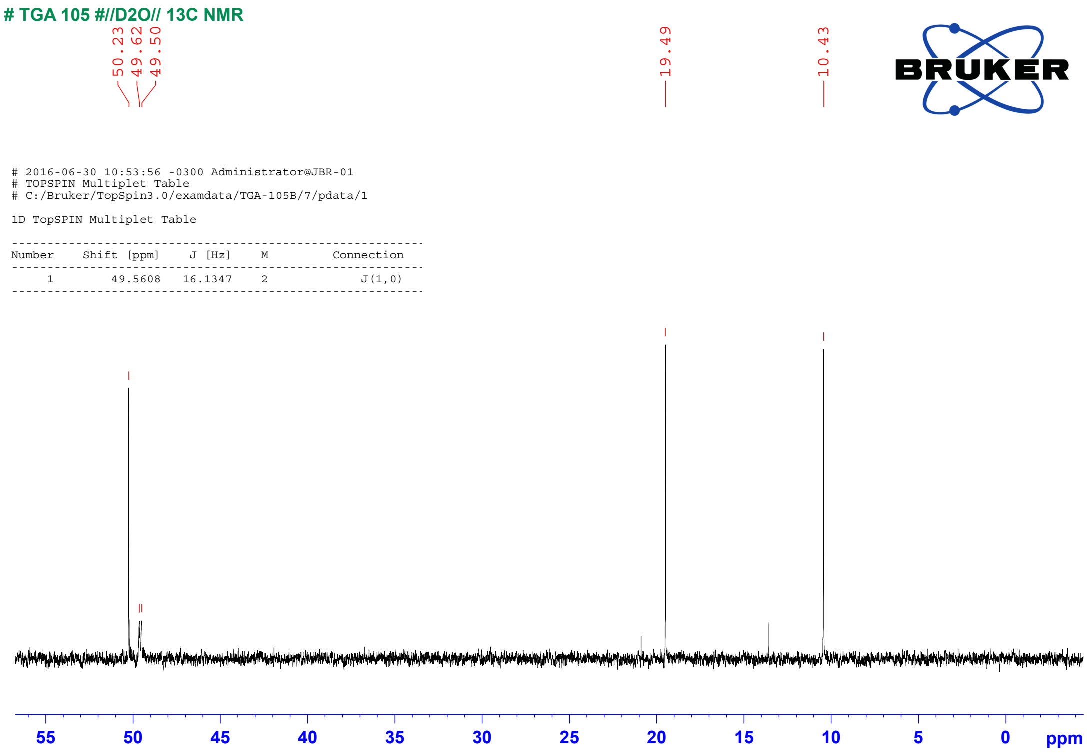

^{13}C NMR spectrum of compound **54**.

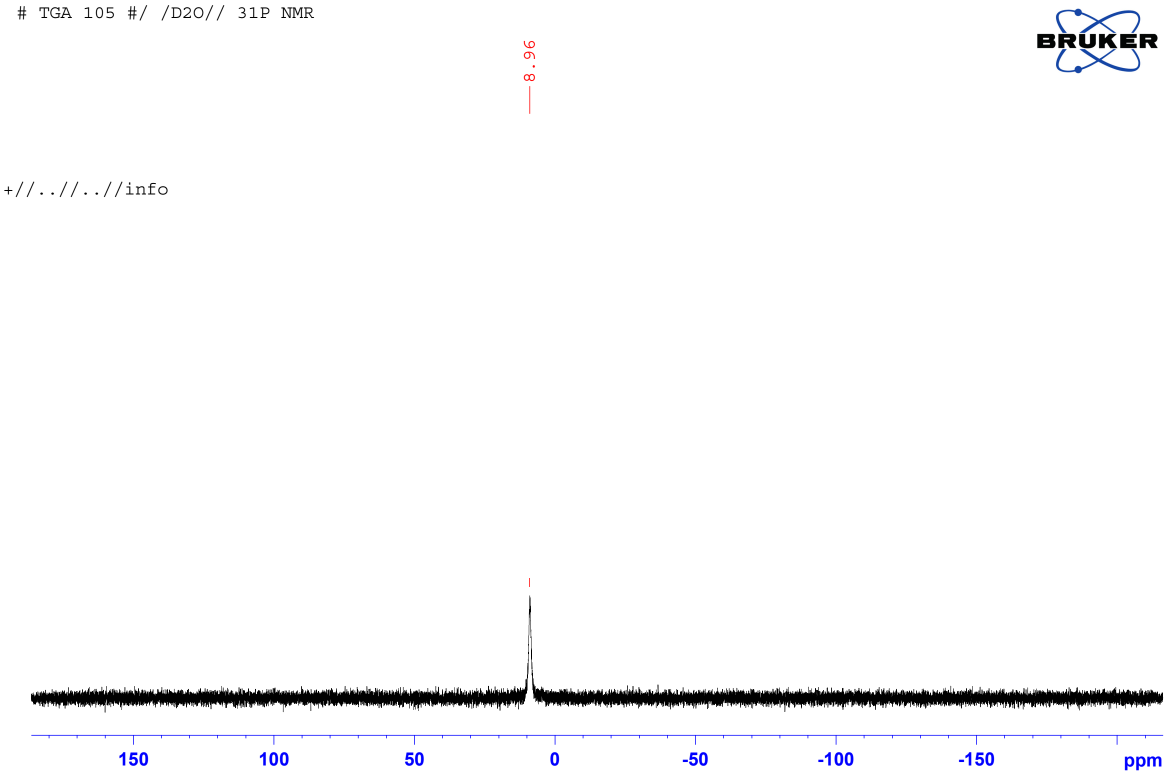

^{31}P NMR spectrum of compound **54**.

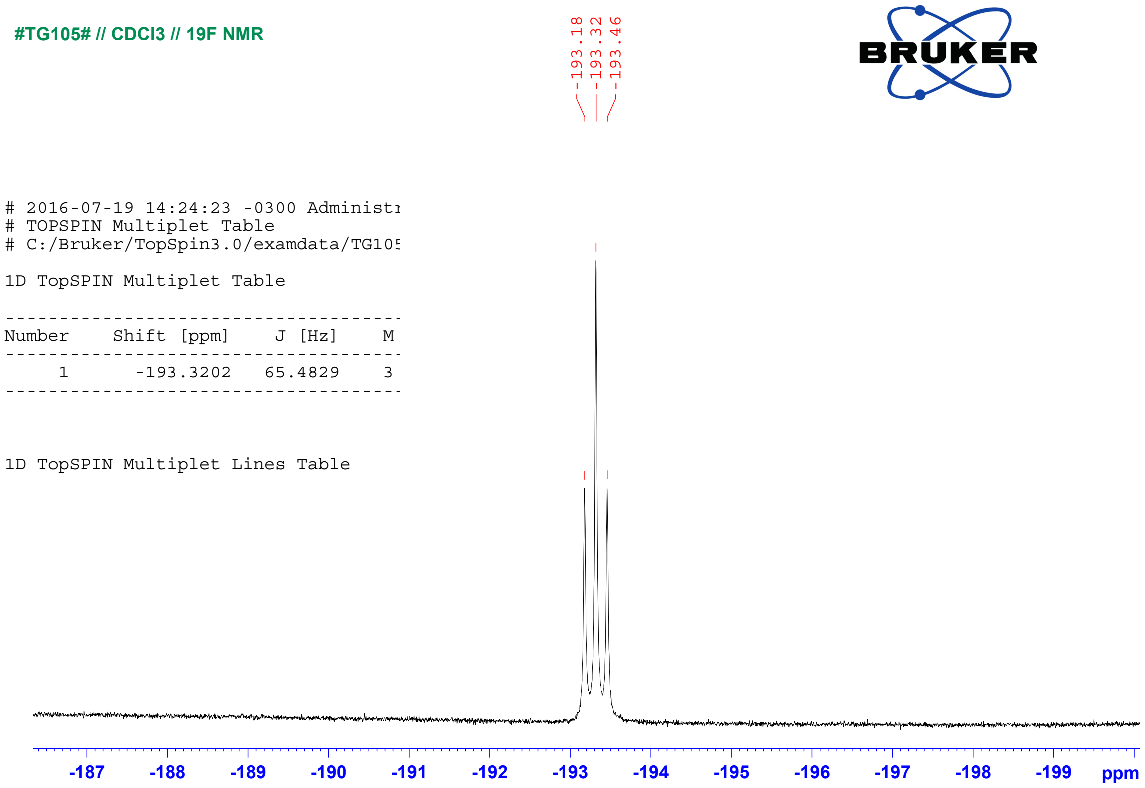

^{19}F NMR spectrum of compound **54**.

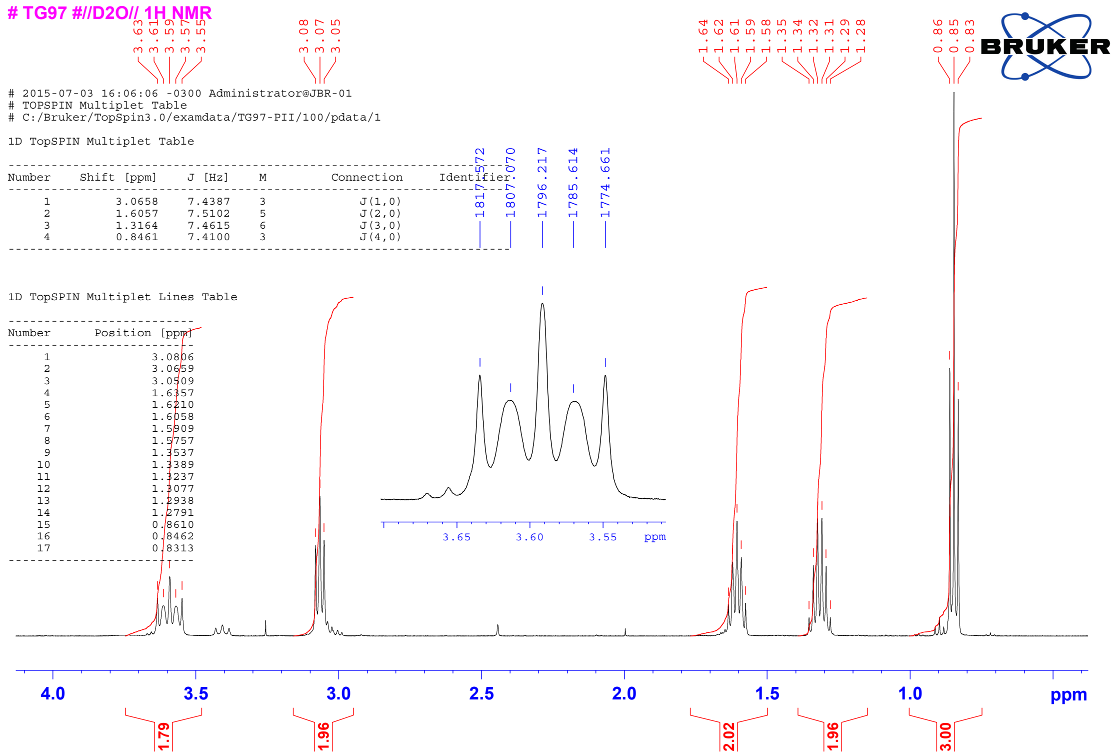

^{1}H NMR spectrum of compound **55**.

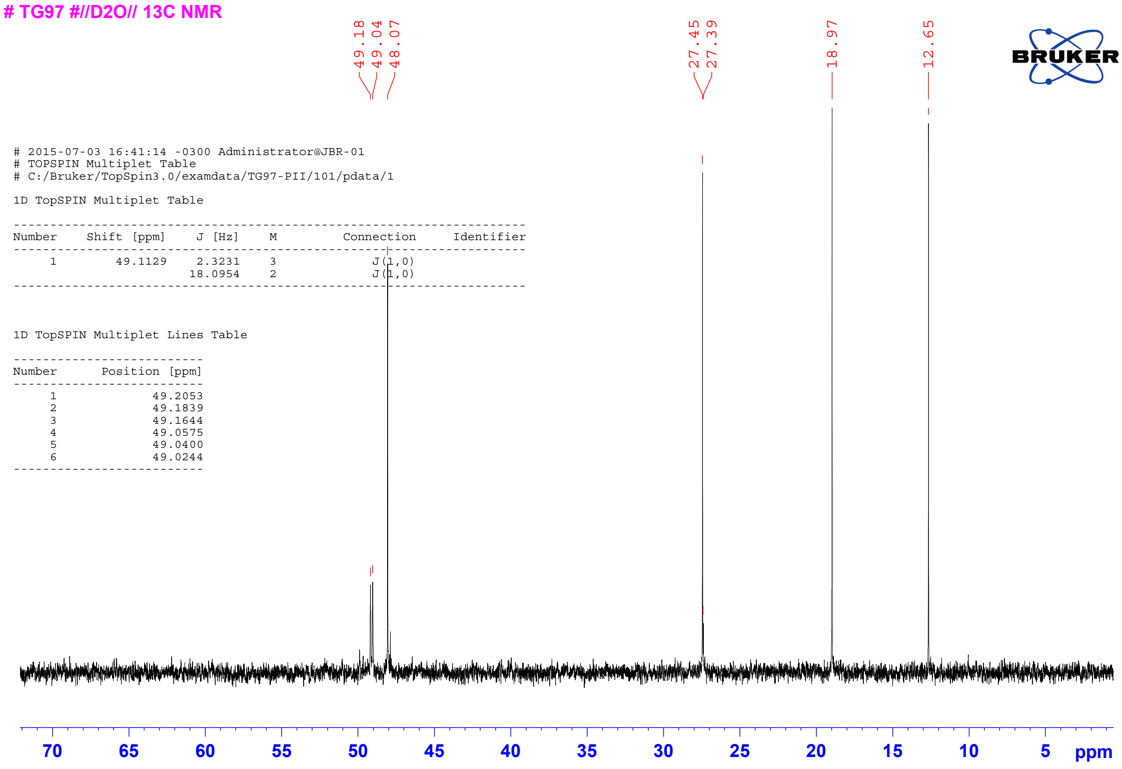

^{13}C NMR spectrum of compound **55**.

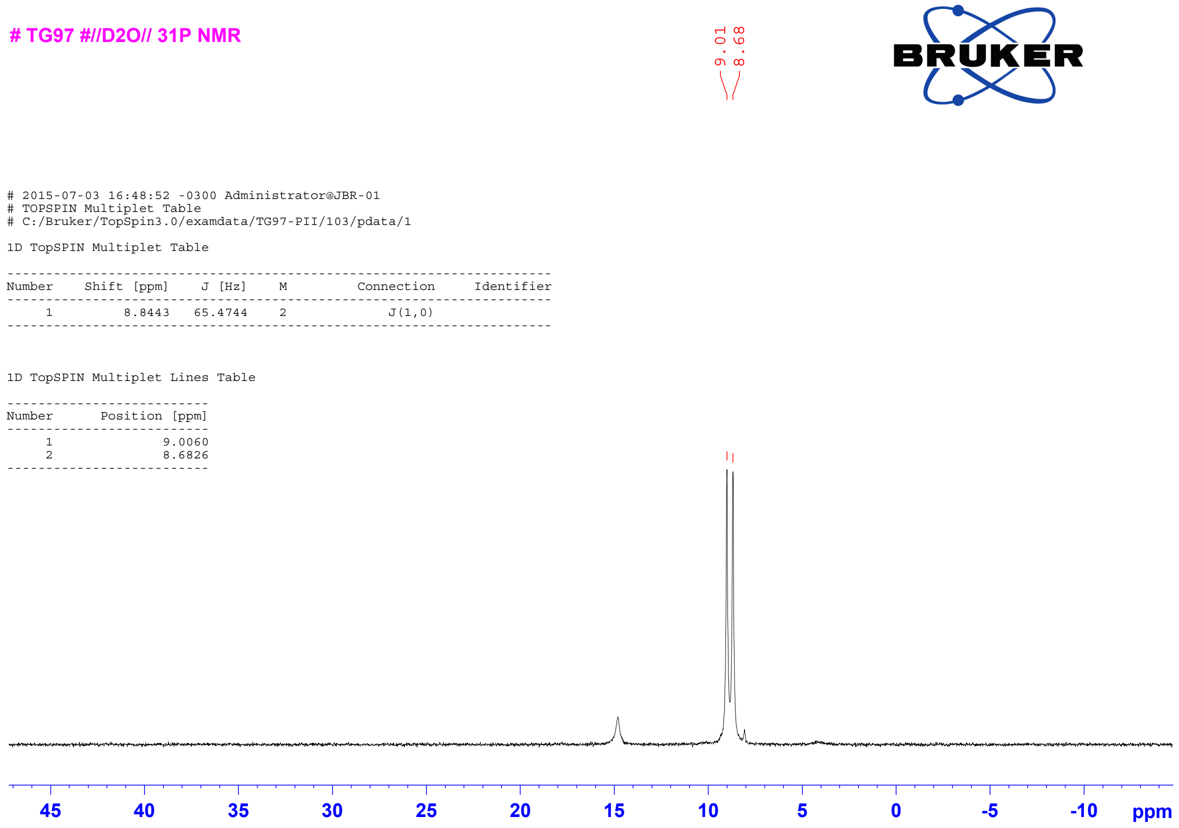

^{31}P NMR spectrum of compound **55**.

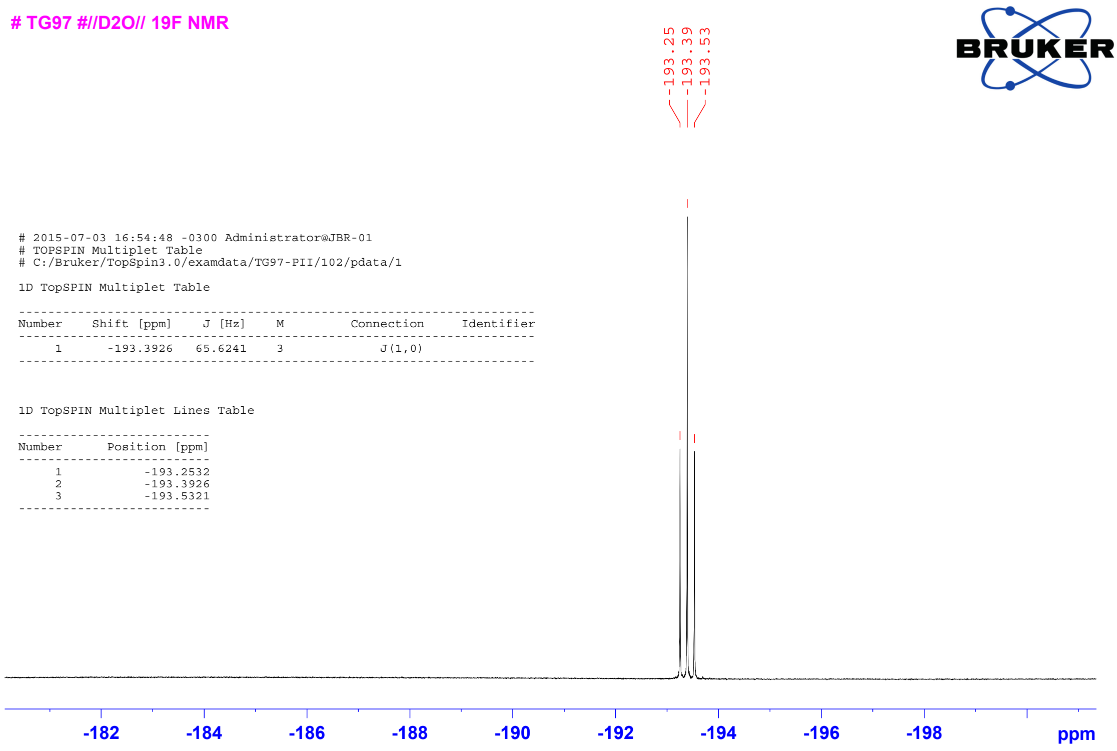

^{19}F NMR spectrum of compound **55**.

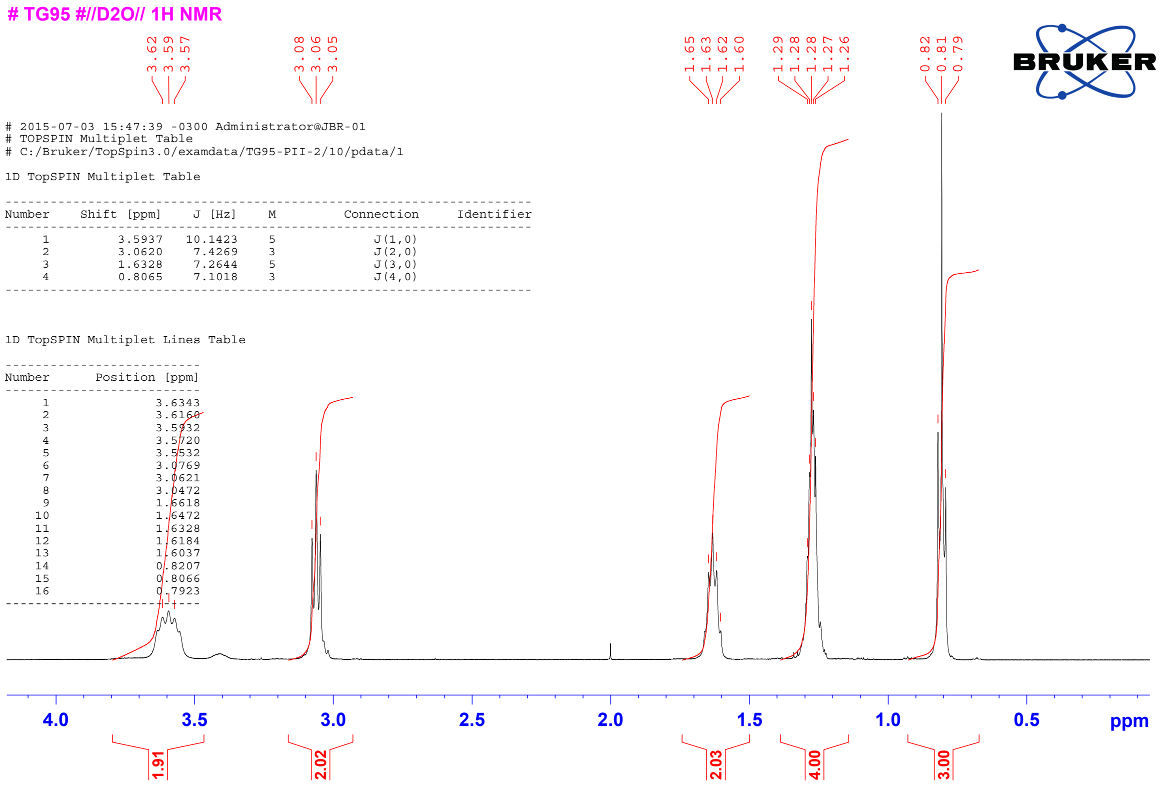

^{1}H NMR spectrum of compound **56**.

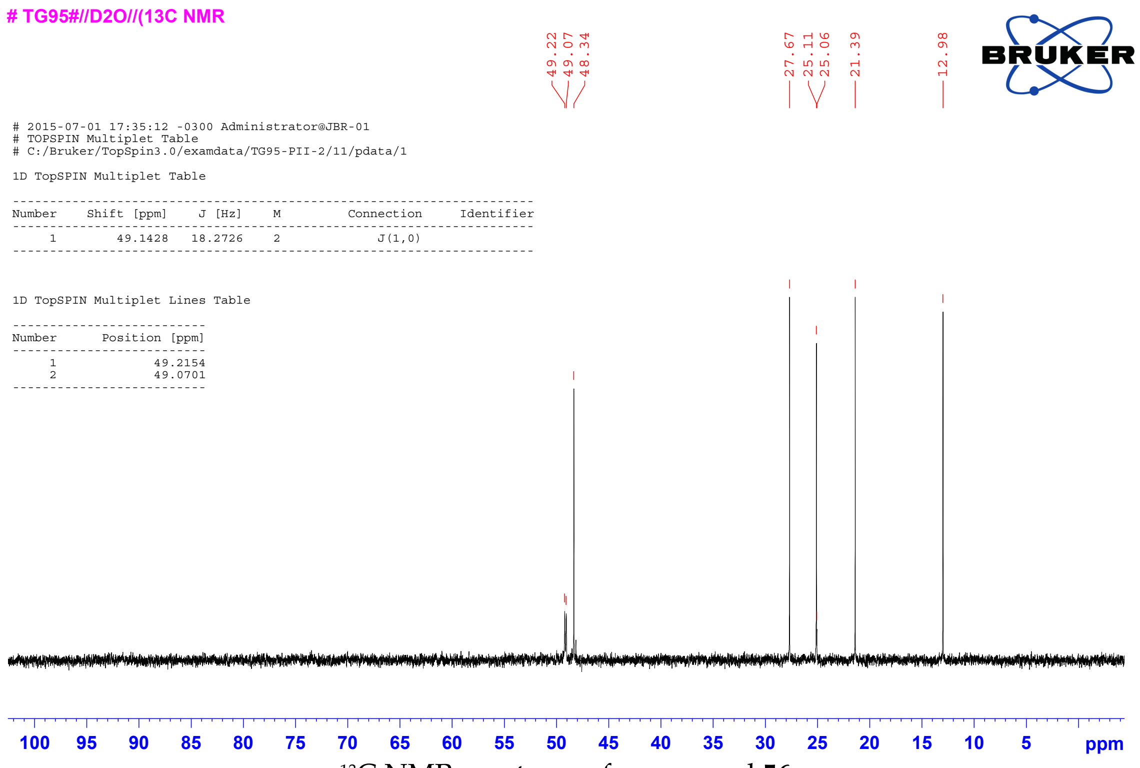

^{13}C NMR spectrum of compound **56**.

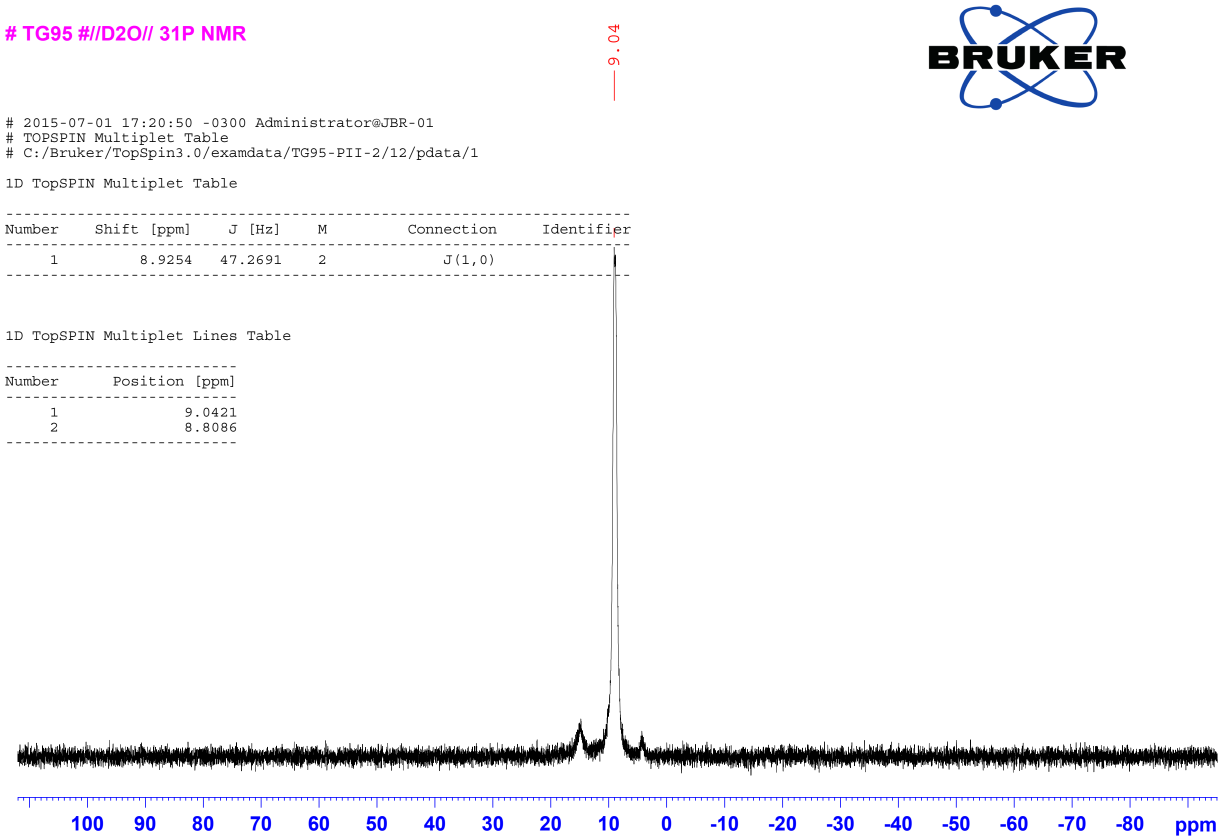

^{31}P NMR spectrum of compound **56**.

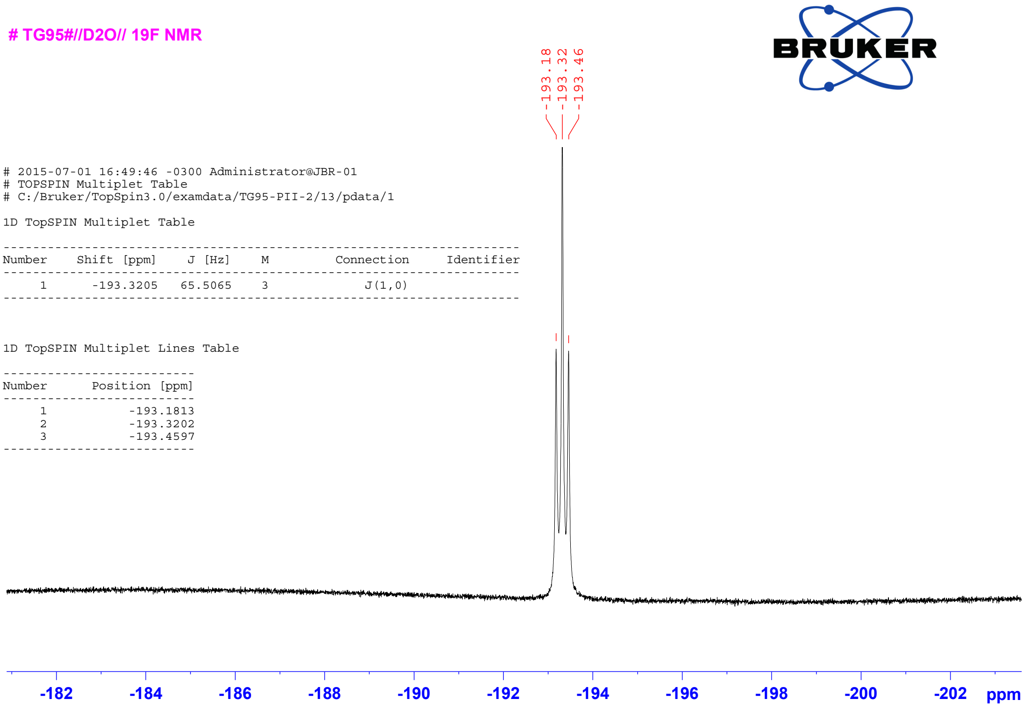

^{19}F NMR spectrum of compound **56**.

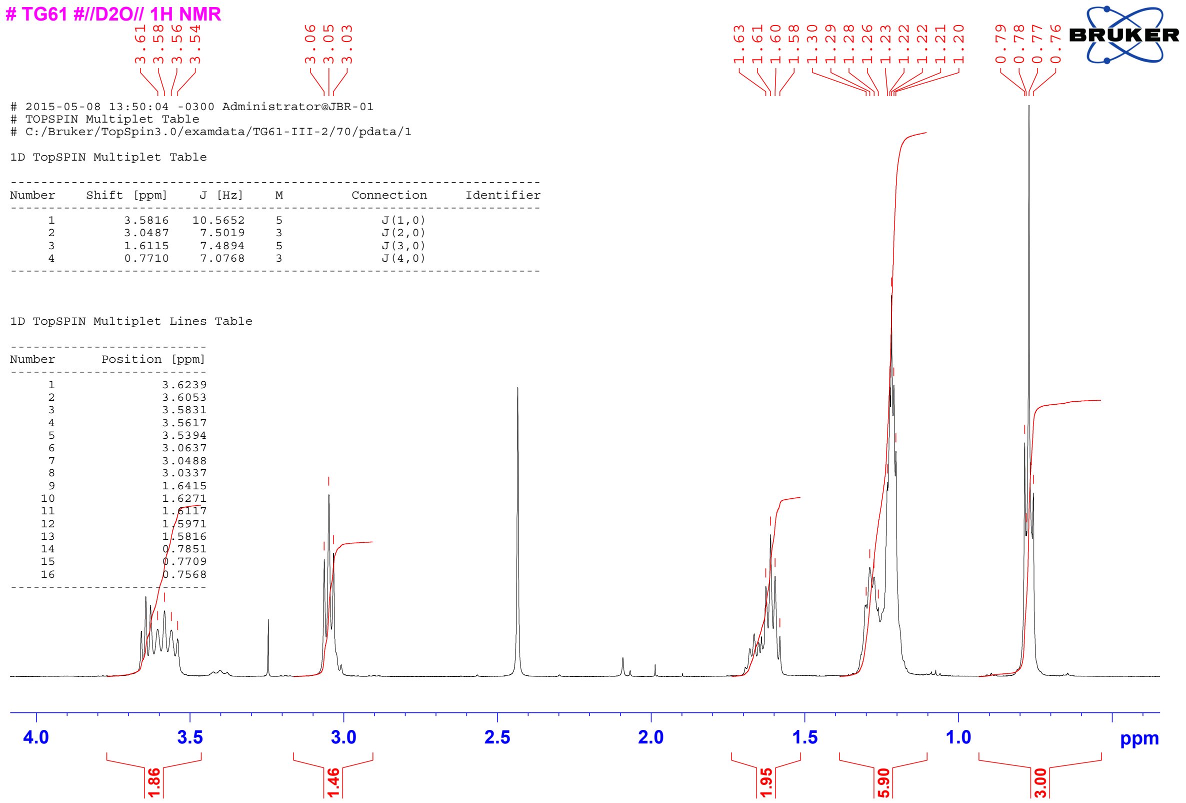

^{1}H NMR spectrum of compound **57**.

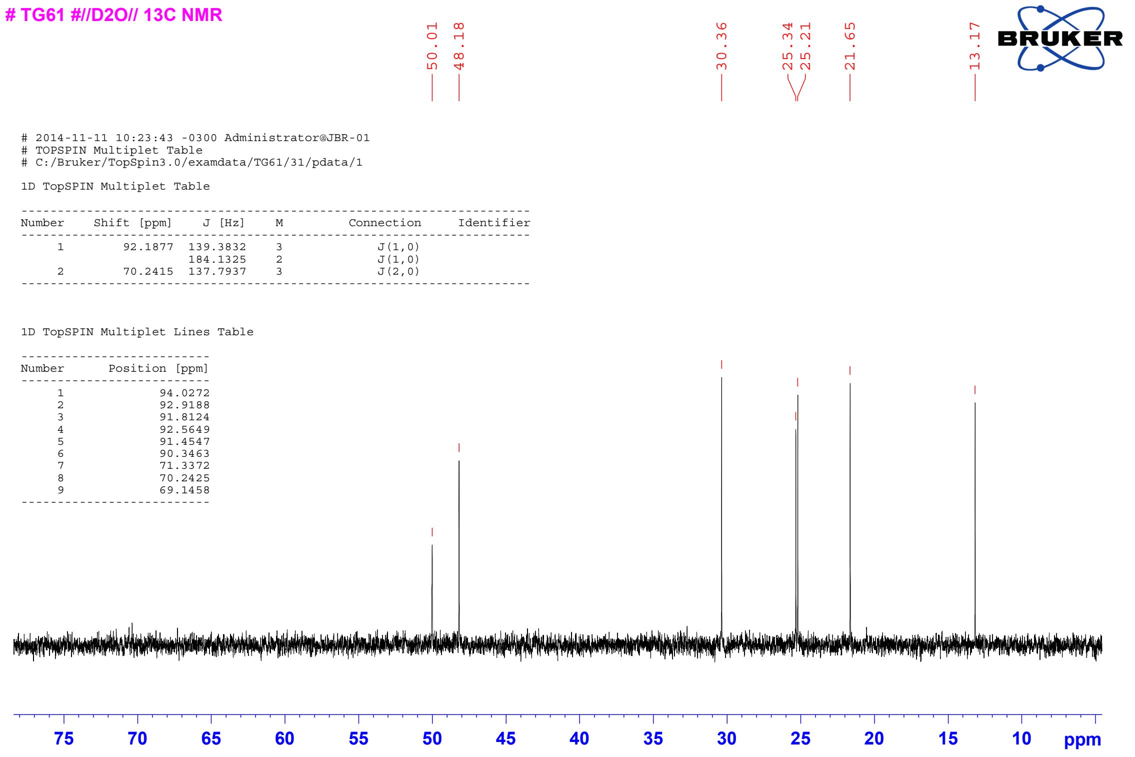

^{13}C NMR spectrum of compound **57**.

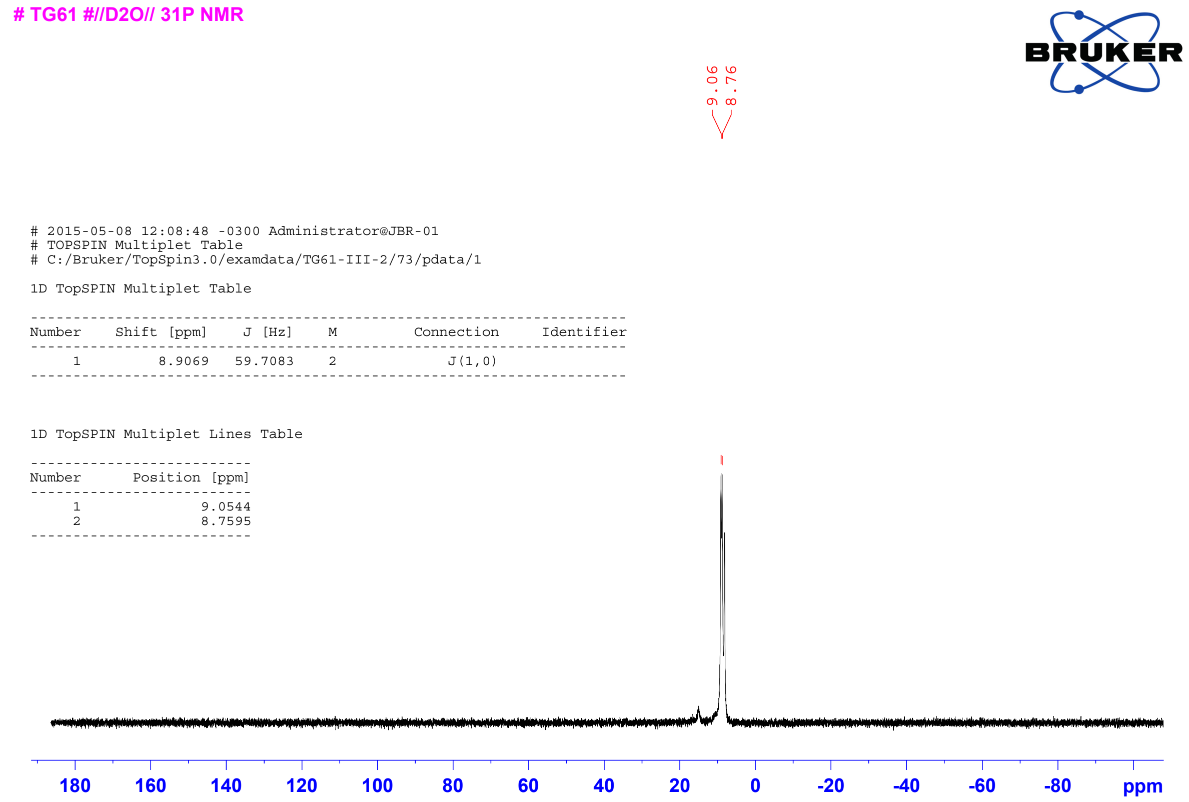

^{31}P NMR spectrum of compound **57**.

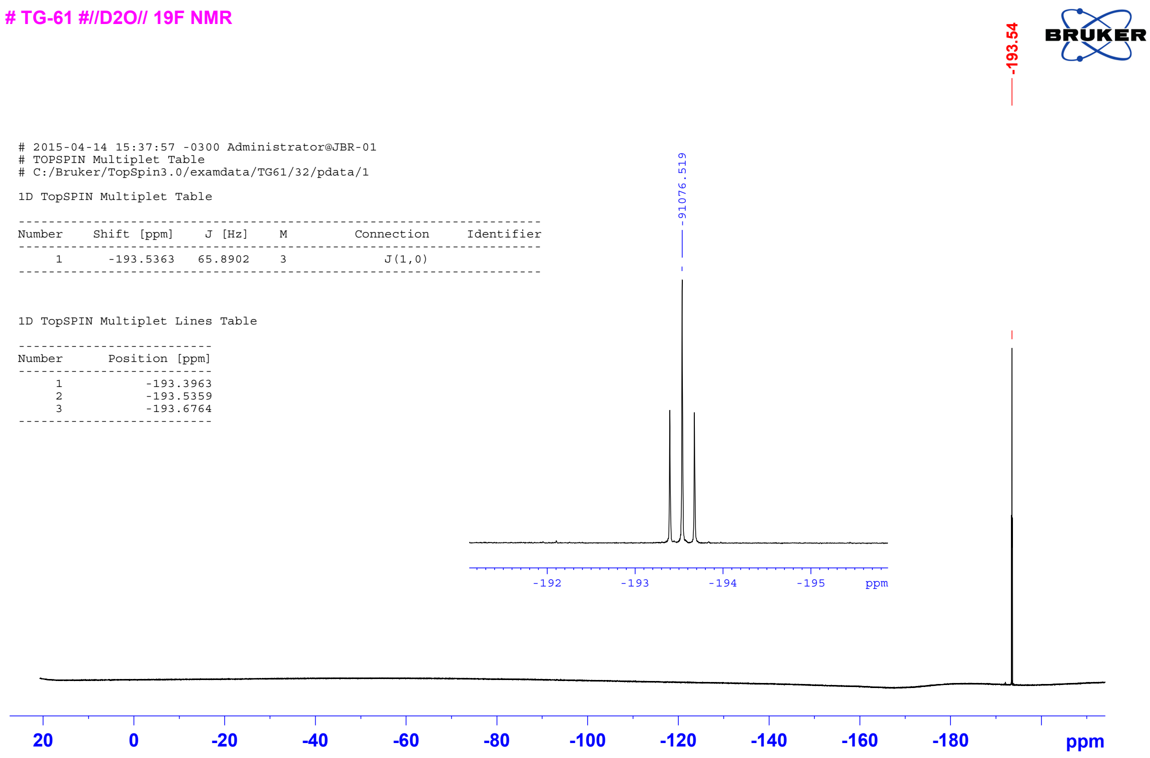

^{19}F NMR spectrum of compound **57**.

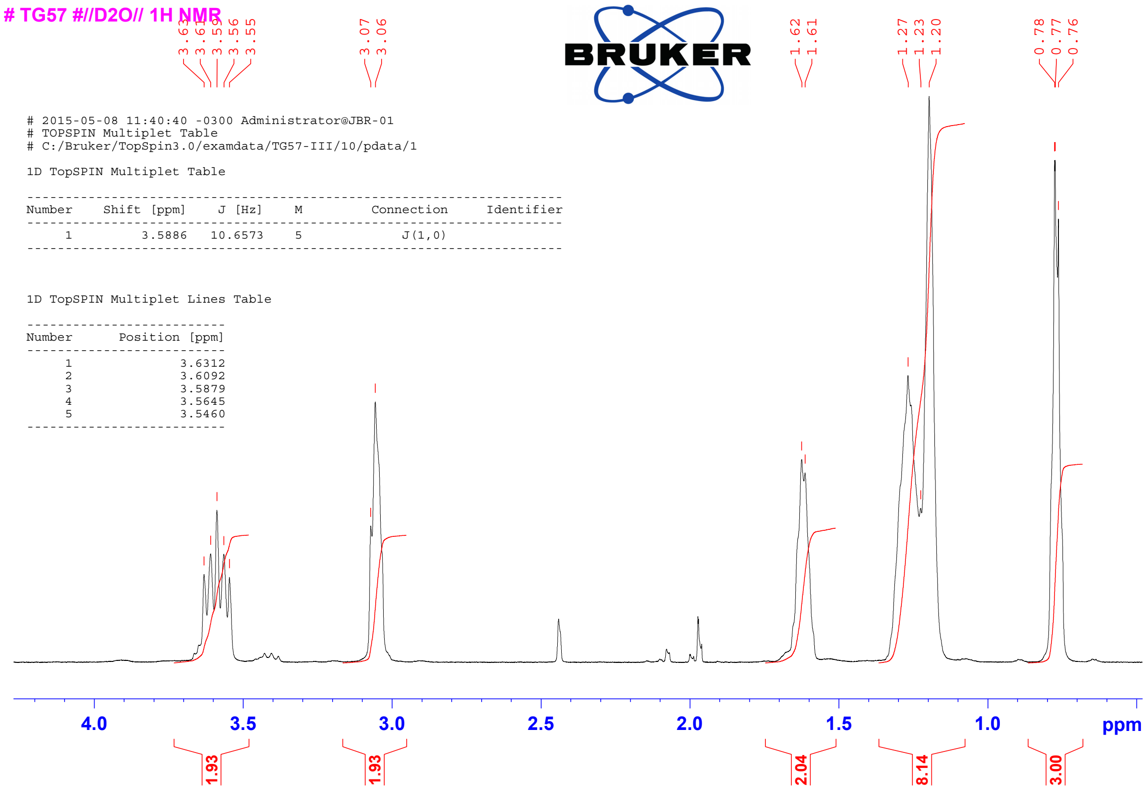

^{1}H NMR spectrum of compound **58**.

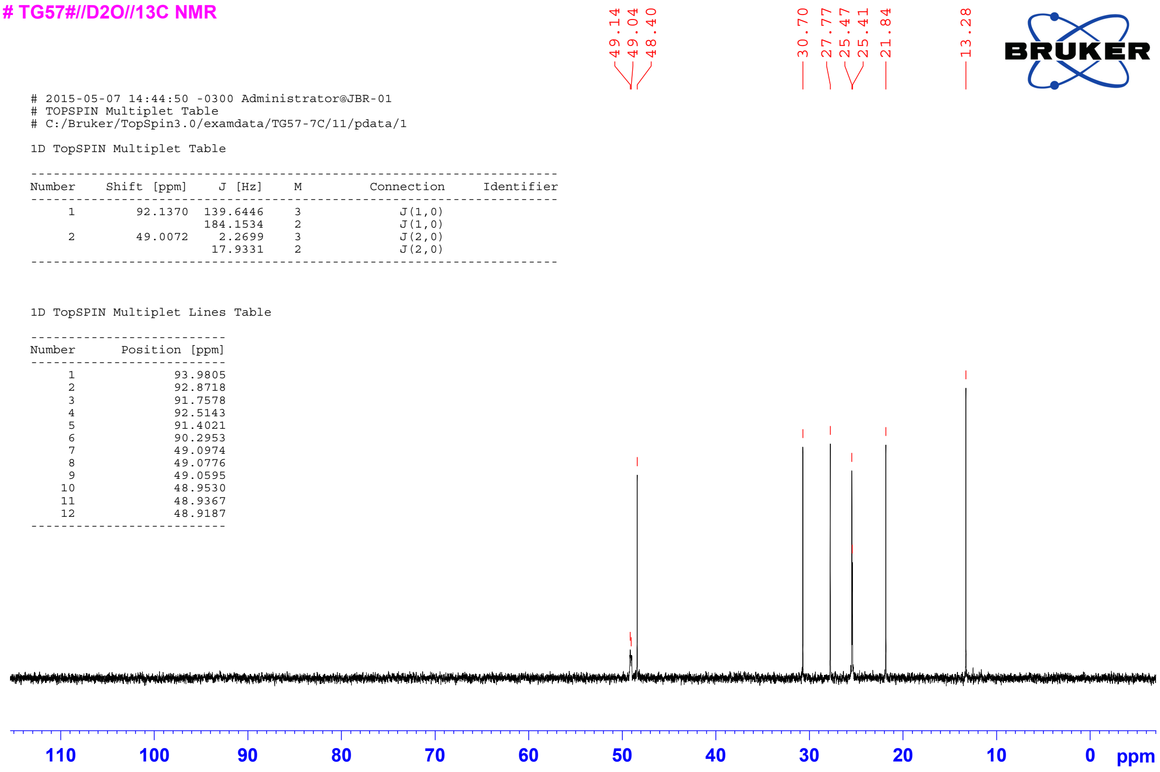

^{13}C NMR spectrum of compound **58**.

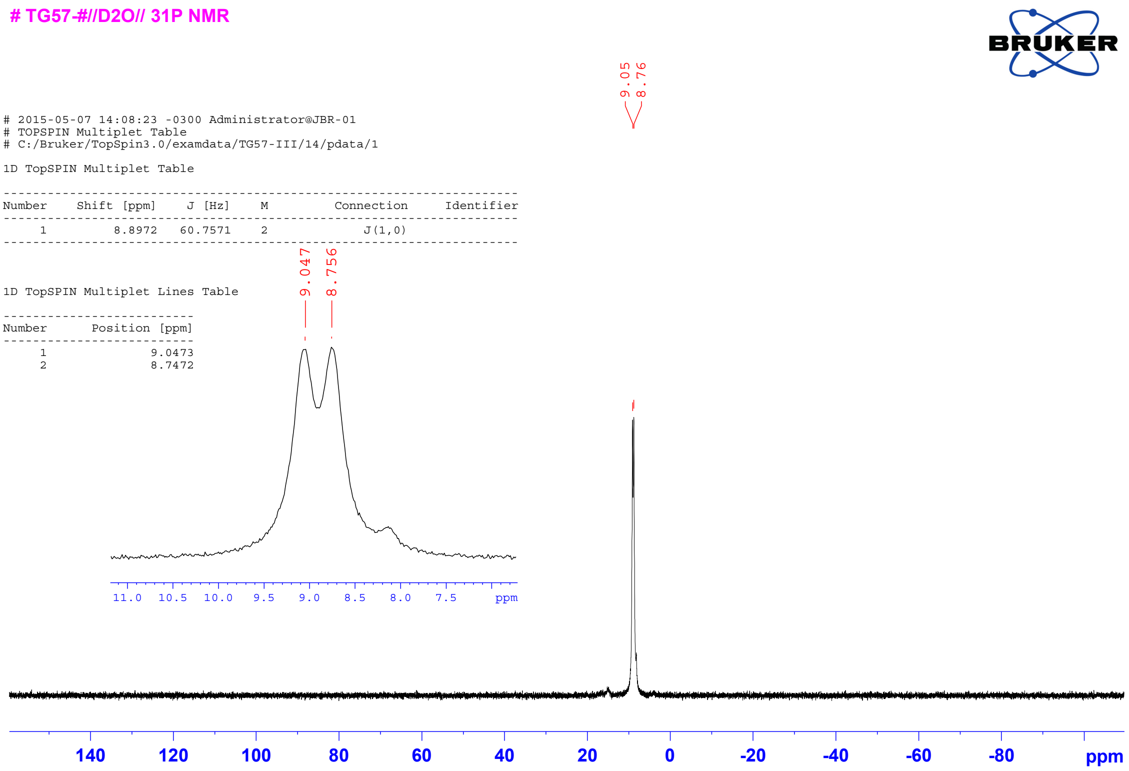

^{31}P NMR spectrum of compound **58**.

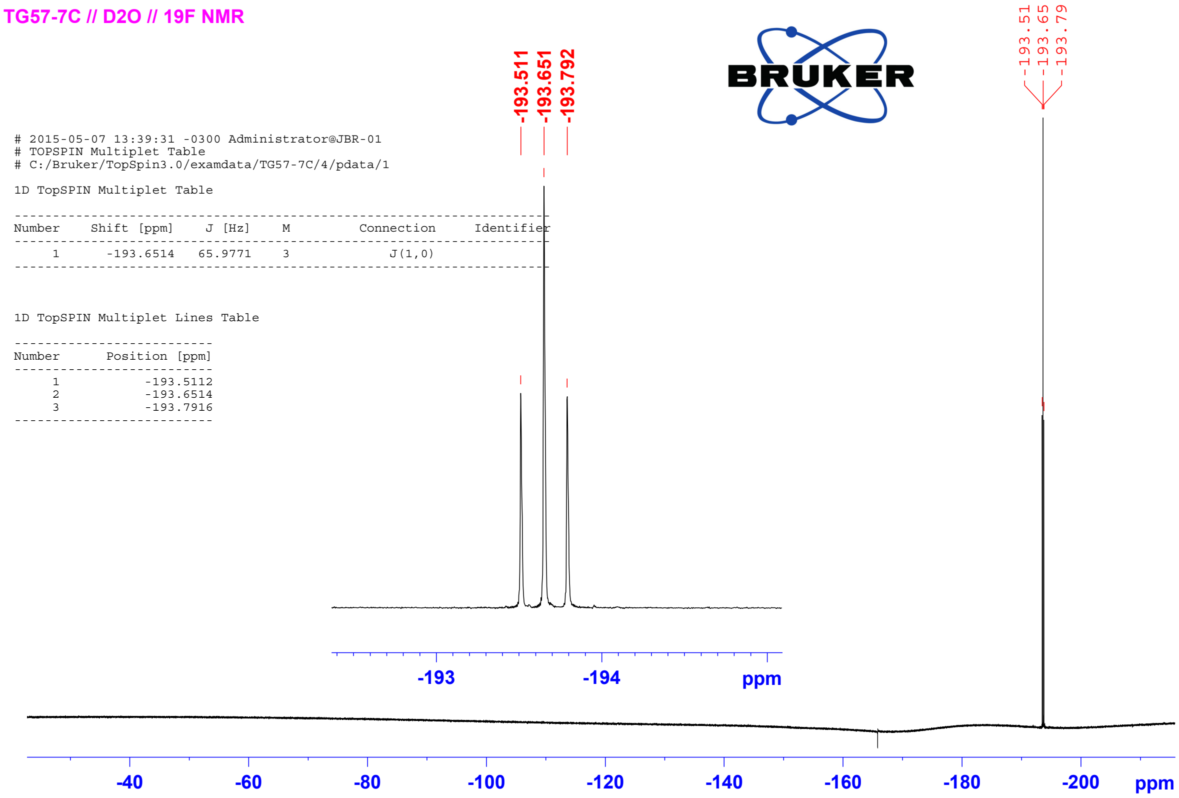

^{19}F NMR spectrum of compound **58**.

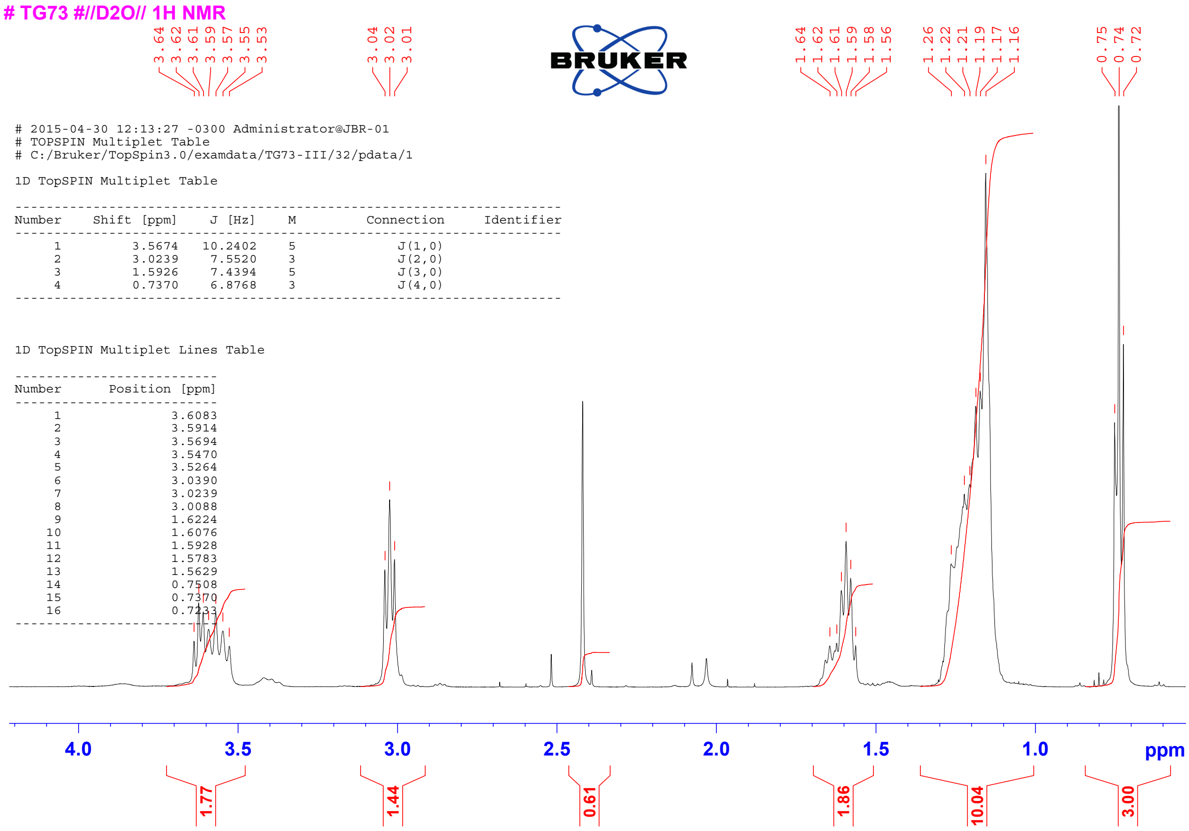

^{1}H NMR spectrum of compound **59**.

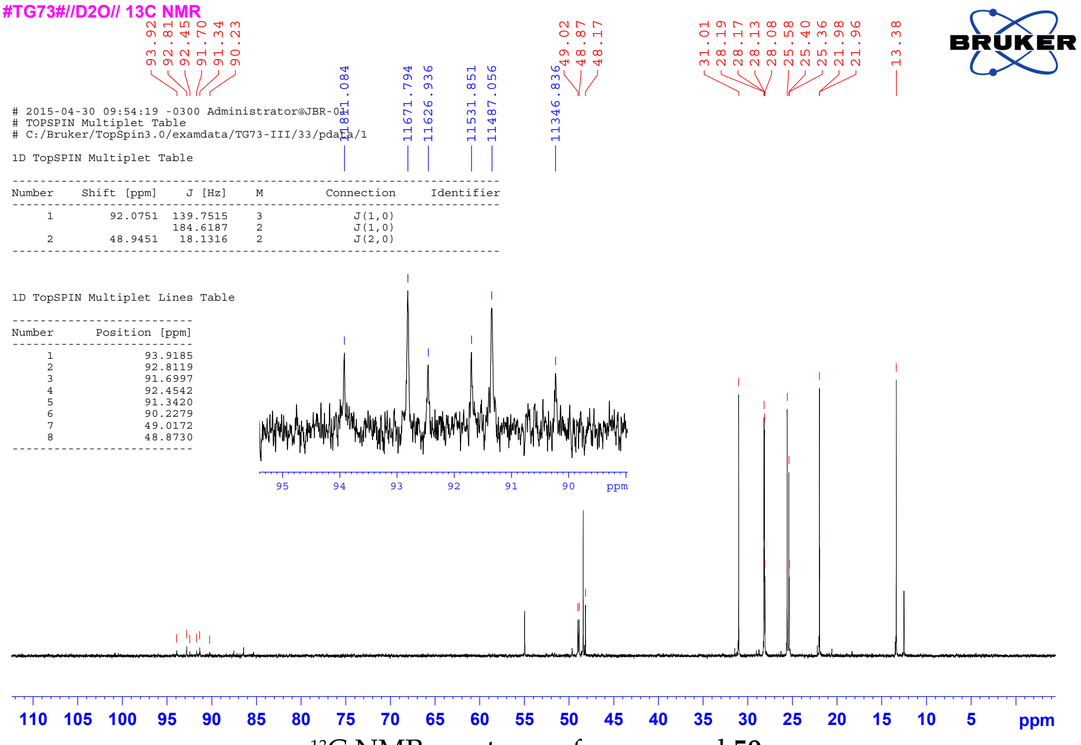

^{13}C NMR spectrum of compound **59**.

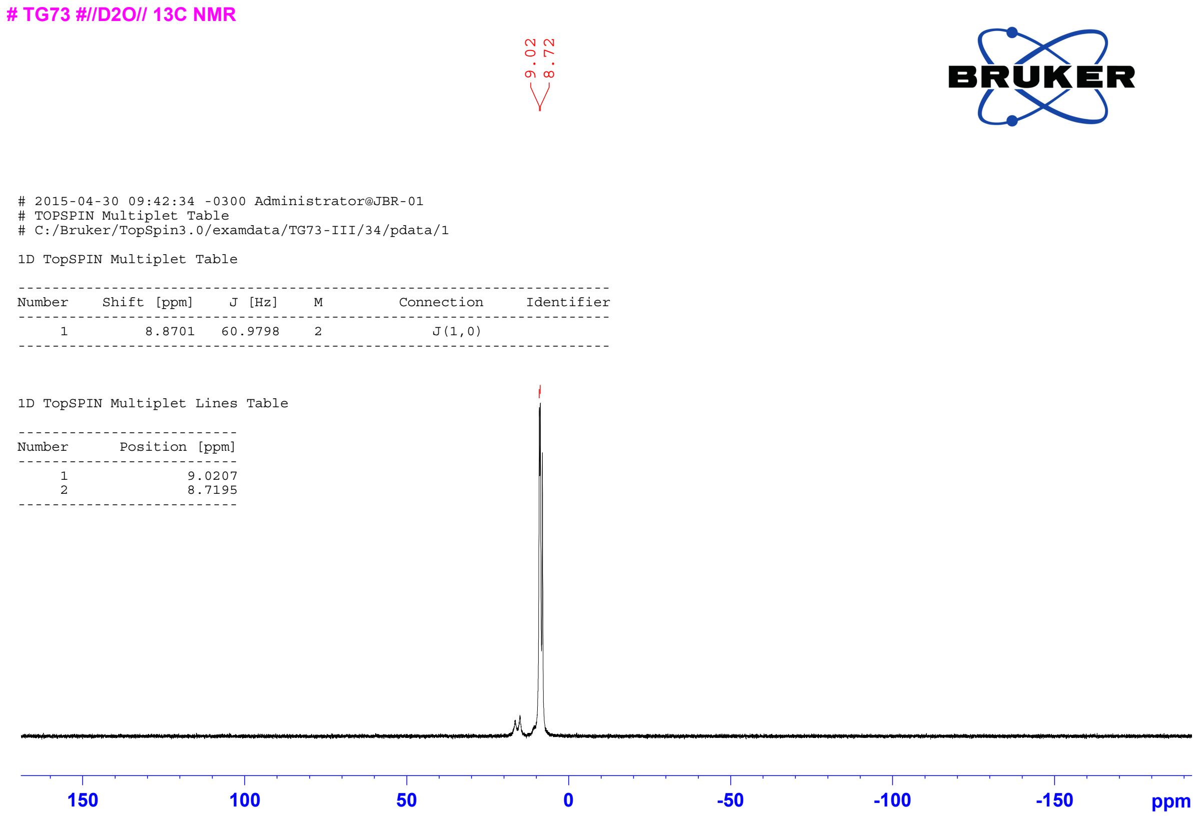

^{31}P NMR spectrum of compound **59**.

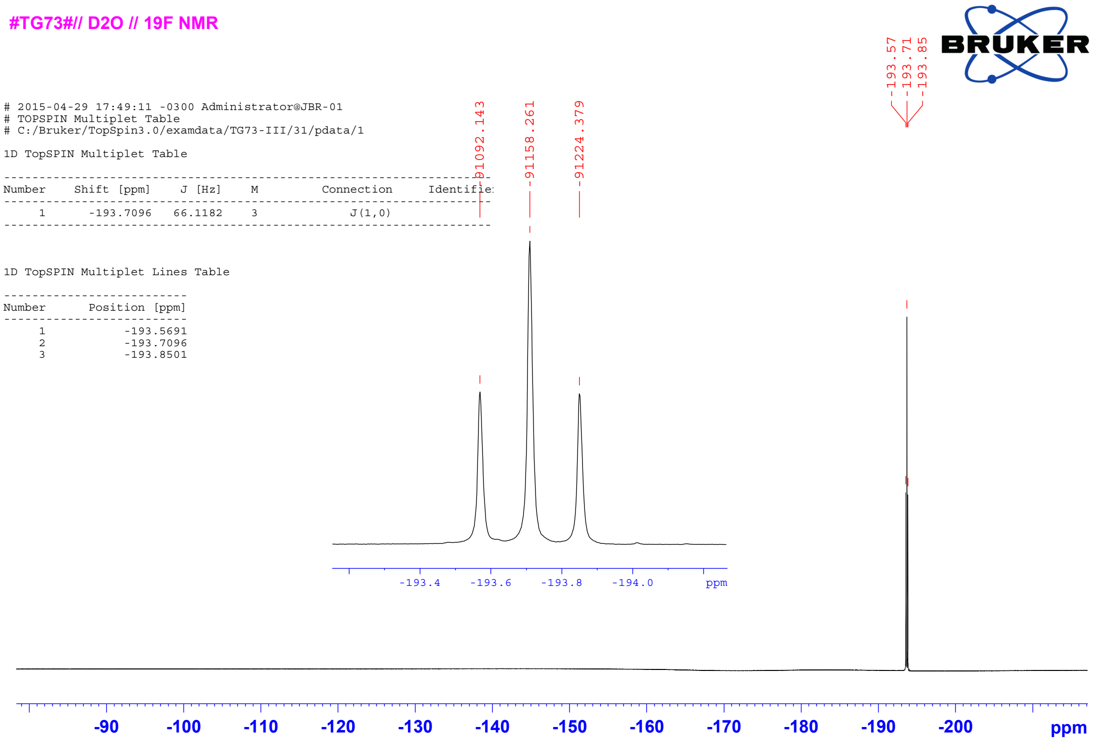

^{19}F NMR spectrum of compound **59**.

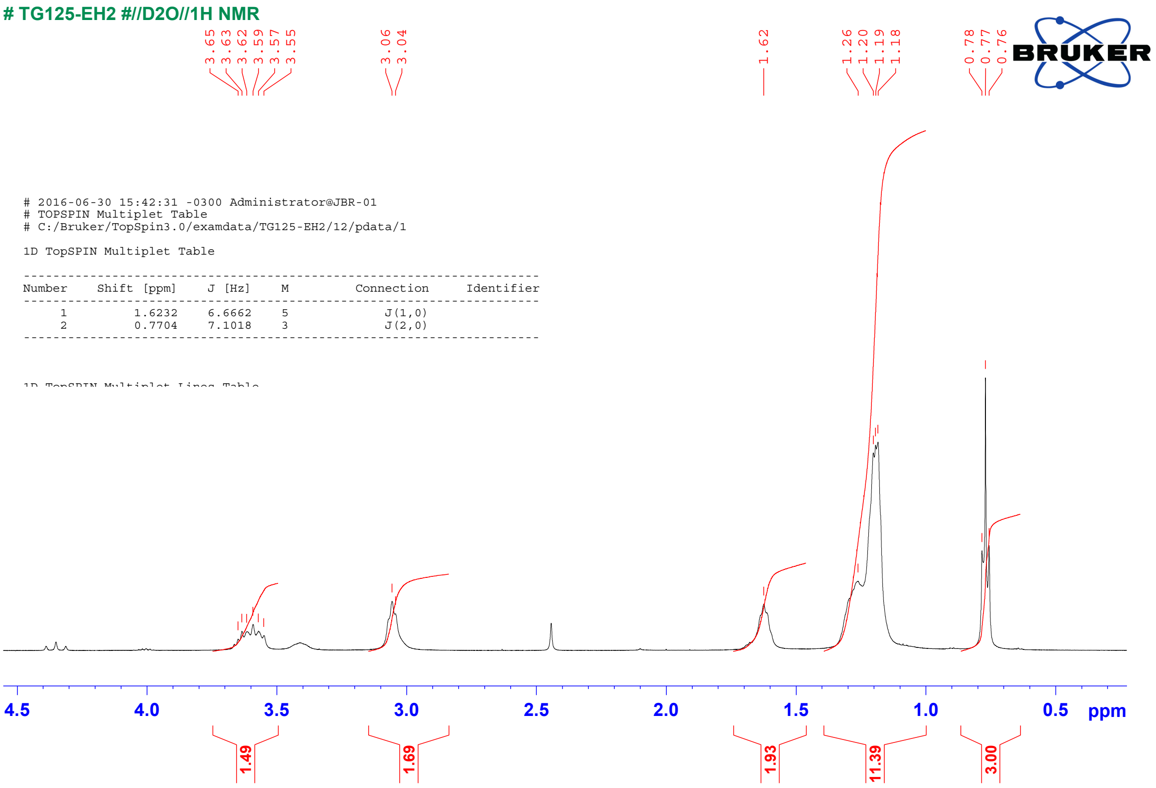

^{1}H NMR spectrum of compound **60**.

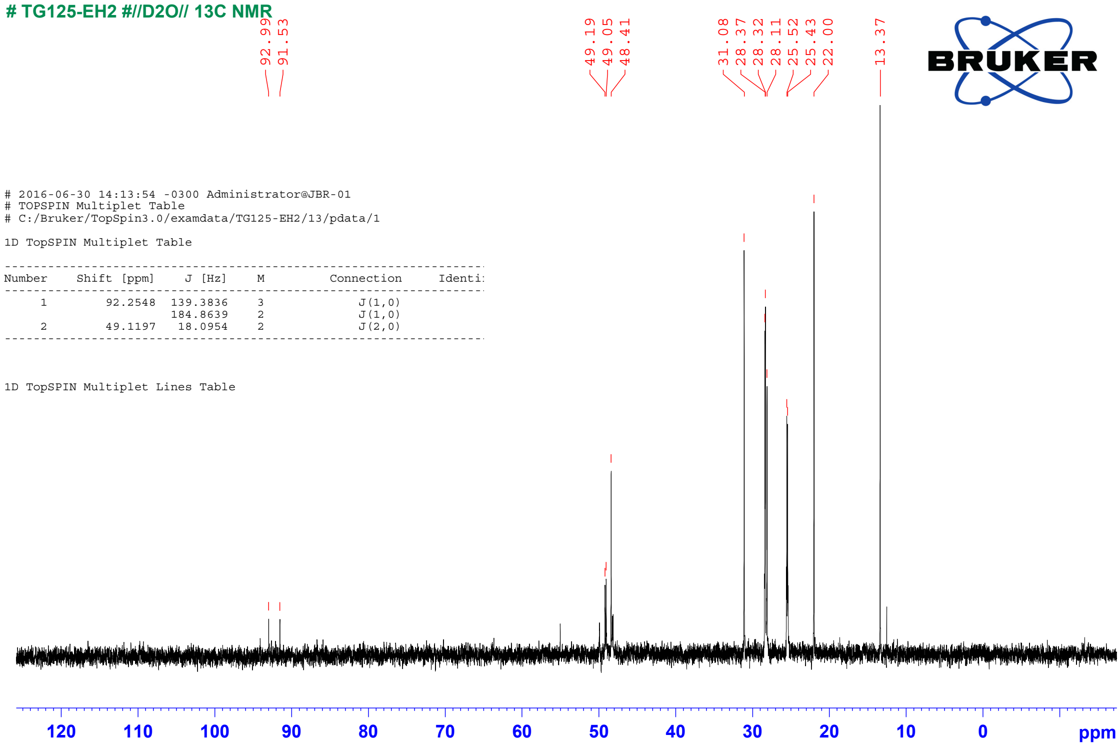

^{13}C NMR spectrum of compound **60**.

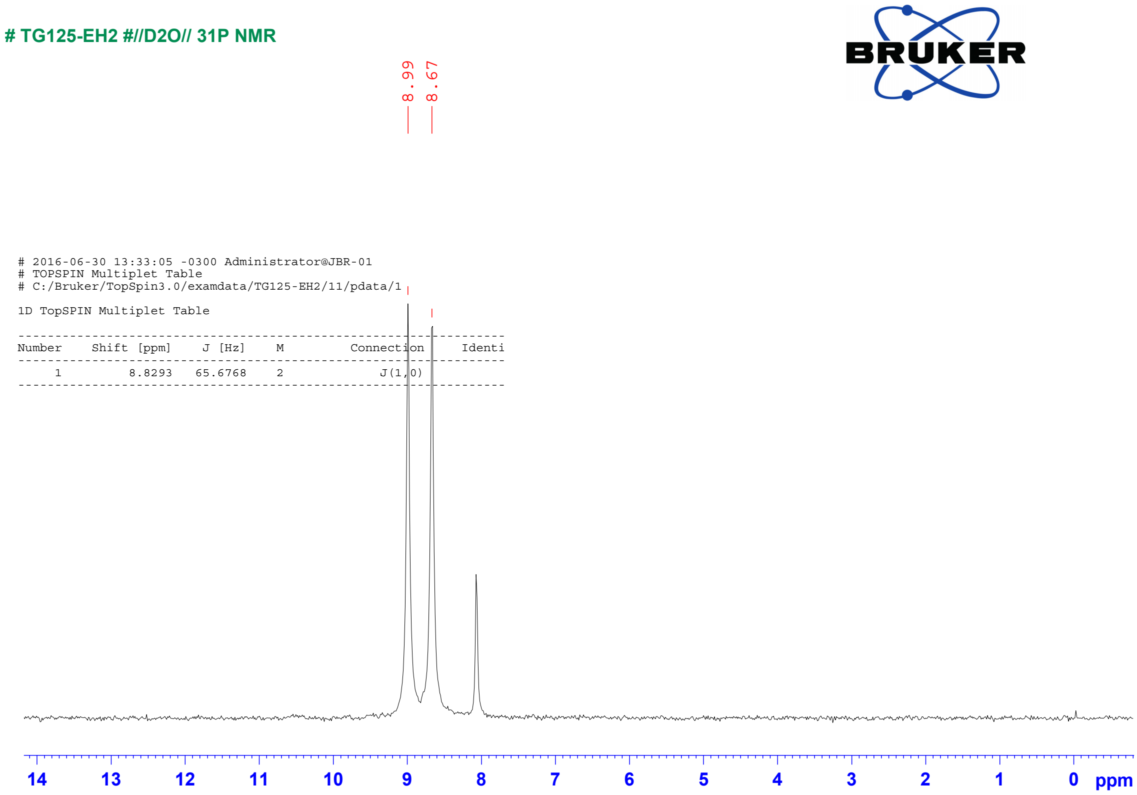

^{31}P NMR spectrum of compound **60**.

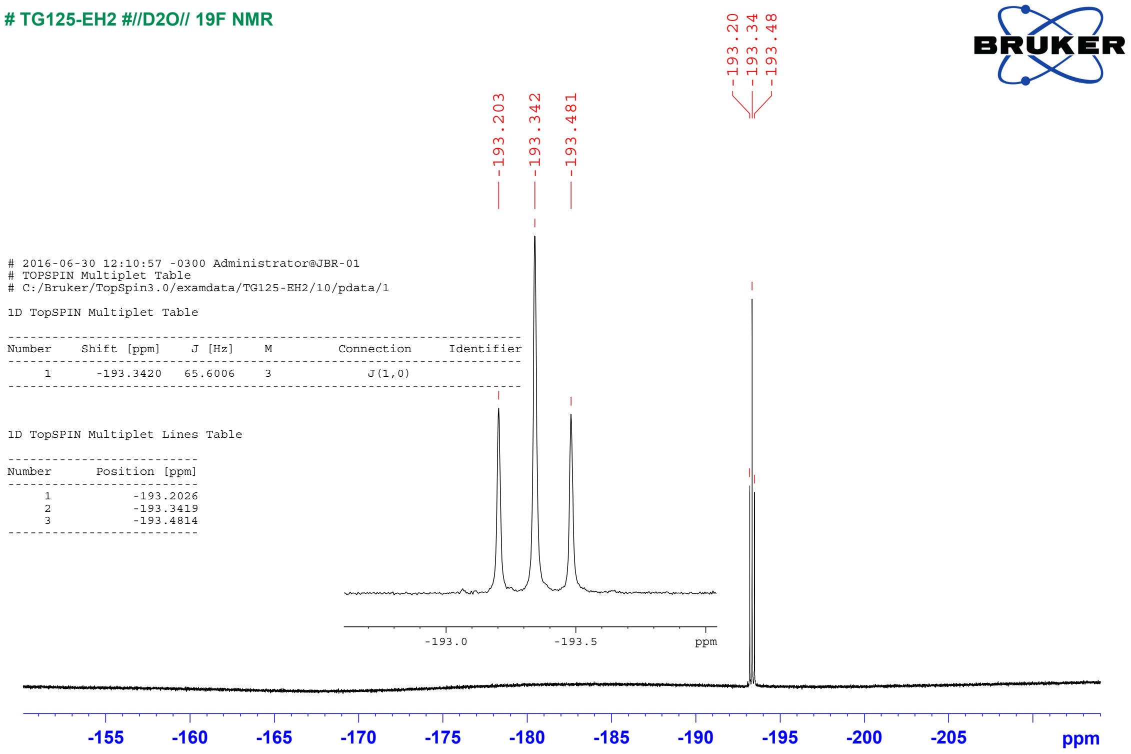

^{19}F NMR spectrum of compound **60**.

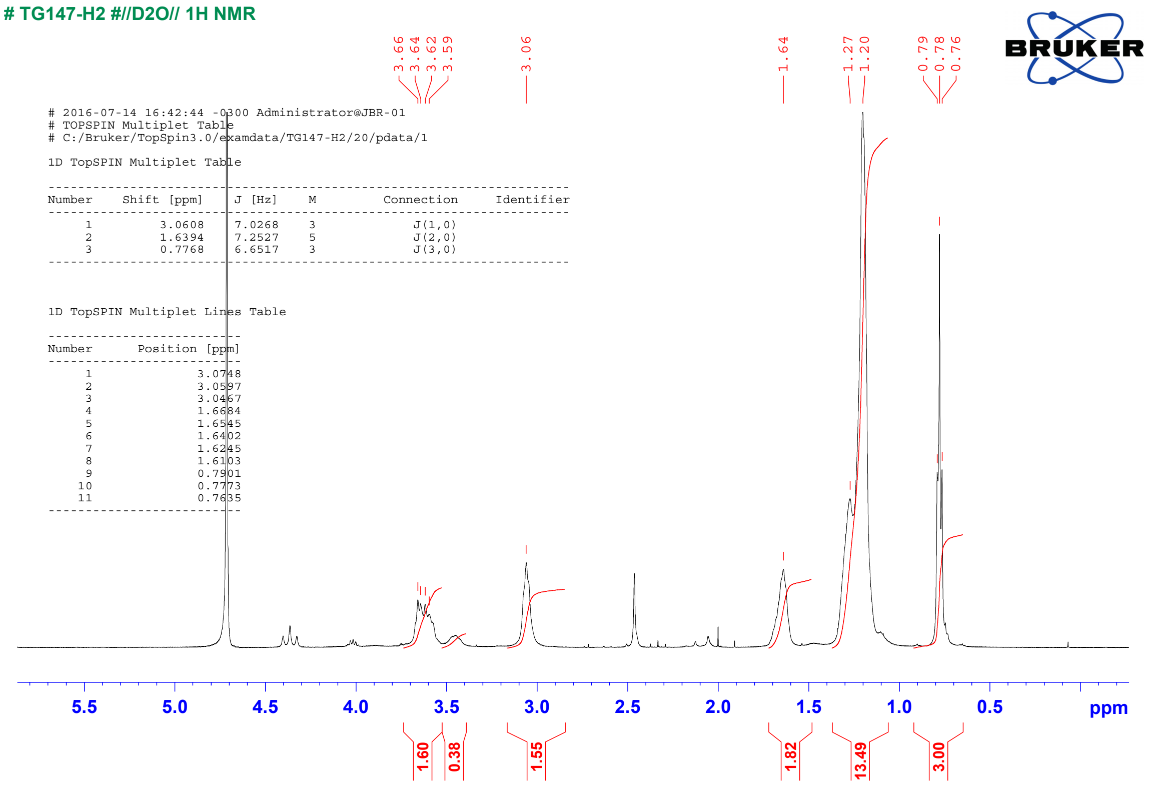

^{1}H NMR spectrum of compound **61**.

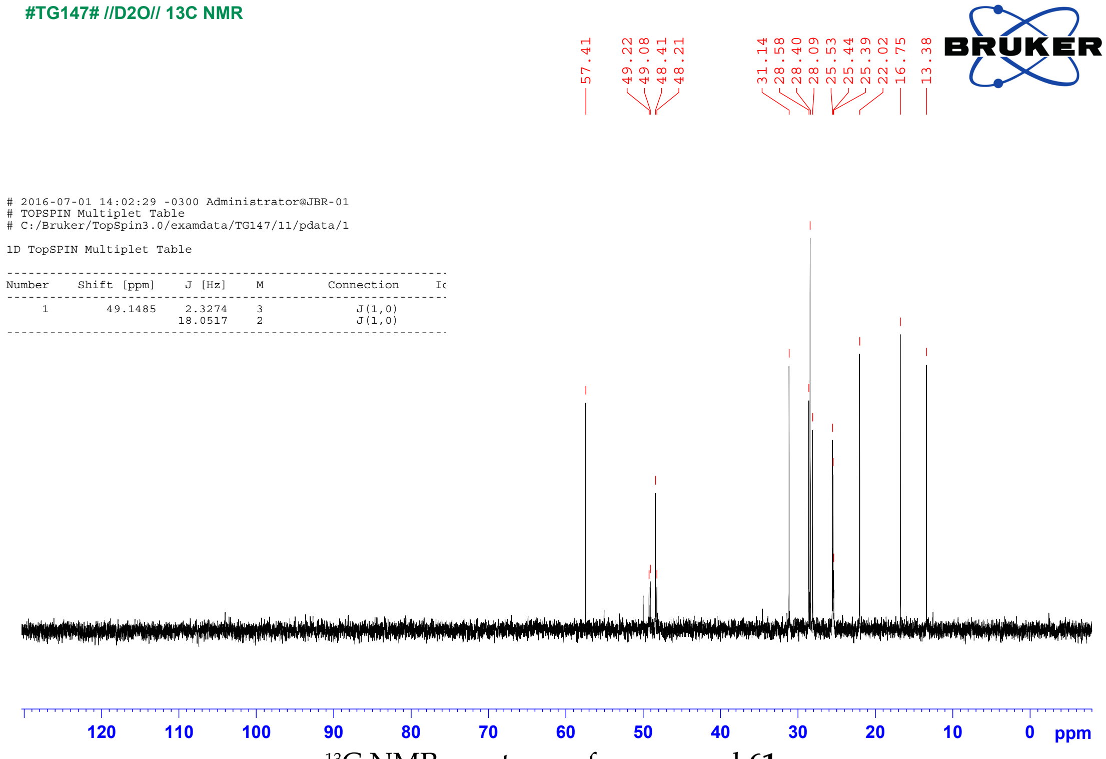

^{13}C NMR spectrum of compound **61**.

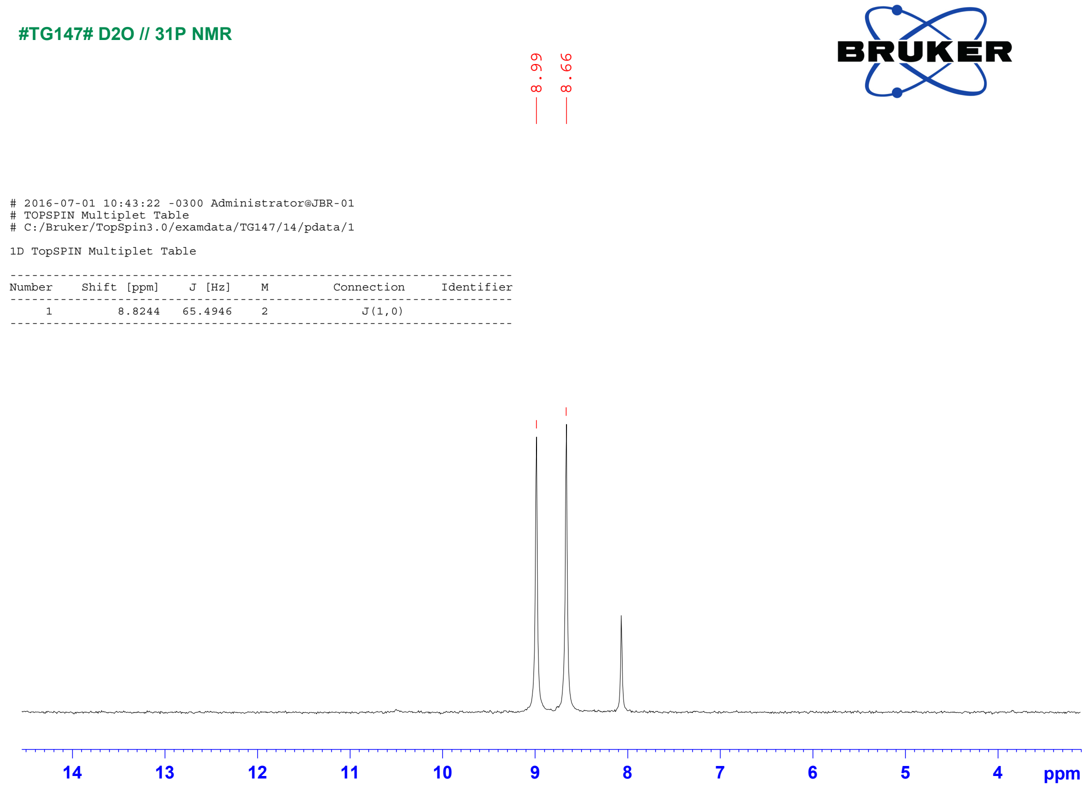

^{31}P NMR spectrum of compound **61**.

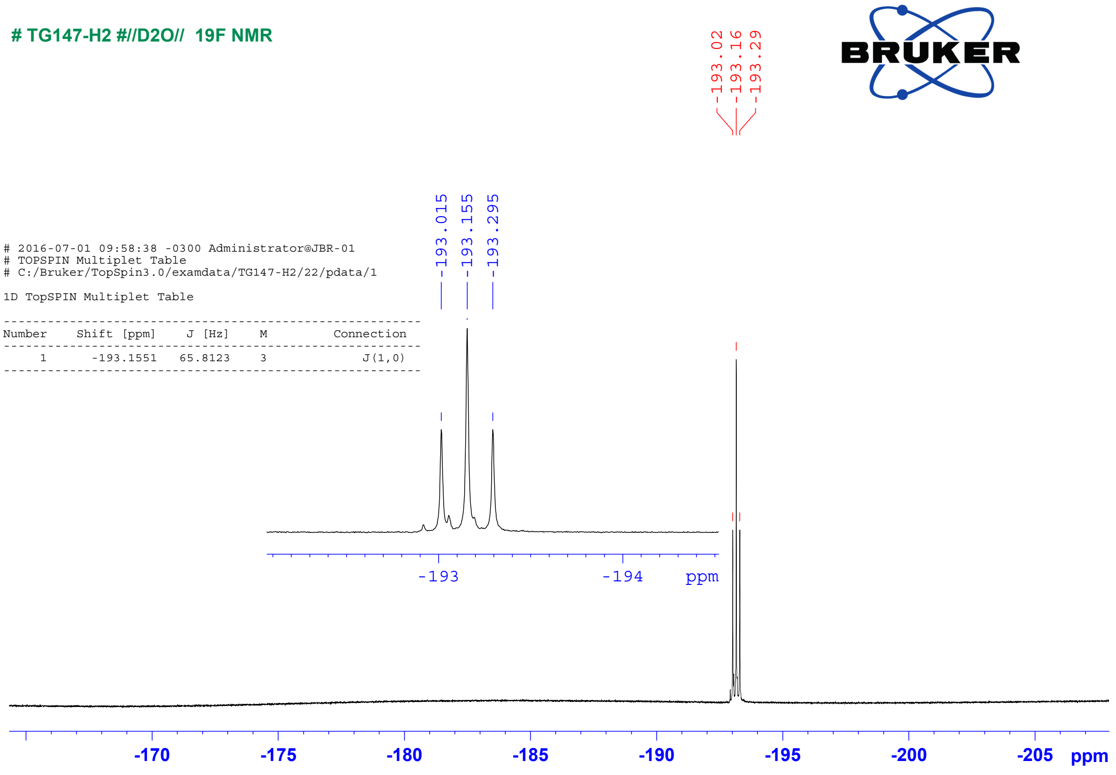

^{19}F NMR spectrum of compound **61**.
